# Click Chemistry-Enabled Parallel Synthesis of *N*-Acyl Sulfonamides and Their Evaluation as Carbonic Anhydrase Inhibitors

**DOI:** 10.3390/molecules31020318

**Published:** 2026-01-16

**Authors:** Oleksii V. Gavrylenko, Bohdan V. Vashchenko, Vasyl Naumchyk, Bohdan S. Sosunovych, Oleksii Chuk, Oleksii Hrabovskyi, Olga Kuchuk, Alla Pogribna, Sergiy O. Nikitin, Anzhelika I. Konovets, Volodymyr S. Brovarets, Sergey A. Zozulya, Dmytro S. Radchenko, Oleksandr O. Grygorenko, Yurii S. Moroz

**Affiliations:** 1 Enamine Ltd., Winston Churchill Street 78, 02094 Kyїv, Ukraine; 2 V. P. Kukhar Institute of Bioorganic Chemistry and Petrochemistry, National Academy of Sciences of Ukraine, Akademik Kukhar Street 1, 02066 Kyїv, Ukraine; 3 Taras Shevchenko National University of Kyiv, Volodymyrska Street 60, 01601 Kyїv, Ukraine; 4 Enamine Scientific Research Institute, Winston Churchill Street 67, 02094 Kyїv, Ukraine; 5 Chemspace LLC, Winston Churchill Street 85, 02094 Kyїv, Ukraine; 6 Palladin Institute of Biochemistry, National Academy of Sciences of Ukraine, Leontovycha Street 9, 01054 Kyїv, Ukraine; 7 Enamine Biology (Bienta)/Enamine Ltd., Winston Churchill Street 78, 02094 Kyїv, Ukraines.zozulya@enamine.net (S.A.Z.); 8 Institute of Molecular Biology and Genetics, National Academy of Sciences of Ukraine, Zabolotnogo Street 150, 03143 Kyїv, Ukraine

**Keywords:** combinatorial chemistry, organosulfur compounds, click chemistry, carbonic anhydrase, chemical space

## Abstract

A synthetically accessible library of *N*-acyl sulfonamides was constructed using a combination of copper(I)-catalyzed azide–alkyne cycloaddition (CuAAC) and *N*-acylation of primary sulfonamides. The proposed two-step reaction sequence had a high experimentally confirmed synthetic success rate (up to 85%) and gave reasonable product yields (up to 61%). As a result of the validation process, a 262-member compound library (out of >70K accessible combinations) was prepared. Biological profiling of the synthesized library by differential scanning fluorimetry and enzymatic assays identified several low micromolar inhibitors of human carbonic anhydrase. The interaction of the discovered hits with the biological target was studied by docking and molecular dynamics.

## 1. Introduction

**Scheme 1:**
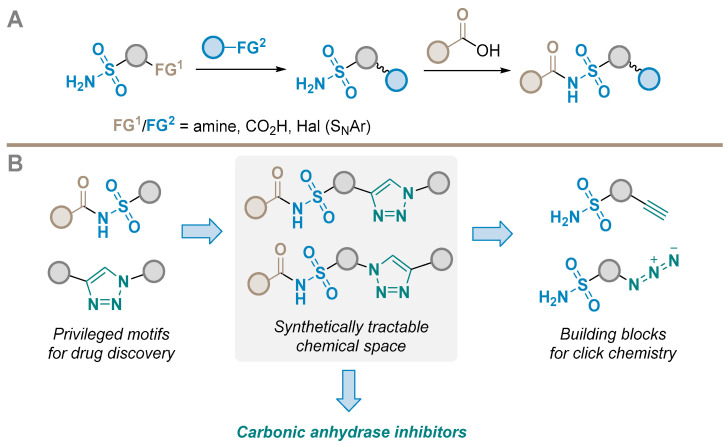
(**A**) Reported approach to the generation of *N*-acyl sulfonamide chemical space. (**B**) Design of this work.

The *N*-Acyl sulfonamide motif has been widely exploited in drug discovery, which is illustrated by numerous approved drugs and drug candidates. Notably, nine newly developed *N*-acyl sulfonamide therapeutics have reached the U.S. market within the last years [1]. Merging amide and sulfonamide groups—two very popular structural elements in medicinal chemistry—into a single moiety can exhibit notable metabolic stability and enhanced binding affinity to biological targets [2,3,4]. *N*-Acyl sulfonamides were suggested as bioisosteres of carboxylic acids, having similar acidity and hydrogen bonding patterns [5]. While such acidic (i.e., anionic at physiological pH) moieties are significant for drug design, they were shown to be vastly underrepresented in the current compound collections [6,7]. A rare example of methodology covering a substantial fraction of theoretically possible *N*-acyl sulfonamide chemical space, which relied on several simple two-step reaction sequences, i.e., amine *N*-acylation or *N*-arylation followed by *N*-acylation of the primary sulfonamide, was recently reported by our group (Scheme 1A) [8].

A promising strategy for chemical space exploration is based on the Nobel Prize-winning click chemistry, copper(I)-catalyzed azide-alkyne cycloaddition (CuAAC), being its most prominent example [9,10,11,12]. Its broad substrate scope, excellent yields, mild conditions, and selectivity in the formation of 1,4-disubstituted 1,2,3-triazoles make it well-suited for efficient molecule construction, easily producing high-purity compounds and thus having a great potential to accelerate drug discovery [13,14,15]. The resulting triazoles are metabolically stable aromatic heterocycles capable of mimicking amide or other similar moieties.

**Figure 1:**
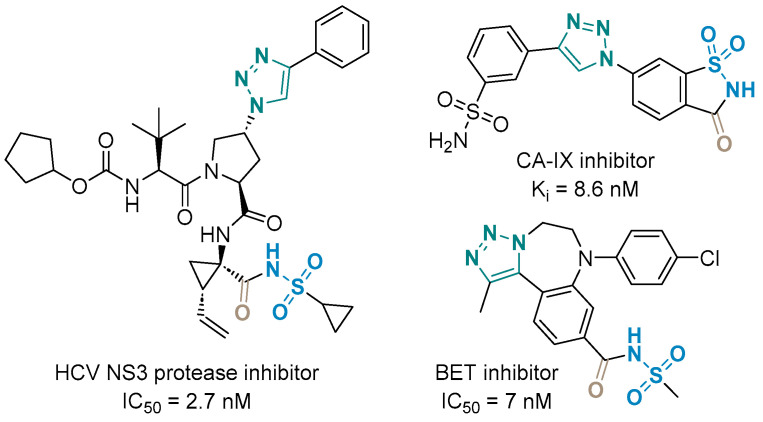
Examples of biologically active compounds having both *N*-acyl sulfonamides and 1,2,3-triazole moieties.

While both *N*-acyl sulfonamides and 1,2,3-triazoles have been represented in drug discovery programs, a combination of these two privileged motifs has remained largely unexplored, according to PubChem [16] and ChEMBL [17] data. Successful examples of this combination include hepatitis C virus (HCV) NS3 protease inhibitors [18], carbonic anhydrase IX (CA-IX) inhibitors [19], or bromodomain-containing protein 4 (BRD4) inhibitors [20] (Figure 1). To address the gap of 1,2,3-triazole-containing *N*-acyl sulfonamides in the accessible chemical space, we designed and synthesized two compound libraries by combining CuAAC with primary sulfonamide *N*-acylation (Scheme 1B). The resulting *N*-acyl sulfonamide libraries were implemented into the discovery of carbonic anhydrase inhibitors through a combination of differential scanning fluorimetry screening, enzymatic assay, docking, and molecular dynamics. Carbonic anhydrases were also widely studied in anticancer research; they are also involved in the treatment of glaucoma, periodic paralysis, idiopathic intracranial hypertension, cystinuria, and various neurological disorders [21,22,23,24,25,26,27,28,29,30].

Throughout this work, the compound numeration followed the convention commonly used in combinatorial chemistry. Thus, if compound library **4** was synthesized using three reactant sets **1**, **2**, and **3**, then the specific reactants were labeled as **1**{*i*}, **2**{*j*}, and **3**{*k*}, and the library member obtained from them—**4**{*i*,*j*,*k*}. To evaluate the parallel synthesis outcome, synthesis success rate (SSR), i.e., a percentage of experiments that led to isolation of the target library member among all planned experiments, was used along with the average product yield.

## 2. Results and Discussion

### 2.1. Parallel Synthesis

**Scheme 2:**
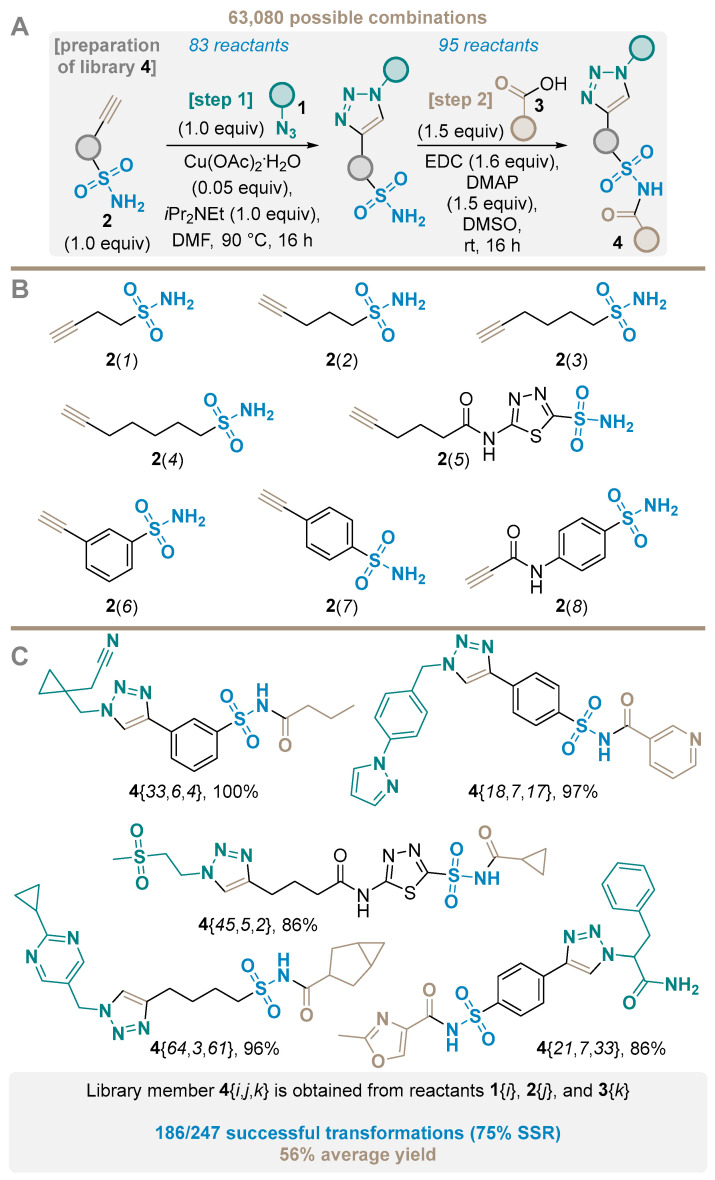
Synthesis of *N*-acyl sulfonamides 4: (**A**) the reaction sequence; (**B**) structures of alkyne sulfonamide building blocks 2; (**C**) representative examples of products 4 and the method outline.

Firstly, alkyne building blocks **2**{*1*–*8*} bearing a primary sulfonamide moiety were used as the starting points for the construction of compound library **4** (Scheme 2A,B). Compounds **1**{*1*–*83*} were introduced into the CuAAC with terminal alkynes **2** under the Cu(OAc)_2_—*i*-Pr_2_NEt catalysis in DMF at 90 °C to afford the corresponding triazole intermediates that were directly acylated with carboxylic acids **3**{*1*–*95*} using EDC and DMAP in DMSO at rt, finally giving the target library members **4**.

Overall, 247 library members **4**{*1*–*8*,*1*–*83*,*1*–*95*} out of 63,080 theoretically possible reactant combinations were deliberately selected for the parallel synthesis. In turn, 186 compounds (out of 247 experiments) were successfully obtained, which corresponds to an overall synthesis success rate of 75% and an average isolated yield of 56%. Selected examples of products **4** are given in Scheme 2C.

**Scheme 3:**
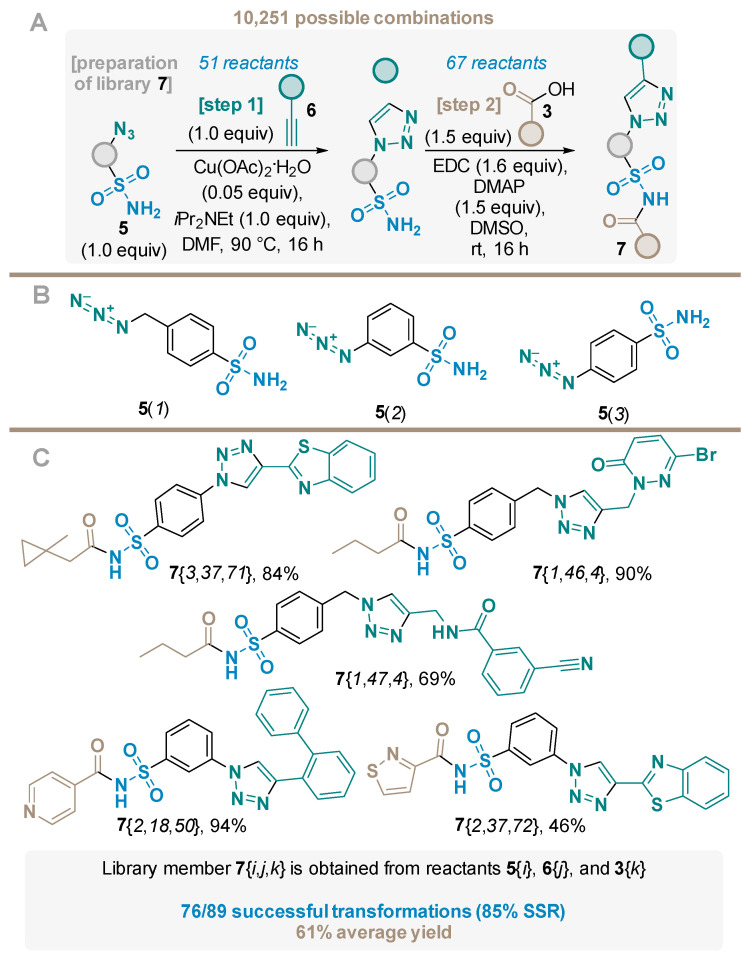
Synthesis of *N*-acyl sulfonamides 7: (**A**) the reaction sequence; (**B**) structures of azido sulfonamide building blocks 5; (**C**) representative examples of products 7 and the method outline.

Analogously, compound library **7** was constructed starting from azido sulfonamides **5**{*1*–*3*} that underwent CuAAC with terminal alkynes **6**{*1*–*51*} followed by treatment with the selected 67 carboxylic acids **3**{*1*–*77*} mentioned above (Scheme 3A,B). As a result, 76 out of 89 deliberately selected library members **7**{*1*–*3*,*1*–*51*,*1*–*77*} (from 10,251 theoretically possible reactant combinations) were isolated, which gives the overall synthesis success rate of 85% and an average isolated yield of 61%. Selected examples of products **7** are shown in Scheme 3C.

### 2.2. Differential Scanning Fluorimetry

**Figure 2:**
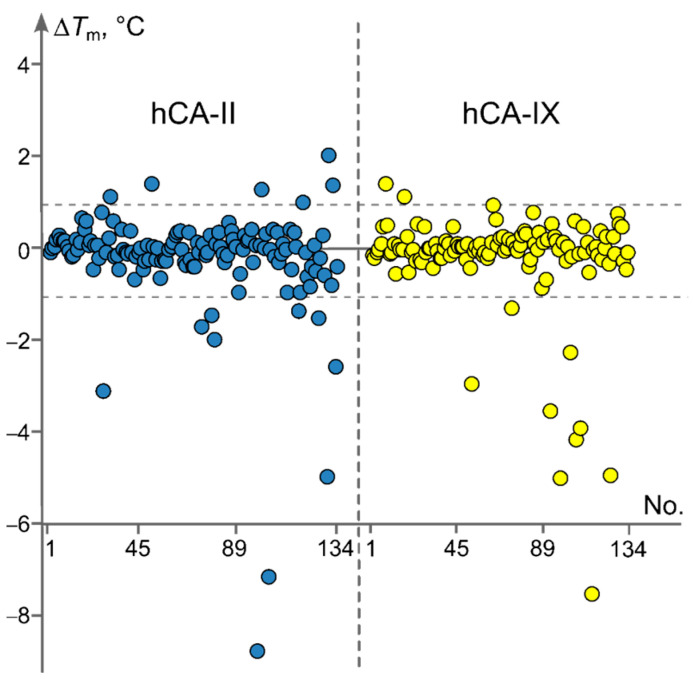
Thermal shifts (Δ*T*_m_) of hCA-II and hCA-IX at 40 μM compound concentration vs. compound number.

Considering our previous results on the inhibition of carbonic anhydrases with *N*-acyl sulfonamides [8], we have aimed at the evaluation of the synthesized compound libraries against two isoforms of human carbonic anhydrases, i.e., recombinant hCA-IX (catalytic domain) and hCA-II (full-length protein). In this way, a set of 134 deliberately selected *N*-acyl sulfonamides obtained in this work was screened in a thermal shift assay (TSA) to study the binding interactions. The screening against both hCA-II and hCA-IX recombinant proteins was conducted at 20 μM and 40 μM compound concentration (Figure 2; see the Supplementary Materials for more details). This primary screening was performed for the identification of compounds inducing significant thermal shifts indicative of target binding, i.e.,

|Δ*T*_m_| ≥ MED(Δ*T*_m_) + 3RSD(Δ*T*_m_),

where Δ*T*_m_—thermal shift for a library member, MED(Δ*T*_m_) and RSD(Δ*T*_m_)—median value and robust standard deviation, respectively, for the compounds screened in the same plate (excluding controls). A minimum thermal shift threshold was 0.5 °C to ensure meaningful interactions.

As a result, 15 primary hCA-IX hits, both positive (5 compounds) and negative shifters (10 compounds, normally considered as less productive) were selected for further hit analysis in the chromogenic enzymatic assay and the determination of IC_50_ values against both hCA-IX and hCA-II. The confirmation of the selected primary hits was performed under the same experimental conditions in quadruplicates.

### 2.3. Esterase Activity Inhibition Screening and IC_50_ Determination

**Figure 3:**
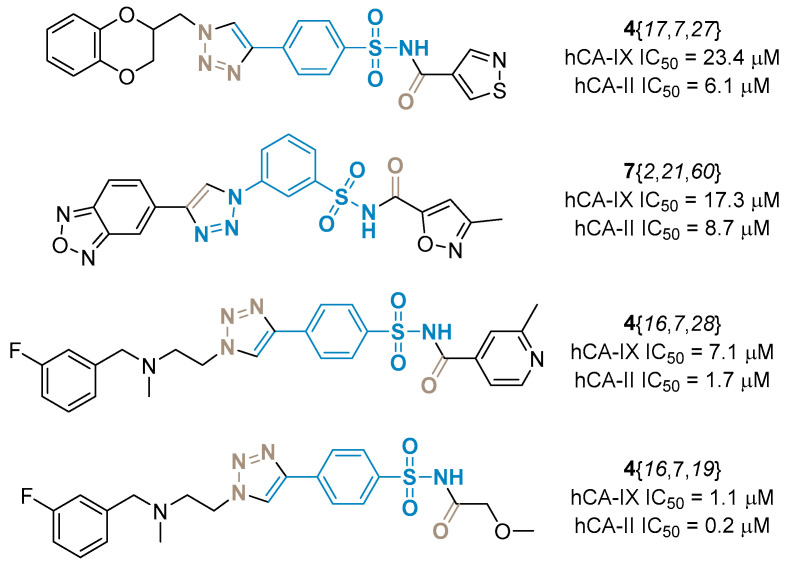
Compounds that demonstrated micromolar inhibition of hCA-II/hCA-IX.

The catalytic activity of hCA-II and hCA-IX was monitored through the enzymatic hydrolysis of *p*-nitrophenyl acetate. The compound concentration varied from 200 µM to 0.01 µM (titration of the investigated compounds at 10-point, 3× serial dilutions, *n* = 4) at a fixed concentration of hCA-II (200 nM) or hCA-IX (917 nM). As a result, four compounds were confirmed to be micromolar inhibitors of human carbonic anhydrases II and IX (Figure 3, Table 1; see Table S1 in the Supplementary Materials for more details).

Two derivatives, namely, **4**{*16*,*7*,*28*} and **4**{*16*,*7*,*19*}, demonstrated potency against both enzyme isoforms below 10 mM, with the lowest IC_50_ values of 0.2 μM and 1.1 μM for hCA-II and hCA-IX, respectively (for **4**{*16*,*7*,*19*}). All four compounds had marginal selectivity towards the hCA-II isoform, in contrast to the *N*-acyl sulfonamide libraries studied in the previous work [8]. All negative shifters observed in the thermal shift assay demonstrated no inhibition in all experiments with hCA-IX, which was not the case for hCA-II (Table S1 in Supplementary Materials).

### 2.4. In Silico Studies

Molecular docking studies were performed to elucidate the possible binding mechanisms for the identified *N*-acyl sulfonamide hits. X-ray diffraction crystal structures of hCA-II (PDB ID: 3K34 [31]) and hCA-IX (PDB ID: 6G9U [32]) were used to create receptor maps via superimposing protein structures, and removing water molecules and other small solutes from the binding site. The docking workflow utilized Molsoft ICM-Pro 3.9-3b [33], and a 5-Å radius around the co-crystallized ligands was defined as the active site. To improve the prediction accuracy, an atomic property field (APF) template derived from the sulfonamide group of the respective co-crystallized ligands was used to guide the ligand placement. Although the template was not enforced as a strict constraint during docking, it helped to efficiently identify the ligand conformation. The template location is provided in the Supplementary Materials, Figures S6 and S7. The results were evaluated using the ICM virtual ligand scoring (ICM-VLS) empirical function that incorporates multiple interaction energy terms including van der Waals, electrostatics, hydrogen bonding, and solvation energies.

All five analyzed compounds exhibited the typical sulfonamide group coordination to the zinc ion, as well as a hydrogen bond with T199 residue, in both CA-II and CA-IX models (Table 1). In the CA-II model, compounds **4**{*17*,*7*,*27*} and **4**{*18*,*7*,*32*} also formed π–π interactions with H94 residue through the isothiazole and thiophene rings, respectively. Meanwhile, compounds **7**{*2*,*21*,*60*} and **4**{*16*,*7*,*28*} formed additional hydrogen bonds with W5 and N62 residues, respectively. In addition to that, a series of hydrophobic interactions was also observed in both models (Table 2). These results suggest that some hCA-II selectivity observed for the most potent compounds can be related to the presence of the specific hydrogen bonds and π–π interactions mentioned above, whereas the hydrophobic contacts provide additional isoform-specific adaptations within the pocket. The reasons behind the high potency of compound **4**{*16*,*7*,*19*} are not clear.

Molecular dynamics simulations for the enzyme–ligand complexes were carried out using GROMACS v. 2023.2 [34] for 5 ns. The molecular mechanics/generalized Born (Poisson–Boltzmann) surface area (MM/GB(PB)SA) method was used to estimate binding free energy [35]. Four different sets of parameters were studied (see Table S1 in the Supplementary Materials). Two of the four studied parameters that are provided in Table 1 (parameter “9” (MM/GBSA method, implicit generalized Born parameter *igb* = 8, interior dielectric constant *intdiel* = 4.0) and parameter “12” (MM/PBSA method, *intdiel* = 4.0); a more negative value means stronger predicted binding affinity) correlated with IC_50_ values for hCA-II in most cases, with more negative values for inhibitors **7**{*2*,*21*,*60*}, **4**{*16*,*7*,*28*}, and **4**{*16*,*7*,*19*} (IC_50_ = 8.74 μM, 1.70 μM, and 0.20 μM, respectively), and less negative for compound **4**{*18*,*7*,*32*} which demonstrated no inhibition. At the same time, the model predicted the inhibition behavior of **4**{*17*,*7*,*27*} poorly (poor interaction energies were predicted by either parameter, while the compound had IC_50_ = 6.07 μM). Meanwhile, both molecular dynamics parameters showed only the possibility of a significant binding for all compounds studied in the case of hCA-IX, with no meaningful trends identified. Nevertheless, the confirmation of the resulting ligand–protein complex stability was achieved.

## 3. Materials and Methods

The solvents were purified according to the standard procedures [36]. All the starting materials were available from Enamine Ltd. (Kyїv, Ukraine) or purchased from other commercial sources. Melting points were measured on the MPA100 OptiMelt automated melting point system (Stanford Research Systems, Sunnyvale, CA, USA). ^1^H and ^13^C{H} spectra were recorded on a Bruker 170 Avance 500 spectrometer (Bruker, Billerica, MA, U. S.) (at 500 MHz for ^1^H NMR, and 126 MHz for ^13^C{H} NMR) and Varian Unity Plus 400 spectrometer (Varian, Palo Alto, CA, U. S.) (101 MHz for ^13^C{H} NMR). NMR chemical shifts are reported in ppm (δ scale) downfield from TMS as an internal standard and are referenced using residual NMR solvent peaks at 2.50 and 39.52 ppm for ^1^H and ^13^C{^1^H} in DMSO-*d_6_*. Coupling constants (*J*) are given in Hz. Spectra are reported as follows: chemical shift (δ, ppm), multiplicity, integration, and coupling constants (Hz). Elemental analyses were performed at the Laboratory of Organic Analysis, Department of Chemistry, Taras Shevchenko National University of Kyiv. Mass spectra were recorded on an Agilent 1100 LC-MSD SL instrument (Agilent, Santa Clara, CA, U. S.) (chemical ionization (CI)).

For the materials and methods related to in vitro and in silico screening, see the Supplementary Materials.

### General Procedure for the Preparations of Libraries 4 and 7 (CuAAC and Subsequent Acylation)

Step 1: Copper(I)-catalyzed azide–alkyne cycloaddition.

Azide (**1** or **5**, 1 mmol) and alkyne (**2** or **6**, 1 mmol) were added to a solution of *N*,*N*-diisopropylethylamine (1 mmol) and Cu(OAc)_2_.H_2_O (0.05 mmol) in DMF (1 mL), and the resulting mixture was stored at 90 °C overnight (ca. 16 h). Then, the solvent was evaporated in vacuo to afford crude intermediate that was used in the next step without purification.

Step 2: Acylation of Sulfonamides.

The crude resulting sulfonamide was dissolved in DMSO (1 mL). Then, carboxylic acid **3** (1.5 equiv.), EDC (1.6 equiv.), and DMAP (1.5 equiv.) were added to the solution, which was then stirred at rt overnight (ca. 16 h). After that, the solvent was evaporated in vacuo, and the resulting crude product (**4** or **7**) was subjected to HPLC purification.

## 4. Conclusions

In this work, we established a parallel synthesis workflow that relies on azido and alkynyl sulfonamides as the bifunctional click chemistry building blocks to rapidly generate structurally diverse 1,2,3-triazole-containing *N*-acyl sulfonamides. By integrating copper-catalyzed azide-alkyne cycloaddition and direct *N*-acylation into a two-step parallel workflow, we achieved the efficient construction of two representative compound libraries (186 and 76 members, respectively, out of 73,331 possible combinations) with overall synthesis success rates of 75–85% and average isolated yields of 56–61%.

Biological evaluation of the compounds obtained against human carbonic anhydrase isoforms hCA-II and hCA-IX was performed as an illustration of their potential for early drug discovery. Thermal shift assay screening resulted in the identification of 15 primary hits. Further orthogonal esterase activity assay revealed four micromolar hCA-II/IX inhibitors, and two of them exhibited submicromolar to low micromolar potency. All binders showed marginal selectivity toward the hCA-II isoform. Additionally, their binding modes were studied by docking and molecular dynamics, which showed a semi-qualitative correlation with the obtained experimental data. Thus, the coordination of the *N*-acyl sulfonamide group to the zinc ion and the hydrogen bond with the T199 residue were common for all compounds in the case of both enzyme isoforms, whereas additional hydrogen bonds or π–π interactions might be the reasons behind the observed marginal hCA-II selectivity.

These results demonstrate that the chemical space of triazole-containing *N*-acylsulfonamides can be a valuable source of biologically active compounds for bioorganic and medicinal chemistry, and, with increased synthetic and commercial accessibility, these compounds should increase their representation in early drug discovery programs.

## Figures and Tables

**Table 1:** Results of thermal shift assay, orthogonal colorimetric enzymatic activity assays (with IC_50_ determination), docking, and molecular dynamics for the most active compounds.

#	Compound	CA-II					hCA-IX				
		Δ*T*_m_, Median	IC_50_, µM	Docking Result	MM/GB(PB)SA, kcal/mol		Δ*T*_m_, Median	IC_50_, µM	Docking Result	MM/GB(PB)SA, kcal/mol	
					Parameter “9” ^a^	Parameter “12” ^b^				Parameter “9” ^a^	Parameter “12” ^b^
1	**4**{*17*,*7*,*27*}	N/A ^c^	6.07	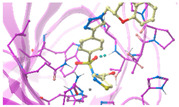	−43.59	−37.69	1.44	23.4	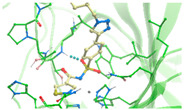	−68.09	−47.24
2	**7**{*2*,*21*,*60*}	N/A ^c^	8.74	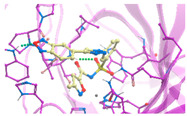	−70.35	−46.40	1.17	17.3	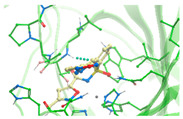	−61.09	−38.36
3	**4**{*16*,*7*,*28*}	−2.55	1.70	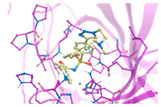	−66.01	−43.91	1.18	7.13	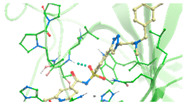	−60.09	−38.98
4	**4**{*16*,*7*,*19*}	−4.95	0.20	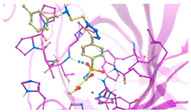	−67.26	−42.44	1.17	1.11	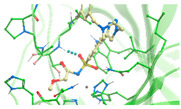	−52.6	−39.19
5	**4**{*18*,*7*,*32*}	N/A ^c^	>100	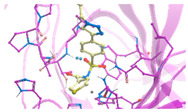	−36.74	−33.74	0.99	84.2 ^d^	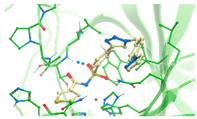	−65.25	−39.84

^a^ Internal parameter “9” was calculated using the MM/GBSA method with the following settings: implicit generalized Born parameter *igb* = 8, interior dielectric constant *intdiel* = 4. ^b^ Internal parameter “12” was calculated using the MM/PBSA method with the following settings: interior dielectric constant *intdiel* = 4. ^c^ N/A—the data are not available. ^d^ Inhibition at high concentration might be related to the low solubility or non-specific binding.

**Table 2:** Amino acid residues of hCA-II and hCA-IX participating in hydrophobic interactions with compounds **4**{*17*,*7*,*27*}, **7**{*2*,*21*,*60*}, **4**{*16*,*7*,*28*}, **4**{*16*,*7*,*19*}, and **4**{*18*,*7*,*32*}.

#	Compound	hCA-II Residues	hCA-IX Residues
1	**4**{*17*,*7*,*27*}	W5, F20, H64, A65, H96, V121, F131, L198, T200, P201, P202	H64, S65, H94, V121, V131, D132, L135, L198, T200, P202
2	**7**{*2*,*21*,*60*}	N62, H64, H96, V121, F131, L141, V143, L198, T200	N62, H64, S65, H96, V121, L198, T200, N244
3	**4**{*16*,*7*,*28*}	H64, A65, H96, V121, F131, G132, V135, Q136, L198, T200, P202, L204	H64, S65, T69, L91, Q92, H96, V121, V131, L198, T200
4	**4**{*16*,*7*,*19*}	F20, H64, A65, H94, H96, V121, F131, L198, T200, P201, P202	H64, S65, L91, Q92, H96, V121, V131, L135, L198, T200, P202
5	**4**{*18*,*7*,*32*}	W5, L60, H64, A65, E69, I91, Q92, H94, H96, V121, L198, T200	H64, S65, T69, L91, Q92, H96, V121, L123, R130, V131, L198, T200

## Data Availability

The data presented in this study are available in the article’s Supplementary Materials.

## Supplementary Materials

The following supporting information can be downloaded at https://www.mdpi.com/article/10.3390/molecules31020318/s1. Refs. [37,38,39,40,41] are cited in the Supplementary Materials.
