# Supplementary material for: Click Chemistry-Enabled Parallel Synthesis of N-Acyl Sulfonamides and Their Evaluation as Carbonic Anhydrase Inhibitors
Source: Molecules. 2026 Jan 16;31(2):318. doi: 10.3390/molecules31020318 (PMC12844257; doi:10.3390/molecules31020318)
Supplement: Supplementary file 1 [file molecules-31-00318-s001.zip › molecules-4021383-supplementary.pdf]

Click-Chemistry-Enabled Parallel Synthesis of *N*-Acyl Sulfonamides and Their Evaluation as Carbonic Anhydrase Inhibitors (Supporting Information)

Oleksii V. Gavrylenko,[a,b] *Bohdan V. Vashchenko,*[c,d] *Vasyl Naumchyk,*[a,c] *Bohdan S. Sosunovych,*[a] *Oleksii Chuk,*[e,f] *Oleksii Hrabovskyi,*[e,f] *Olga Kuchuk,*[g] *Alla Pogribna,*[g,h] *Sergiy O. Nikitin,*[c,g] *Anzhelika I. Konovets,*[a] *Volodymyr S. Brovarets,*[b] *Sergey A. Zozulya,*[g] *Dmytro S. Radchenko,*[a] *Oleksandr O. Grygorenko,*[a,c,d]* *Yurii S. Moroz*[a,c,e]*

[a] Enamine Ltd. (www.enamine.net), Winston Churchill Street 78, Kyïv 02094, Ukraine

[b] V. P. Kukhar Institute of Bioorganic Chemistry and Petrochemistry, National Academy of Sciences of Ukraine, Akademik Kukhar Street 1, Kyïv 02066, Ukraine

[c] Taras Shevchenko National University of Kyiv, Volodymyrska Street 60, 01601, Kyïv, Ukraine

[d] Enamine Scientific Research Institute, Winston Churchill Street 67, Kyïv 02094, Ukraine

[e] Chemspace LLC (www.chem-space.com), Winston Churchill Street 85, Kyïv, 02094, Ukraine

[f] Palladin Institute of Biochemistry, National Academy of Sciences of Ukraine, Leontovycha Street 9, Kyïv 01054, Ukraine

[g] Enamine Biology (Bienta)/Enamine Ltd. (www.bienta.net), Winston Churchill Street 78, Kyïv 02094, Ukraine

[h] Institute of Molecular Biology and Genetics, National Academy of Sciences of Ukraine, Zabolotnogo Street 150, Kyïv 03143, Ukraine

Table of Contents

In vitro screening experimental details S5

hCA esterase activity inhibition screening assay (with IC_{50} determination) S8

In silico screening details S17

Characterization data for the synthesized compounds S19

Parallel synthesis details S27

N-((3-(1-((2-Ethylthiazol-4-yl)methyl)-1*H*-1,2,3-triazol-4-yl)phenyl)sulfonyl)nicotinamide (4{32,6,17}) ^{1}H NMR S34

N-((3-(1-((2-Ethylthiazol-4-yl)methyl)-1*H*-1,2,3-triazol-4-yl)phenyl)sulfonyl)nicotinamide (4{32,6,17}) ^{13}C NMR S35

N-((3-(1-((1-(Cyanomethyl)cyclopropyl)methyl)-1*H*-1,2,3-triazol-4-yl)phenyl)sulfonyl)butyramide (4{33,6,4}) ^{1}H NMR S36

N-((3-(1-((1-(Cyanomethyl)cyclopropyl)methyl)-1*H*-1,2,3-triazol-4-yl)phenyl)sulfonyl)butyramide (4{33,6,4}) ^{13}C NMR S37

N-((4-(1-(4-(1*H*-Pyrazol-1-yl)benzyl)-1*H*-1,2,3-triazol-4-yl)phenyl)sulfonyl)nicotinamide (4{18,7,17}) ^{1}H NMR S38

N-((4-(1-(4-(1*H*-Pyrazol-1-yl)benzyl)-1*H*-1,2,3-triazol-4-yl)phenyl)sulfonyl)nicotinamide (4{18,7,17}) ^{13}C NMR S39

1-(3,4-Dimethoxybenzyl)-*N*-(4-(*N*-(2-ethoxyacetyl)sulfamoyl)phenyl)-1*H*-1,2,3-triazole-4-carboxamide (4{1,8,1}) ^{1}H NMR S40

1-(3,4-Dimethoxybenzyl)-*N*-(4-(*N*-(2-ethoxyacetyl)sulfamoyl)phenyl)-1*H*-1,2,3-triazole-4-carboxamide (4{1,8,1}) ^{13}C NMR S41

N-((3-(1-(4-(1*H*-Pyrazol-1-yl)benzyl)-1*H*-1,2,3-triazol-4-yl)phenyl)sulfonyl)nicotinamide (4{18,6,17}) ^{1}H NMR S42

N-((3-(1-(4-(1*H*-Pyrazol-1-yl)benzyl)-1*H*-1,2,3-triazol-4-yl)phenyl)sulfonyl)nicotinamide (4{18,6,17}) ^{13}C NMR S43

N-((4-(4-(Benzo[*d*]thiazol-2-yl)-1*H*-1,2,3-triazol-1-yl)phenyl)sulfonyl)-2-(1-methylcyclopropyl)acetamide (7{3,37,71}) ^{1}H NMR S44

N-((4-(4-(Benzo[*d*]thiazol-2-yl)-1*H*-1,2,3-triazol-1-yl)phenyl)sulfonyl)-2-(1-methylcyclopropyl)acetamide (7{3,37,71}) ^{13}C NMR S45

N-((5-(4-(1-(3,4-Dimethoxybenzyl)-1*H*-1,2,3-triazol-4-yl)butanamido)-1,3,4-thiadiazol-2-yl)sulfonyl)cyclopropanecarboxamide (4{1,5,2}) ^{1}H NMR S46

N-((5-(4-(1-(3,4-Dimethoxybenzyl)-1*H*-1,2,3-triazol-4-yl)butanamido)-1,3,4-thiadiazol-2-yl)sulfonyl)cyclopropanecarboxamide (4{1,5,2}) ^{13}C NMR S47

N-((4-((4-((3-Bromo-6-oxopyridazin-1(6*H*)-yl)methyl)-1*H*-1,2,3-triazol-1-yl)methyl)phenyl)sulfonyl)butyramide (7{1,46,4}) ^{1}H NMR S48

N-((4-((4-((3-Bromo-6-oxopyridazin-1(6*H*)-yl)methyl)-1*H*-1,2,3-triazol-1-yl)methyl)phenyl)sulfonyl)butyramide (7{1,46,4}) ^{13}C NMR S49

6-Methyl-*N*-((5-(4-(1-(2-(methylsulfonyl)ethyl)-1*H*-1,2,3-triazol-4-yl)butanamido)-1,3,4-thiadiazol-2-yl)sulfonyl)pyrazine-2-carboxamide (4{45,5,30}) ^{1}H NMR S50

6-Methyl-*N*-((5-(4-(1-(2-(methylsulfonyl)ethyl)-1*H*-1,2,3-triazol-4-yl)butanamido)-1,3,4-thiadiazol-2-yl)sulfonyl)pyrazine-2-carboxamide (4{45,5,30}) ^{13}C NMR S51

1-Methyl-*N*-((3-(1-((1-methyl-1*H*-pyrazol-4-yl)methyl)-1*H*-1,2,3-triazol-4-yl)phenyl)sulfonyl)-1*H*-pyrazole-4-carboxamide (4{34,6,47}) ^{1}H NMR S52

1-Methyl-*N*-((3-(1-((1-methyl-1*H*-pyrazol-4-yl)methyl)-1*H*-1,2,3-triazol-4-yl)phenyl)sulfonyl)-1*H*-pyrazole-4-carboxamide (4{34,6,47}) ^{13}C NMR S53

N-((4-((4-(5-Bromopyridin-3-yl)-1*H*-1,2,3-triazol-1-yl)methyl)phenyl)sulfonyl)butyramide (7{1,35,4}) ^{1}H NMR S54

N-((4-((4-(5-Bromopyridin-3-yl)-1*H*-1,2,3-triazol-1-yl)methyl)phenyl)sulfonyl)butyramide (7{1,35,4}) ^{13}C NMR S55

N-((1-(4-(*N*-Butyrylsulfamoyl)benzyl)-1*H*-1,2,3-triazol-4-yl)methyl)-3-cyanobenzamide (7{1,47,4}) ^{1}H NMR S56

N-((1-(4-(*N*-Butyrylsulfamoyl)benzyl)-1*H*-1,2,3-triazol-4-yl)methyl)-3-cyanobenzamide (7{1,47,4}) ^{13}C NMR S57

N-((3-(4-([1,1'-Biphenyl]-2-yl)-1*H*-1,2,3-triazol-1-yl)phenyl)sulfonyl)isonicotinamide (7{2,18,50}) ^{1}H NMR S58

N-((3-(4-([1,1'-Biphenyl]-2-yl)-1*H*-1,2,3-triazol-1-yl)phenyl)sulfonyl)isonicotinamide (7{2,18,50}) ^{13}C NMR S59

N-((3-(1-(3-(*tert*-Butyl)phenyl)-1*H*-1,2,3-triazol-4-yl)phenyl)sulfonyl)furan-3-carboxamide (4{10,6,48}) ^{1}H NMR S60

N-((3-(1-(3-(*tert*-Butyl)phenyl)-1*H*-1,2,3-triazol-4-yl)phenyl)sulfonyl)furan-3-carboxamide (4{10,6,48}) ^{13}C NMR S61

N-((5-(4-(1-(3-Acetylbenzyl)-1*H*-1,2,3-triazol-4-yl)butanamido)-1,3,4-thiadiazol-2-yl)sulfonyl)thiazole-4-carboxamide (4{11,5,80}) ^{1}H NMR S62

N-((5-(4-(1-(3-Acetylbenzyl)-1*H*-1,2,3-triazol-4-yl)butanamido)-1,3,4-thiadiazol-2-yl)sulfonyl)thiazole-4-carboxamide (4{11,5,80}) ^{13}C NMR S63

3-Methyl-*N*-((4-(1-((3-methylisoxazol-5-yl)methyl)-1*H*-1,2,3-triazol-4-yl)phenyl)sulfonyl)butanamide (4{20,7,16}) ^{1}H NMR S64

3-Methyl-*N*-((4-(1-((3-methylisoxazol-5-yl)methyl)-1*H*-1,2,3-triazol-4-yl)phenyl)sulfonyl)butanamide (4{20,7,16}) ^{13}C NMR S65

N-((4-(1-(4-(1*H*-Pyrazol-1-yl)benzyl)-1*H*-1,2,3-triazol-4-yl)phenyl)sulfonyl)thiophene-3-carboxamide (4{18,7,32}) ^{1}H NMR S66

N-((4-(1-(4-(1*H*-Pyrazol-1-yl)benzyl)-1*H*-1,2,3-triazol-4-yl)phenyl)sulfonyl)thiophene-3-carboxamide (4{18,7,32}) ^{13}C NMR S67

N-((4-(1-(1-Amino-1-oxo-3-phenylpropan-2-yl)-1*H*-1,2,3-triazol-4-yl)phenyl)sulfonyl)-2-methyloxazole-4-carboxamide (4{21,7,33}) ^{1}H NMR S68

N-((4-(1-(1-Amino-1-oxo-3-phenylpropan-2-yl)-1*H*-1,2,3-triazol-4-yl)phenyl)sulfonyl)-2-methyloxazole-4-carboxamide (4{21,7,33}) ^{13}C NMR S69

N-((3-(4-(Benzo[*d*]thiazol-2-yl)-1*H*-1,2,3-triazol-1-yl)phenyl)sulfonyl)isothiazole-3-carboxamide (7{2,37,72}) ^{1}H NMR S70

N-((3-(4-(Benzo[*d*]thiazol-2-yl)-1*H*-1,2,3-triazol-1-yl)phenyl)sulfonyl)isothiazole-3-carboxamide (7{2,37,72}) ^{13}C NMR S71

N-(4-(*N*-(2-Ethoxyacetyl)sulfamoyl)phenyl)-1-(naphthalen-1-ylmethyl)-1*H*-1,2,3-triazole-4-carboxamide (4{2,8,1}) ^{1}H NMR S72

N-(4-(*N*-(2-Ethoxyacetyl)sulfamoyl)phenyl)-1-(naphthalen-1-ylmethyl)-1*H*-1,2,3-triazole-4-carboxamide (4{2,8,1}) ^{13}C NMR S73

3,3-Dimethyl-*N*-((5-(4-(1-(2-(methylsulfonyl)ethyl)-1*H*-1,2,3-triazol-4-yl)butanamido)-1,3,4-thiadiazol-2-yl)sulfonyl)butanamide (4{45,5,81}) ^{1}H NMR S74

3,3-Dimethyl-*N*-((5-(4-(1-(2-(methylsulfonyl)ethyl)-1*H*-1,2,3-triazol-4-yl)butanamido)-1,3,4-thiadiazol-2-yl)sulfonyl)butanamide (4{45,5,81}) ^{13}C NMR S75

N-((5-(4-(1-(2-(Methylsulfonyl)ethyl)-1*H*-1,2,3-triazol-4-yl)butanamido)-1,3,4-thiadiazol-2-yl)sulfonyl)cyclopropanecarboxamide (4{45,5,2}) ^{1}H NMR S76

N-((5-(4-(1-(2-(Methylsulfonyl)ethyl)-1*H*-1,2,3-triazol-4-yl)butanamido)-1,3,4-thiadiazol-2-yl)sulfonyl)cyclopropanecarboxamide (4{45,5,2}) ^{13}C NMR S77

3-Cyclobutyl-*N*-((5-(1-((5-cyclopropyl-1,3,4-oxadiazol-2-yl)methyl)-1*H*-1,2,3-triazol-4-yl)pentyl)sulfonyl)propanamide (4{51,4,75}) ^{1}H NMR S78

3-Cyclobutyl-*N*-((5-(1-((5-cyclopropyl-1,3,4-oxadiazol-2-yl)methyl)-1*H*-1,2,3-triazol-4-yl)pentyl)sulfonyl)propanamide (4{51,4,75}) ^{13}C NMR S79

N-((5-(4-(1-(2-(Methylsulfonyl)ethyl)-1*H*-1,2,3-triazol-4-yl)butanamido)-1,3,4-thiadiazol-2-yl)sulfonyl)cyclobutanecarboxamide (4{45,5,78}) ^{1}H NMR S80

N-((5-(4-(1-(2-(Methylsulfonyl)ethyl)-1*H*-1,2,3-triazol-4-yl)butanamido)-1,3,4-thiadiazol-2-yl)sulfonyl)cyclobutanecarboxamide (4{45,5,78}) ^{13}C NMR S81

N-((3-(1-(4-(*tert*-Butyl)phenyl)-1*H*-1,2,3-triazol-4-yl)phenyl)sulfonyl)isonicotinamide (4{19,6,50}) ^{1}H NMR S82

N-((3-(1-(4-(*tert*-Butyl)phenyl)-1*H*-1,2,3-triazol-4-yl)phenyl)sulfonyl)isonicotinamide (4{19,6,50}) ^{13}C NMR S83

(*S*)-*N*-((3-(4-(2-Methyl-2*H*-indazol-6-yl)-1*H*-1,2,3-triazol-1-yl)phenyl)sulfonyl)-1,4-dioxane-2-carboxamide (7{2,20,59}) ^{1}H NMR S84

(*S*)-*N*-((3-(4-(2-Methyl-2*H*-indazol-6-yl)-1*H*-1,2,3-triazol-1-yl)phenyl)sulfonyl)-1,4-dioxane-2-carboxamide (7{2,20,59}) ^{13}C NMR S85

N-((5-(4-(1-(4-Acetylbenzyl)-1*H*-1,2,3-triazol-4-yl)butanamido)-1,3,4-thiadiazol-2-yl)sulfonyl)isonicotinamide (4{43,5,50}) ^{1}H NMR S86

N-((5-(4-(1-(4-Acetylbenzyl)-1*H*-1,2,3-triazol-4-yl)butanamido)-1,3,4-thiadiazol-2-yl)sulfonyl)isonicotinamide (4{43,5,50}) ^{13}C NMR S87

N-((4-(1-((2-Cyclopropylpyrimidin-5-yl)methyl)-1*H*-1,2,3-triazol-4-yl)butyl)sulfonyl)bicyclo[3.1.0]hexane-3-carboxamide (4{64,3,61}) ^{1}H NMR S88

N-((4-(1-((2-Cyclopropylpyrimidin-5-yl)methyl)-1*H*-1,2,3-triazol-4-yl)butyl)sulfonyl)bicyclo[3.1.0]hexane-3-carboxamide (4{64,3,61}) ^{13}C NMR S89

N-((4-(1-((2,3-Dihydrobenzo[*b*][1,4]dioxin-2-yl)methyl)-1*H*-1,2,3-triazol-4-yl)phenyl)sulfonyl)-3-methylcyclobutane-1-carboxamide (4{17,7,31}) ^{1}H NMR S90

N-((4-(1-((2,3-Dihydrobenzo[*b*][1,4]dioxin-2-yl)methyl)-1*H*-1,2,3-triazol-4-yl)phenyl)sulfonyl)-3-methylcyclobutane-1-carboxamide (4{17,7,31}) ^{13}C NMR S91

References S92

In vitro screening experimental details

Identification of carbonic anhydrase IX and II binders by thermal shift assay (TSA) (primary screening)

Screening of the selection of compounds against the recombinant human carbonic anhydrase IX protein (hCA-IX) and human carbonic anhydrase II protein (hCA-II) was done by protein thermal shift assay in singletons. Sypro Orange (10 µM) was used as a reporter dye, and acetazolamide (20 µM or 40 µM) – as a reference binder. Negative and positive controls were read at sixteen repeats. The screening compounds were tested at two concentrations – 20 µM or 40 µM. The total reaction volume was 10 µL, with hCA-IX or hCA-II concentrations of 0.2 mg/mL. Mutated version of human hCA-IX catalytic domain and non-tagged hCA-II protein with the native amino acid sequence were recombinantly produced in house in an *E. coli* expression system according to the previously published protocols.[1,2]

Screening compounds, reporter dye, and reference compound were formatted into well plates (PCR microplate, 384-well, white, Axygen, Cat. PCR-384-LC480-W) using Echo 550 acoustic liquid handler (Labcyte Inc). TSypro Orange was mixed with the target protein and manually formatted onto the assay-ready plates prepared with Labcyte ECHO liquid handler. he working solutions of hCA-IX or hCA-II was freshly prepared in the assay buffer (10 mM HEPES, 200 mM NaCl; pH 7.2), and aliquoted into the assay-ready compound plates using an automated multichannel dispenser Multidrop Combi (Thermo Fischer Scientific) immediately prior to the analysis. The plate was sealed with Excel Scientific ThermalSeal RT™ TS-RT2RR-100 optical sealing film, mixed for 1 min at 700 rpm, centrifuged for 20 sec, and placed into qTower 84G rtPCR cycler (Analytic Jena).

Melting parameters (for qTower^{3}84): pre-incubation: 25 °C, 5 min; temperature range: 30–85 °C; rate: 0.1 °C/s; equilibration time: 3 s; filter: tetramethylrhodamine (TAMRA), gain: 5.

The assay was performed at two concentrations because the initial 20 µM run yielded low-quality readouts in some cases; therefore, data from both runs are reported. Acetazolamide was included in each plate as a reference control in 16 replicates; the values were calculated from these replicates. For the tested compounds, values were calculated as plate-averaged means for each protein and each concentration. The primary hits were identified using a dedicated in-house developed software for generating melt curves and calculating protein melt temperatures by the derivative method and applying the formula: $\Delta T_m \geq \mathrm{MED}(\Delta T_m) \pm \mathrm{RSD}(\Delta T_m)$, where ΔT_m – thermal shift for a particular library member, $\mathrm{MED}(\Delta T_m)$ and $\mathrm{RSD}(\Delta T_m)$ – median value and robust standard deviation, respectively, for the compounds screened in the same plate (excluding controls) (Figures S1 and S2). A minimum thermal shift threshold was 0.5 °C to ensure meaningful interactions.

The details of measurements for 4 best compounds of this study and acetazolamide are given in Table S1. The confirmation of the selected primary hits was performed under the same experimental conditions in quadruplicates.

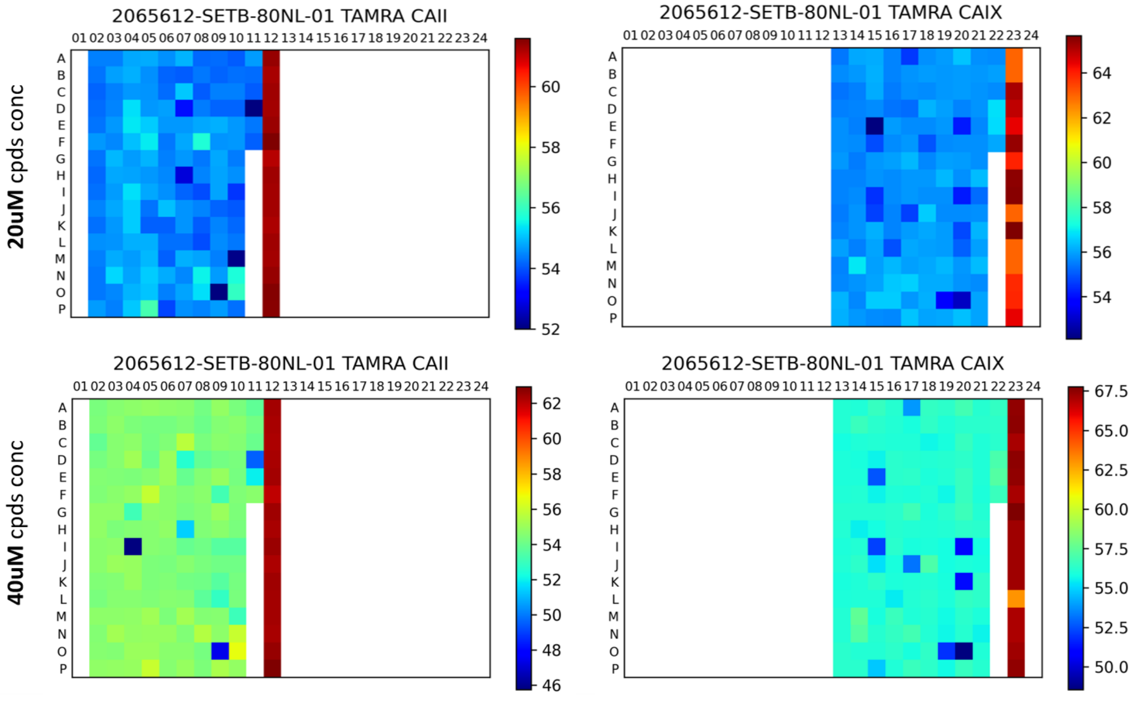

Figure S1. TSA results presented as heat maps

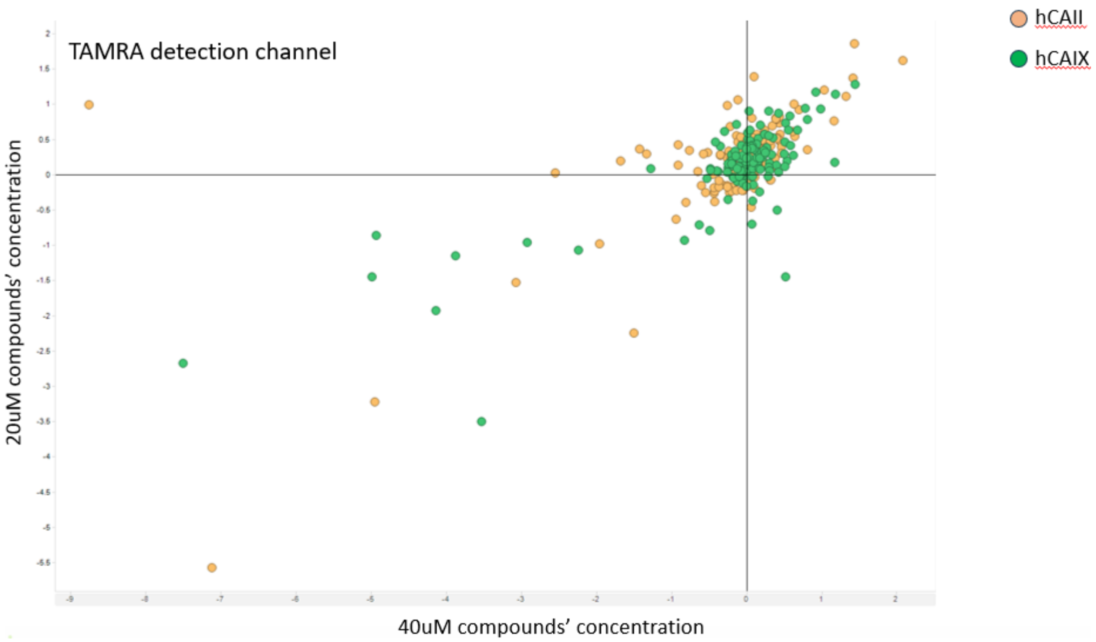

Figure S2. Correlation of thermal shift values observed at 20 μM and 40 μM

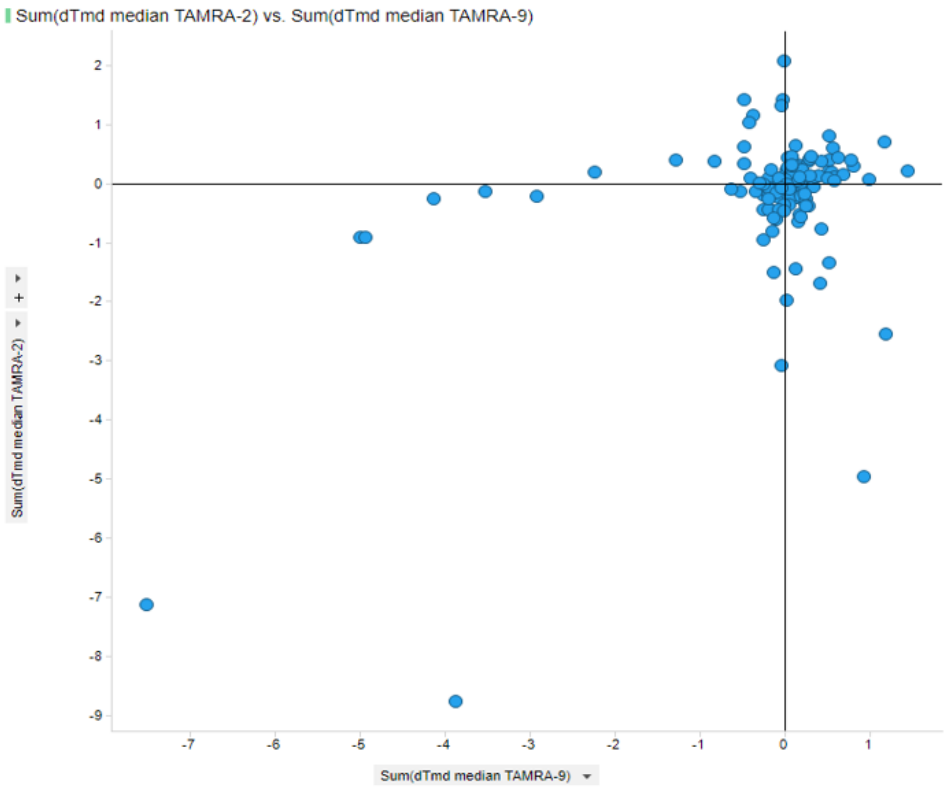

Figure S3. Correlation of thermal shift values for hCA-II and hCA-IX (40 μM compound concentration)

Table S1. Measurement details for differential scanning fluorimetry (DSF) screening of compounds **4**{*17*,*7*,*27*}, **7**{*2*,*21*,*60*}, **4**{*16*,*7*,*28*}, and **4**{*16*,*7*,*19*} against hCA-II and hCA-IX at 20 and 40 μM.

Test[a]	Compound	Protein	T_m, °C	ΔT_m, °C	DMSO T_m, median, °C	DMSO T_m RSD, °C	Acetazol-amide T_m, median, °C	Acetazol-amide T_m RSD, °C	Acetazol-amide ΔT_m, median, °C	MED(T_m), °C	RSD(T_m), °C	MED(ΔT_m), °C
Screening at 20 μM	**4**{*17,7,27*}	hCA-II	54.82	0.50	54.32	0.14	61.3	0.09	6.98	54.60	0.37	0.28
	7{*2,21,60*}	hCA-II	55.24	0.92								
	**4**{*16,7,28*}	hCA-II	54.35	0.03								
	**4**{*16,7,19*}	hCA-II	51.10	–3.22								
Screening at 40 μM	**4**{*17,7,27*}	hCA-II	54.73	0.21	54.52	0.25	62.36	0.11	7.84	54.53	0.36	0.01
	7{*2,21,60*}	hCA-II	55.22	0.70								
	**4**{*16,7,28*}	hCA-II	51.97	–2.55								
	**4**{*16,7,19*}	hCA-II	49.58	–4.95								
Screening at 20 μM	**4**{*17,7,27*}	hCA-IX	56.91	1.28	55.63	0.15	64.18	1.74	8.55	55.83	0.25	0.20
	7{*2,21,60*}	hCA-IX	55.81	0.18								
	**4**{*16,7,28*}	hCA-IX	56.77	1.14								
	**4**{*16,7,19*}	hCA-IX	56.80	1.17								
Screening at 40 μM	**4**{*17,7,27*}	hCA-IX	57.51	1.44	56.06	0.14	67.22	0.31	11.16	56.09	0.29	0.03
	7{*2,21,60*}	hCA-IX	57.23	1.17								
	**4**{*16,7,28*}	hCA-IX	57.24	1.18								
	**4**{*16,7,19*}	hCA-IX	56.98	0.92								

hCA esterase activity inhibition screening assay (with IC_{50} determination)

The protocol was adapted from the work by Wu and co-authors.[3] Briefly, the catalytic activity of human hCA-IX (catalytic domain) and hCA-II recombinant proteins was monitored by following the hydrolysis of *p*-nitrophenyl acetate (4-NPA, purchased from Sigma-Aldrich (Cat. 46021), 5 mM stock solution prepared in EtOH).

The selected compounds, as well as acetazolamide (purchased from Sigma-Aldrich (Cat. A6011), 10–20 mM stock prepared in DMSO) as a reference compound, were titrated from 200 μM to 0.01 μM at 3× serial dilutions (10-point curves) and formatted into 384-well plates (Thermo Scientific Nunc, 264723) using Echo 550 acoustic liquid handler (Labcyte Inc). The working solutions of hCA-II and hCA-IX were freshly prepared in the assay buffer (25 mM Tris-HCl, 200 mM NaCl, pH 7.5), and aliquoted into the assay-ready plates with tested compounds immediately prior to the analysis using an automated multichannel dispenser Multidrop Combi (Thermo Fischer Scientific).

The products of the hydrolysis reaction are acetate and nitrophenolate, which ionizes to give a bright yellow anion that is detected by measuring its absorbance at 405 nm with a visible spectrophotometer M5 (Molecular Devices) using 384-well plates (non-treated; Polystyrene; non-sterile; Nunc#264723).

The compounds were formatted into 384-well plates in the following manner. The first two columns (n = 32 wells) contained DMSO only and were used as a control to determine minimum, or 0% inhibition of the reaction. The last two columns (n = 32 wells) contained acetazolamide at ×4 concentration (800 μM) and were used as a control to determine maximum, or 100% inhibition of the reaction. In between, the tested compounds were placed at the defined dilution step of the 10-points concentration curve, each point of which was a quadruplicate (n = 4 wells). Thus, the calculated maximum and minimum inhibition, each calculated as mean of 32 individual data points with standard deviation of <0.05, were used for further assessment of each point of inhibition curves; they were not shown in the plots.

Concentration of enzymes were fixed at 917 nM for hCA-IX and 200 nM for hCA-II. Compound titrations were performed with Labcyte Echo liquid handler to prepare assay-ready plates. Reaction was run in 25 μL of assay buffer per well (25 mM Tris-HCl,150 mM NaCl, pH 8.0).

Enzymatic reactions were initiated by pipetting 5 mM stock solution of 4-nitrophenyl acetate (4-NPA) in ethanol to each well (final concentration in the reaction mixture – 0.5 mM for hCA-II and 0.9 mM for hCA-IX). The final reaction volume was 27.5 μL. The enzyme activity was evaluated by monitoring the production of colored product, 4-nitrophenol (4-NP), at 405 nm every 3 min for up to 2 h at 25°C using spectrophotometric microplate reader SpectraMax M5 (Molecular Devices). The assay was performed twice for each enzyme.

Each data point of the titration curve was calculated as average of aggregate data value of four measurements per each concentration (technical replicate for each concentration n = 4). The IC_{50} values were calculated using GraphPad Prizm software.

Figures S4 and S5 show the obtained dose-response curves. The raw data for 4 best compounds is given in Table S2. ΔT_m vs IC_{50} correlation for the activity against hCA-IX and hCA-II is given in Table S3.

The known carbonic anhydrase family inhibitor, acetazolamide, was used for two purposes: firstly, to obtain the data for maximum inhibition of the enzyme (as described above) and apply this value for further calculation of the percent inhibition for the tested compounds (this data was not shown in the plots). Secondly, it was also titrated as one of the tested compounds to ensure that the assay is performing properly withing the given range of the known inhibitor concentrations. That is why the IC_{50} curve of acetazolamide was shown along with the IC_{50} curves of the tested compounds in Figures S4 and S5.

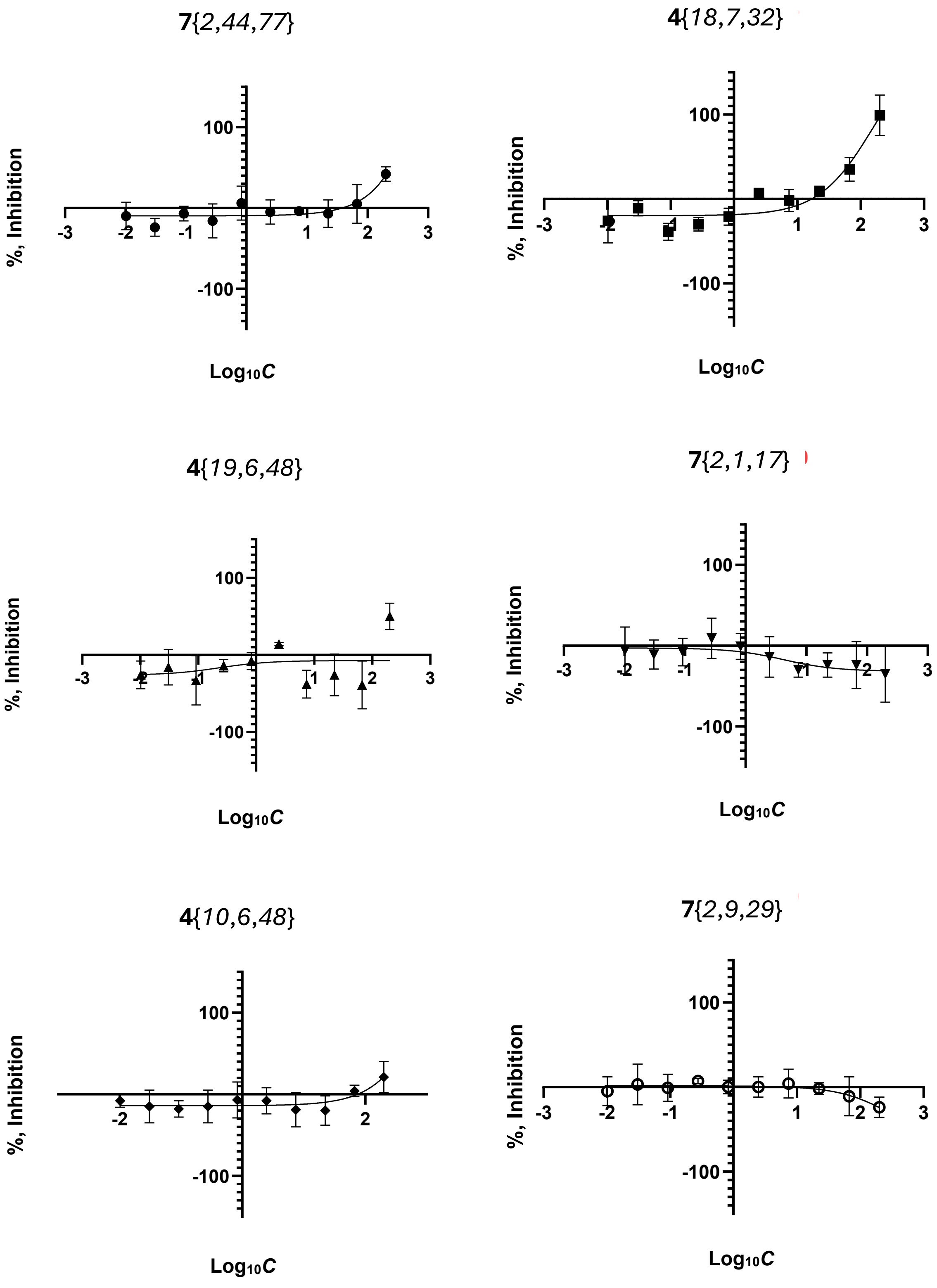

Figure S4. Dose-response curves for IC_{50} determination (200 nM of hCA-II, 0.5 mM of 4-NPA, $n = 4$)

7{*3,19,57*}

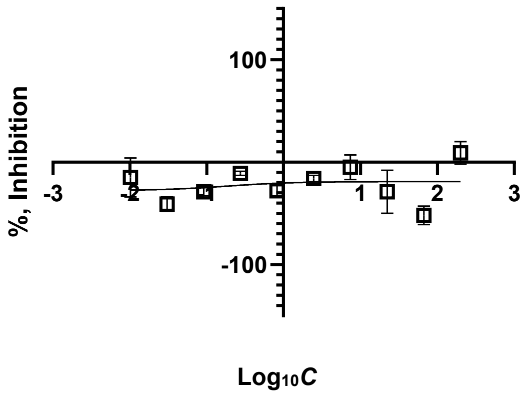

7{*2,21,60*}

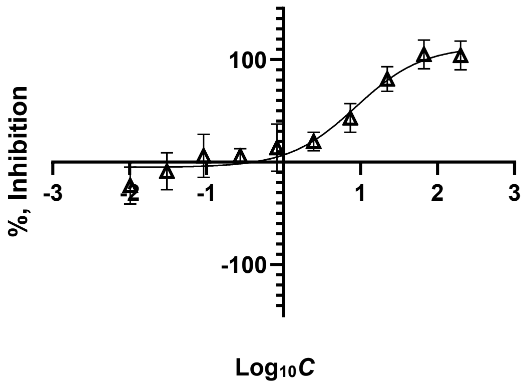

4{*17,7,27*}

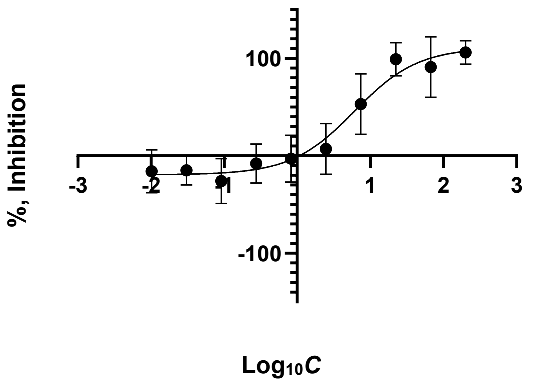

4{*16,7,28*}

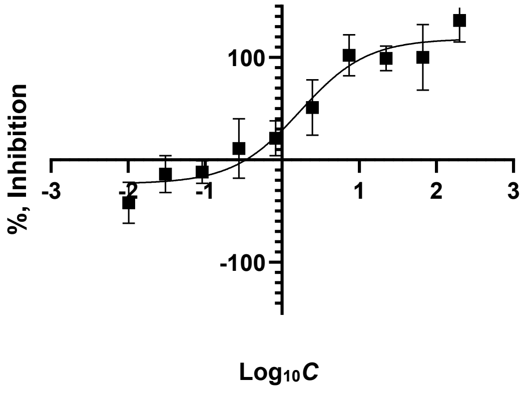

4{*16,7,19*}

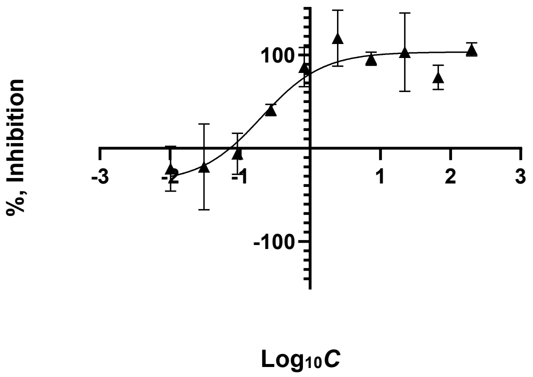

7{*2,4,21*}

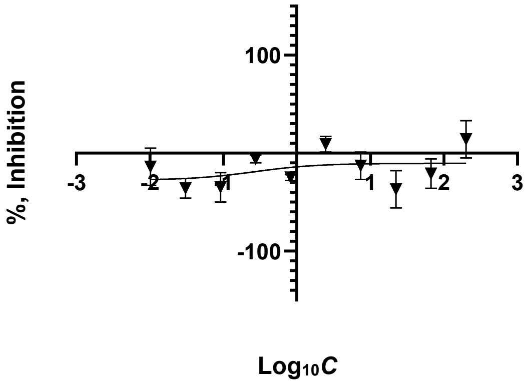

Figure S4 *(continued)*.

7{*1,16,5*}

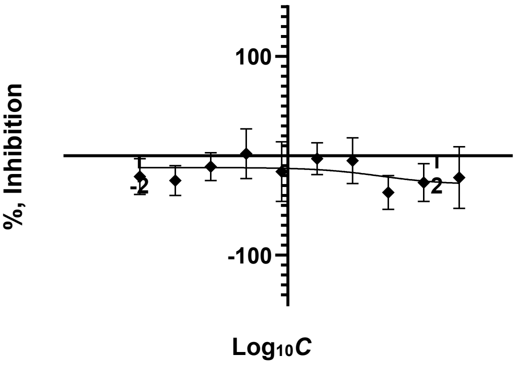

7{*3,43,23*}

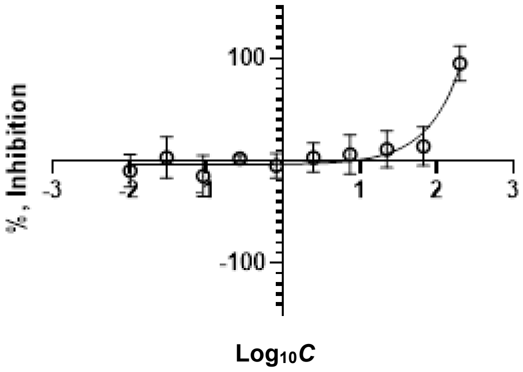

4{*2,8,3*}

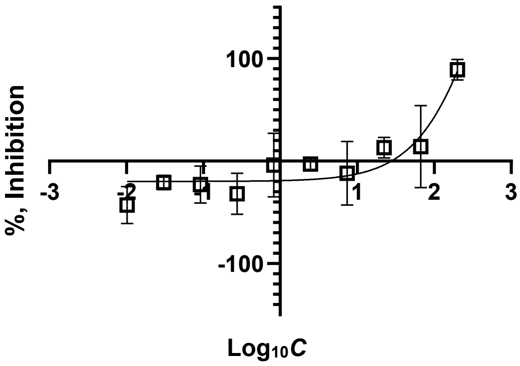

Acetazolamide

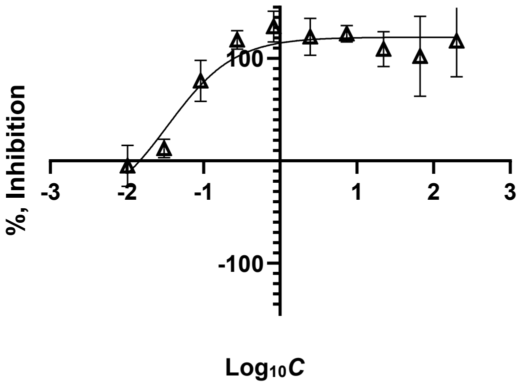

Figure S4 *(continued)*.

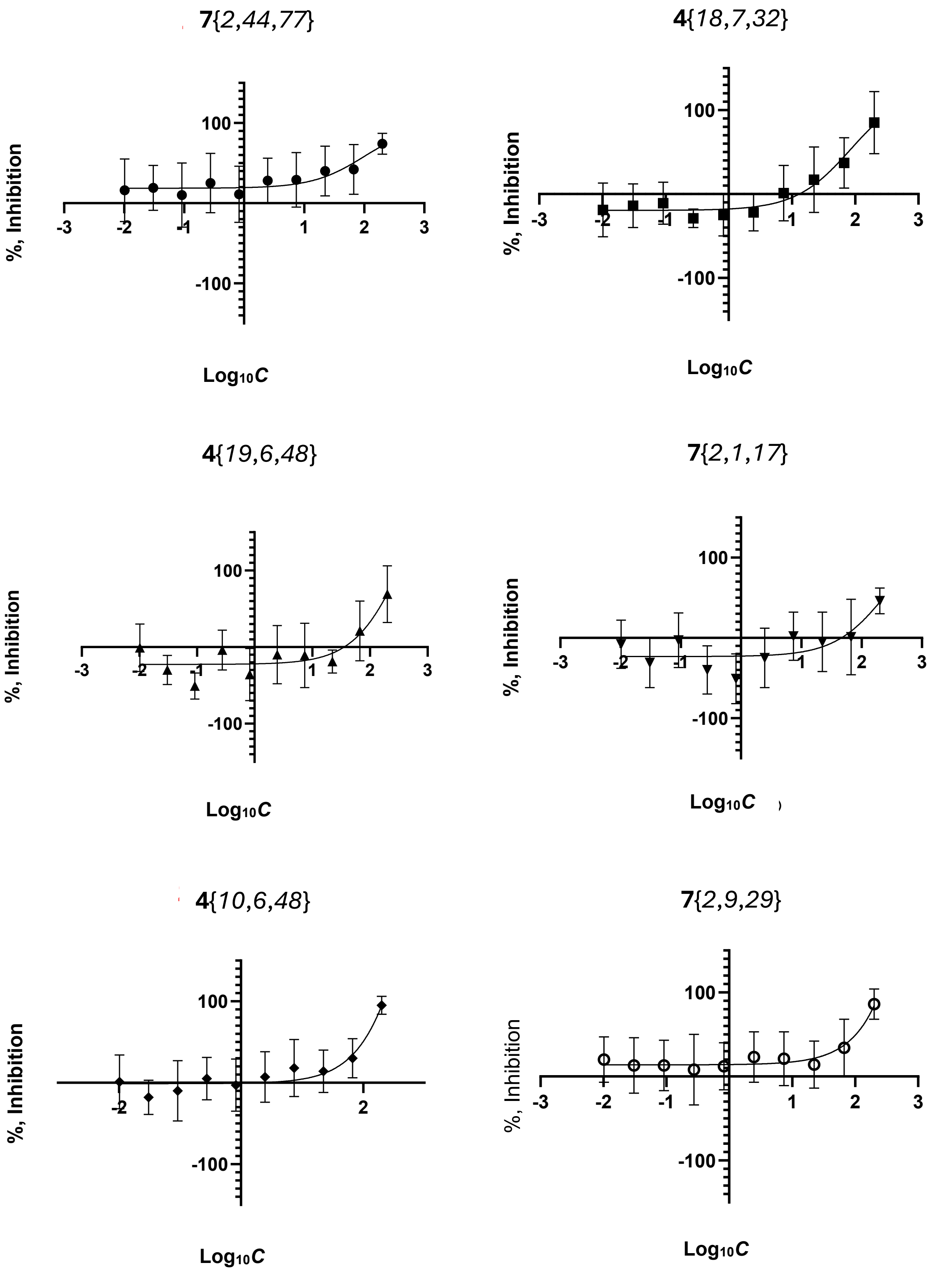

Figure S5. Dose-response curves for IC_{50} determination (917 nM of hCA-IX, 0.5 mM of 4-NPA, n = 4)

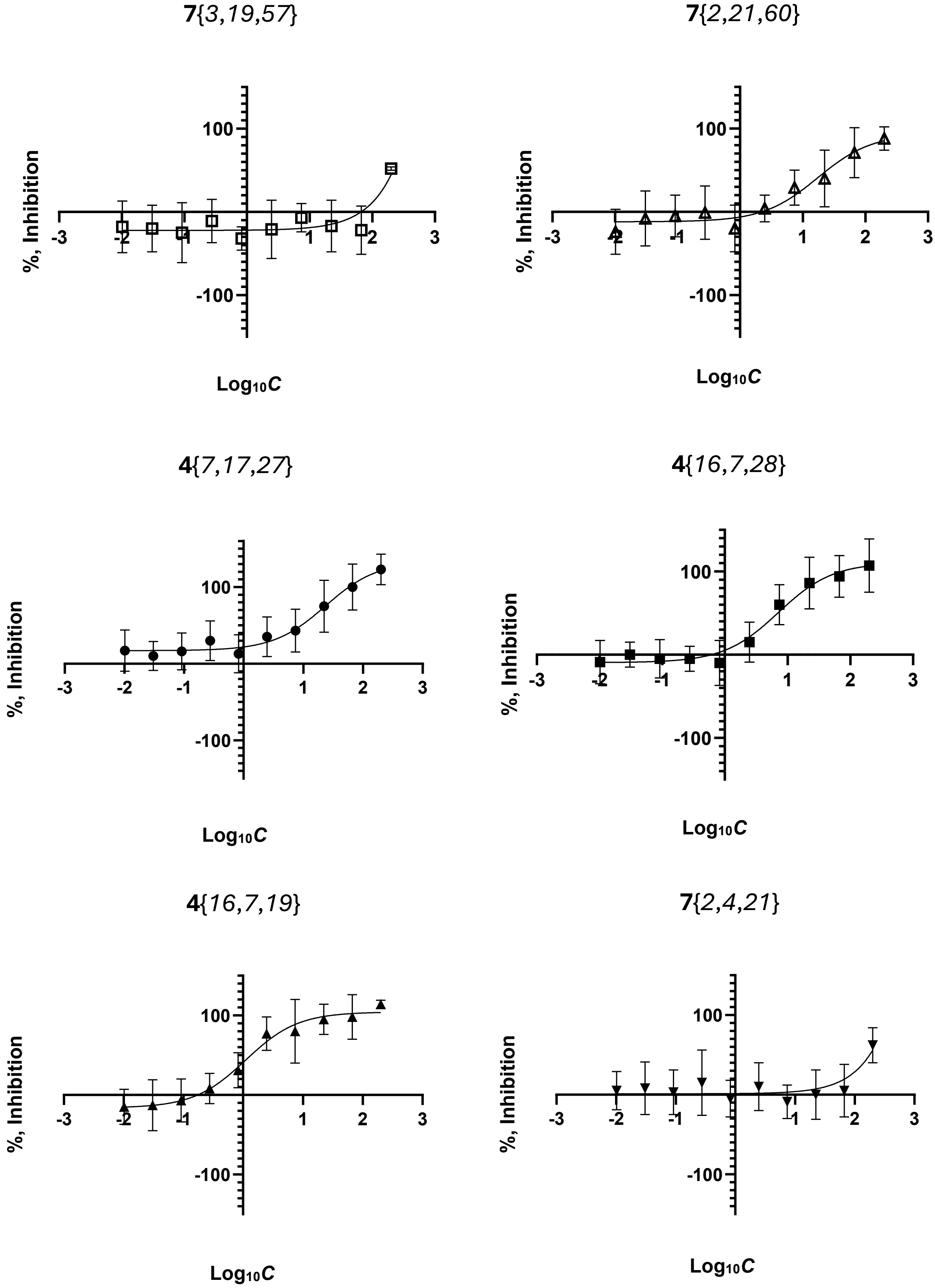

Figure S5 (*continued*).

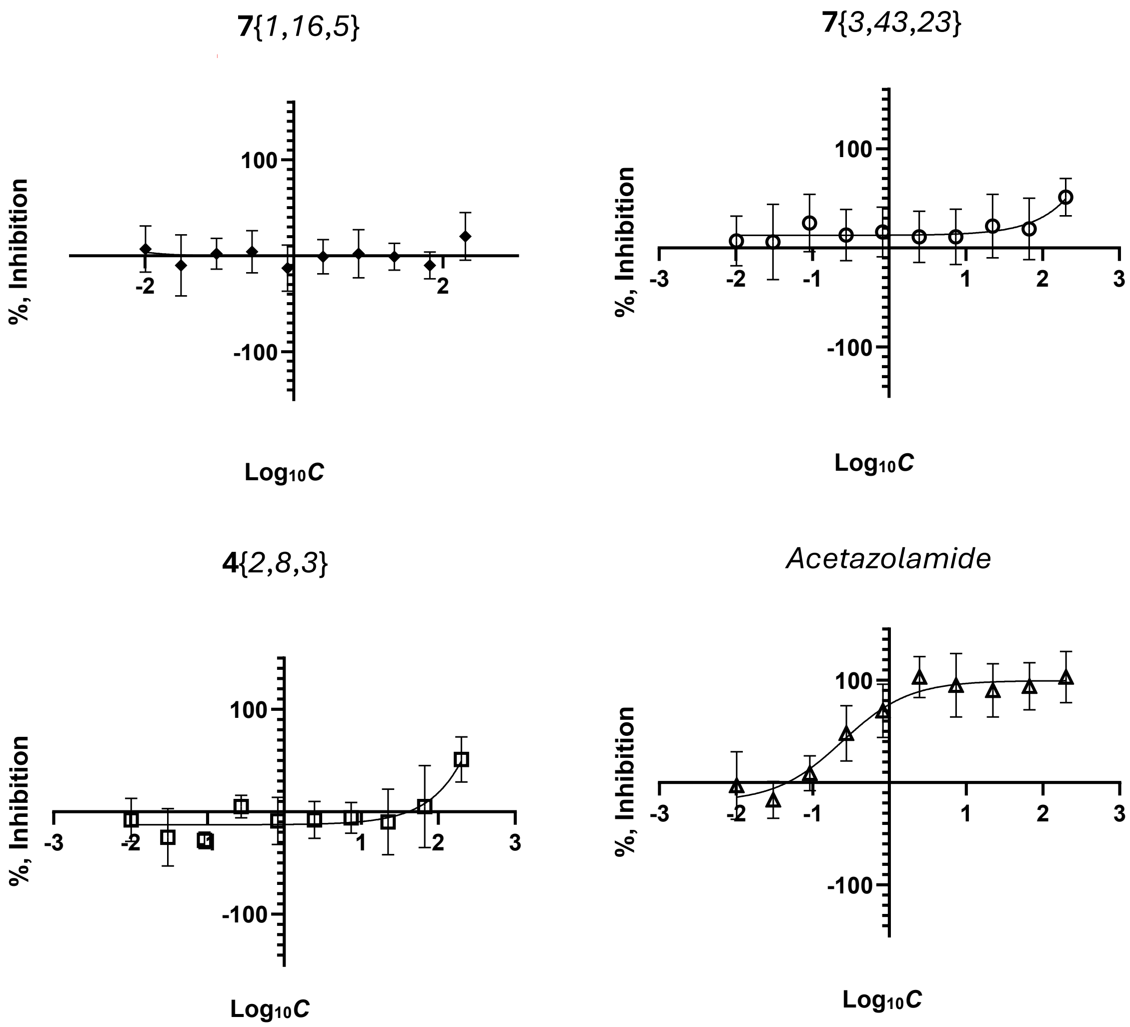

Figure S5 (*continued*).

Table S2. Raw data used for the IC_{50} determination for compounds **4**{*17,7,27*}, **7**{*2,21,60*}, **4**{*16,7,28*}, **4**{*16,7,19*}, and acetazolamide (n = 4)

Compound	Protein	Absorbance (A, in quadruplicates) at the compound concentration of																			
		200 µM		67 µM		33 µM		17 µM		8 µM		4 µM		2 µM		1 µM		0.52 µM		0.26 µM	
**7**{*2,21,60*}	hCA-II	0.3374	0.3658	0.366	0.4021	0.3166	0.3728	0.3069	0.3204	0.3133	0.3355	0.2798	0.3629	0.2957	0.367	0.3366	0.3843	0.4235	0.4493	0.4312	0.4824
		0.2786	0.2534	0.2866	0.2717	0.2762	0.3171	0.2888	0.2913	0.2915	0.2391	0.3016	0.2658	0.2975	0.3169	0.3736	0.3201	0.4433	0.4208	0.4767	0.429
**4**{*17,7,27*}		0.2668	0.3212	0.2884	0.3338	0.3523	0.3741	0.3751	0.4896	0.4816	0.5017	0.4731	0.4971	0.4287	0.5019	0.476	0.5081	0.4611	0.504	0.4513	0.4718
		0.2987	0.2652	0.3151	0.2556	0.3474	0.3239	0.3914	0.3336	0.4321	0.3807	0.4507	0.4303	0.476	0.4322	0.513	0.4288	0.4972	0.4575	0.5101	0.4282
**4**{*16,7,28*}		0.2709	0.3003	0.335	0.3389	0.3383	0.3353	0.3564	0.364	0.3825	0.4015	0.4008	0.4394	0.4295	0.4527	0.4449	0.5243	0.4452	0.5561	0.456	0.4885
		0.2936	0.2496	0.2914	0.2741	0.3212	0.2761	0.3286	0.3121	0.3788	0.3273	0.4429	0.36	0.416	0.4097	0.4871	0.4399	0.4715	0.4404	0.4887	0.4409
**4**{*16,7,19*}		0.321	0.2891	0.3152	0.3246	0.3134	0.3684	0.3815	0.307	0.3115	0.3943	0.3106	0.4365	0.387	0.4343	0.3928	0.523	0.4417	0.5152	0.4764	0.5144
		0.3802	0.2817	0.3458	0.2881	0.3572	0.2984	0.3386	0.3165	0.3284	0.343	0.3916	0.3138	0.4145	0.3664	0.5279	0.4487	0.5481	0.4614	0.5783	0.4604
Acetazolamide		0.2687	0.3328	0.3492	0.3609	0.2823	0.3424	0.3388	0.3096	0.2823	0.3203	0.2681	0.3106	0.2914	0.3622	0.3384	0.3873	0.4039	0.4401	0.435	0.4842
		0.2866	0.2457	0.2839	0.2443	0.2919	0.3036	0.2978	0.2504	0.2793	0.2496	0.3093	0.2996	0.3316	0.3329	0.3773	0.3399	0.4306	0.3907	0.474	0.4248
**7**{*2,21,60*}	hCA-IX	0.7784	0.8353	0.8091	0.9353	0.8697	1.0116	0.9068	0.9964	0.9357	1.0434	1.0098	1.1056	0.9504	1.0842	0.9623	1.0683	0.9607	1.1103	0.9956	1.1272
		0.9896	0.8253	0.9227	0.7688	0.9954	0.8451	1.0633	0.9125	1.0398	0.9755	1.1255	0.9591	1.1467	0.9488	1.1508	0.9845	1.1008	0.9647	1.1184	1.0127
**4**{*17,7,27*}		0.6666	0.7646	0.7193	0.8865	0.7437	0.9593	0.8691	1.0075	0.8947	1.0229	0.9621	1.084	0.8986	1.0472	0.9469	1.0686	0.9738	1.0468	0.9041	1.0567
		0.8098	0.6941	0.8536	0.7048	0.9121	0.7864	0.9891	0.8232	1.0018	0.8672	1.0438	0.8856	1.0241	0.8967	1.046	0.9245	1.0877	0.9498	1.0653	0.9289
**4**{*16,7,28*}		0.6613	0.8194	0.725	0.9268	0.733	0.8851	0.8291	0.9565	0.9391	1.058	0.9874	1.1308	0.9473	1.0942	0.9957	1.1002	0.9774	1.0822	1.0221	1.0923
		0.8978	0.7185	0.8489	0.7574	0.9194	0.7609	0.9463	0.8267	1.1085	0.9389	1.122	0.9987	1.0742	1.0199	1.1364	0.9858	1.0668	1.0066	1.1514	0.9475
**4**{*16,7,19*}		0.7441	0.7625	0.6974	0.8389	0.7542	0.8469	0.7487	0.9495	0.7755	0.8786	0.8989	1.0108	0.965	1.1183	0.9762	1.1117	0.9655	1.21	0.9897	1.1801
		0.8485	0.7398	0.9212	0.7465	0.8489	0.7408	0.9268	0.6928	0.956	0.8076	1.0271	0.9044	1.0607	0.9688	1.1443	0.9956	1.1259	1.008	1.1015	1.0446
Acetazolamide		0.7093	0.8315	0.7445	0.8605	0.7346	0.8633	0.6967	0.8576	0.7305	0.8312	0.802	0.9197	0.8211	0.977	0.9027	1.0315	0.973	1.1084	0.9811	1.1058
		0.8072	0.7283	0.8608	0.6952	0.9012	0.7583	0.8951	0.7404	0.8284	0.7256	0.929	0.7563	0.9911	0.8546	1.0812	0.9943	1.134	1.0468	1.1467	0.9422

Table S3. Results of thermal shift and orthogonal colorimetric enzymatic activity assays (with IC_{50} determination) for 15 primary hits (with acetazolamide as a control, $n = 4$)

Entry	Compound	hCA-II		hCA-IX	
		ΔT_m, median (at 40 μM)	IC_{50}, μM	ΔT_m, median (at 40 μM)	IC_{50}, μM
1	**4**{*17*,*7*,*27*}	0.21	6.07	1.44	23.4
2	**7**{*2*,*21*,*60*}	0.70	8.74	1.17	17.3
3	**4**{*16*,*7*,*28*}	–2.55	1.70	1.18	7.13
4	**4**{*16*,*7*,*19*}	–4.95	0.20	1.17	1.11
5	**4**{*18*,*7*,*32*}	<0.5	>100	0.99	84.2[a]
6	**4**{*10*,*6*,*48*}	<0.5	>100	–1.28	>100
7	**7**{*2*,*1*,*17*}	<0.5	>100	–2.24	>100
8	**4**{*19*,*6*,*48*}	<0.5	>100	–2.92	>100
9	**7**{*3*,*19*,*57*}	1.06	>100	–3.53	>100
10	**7**{*2*,*9*,*29*}	–8.76	>100	–3.88	>100
11	**7**{*2*,*44*,*77*}	<0.5	>100	–4.14	>100
12	**7**{*1*,*16*,*5*}	<0.5	>100	–4.93	>100
13	**7**{*3*,*43*,*23*}	<0.5	>100	–4.99	>100
14	**7**{*2*,*4*,*21*}	–7.12	>100	–7.51	>100
15	**4**{*2*,*8*,*3*}	<0.5	>100	–1.45	>100
16	Acetazolamide	N/A[b]	0.034	N/A[b]	0.243

[a] Inhibition at high concentration might be related to the low solubility or non-specific binding
[b] The data is not available

In silico screening details

Molecular Docking. X-ray crystal structures of CA-II with the inhibitor SUA (PDB ID: 3K34),[4] and CA-IX with the inhibitor VR16-10 (PDB ID: 6G9U)[5] were superimposed and used to create receptor maps. The receptors and a set of acyl sulfonamide derivatives were prepared for molecular docking using Molsoft ICM-Pro 3.9-3b.[6] The binding site was defined with a 5 Å radius around the corresponding ligand in each co-crystal structure. Water molecules and other low molecular-weight solutes were removed to generate the receptor map. During the docking process, an APF template was employed to guide the placement of the sulfonamide group within the binding pocket. The APF template was derived from the sulfonamide group of the ligand in the respective structure file. Although the template was not enforced as a strict constraint during docking, it helped to efficiently identify the correct ligand conformation. The template locations are provided in Figures S6 and S7. ICM-Pro was used to dock the acyl sulfonamide derivatives, applying the default box size and an effort value of 2.0. Molsoft ICM generates conformers on the fly during the docking process using the Monte Carlo algorithm. Ligand-receptor complexes were evaluated using the ICM-VLS empirical scoring function, which incorporates van der Waals energy, electrostatics, hydrogen bonding, conformational entropy loss, and solvation electrostatic energy change.[7,8] Visual analysis of the docking results was performed using Molsoft ICM-Pro 3.9-3b.

Molecular dynamics simulations and mmGBSA calculations. MD simulations were carried out for 5 ns for complexes of carbonic anhydrase II/IX that contained Zn atom in active site and ligands, using GROMACS (version 2023.2) software package[9] employing the Amber ff99SB-ILDN all-atom force field for protein and GAFF for ligands. The system was solvated in a cubic box using the TIP3P water model and neutralized by adding Na^+ and Cl^- counterions with final ions concentration 0.15 M. After that, each system was energy minimized using the steepest descent algorithm for 100,000 steps to remove steric clashes. Following this, the systems were equilibrated at 310 K under NVT conditions for 500 ps, followed by 500 ps under NPT conditions at the same temperature and 1 atm pressure. During the equilibrations, the heavy atoms of the protein and ligand were restrained with a force constant of 1000 kJ mol^{-1} nm^{-2}, which allows the water molecules to adjust around the solute. Production runs were then carried out under NPT conditions (310 K, 1 atm) with an integration time step of 2 fs. We used a 300 kJ mol^{-1} nm^{-2} constraint on the protein's backbone atoms to prevent structural drift due to force field inaccuracies. The temperature was controlled using the v-rescale thermostat with a coupling constant of 0.1 ps, while the pressure was maintained via the Parrinello-Rahman barostat with a 5 ps coupling constant. Bonds involving hydrogen atoms were constrained using the LINCS algorithm. Long-range electrostatic interactions were computed using the smooth particle-mesh Ewald (PME) method. Cutoffs of 1.0 nm were used to treat the short-range electrostatic and van der Waals interactions; the potential-shift-Verlet algorithm was applied to take care of both interactions by smoothly shifting beyond the cutoff.

The molecular mechanics-generalized Born surface area [MM/GBSA] method was used to calculate binding free energy for all complexes, using the gmx_MMPBSA tool (Figure S5).[10] MM-PBSA calculates the binding free energy (ΔG_{bind}) of a complex using the following equation:

$$\Delta G_{\text{bind}} = G_{\text{complex}} - (G_{\text{protein}} + G_{\text{ligand}})$$

Each term is free energy that was estimated as

$$G = E_{\text{MM}} + G_{\text{solv}} - TS$$

where E_{MM} – molecular mechanics energy (bonded + van der Waals + electrostatic terms); G_{solv} – solvation free energy (the polar part was calculated by solving the Poisson–Boltzmann equation, and the non-polar part was estimated from solvent-acceSible surface area); TS – entropic term.

Firstly, output trajectories were fitted and PBC conditions were removed; 100 frames from the final 2 ns of trajectory were selected for calculations. The solvation energy was calculated using the GBNECK2 implicit solvent model (igb=8) with the mbondi2 radii set. The solvent accessible surface area was estimated using the LCPO algorithm. An external dielectric constant of 80 and a solute dielectric constant of 4 were applied.

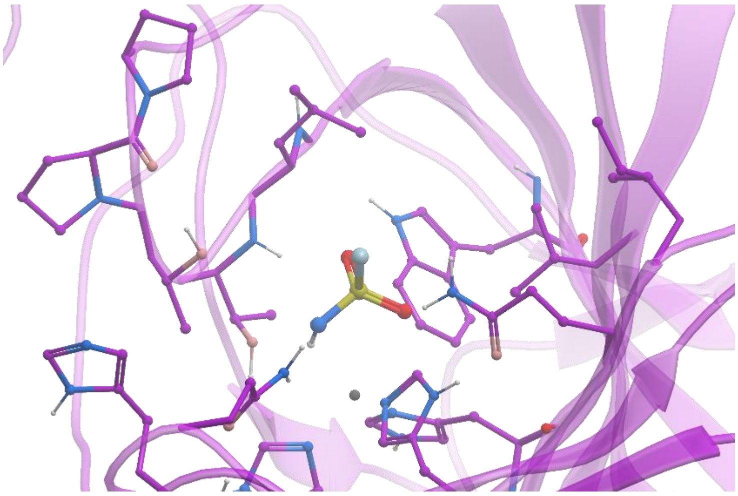

Figure S6. Template for hCA-II.

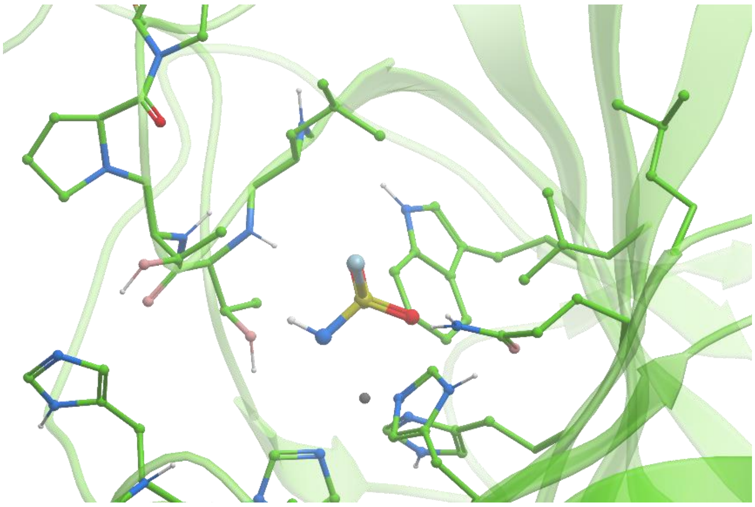

Figure S7. Template for hCA-IX.

Table S5. Molecular dynamics results for the most active compounds.

	hCA-II, MM/PB(GB)SA, kcal/mol					hCA-IX, MM/PB(GB)SA, kcal/mol			
Compound	Parameter 9 GB, *igb* = 8, *intdiel* = 4.0	Parameter 10, PB, *indiel* = 1.0	Parameter 11 PB, *indiel* = 2.0	Parameter 12 PB, *indiel* = 4.0		Parameter 9 GB, *igb* = 8, *intdiel* = 4.0	Parameter 10, PB, *indiel* = 1.0	Parameter 11 PB, *indiel* = 2.0	Parameter 12 PB, *indiel* = 4.0
**4**{*17,7,27*}	–43.59	–11.07	–28.8	–37.69		–68.09	–33.91	–42.76	–47.24
**7**{*2,21,60*}	–70.35	–47.59	–46.8	–46.4		–61.09	–14.55	–30.62	–38.36
**4**{*16,7,28*}	–66.01	–46.5	–44.81	–43.91		–60.09	–44.35	–40.86	–38.98
**4**{*16,7,19*}	–67.26	–42.47	–42.48	–42.44		–52.6	–36.06	–38.16	–39.19
**4**{*18,7,32*}	–36.74	–14.87	–27.42	–33.74		–65.25	–35.42	–38.57	–39.84

Characterization data for the synthesized compounds

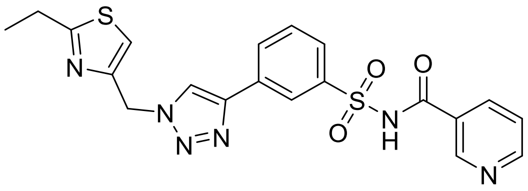

***N*-((3-(1-((2-Ethylthiazol-4-yl)methyl)-1*H*-1,2,3-triazol-4-yl)phenyl)sulfonyl)nicotinamide (4{32,6,17}).**
Yield 99.7 mg (100%); colorless glass solid.
^{1}H NMR (500 MHz, DMSO-d_6) δ 12.70 (br s, 1H), 9.13 – 8.74 (m, 3H), 8.50 (t, J = 1.9 Hz, 1H), 8.28 (d, J = 7.8 Hz, 1H), 8.15 (d, J = 7.8 Hz, 1H), 8.00 – 7.91 (m, 1H), 7.71 (t, J = 7.8 Hz, 1H), 7.62 – 7.46 (m, 2H), 5.72 (s, 2H), 2.95 (q, J = 7.5 Hz, 2H), 1.26 (t, J = 7.5 Hz, 3H).
^{13}C NMR (126 MHz, DMSO-d_6) δ 173.1, 164.4, 152.6, 149.2, 148.7, 144.9, 140.6, 136.8, 131.4, 131.2, 130.0, 129.8, 129.7, 126.8, 123.9, 122.6, 117.6, 49.2, 26.1, 13.8.
LC/MS (ES-API) m/z = 455 [M+H]$^+$.
Anal. calcd. for $C_{20}H_{18}N_6O_3S_2$: C 52.85; H 3.99; N 18.49; S 14.11. Found: C 53.04; H 3.61; N 18.11; S 14.30.

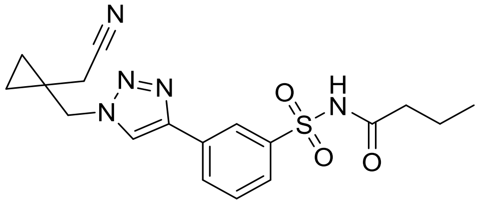

***N*-((3-(1-((1-(Cyanomethyl)cyclopropyl)methyl)-1*H*-1,2,3-triazol-4-yl)phenyl)sulfonyl)butyramide (4{33,6,4}).** Yield 99.5 mg (100%); yellowish oil.
^{1}H NMR (500 MHz, DMSO-d_6) δ 12.13 (br s, 1H), 8.82 (s, 1H), 8.41 (s, 1H), 8.16 (d, J = 7.9 Hz, 1H), 7.86 (d, J = 7.9 Hz, 1H), 7.71 (t, J = 7.9 Hz, 1H), 4.44 (s, 2H), 2.59 (s, 2H), 2.19 (t, J = 7.2 Hz, 2H), 1.51 – 1.34 (m, 2H), 0.99 – 0.85 (m, 2H), 0.80 – 0.61 (m, 5H).
^{13}C NMR (151 MHz, DMSO-d_6) δ 171.7, 144.9, 140.4, 131.5, 130.0, 129.8, 126.5, 123.7, 122.6, 118.5, 55.4, 37.2, 22.4, 18.2, 17.5, 13.1, 10.8.
LC/MS (ES-API) m/z = 388 [M+H]$^+$.
Anal. calcd. for $C_{18}H_{21}N_5O_3S$: C 55.8; H 5.46; N 18.08; S 8.27. Found: C 55.91; H 5.75; N 17.83; S 7.88.

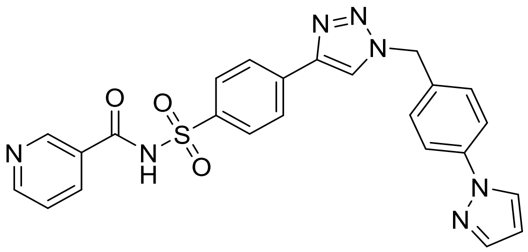

***N*-((4-(1-(4-(1*H*-Pyrazol-1-yl)benzyl)-1*H*-1,2,3-triazol-4-yl)phenyl)sulfonyl)nicotinamide (4{18,7,17}).**
Yield 97.2mg (97%); yellowish viscous oil.
^{1}H NMR (500 MHz, DMSO-d_6) δ 12.85 (br s, 1H), 9.18 – 8.72 (m, 3H), 8.49 (d, J = 2.6 Hz, 1H), 8.26 (d, J = 8.0 Hz, 1H), 8.10 (d, J = 8.6 Hz, 2H), 8.06 (d, J = 8.6 Hz, 2H), 7.89 – 7.81 (m, 2H), 7.74 (d, J = 1.7 Hz, 1H), 7.61 – 7.43 (m, 3H), 6.54 (t, J = 2.1 Hz, 1H), 5.72 (s, 2H).
^{13}C NMR (151 MHz, DMSO-d_6) δ 164.4, 152.8, 148.8, 148.2, 145.2, 141.1, 139.5, 138.5, 136.6, 135.4, 133.4, 129.3, 128.5, 127.8, 125.4, 123.9, 123.1, 118.6, 108.0, 52.6.
LC/MS (ES-API) m/z = 486 [M+H]$^+$.
Anal. calcd. for $C_{24}H_{19}N_7O_3S$: C 59.37; H 3.94; N 20.19; S 6.60. Found: C 59.51; H 3.54; N 20.49; S 6.73.

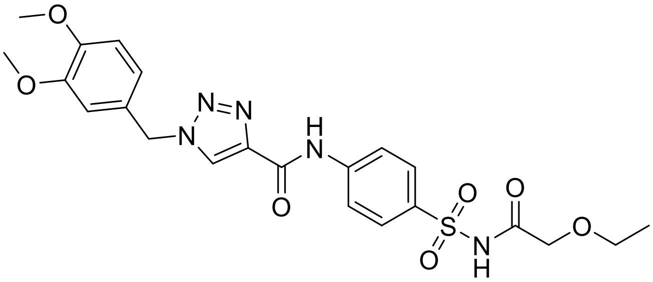

1-(3,4-Dimethoxybenzyl)-*N*-(4-(*N*-(2-ethoxyacetyl)sulfamoyl)phenyl)-1*H*-1,2,3-triazole-4-carboxamide (4{1,8,1}). Yield 96.9mg (97%); yellowish glass solid.
^{1}H NMR (500 MHz, DMSO-d_6) δ 12.02 (br s, 1H), 10.89 (s, 1H), 8.84 (s, 1H), 8.06 (d, J = 8.8 Hz, 2H), 7.89 (d, J = 8.8 Hz, 2H), 7.08 (d, J = 1.8 Hz, 1H), 6.96 – 6.85 (m, 2H), 5.60 (s, 2H), 3.93 (s, 2H), 3.75 (s, 3H), 3.74 (s, 3H), 3.39 (q, J = 7.0 Hz, 2H), 1.06 (t, J = 7.0 Hz, 3H).
^{13}C NMR (126 MHz, DMSO-d_6) δ 168.9, 158.7, 148.9, 148.8, 143.2, 142.4, 133.5, 128.5, 127.6, 127.5, 120.8, 119.8, 112.2, 112.0, 68.7, 66.2, 55.5, 53.2, 14.8.
LC/MS (ES-API) *m/z* = 504 [M+H]$^+$.
Anal. calcd. for $C_{22}H_{25}N_5O_7S$: C 52.48; H 5.00; N 13.91; S 6.37. Found: C 52.16; H 4.98; N 14.03; S 6.34.

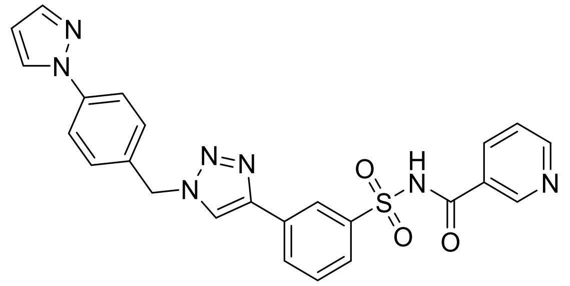

***N*-((3-(1-(4-(1*H*-Pyrazol-1-yl)benzyl)-1*H*-1,2,3-triazol-4-yl)phenyl)sulfonyl)nicotinamide (4{18,6,17}).** Yield 99.5 mg (100%); yellowish glass solid.
^{1}H NMR (500 MHz, DMSO-d_6) δ 12.90 (br s, 1H), 9.25 – 8.78 (m, 3H), 8.56 – 8.40 (m, 2H), 8.28 (d, J = 8.0 Hz, 1H), 8.14 (d, J = 8.0 Hz, 1H), 7.95 (d, J = 8.3 Hz, 1H), 7.86 (d, J = 8.3 Hz, 2H), 7.78 – 7.64 (m, 2H), 7.62 – 7.43 (m, 3H), 6.54 (t, J = 2.1 Hz, 1H), 5.71 (s, 2H).
^{13}C NMR (151 MHz, DMSO-d_6) δ 164.5, 152.6, 148.7, 145.1, 141.1, 140.7, 139.5, 136.8, 133.5, 131.4, 129.9, 129.8, 129.3, 127.8, 126.9, 124.0, 123.9, 122.5, 118.6, 108.0, 52.6.
LC/MS (ES-API) *m/z* = 486 [M+H]$^+$.
Anal. calcd. for $C_{24}H_{19}N_7O_3S$: C 59.37; H 3.94; N 20.19; S 6.60. Found: C 59.22; H 4.27; N 20.37; S 6.57.

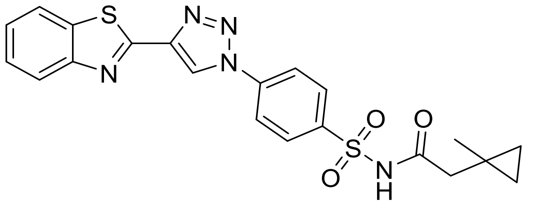

***N*-((4-(4-(Benzo[*d*]thiazol-2-yl)-1*H*-1,2,3-triazol-1-yl)phenyl)sulfonyl)-2-(1-methylcyclopropyl)acetamide (7{3,37,71}).** Yield 83.7mg (84%); yellowish oil.
^{1}H NMR (500 MHz, DMSO-d_6) δ 12.18 (br s, 1H), 9.82 (s, 1H), 8.34 (d, J = 8.6 Hz, 2H), 8.21 (d, J = 7.8 Hz, 1H), 8.16 (d, J = 8.6 Hz, 2H), 8.07 (d, J = 7.8 Hz, 1H), 7.59 (t, J = 7.8 Hz, 1H), 7.51 (t, J = 7.8 Hz, 1H), 2.15 (s, 2H), 0.93 (s, 3H), 0.35 (t, J = 4.7 Hz, 2H), 0.23 (t, J = 4.7 Hz, 2H).
^{13}C NMR (126 MHz, DMSO-d_6) δ 170.5, 158.2, 153.2, 143.1, 139.6, 139.4, 133.9, 129.5, 126.7, 125.7, 122.7, 122.5, 122.4, 120.8, 44.3, 22.7, 13.1, 12.1, 1.1.
LC/MS (ES-API) *m/z* = 454 [M+H]$^+$.
Anal. calcd. for $C_{21}H_{19}N_5O_3S_2$: C 55.61; H 4.22; N 15.44; S 14.14. Found: C 55.68; H 4.03; N 15.58; S 13.80.

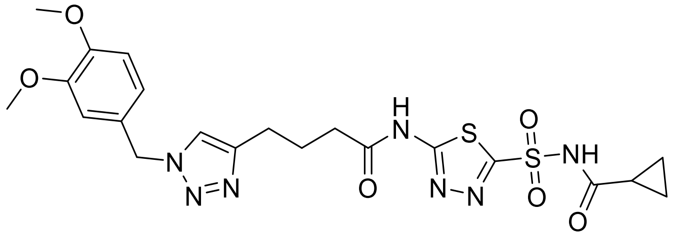

***N*-((5-(4-(1-(3,4-Dimethoxybenzyl)-1*H*-1,2,3-triazol-4-yl)butanamido)-1,3,4-thiadiazol-2-yl)sulfonyl)-cyclopropanecarboxamide (4{1,5,2}).** Yield 99.4 mg (99%); colorless glass solid.
^{1}H NMR (500 MHz, DMSO-d_6) δ 13.57 – 13.02 (m, 2H), 7.87 (s, 1H), 6.96 (s, 1H), 6.90 (d, J = 8.1 Hz, 1H), 6.81 (d, J = 8.1 Hz, 1H), 5.41 (s, 2H), 3.77 – 3.61 (m, 6H), 2.63 (t, J = 7.4 Hz, 2H), 2.57 (t, J = 7.4 Hz, 2H), 1.92 (p, J = 7.4 Hz, 2H), 1.78 – 1.65 (m, 1H), 0.95 – 0.83 (m, 2H), 0.80 – 0.71 (m, 2H).
^{13}C NMR (151 MHz, DMSO-d_6) δ 173.2, 172.2, 162.3, 160.6, 148.7, 148.6, 146.3, 128.3, 121.9, 120.5, 112.0, 111.9, 55.52, 55.49, 52.6, 34.2, 24.3, 24.1, 14.1, 9.2.
LC/MS (ES-API) *m/z* = 536 [M+H]$^+$.

Anal. calcd. for $C_{21}H_{25}N_7O_6S_2$: C 47.09; H 4.71; N 18.31; S 11.97. Found: C 46.90; H 5.05; N 18.11; S 12.23.

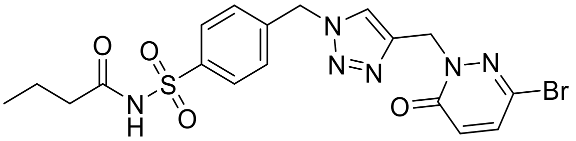

***N*-((4-((4-((3-Bromo-6-oxopyridazin-1(6*H*)-yl)methyl)-1*H*-1,2,3-triazol-1-yl)methyl)phenyl)sulfonyl)-butyramide (7{1,46,4}).** Yield 90.0 mg (90%); amber glass solid.

^{1}H NMR (500 MHz, DMSO-d_6) δ 12.06 (br s, 1H), 8.22 (s, 1H), 7.89 (d, *J* = 8.1 Hz, 2H), 7.61 (d, *J* = 9.7 Hz, 1H), 7.48 (d, *J* = 8.1 Hz, 2H), 6.97 (d, *J* = 9.7 Hz, 1H), 5.71 (s, 2H), 5.26 (s, 2H), 2.16 (t, *J* = 7.3 Hz, 2H), 1.40 (h, *J* = 7.3 Hz, 2H), 0.73 (t, *J* = 7.3 Hz, 3H).

^{13}C NMR (151 MHz, DMSO-d_6) δ 171.5, 158.0, 141.9, 141.6, 139.1, 137.1, 131.9, 128.3, 127.9, 126.4, 124.7, 52.1, 46.6, 37.1, 17.4, 13.1.

LC/MS (ES-API) *m/z* = 495/497 as 1/1 $[M+H]^+$.

Anal. calcd. for $C_{18}H_{19}BrN_6O_4S$: C 43.65; H 3.87; N 16.97; S 6.47; Br 16.13. Found: C 43.52; H 3.64; N 17.31; S 6.29; Br 16.32.

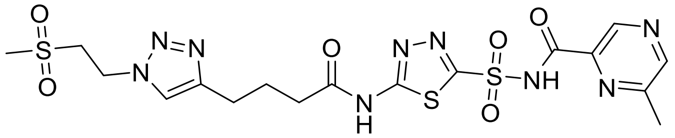

6-Methyl-*N*-((5-(4-(1-(2-(methylsulfonyl)ethyl)-1*H*-1,2,3-triazol-4-yl)butanamido)-1,3,4-thiadiazol-2-yl)-sulfonyl)pyrazine-2-carboxamide (4{45,5,30}). Yield 96.6 mg (97%); yellowish viscous oil.

^{1}H NMR (500 MHz, DMSO-d_6) δ 12.92 (br s, 2H), 8.91 (s, 1H), 8.69 (s, 1H), 7.98 (s, 1H), 4.75 (t, *J* = 7.0 Hz, 2H), 3.77 (t, *J* = 7.0 Hz, 2H), 2.94 (s, 3H), 2.67 (t, *J* = 7.6 Hz, 2H), 2.60 – 2.51 (m, 5H), 1.94 (p, *J* = 7.6 Hz, 2H).

^{13}C NMR (151 MHz, DMSO-d_6) δ 171.8, 166.2, 163.4, 161.5, 152.8, 146.8, 146.2, 145.5, 141.5, 122.6, 53.0, 43.1, 40.7, 34.1, 24.3, 24.2, 20.9.

LC/MS (ES-API) *m/z* = 542 $[M–H]^-$.

Anal. calcd. for $C_{17}H_{21}N_9O_6S_3$: C 37.56; H 3.89; N 23.19; S 17.69. Found: C 37.53; H 3.84; N 23.03; S 17.93.

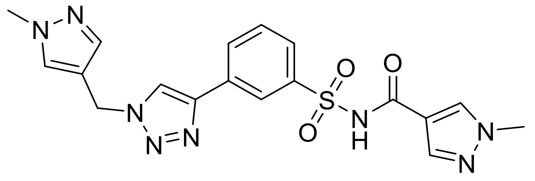

1-Methyl-*N*-((3-(1-((1-methyl-1*H*-pyrazol-4-yl)methyl)-1*H*-1,2,3-triazol-4-yl)phenyl)sulfonyl)-1*H*-pyrazole-4-carboxamide (4{34,6,47}). Yield 94.7 mg (95%); yellowish oil.

^{1}H NMR (500 MHz, DMSO-d_6) δ 12.24 (br s, 1H), 8.70 (s, 1H), 8.44 (s, 1H), 8.29 (s, 1H), 8.10 (d, *J* = 8.0 Hz, 1H), 7.94 (s, 1H), 7.88 (d, *J* = 8.0 Hz, 1H), 7.81 (s, 1H), 7.68 (t, *J* = 8.0 Hz, 1H), 7.52 (s, 1H), 5.51 (s, 2H), 3.83 (s, 3H), 3.81 (s, 3H).

^{13}C NMR (151 MHz, DMSO-d_6) δ 160.0, 144.9, 140.7, 139.8, 138.4, 134.0, 131.5, 130.5, 129.9, 129.8, 126.6, 123.7, 121.8, 115.6, 115.2, 44.1, 38.9, 38.5.

LC/MS (ES-API) *m/z* = 427 $[M+H]^+$.

Anal. calcd. for $C_{18}H_{18}N_8O_3S$: C 50.70; H 4.25; N 26.28; S 7.52. Found: C 50.79; H 4.62; N 26.56; S 7.56.

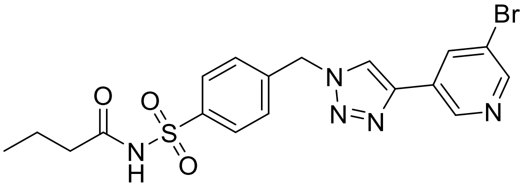

***N*-((4-((4-(5-Bromopyridin-3-yl)-1*H*-1,2,3-triazol-1-yl)methyl)phenyl)sulfonyl)butyramide (7{1,35,4}).** Yield 56.7 mg (57%); amber glass solid.

^{1}H NMR (500 MHz, DMSO-d_6) δ 12.08 (br s, 1H), 9.06 (s, 1H), 8.87 (s, 1H), 8.68 (s, 1H), 8.47 (t, *J* = 2.1 Hz, 1H), 7.93 (d, *J* = 8.2 Hz, 2H), 7.55 (d, *J* = 8.2 Hz, 2H), 5.83 (s, 2H), 2.16 (t, *J* = 7.3 Hz, 2H), 1.40 (h, *J* = 7.3 Hz, 2H), 0.74 (t, *J* = 7.3 Hz, 3H).

^{13}C NMR (151 MHz, DMSO-d_6) δ 171.5, 149.4, 144.8, 142.6, 141.2, 139.3, 134.7, 128.4, 128.3, 128.0, 123.5, 120.6, 52.5, 37.1, 17.4, 13.1.

LC/MS (ES-API) *m/z* = 464/466 as 1/1 $[M+H]^+$.

Anal. calcd. for $C_{18}H_{18}BrN_5O_3S$: C 46.56; H 3.91; N 15.08; S 6.90; Br 17.21. Found: C 46.48; H 3.65; N 14.98; S 7.08; Br 17.23.

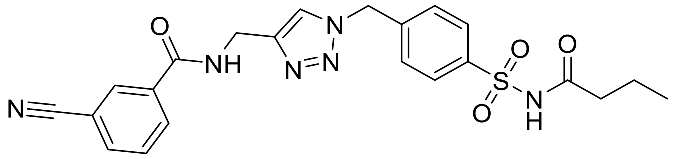

***N*-((1-(4-(*N*-Butyrylsulfamoyl)benzyl)-1*H*-1,2,3-triazol-4-yl)methyl)-3-cyanobenzamide (7{1,47,4}).**
Yield 69.3 mg (69%); colorless glass solid.
^{1}H NMR (500 MHz, DMSO-d_6) δ 12.04 (br s, 1H), 9.21 (t, *J* = 5.6 Hz, 1H), 8.26 (s, 1H), 8.15 (d, *J* = 7.8 Hz, 1H), 8.12 (s, 1H), 7.99 (d, *J* = 7.8 Hz, 1H), 7.88 (d, *J* = 8.0 Hz, 2H), 7.68 (t, *J* = 7.8 Hz, 1H), 7.48 (d, *J* = 8.0 Hz, 2H), 5.68 (s, 2H), 4.52 (d, *J* = 5.6 Hz, 2H), 2.14 (t, *J* = 7.4 Hz, 2H), 1.38 (h, *J* = 7.4 Hz, 2H), 0.71 (t, *J* = 7.4 Hz, 3H).
^{13}C NMR (151 MHz, DMSO-d_6) δ 171.6, 164.3, 145.0, 141.7, 139.1, 135.1, 134.7, 132.1, 130.9, 129.8, 128.4, 127.9, 123.6, 118.3, 111.5, 52.0, 37.1, 35.0, 17.4, 13.1.
LC/MS (ES-API) *m/z* = 467 $[M+H]^+$.
Anal. calcd. for $C_{22}H_{22}N_6O_4S$: C 56.64; H 4.75; N 18.01; S 6.87. Found: C 56.25; H 4.90; N 17.89; S 6.65.

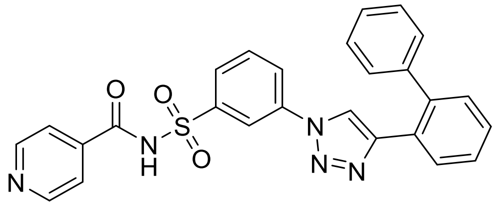

***N*-((3-(4-([1,1'-Biphenyl]-2-yl)-1*H*-1,2,3-triazol-1-yl)phenyl)sulfonyl)isonicotinamide (7{2,18,50}).**
Yield 93.6 mg (94%); colorless oil.
^{1}H NMR (500 MHz, DMSO-d_6) δ 8.82 (d, *J* = 5.5 Hz, 2H), 8.30 (t, *J* = 2.0 Hz, 1H), 8.11 (s, 1H), 8.02 (d, *J* = 5.5 Hz, 2H), 7.99 (d, *J* = 7.9 Hz, 1H), 7.90 – 7.82 (m, 2H), 7.73 (t, *J* = 7.9 Hz, 1H), 7.56 – 7.48 (m, 2H), 7.45 – 7.40 (m, 1H), 7.39 – 7.22 (m, 5H), 3.81 (br s, 1H).
^{13}C NMR (151 MHz, DMSO-d_6) δ 165.0, 147.0, 146.8, 144.0, 140.7, 140.5, 136.1, 130.4, 129.5, 129.1, 128.7, 128.5, 128.3, 127.7, 127.3, 127.2, 123.5, 123.1, 121.3, 118.7.
LC/MS (ES-API) *m/z* = 482 $[M+H]^+$.
Anal. calcd. for $C_{26}H_{19}N_5O_3S$: C 64.85; H 3.98; N 14.54; S 6.66. Found: C 65.15; H 4.23; N 14.14; S 7.05.

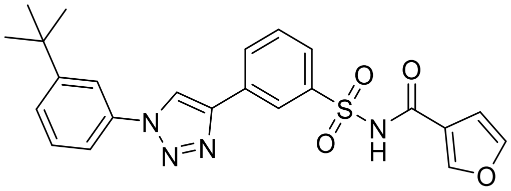

***N*-((3-(1-(3-(*tert*-Butyl)phenyl)-1*H*-1,2,3-triazol-4-yl)phenyl)sulfonyl)furan-3-carboxamide (4{10,6,48}).**
Yield 92.7 mg (93%); yellowish oil.
^{1}H NMR (500 MHz, DMSO-d_6) δ 12.50 (br s, 1H), 9.52 (s, 1H), 8.58 (t, *J* = 1.9 Hz, 1H), 8.50 – 8.44 (m, 1H), 8.29 – 8.24 (m, 1H), 8.02 – 7.91 (m, 2H), 7.83 – 7.75 (m, 3H), 7.63 – 7.50 (m, 2H), 6.85 (d, *J* = 1.9 Hz, 1H), 1.37 (s, 9H).
^{13}C NMR (151 MHz, DMSO-d_6) δ 160.2, 153.0, 148.1, 145.6, 144.8, 140.4, 136.4, 131.2, 130.3, 130.0, 129.6, 127.0, 125.8, 124.1, 120.7, 120.4, 117.5, 117.1, 109.1, 34.8, 30.9.
LC/MS (ES-API) *m/z* = 451 $[M+H]^+$.
Anal. calcd. for $C_{23}H_{22}N_4O_4S$: C 61.32; H 4.92; N 12.44; S 7.12. Found: C 61.60; H 4.84; N 12.51; S 7.23.

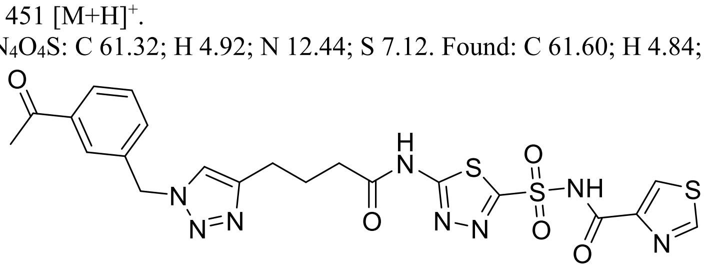

***N*-((5-(4-(1-(3-Acetylbenzyl)-1*H*-1,2,3-triazol-4-yl)butanamido)-1,3,4-thiadiazol-2-yl)sulfonyl)thiazole-4-carboxamide (4{11,5,80}).** Yield 90.6 mg (91%); yellowish glass solid.
^{1}H NMR (500 MHz, DMSO-d_6) δ 13.07 (br s, 2H), 9.20 (d, *J* = 1.9 Hz, 1H), 8.59 (d, *J* = 1.9 Hz, 1H), 7.99 (s, 1H), 7.95 – 7.87 (m, 2H), 7.56 – 7.49 (m, 2H), 5.63 (s, 2H), 2.67 (t, *J* = 7.5 Hz, 2H), 2.62 – 2.55 (m, 5H), 1.95 (p, *J* = 7.5 Hz, 2H).
^{13}C NMR (101 MHz, DMSO-d_6) δ 197.6, 172.1, 162.1, 161.8, 160.9, 155.5, 148.9, 146.5, 137.1, 136.8, 132.6, 129.2, 128.7, 128.1, 127.4, 122.4, 52.3, 34.2, 26.8, 24.3, 24.1.
LC/MS (ES-API) *m/z* = 561 $[M+H]^+$.
Anal. calcd. for $C_{21}H_{20}N_8O_5S_3$: C 44.99; H 3.60; N 19.99; S 17.16. Found: C 45.31; H 3.89; N 19.72; S 17.04.

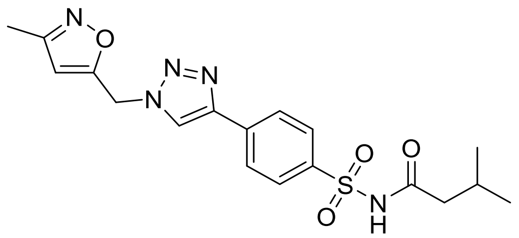

3-Methyl-*N*-((4-(1-((3-methylisoxazol-5-yl)methyl)-1*H*-1,2,3-triazol-4-yl)phenyl)sulfonyl)butanamide (4{20,7,16}). Yield 88.0 mg (88%); yellowish viscous oil.
^{1}H NMR (500 MHz, DMSO-*d*$_6$) δ 12.07 (br s, 1H), 8.86 (s, 1H), 8.10 (d, *J* = 8.6 Hz, 2H), 7.97 (d, *J* = 8.6 Hz, 2H), 6.46 (s, 1H), 5.93 (s, 2H), 2.23 (s, 3H), 2.07 (d, *J* = 7.2 Hz, 2H), 1.93 – 1.82 (m, 1H), 0.76 (d, *J* = 6.6 Hz, 6H).
^{13}C NMR (151 MHz, DMSO-*d*$_6$) δ 171.0, 165.4, 160.0, 145.2, 138.4, 135.1, 128.3, 125.5, 123.5, 104.9, 44.7, 44.3, 25.1, 21.8, 10.9.
LC/MS (ES-API) *m/z* = 404 [M+H]$^+$.
Anal. calcd. for $C_{18}H_{21}N_5O_4S$: C 53.59; H 5.25; N 17.36; S 7.95. Found: C 53.47; H 5.10; N 17.23; S 7.75.

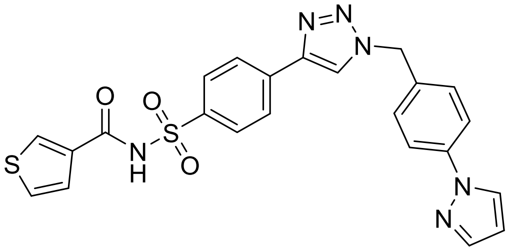

***N*-((4-(1-(4-(1*H*-Pyrazol-1-yl)benzyl)-1*H*-1,2,3-triazol-4-yl)phenyl)sulfonyl)thiophene-3-carboxamide (4{18,7,32}).** Yield 86.4 mg (86%); colorless glass solid.
^{1}H NMR (500 MHz, DMSO-*d*$_6$) δ 12.44 (br s, 1H), 8.83 (s, 1H), 8.49 (s, 1H), 8.45 (s, 1H), 8.10 (d, *J* = 8.3 Hz, 2H), 8.04 (d, *J* = 7.4 Hz, 2H), 7.86 (d, *J* = 8.1 Hz, 2H), 7.74 (s, 1H), 7.63 – 7.58 (m, 1H), 7.54 – 7.44 (m, 3H), 6.60 – 6.44 (m, 1H), 5.72 (s, 2H).
^{13}C NMR (151 MHz, DMSO-*d*$_6$) δ 160.4, 145.1, 141.1, 139.5, 138.5, 135.4, 134.7, 133.4, 132.9, 129.3, 128.5, 127.8, 127.5, 127.2, 125.4, 123.1, 118.6, 108.0, 52.6.
LC/MS (ES-API) *m/z* = 491 [M+H]$^+$.
Anal. calcd. for $C_{23}H_{18}N_6O_3S_2$: C 56.31; H 3.70; N 17.13; S 13.07. Found: C 56.68; H 3.70; N 17.03; S 12.73.

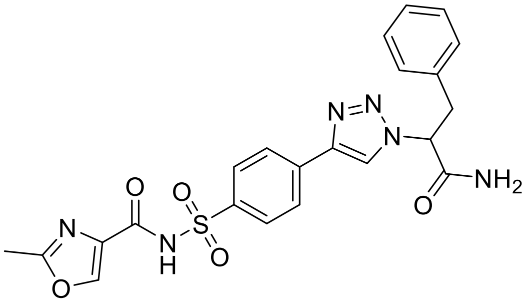

***N*-((4-(1-(1-Amino-1-oxo-3-phenylpropan-2-yl)-1*H*-1,2,3-triazol-4-yl)phenyl)sulfonyl)-2-methyloxazole-4-carboxamide (4{21,7,33}).** Yield 86.4 mg (86%); colorless glass solid.
^{1}H NMR (500 MHz, DMSO-*d*$_6$) δ 12.42 (br s, 1H), 9.02 (s, 1H), 8.68 (s, 1H), 8.08 (d, *J* = 8.2 Hz, 2H), 8.03 (d, *J* = 8.2 Hz, 2H), 7.99 (s, 1H), 7.53 (s, 1H), 7.32 – 7.09 (m, 5H), 5.64 (dd, *J* = 10.5, 5.4 Hz, 1H), 3.50 (dd, *J* = 14.3, 5.4 Hz, 1H), 3.42 (dd, *J* = 14.3, 10.5 Hz, 1H), 2.44 (s, 3H).
^{13}C NMR (151 MHz, DMSO-*d*$_6$) δ 169.0, 161.8, 158.8, 144.5, 144.4, 138.3, 136.2, 135.6, 133.8, 128.8, 128.5, 128.3, 126.8, 125.2, 122.7, 64.2, 37.5, 13.3.
LC/MS (ES-API) *m/z* = 481 [M+H]$^+$.
Anal. calcd. for $C_{22}H_{20}N_6O_5S$: C 54.99; H 4.20; N 17.49; S 6.67. Found: C 55.11; H 4.59; N 17.20; S 6.45.

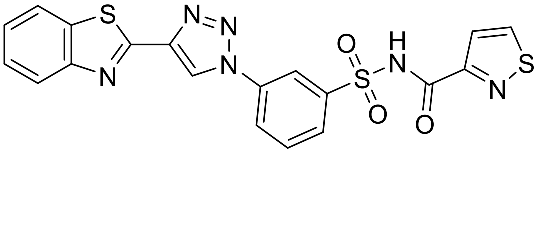

***N*-((3-(4-(Benzo[*d*]thiazol-2-yl)-1*H*-1,2,3-triazol-1-yl)phenyl)sulfonyl)isothiazole-3-carboxamide (7{2,37,72}).** Yield 46.4 mg (46%); colorless glass solid.
^{1}H NMR (500 MHz, DMSO-d_6) δ 12.82 (br s, 1H), 9.81 (s, 1H), 9.18 (d, J = 4.8 Hz, 1H), 8.69 (s, 1H), 8.40 (d, J = 7.7 Hz, 1H), 8.21 (d, J = 8.0 Hz, 1H), 8.16 (d, J = 7.7 Hz, 1H), 8.07 (d, J = 8.0 Hz, 1H), 7.94 (t, J = 8.0 Hz, 1H), 7.81 (d, J = 4.8 Hz, 1H), 7.59 (t, J = 7.7 Hz, 1H), 7.51 (t, J = 7.7 Hz, 1H).
^{13}C NMR (126 MHz, DMSO-d_6) δ 160.3, 159.3, 158.2, 153.2, 153.1, 143.1, 141.2, 136.4, 133.9, 131.0, 128.0, 126.7, 125.7, 125.3, 124.8, 122.7, 122.5, 119.5.
LC/MS (ES-API) m/z = 469 [M+H]$^+$.
Anal. calcd. for $C_{19}H_{12}N_6O_3S_3$: C 48.71; H 2.58; N 17.94; S 20.53. Found: C 48.94; H 2.20; N 17.77; S 20.36.

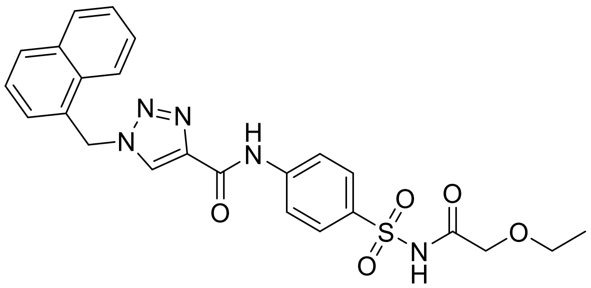

***N*-(4-(*N*-(2-Ethoxyacetyl)sulfamoyl)phenyl)-1-(naphthalen-1-ylmethyl)-1*H*-1,2,3-triazole-4-carboxamide (4{2,8,1}).** Yield 85.6 mg (86%); yellowish glass solid.
^{1}H NMR (500 MHz, DMSO-d_6) δ 12.03 (br s, 1H), 10.87 (s, 1H), 8.83 (s, 1H), 8.22 (d, J = 8.4 Hz, 1H), 8.07 – 7.93 (m, 4H), 7.88 (d, J = 8.4 Hz, 2H), 7.66 – 7.51 (m, 3H), 7.48 (d, J = 7.0 Hz, 1H), 6.21 (s, 2H), 3.93 (s, 2H), 3.39 (q, J = 7.0 Hz, 2H), 1.05 (t, J = 7.0 Hz, 3H).
^{13}C NMR (101 MHz, DMSO-d_6) δ 169.0, 158.7, 143.2, 142.4, 133.6, 133.4, 131.0, 130.5, 129.2, 128.8, 128.6, 128.0, 127.5, 126.9, 126.3, 125.6, 123.2, 119.9, 68.8, 66.2, 51.2, 14.9.
LC/MS (ES-API) m/z = 494 [M+H]$^+$.
Anal. calcd. for $C_{24}H_{23}N_5O_5S$: C 58.41; H 4.70; N 14.19; S 6.50. Found: C 58.63; H 4.85; N 13.89; S 6.33.

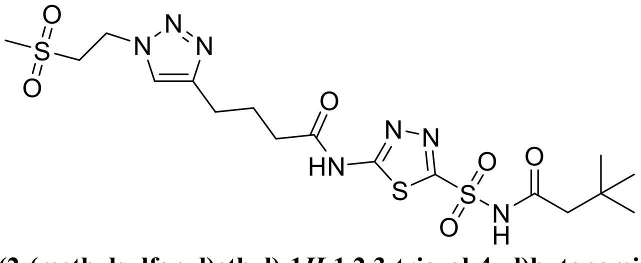

3,3-Dimethyl-*N*-((5-(4-(1-(2-(methylsulfonyl)ethyl)-1*H*-1,2,3-triazol-4-yl)butanamido)-1,3,4-thiadiazol-2-yl)-sulfonyl)butanamide (4{45,5,81}). Yield 88.2 mg (88%); yellowish viscous oil.
^{1}H NMR (500 MHz, DMSO-d_6) δ 13.16 (s, 1H), 12.87 (br s, 1H), 7.98 (s, 1H), 4.75 (t, J = 7.0 Hz, 2H), 3.78 (t, J = 7.0 Hz, 2H), 2.94 (s, 3H), 2.68 (t, J = 7.5 Hz, 2H), 2.61 (t, J = 7.5 Hz, 2H), 2.16 (s, 2H), 1.95 (p, J = 7.5 Hz, 2H), 0.91 (s, 9H).
^{13}C NMR (126 MHz, DMSO-d_6) δ 172.2, 171.1, 162.3, 160.6, 146.2, 122.5, 53.0, 48.0, 43.0, 40.7, 34.1, 30.9, 29.2, 24.2, 24.0.
LC/MS (ES-API) m/z = 522 [M+H]$^+$.
Anal. calcd. for $C_{17}H_{27}N_7O_6S_3$: C 39.14; H 5.22; N 18.80; S 18.44. Found: C 38.99; H 5.40; N 18.99; S 18.19.

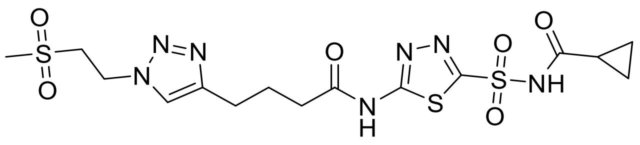

***N*-((5-(4-(1-(2-(Methylsulfonyl)ethyl)-1*H*-1,2,3-triazol-4-yl)butanamido)-1,3,4-thiadiazol-2-yl)sulfonyl)-cyclopropanecarboxamide (4{45,5,2}).** Yield 85.7 mg (86%); yellowish viscous oil.
^{1}H NMR (500 MHz, DMSO-d_6) δ 13.55 – 12.96 (m, 2H), 7.97 (s, 1H), 4.75 (t, J = 7.0 Hz, 2H), 3.78 (t, J = 7.0 Hz, 2H), 2.94 (s, 3H), 2.67 (t, J = 7.5 Hz, 2H), 2.60 (t, J = 7.5 Hz, 2H), 1.94 (p, J = 7.5 Hz, 2H), 1.81 – 1.70 (m, 1H), 0.96 – 0.87 (m, 2H), 0.84 – 0.71 (m, 2H).
^{13}C NMR (151 MHz, DMSO-d_6) δ 173.1, 172.2, 162.4, 160.5, 146.2, 122.6, 53.0, 43.0, 40.7, 34.1, 24.3, 24.1, 14.1, 9.2.
LC/MS (ES-API) m/z = 492 [M+H]$^+$.
Anal. calcd. for $C_{15}H_{21}N_7O_6S_3$: C 36.65; H 4.31; N 19.95; S 19.57. Found: C 36.95; H 4.58; N 20.35; S 19.31.

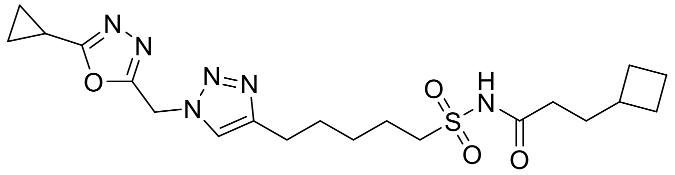

3-Cyclobutyl-*N*-((5-(1-((5-cyclopropyl-1,3,4-oxadiazol-2-yl)methyl)-1*H*-1,2,3-triazol-4-yl)pentyl)sulfonyl)-propanamide (4{51,4,75}). Yield 82.0 mg (82%); colorless oil.
^{1}H NMR (500 MHz, DMSO-d_6) δ 11.54 (br s, 1H), 7.98 (s, 1H), 5.87 (s, 2H), 3.37 – 3.33 (m, 2H), 2.62 (t, J = 7.6 Hz, 2H), 2.27 – 2.12 (m, 4H), 2.01 – 1.91 (m, 2H), 1.85 – 1.50 (m, 10H), 1.45 – 1.37 (m, 2H), 1.17 – 1.09 (m, 2H), 1.01 – 0.92 (m, 2H).
^{13}C NMR (151 MHz, DMSO-d_6) δ 172.6, 169.0, 160.5, 147.1, 122.7, 51.7, 43.4, 34.7, 33.3, 31.1, 28.2, 27.3, 26.8, 24.6, 22.6, 17.7, 8.1, 5.6.
LC/MS (ES-API) *m/z* = 451 [M+H]$^+$.
Anal. calcd. for $C_{20}H_{30}N_6O_4S$: C 53.32; H 6.71; N 18.65; S 7.12. Found: C 53.28; H 6.51; N 18.75; S 7.28.

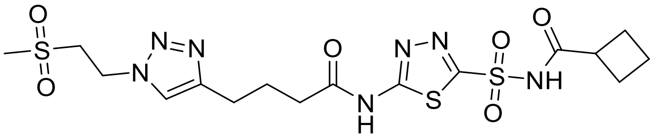

***N*-((5-(4-(1-(2-(Methylsulfonyl)ethyl)-1*H*-1,2,3-triazol-4-yl)butanamido)-1,3,4-thiadiazol-2-yl)sulfonyl)-cyclobutanecarboxamide (4{45,5,78}).** Yield 82.7 mg (83%); colorless oil.
^{1}H NMR (500 MHz, DMSO-d_6) δ 13.17 (s, 1H), 12.81 (br s, 1H), 7.98 (s, 1H), 4.75 (t, J = 7.0 Hz, 2H), 3.78 (t, J = 7.0 Hz, 2H), 3.18 (p, J = 8.4 Hz, 1H), 2.94 (s, 3H), 2.68 (t, J = 7.5 Hz, 2H), 2.61 (t, J = 7.4 Hz, 2H), 2.12 – 2.04 (m, 4H), 2.00 – 1.84 (m, 3H), 1.77 – 1.68 (m, 1H).
^{13}C NMR (151 MHz, DMSO-d_6) δ 173.8, 172.2, 162.3, 160.5, 146.2, 122.6, 53.0, 43.0, 40.7, 38.7, 34.1, 24.3, 24.1, 23.9, 17.4.
LC/MS (ES-API) *m/z* = 506 [M+H]$^+$.
Anal. calcd. for $C_{16}H_{23}N_7O_6S_3$: C 38.01; H 4.59; N 19.39; S 19.02. Found: C 37.93; H 4.99; N 19.77; S 18.99.

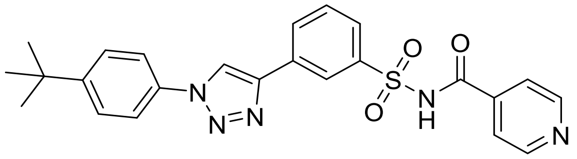

***N*-((3-(1-(4-(*tert*-Butyl)phenyl)-1*H*-1,2,3-triazol-4-yl)phenyl)sulfonyl)isonicotinamide (4{19,6,50}).**
Yield 80.0 mg (80%); yellowish viscous oil.
^{1}H NMR (500 MHz, DMSO-d_6) δ 9.45 (s, 1H), 8.85 – 8.74 (m, 2H), 8.56 (s, 1H), 8.20 (d, J = 7.8 Hz, 1H), 7.98 (d, J = 7.8 Hz, 1H), 7.93 – 7.84 (m, 4H), 7.74 (t, J = 7.8 Hz, 1H), 7.65 (d, J = 8.6 Hz, 2H), 3.61 (br s, 1H), 1.34 (s, 9H).
^{13}C NMR (151 MHz, DMSO-d_6) δ 164.6, 151.5, 148.7, 145.8, 141.4, 141.2, 134.2, 131.0, 129.7, 129.7, 127.1, 126.6, 124.0, 122.6, 120.4, 119.8, 34.5, 31.0.
LC/MS (ES-API) *m/z* = 462 [M+H]$^+$.
Anal. calcd. for $C_{24}H_{23}N_5O_3S$: C 62.46; H 5.02; N 15.17; S 6.95. Found: C 62.30; H 4.91; N 15.03; S 6.65.

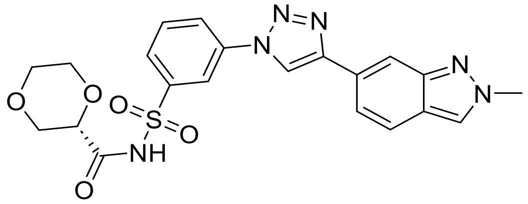

(*S*)-*N*-((3-(4-(2-Methyl-2*H*-indazol-6-yl)-1*H*-1,2,3-triazol-1-yl)phenyl)sulfonyl)-1,4-dioxane-2-carboxamide (7{2,20,59}). Yield 73.4 mg (73%); colorless glass solid.
^{1}H NMR (500 MHz, DMSO-d_6) δ 12.46 (br s, 1H), 9.53 (s, 1H), 8.52 (s, 1H), 8.38 (s, 1H), 8.32 (d, J = 8.0 Hz, 1H), 8.20 (s, 1H), 8.05 (d, J = 8.0 Hz, 1H), 7.93 (t, J = 8.0 Hz, 1H), 7.83 (d, J = 8.6 Hz, 1H), 7.68 (d, J = 8.6 Hz, 1H), 4.20 (s, 3H), 4.16 (dd, J = 8.1, 3.1 Hz, 1H), 3.83 – 3.74 (m, 2H), 3.64 – 3.54 (m, 2H), 3.54 – 3.43 (m, 2H).
^{13}C NMR (151 MHz, DMSO-d_6) δ 168.1, 148.4, 148.2, 140.9, 136.8, 131.2, 127.3, 126.9, 124.9, 124.8, 121.5, 121.3, 119.9, 119.2, 118.7, 113.0, 73.8, 66.3, 65.5, 65.1, 40.1.
LC/MS (ES-API) *m/z* = 469 [M+H]$^+$.
Anal. calcd. for $C_{21}H_{20}N_6O_5S$: C 53.84; H 4.30; N 17.94; S 6.84. Found: C 54.02; H 4.36; N 18.19; S 6.97.

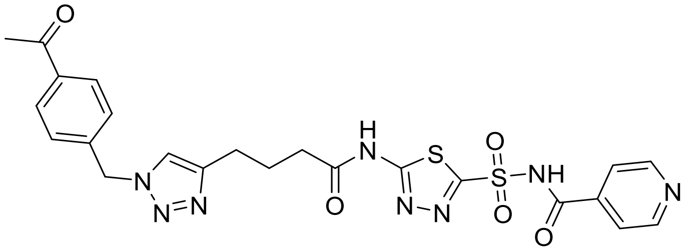

***N*-((5-(4-(1-(4-Acetylbenzyl)-1*H*-1,2,3-triazol-4-yl)butanamido)-1,3,4-thiadiazol-2-yl)sulfonyl)-isonicotinamide (4{43,5,50}).** Yield 80.6 mg (81%); amber glass solid.
^{1}H NMR (500 MHz, DMSO-d_6) δ 12.70 (br s, 2H), 8.91 (d, J = 5.8 Hz, 2H), 8.26 (d, J = 5.8 Hz, 2H), 7.98 (s, 1H), 7.94 (d, J = 8.1 Hz, 2H), 7.38 (d, J = 8.1 Hz, 2H), 5.64 (s, 2H), 2.67 (t, J = 7.5 Hz, 2H), 2.58 – 2.53 (m, 5H), 1.94 (p, J = 7.5 Hz, 2H).
^{13}C NMR (101 MHz, DMSO-d_6) δ 197.5, 171.6, 166.2, 165.9, 160.6, 151.9, 146.5, 143.7, 141.3, 136.4, 128.7, 127.9, 125.0, 122.5, 52.2, 34.2, 26.8, 24.4, 24.2.
LC/MS (ES-API) *m/z* = 555 [M+H]$^+$.
Anal. calcd. for $C_{23}H_{22}N_8O_5S_2$: C 49.81; H 4.00; N 20.20; S 11.56. Found: C 49.59; H 3.84; N 19.88; S 11.45.

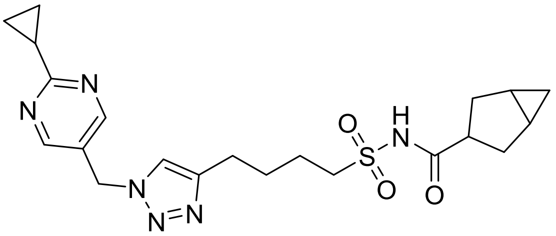

***N*-((4-(1-((2-Cyclopropylpyrimidin-5-yl)methyl)-1*H*-1,2,3-triazol-4-yl)butyl)sulfonyl)bicyclo[3.1.0]hexane-3-carboxamide (4{64,3,61}).** Yield 95.8 mg (96%); colorless glass solid.
^{1}H NMR (500 MHz, DMSO-d_6) δ 11.47 (br s, 1H), 8.62 (s, 2H), 7.95 (s, 1H), 5.55 (s, 2H), 3.40 – 3.33 (m, 2H), 2.61 (t, J = 6.9 Hz, 2H), 2.41 – 2.32 (m, 1H), 2.22 – 2.14 (m, 1H), 1.89 (dd, J = 12.2, 7.7 Hz, 2H), 1.83 – 1.74 (m, 2H), 1.73 – 1.58 (m, 4H), 1.34 – 1.19 (m, 2H), 1.10 – 0.94 (m, 4H), 0.36 – 0.26 (m, 1H), 0.05 – -0.03 (m, 1H).
^{13}C NMR (101 MHz, DMSO-d_6) δ 174.1, 170.9, 156.8, 146.7, 126.4, 122.1, 51.4, 47.8, 30.9, 27.1, 24.4, 22.5, 17.7, 15.7, 10.6, 9.8, 6.3.
LC/MS (ES-API) *m/z* = 445 [M+H]$^+$.
Anal. calcd. for $C_{21}H_{28}N_6O_3S$: C 56.74; H 6.35; N 18.90; S 7.21. Found: C 56.77; H 6.16; N 19.17; S 7.53.

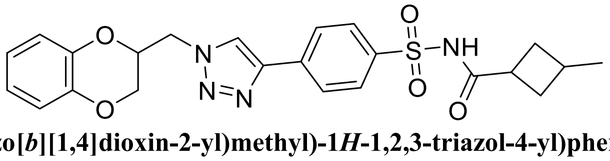

***N*-((4-(1-((2,3-Dihydrobenzo[*b*][1,4]dioxin-2-yl)methyl)-1*H*-1,2,3-triazol-4-yl)phenyl)sulfonyl)-3-methylcyclobutane-1-carboxamide (4{17,7,31}).** Yield 98.9 mg (99%); yellowish oil.
^{1}H NMR (500 MHz, DMSO-d_6) δ 11.97 (br s, 1H), 8.79 (s, 1H), 8.11 (d, J = 8.2 Hz, 2H), 7.98 (dd, J = 8.5, 3.5 Hz, 2H), 6.95 – 6.79 (m, 4H), 4.90 – 4.82 (m, 1H), 4.79 – 4.69 (m, 2H), 4.44 (d, J = 10.5 Hz, 1H), 4.04 (dd, J = 11.6, 6.0 Hz, 1H), 3.10 – 2.87 (m, 1H), 2.28 – 2.06 (m, 3H), 1.73 – 1.49 (m, 2H), 1.02 (d, J = 6.6 Hz, 1.4H), 0.91 (d, J = 6.6 Hz, 1.6H).
^{13}C NMR (151 MHz, DMSO-d_6) δ 173.7, 172.9, 144.9, 142.7, 142.0, 138.3, 138.3, 135.4, 135.4, 128.3, 125.5, 123.9, 121.7, 121.6, 117.3, 117.0, 71.2, 64.6, 49.7, 35.2, 34.9, 31.8, 30.7, 25.8, 25.6, 21.5, 21.4.
LC/MS (ES-API) *m/z* = 469 [M+H]$^+$.
Anal. calcd. for $C_{23}H_{24}N_4O_5S$: C 58.96; H 5.16; N 11.96; S 6.84. Found: C 59.29; H 4.89; N 12.03; S 6.68.

Parallel synthesis details

Table S4. SMILES and yields of products **4** and **7** along with the corresponding reactants.

	Library 4							
**#**	**Product SMILES**	**Reactant 1 SMILES**	**Reactant 2 SMILES**	**Reactant 3 SMILES**	**Product ID**	**LC/MS *m/z***	**Yield, mg**	**Yield, %**
1	CCOCC(=O)NS(=O)(=O)C=1C=CC(=CC1)NC(=O)C2=CN(CC=3C=CC(OC)=C(C3)OC)N=N2	COC1=CC=C(C=C1OC)CN=[N+]=[N-]	C#CC(=O)NC=1C=CC(=CC1)S(N)(=O)=O	CCOCC(=O)O	**4**{*1,8,1*}	504 [M+H]$^+$	99.8	100
2	O=C(O)C(F)(F)F.CC=1C=C(C=CN1)C(=O)NS(=O)(=O)C=2C=CC(=CC2)C3=CN(CCN(C)CC=4C=CC=C(F)C4)N=N3	CN(CCN=[N+]=[N-])CC1=CC=CC(F)=C1	C#CC=1C=CC(=CC1)S(N)(=O)=O	CC=1C=C(C=CN1)C(=O)O	**4**{*16,7,28*}	509 [M–TFA+H]$^+$	99.7	100
3	CCC1=NC(=CS1)CN2C=C(N=N2)C=3C=CC=C(C3)S(=O)(=O)NC(=O)C=4C=CC=NC4	CCC1=NC(=CS1)CN=[N+]=[N-]	C#CC=1C=CC=C(C1)S(N)(=O)=O	O=C(O)C=1C=CC=NC1	**4**{*32,6,17*}	455 [M+H]$^+$	99.7	100
4	CCCC(=O)NS(=O)(=O)C=1C=CC=C(C1)C2=CN(CC3(CC#N)CC3)N=N2	[N-]=[N+]=NCC1(CC#N)CC1	C#CC=1C=CC=C(C1)S(N)(=O)=O	CCCC(=O)O	**4**{*33,6,4*}	388 [M+H]$^+$	99.5	100
5	O=C(NS(=O)(=O)C=1C=CC=C(C1)C2=CN(CC=3C=CC(=CC3)N4C=CC=N4)N=N2)C=5C=CC=NC5	[N-]=[N+]=NCC=1C=CC(=CC1)N2C=CC=N2	C#CC=1C=CC=C(C1)S(N)(=O)=O	O=C(O)C=1C=CC=NC1	**4**{*18,6,17*}	486 [M+H]$^+$	99.5	100
6	COC=1C=CC(=CC1OC)CN2C=C(CCCC(=O)NC3=NN=C(S3)S(=O)(=O)NC(=O)C4CC4)N=N2	COC1=CC=C(C=C1OC)CN=[N+]=[N-]	C#CCCCC(=O)NC1=NN=C(S1)S(N)(=O)=O	O=C(O)C1CC1	**4**{*1,5,2*}	536 [M+H]$^+$	99.4	99
7	CC1=CC=C(CN2C=C(CCCCS(=O)(=O)NC(=O)C3CC43CCC4)N=N2)O1	CC1=CC=C(CN=[N+]=[N-])O1	C#CCCCCS(N)(=O)=O	O=C(O)C1CC21CCC2	**4**{*59,3,29*}	407 [M+H]$^+$	99.2	99
8	CC1CC(C1)C(=O)NS(=O)(=O)C=2C=CC(=CC2)C3=CN(CC4COC=5C=CC=CC5O4)N=N3	[N-]=[N+]=NCC1COC=2C=CC=CC2O1	C#CC=1C=CC(=CC1)S(N)(=O)=O	CC1CC(C1)C(=O)O	**4**{*17,7,31*}	469 [M+H]$^+$	98.9	99
9	CCC1(C)CC1C(=O)NS(=O)(=O)CCCC2=CN(CCC3=C(C=NN3C)C(=O)OC)N=N2	COC(=O)C=1C=NN(C)C1CCN=[N+]=[N-]	C#CCCCS(N)(=O)=O	CCC1(C)CC1C(=O)O	**4**{*71,2,70*}	467 [M+H]$^+$	98.8	99
10	O=C(NS(=O)(=O)CCCCC1=CN(CC2C3CC=CCC23)N=N1)C4CC54CC5	[N-]=[N+]=NCC1C2CC=CCC12	C#CCCCCS(N)(=O)=O	O=C(O)C1CC21CC2	**4**{*62,3,21*}	405 [M+H]$^+$	98.2	98
11	CC12CC(C1)(C2)C(=O)NS(=O)(=O)CCCCC3=CN(CCC(F)(F)C=4C=CN=CC4)N=N3	[N-]=[N+]=NCCC(F)(F)C=1C=CN=CC1	C#CCCCCS(N)(=O)=O	CC12CC(C1)(C2)C(=O)O	**4**{*63,3,64*}	468 [M+H]$^+$	97.3	97
12	O=C(NS(=O)(=O)C=1C=CC(=CC1)C2=CN(CC=3C=CC(=CC3)N4C=CC=N4)N=N2)C=5C=CC=NC5	[N-]=[N+]=NCC=1C=CC(=CC1)N2C=CC=N2	C#CC=1C=CC(=CC1)S(N)(=O)=O	O=C(O)C=1C=CC=NC1	**4**{*18,7,17*}	486 [M+H]$^+$	97.2	97
13	CC=1C=NC=C(N1)C(=O)NS(=O)(=O)C2=NN=C(NC(=O)CCCC3=CN(CCS(C)(=O)=O)N=N3)S2	CS(=O)(=O)CCN=[N+]=[N-]	C#CCCCC(=O)NC1=NN=C(S1)S(N)(=O)=O	CC=1C=NC=C(N1)C(=O)O	**4**{*45,5,30*}	542 [M–H]$^-$	96.6	97
14	CC1=CC=C(O1)C(=O)NS(=O)(=O)C=2C=CC=C(C2)C3=CN(CC4(CC#N)CC4)N=N3	[N-]=[N+]=NCC1(CC#N)CC1	C#CC=1C=CC=C(C1)S(N)(=O)=O	CC1=CC=C(O1)C(=O)O	**4**{*33,6,43*}	426 [M+H]$^+$	96.4	96
15	CC(C)(C)C=1C=CC(=CC1)N2C=C(N=N2)C=3C=CC(=CC3)S(=O)(=O)NC(=O)C=4C=CSC4	CC(C)(C)C=1C=CC(=CC1)N=[N+]=[N-]	C#CC=1C=CC(=CC1)S(N)(=O)=O	O=C(O)C=1C=CSC1	**4**{*19,7,32*}	467 [M+H]$^+$	96.2	96
16	O=C(O)C(F)(F)F.CC1=CC(=CO1)C(=O)NS(=O)(=O)CCCC2=CN(N=N2)[C@H](C)C=3C=CC(=CC3)N4C=CN=C4	C[C@@H](N=[N+]=[N-])C=1C=CC(=CC1)N2C=CN=C2	C#CCCCS(N)(=O)=O	CC1=CC(=CO1)C(=O)O	**4**{*72,2,66*}	469 [M–TFA+H]$^+$	95.9	96
17	O=C(O)C(F)(F)F.COC=1C(C)=CN=C(CN2C=C(CCCC(=O)NC3=NN=C(S3)S(=O)(=O)NC(=O)C4CCC4)N=N2)C1C	COC1=C(C)C=NC(CN=[N+]=[N-])=C1C	C#CCCCC(=O)NC1=NN=C(S1)S(N)(=O)=O	O=C(O)C1CCC1	**4**{*46,5,78*}	549 [M–TFA+H]$^+$	95.8	96
18	O=C(NS(=O)(=O)CCCCC1=CN(CC=2C=NC(=NC2)C3CC3)N=N1)C4CC5CC5C4	[N-]=[N+]=NCC=1C=NC(=NC1)C2CC2	C#CCCCCS(N)(=O)=O	O=C(O)C1CC2CC2C1	**4**{*64,3,61*}	445 [M+H]$^+$	95.8	96
19	O=C(O)C(F)(F)F.CN(CCN1C=C(N=N1)C=2C=CC(=CC2)S(=O)(=O)NC(=O)C=3C=CC=CN3)CC=4C=CC=C(F)C4	CN(CCN=[N+]=[N-])CC1=CC=CC(F)=C1	C#CC=1C=CC(=CC1)S(N)(=O)=O	O=C(O)C=1C=CC=CN1	**4**{*16,7,18*}	495 [M–TFA+H]$^+$	95.2	95
20	CN1C=C(C=N1)CN2C=C(N=N2)C=3C=CC=C(C3)S(=O)(=O)NC(=O)C=4C=NN(C)C4	CN1C=C(C=N1)CN=[N+]=[N-]	C#CC=1C=CC=C(C1)S(N)(=O)=O	CN1C=C(C=N1)C(=O)O	**4**{*34,6,47*}	427 [M+H]$^+$	94.7	95
21	O=C(O)C(F)(F)F.COC=1N=CC=NC1C(=O)NS(=O)(=O)C2=NN=C(NC(=O)CCCC3=CN(CC=4N=CC(C)=C(OC)C4C)N=N3)S2	COC1=C(C)C=NC(CN=[N+]=[N-])=C1C	C#CCCCC(=O)NC1=NN=C(S1)S(N)(=O)=O	COC=1N=CC=NC1C(=O)O	**4**{*46,5,79*}	603 [M–TFA+H]$^+$	94.4	94
22	CC(C)(C)C=1C=CC=C(C1)N2C=C(N=N2)C=3C=CC=C(C3)S(=O)(=O)NC(=O)C=4C=COC4	CC(C)(C)C1=CC=CC(=C1)N=[N+]=[N-]	C#CC=1C=CC=C(C1)S(N)(=O)=O	O=C(O)C=1C=COC1	**4**{*10,6,48*}	451 [M+H]$^+$	92.7	93
23	CC(=O)C=1C=CC=C(C1)CN2C=C(CCCC(=O)NC3=NN=C(S3)S(=O)(=O)NC(=O)C4=CSC=N4)N=N2	CC(=O)C1=CC=CC(=C1)CN=[N+]=[N-]	C#CCCCC(=O)NC1=NN=C(S1)S(N)(=O)=O	O=C(O)C1=CSC=N1	**4**{*11,5,80*}	561 [M+H]$^+$	90.6	91
24	O=C(O)C(F)(F)F.COCC(=O)NS(=O)(=O)C=1C=CC(=CC1)C2=CN(CCN(C)CC=3C=CC=C(F)C3)N=N2	CN(CCN=[N+]=[N-])CC1=CC=CC(F)=C1	C#CC=1C=CC(=CC1)S(N)(=O)=O	COCC(=O)O	**4**{*16,7,19*}	462 [M–TFA+H]$^+$	88.8	89
25	CC(C)(C)CC(=O)NS(=O)(=O)C1=NN=C(NC(=O)CCCC2=CN(CCS(C)(=O)=O)N=N2)S1	CS(=O)(=O)CCN=[N+]=[N-]	C#CCCCC(=O)NC1=NN=C(S1)S(N)(=O)=O	CC(C)(C)CC(=O)O	**4**{*45,5,81*}	522 [M+H]$^+$	88.2	88
26	CC=1C=C(CN2C=C(N=N2)C=3C=CC(=CC3)S(=O)(=O)NC(=O)CC(C)C)ON1	CC=1C=C(CN=[N+]=[N-])ON1	C#CC=1C=CC(=CC1)S(N)(=O)=O	CC(C)CC(=O)O	**4**{*20,7,16*}	404 [M+H]$^+$	88	88
27	O=C(NS(=O)(=O)C=1C=CC(=CC1)C2=CN(CC=3C=CC(=CC3)N4C=CC=N4)N=N2)C=5C=CSC5	[N-]=[N+]=NCC=1C=CC(=CC1)N2C=CC=N2	C#CC=1C=CC(=CC1)S(N)(=O)=O	O=C(O)C=1C=CSC1	**4**{*18,7,32*}	491 [M+H]$^+$	86.4	86
28	CC1=NC(=CO1)C(=O)NS(=O)(=O)C=2C=CC(=CC2)C3=CN(N=N3)C(CC=4C=CC=CC4)C(N)=O	[N-]=[N+]=NC(CC=1C=CC=CC1)C(N)=O	C#CC=1C=CC(=CC1)S(N)(=O)=O	CC1=NC(=CO1)C(=O)O	**4**{*21,7,33*}	481 [M+H]$^+$	86.4	86
29	CN1N=CC=C1C(=O)NS(=O)(=O)C=2C=CC=C(C2)C3=CN(N=N3)C=4C=CC=C(C4)C(C)(C)C	CC(C)(C)C1=CC=CC(=C1)N=[N+]=[N-]	C#CC=1C=CC=C(C1)S(N)(=O)=O	CN1N=CC=C1C(=O)O	**4**{*10,6,49*}	465 [M+H]$^+$	86.1	86
30	CS(=O)(=O)CCN1C=C(CCCC(=O)NC2=NN=C(S2)S(=O)(=O)NC(=O)C3CC3)N=N1	CS(=O)(=O)CCN=[N+]=[N-]	C#CCCCC(=O)NC1=NN=C(S1)S(N)(=O)=O	O=C(O)C1CC1	**4**{*45,5,2*}	492 [M+H]$^+$	85.7	86
31	CC1CC(C1)C(=O)NS(=O)(=O)CCCCCC2=CN(CC3COC=4C=CC=CC4O3)N=N2	[N-]=[N+]=NCC1COC=2C=CC=CC2O1	C#CCCCCCS(N)(=O)=O	CC1CC(C1)C(=O)O	**4**{*17,4,31*}	463 [M+H]$^+$	85.7	86
32	CCOCC(=O)NS(=O)(=O)C=1C=CC(=CC1)NC(=O)C2=CN(CC=3C=CC=C4C=CC=CC34)N=N2	[N-]=[N+]=NCC1=CC=CC2=CC=CC=C12	C#CC(=O)NC=1C=CC(=CC1)S(N)(=O)=O	CCOCC(=O)O	**4**{*2,8,1*}	494 [M+H]$^+$	85.6	86
33	O=C(O)C(F)(F)F.CC(C)CC(=O)NS(=O)(=O)C=1C=CC(=CC1)C2=CN(CCN(C)CC=3C=CC=C(F)C3)N=N2	CN(CCN=[N+]=[N-])CC1=CC=CC(F)=C1	C#CC=1C=CC(=CC1)S(N)(=O)=O	CC(C)CC(=O)O	**4**{*16,7,16*}	474 [M–TFA+H]$^+$	84.7	85
34	CCOC(=O)C1CCC(=CN2C=C(CCCS(=O)(=O)NC(=O)[C@@H]3C[C@H]3C(C)C)N=N2)CC1 |&1:22,24,r|	CCOC(=O)C1CCC(=CN=[N+]=[N-])CC1	C#CCCCS(N)(=O)=O	CC(C)[C@@H]1C[C@H]1C(=O)O |o1:3,5,r|	**4**{*73,2,57*}	467 [M+H]$^+$	83.3	83
35	O=C(CCCC1=CN(N=N1)C=2C(F)=CC(Cl)=CC2F)NC3=NN=C(S3)S(=O)(=O)NC(=O)C4=CC=CS4	[N-]=[N+]=NC=1C(F)=CC(Cl)=CC1F	C#CCCCC(=O)NC1=NN=C(S1)S(N)(=O)=O	O=C(O)C1=CC=CS1	**4**{*42,5,53*}	574/576 as 3/1 [M+H]$^+$	83.2	83
36	COC(=O)C=1C=C(F)C=C(C1)CN2C=C(CCCCCS(=O)(=O)NC(=O)CC3(C)CC3)N=N2	COC(=O)C1=CC(F)=CC(=C1)CN=[N+]=[N-]	C#CCCCCCS(N)(=O)=O	CC1(CC(=O)O)CC1	**4**{*50,4,71*}	481 [M+H]$^+$	82.8	83
37	CS(=O)(=O)CCN1C=C(CCCC(=O)NC2=NN=C(S2)S(=O)(=O)NC(=O)C3CCC3)N=N1	CS(=O)(=O)CCN=[N+]=[N-]	C#CCCCC(=O)NC1=NN=C(S1)S(N)(=O)=O	O=C(O)C1CCC1	**4**{*45,5,78*}	506 [M+H]$^+$	82.7	83
38	O=C(CCC1CCC1)NS(=O)(=O)CCCCCC2=CN(CC3=NN=C(O3)C4CC4)N=N2	[N-]=[N+]=NCC1=NN=C(O1)C2CC2	C#CCCCCCS(N)(=O)=O	O=C(O)CCC1CCC1	**4**{*51,4,75*}	451 [M+H]$^+$	82	82
39	COC(=O)C=1C=CC=C(CN2C=C(CCCCS(=O)(=O)NC(=O)C3CC4CC4C3)N=N2)C1F	COC(=O)C1=CC=CC(CN=[N+]=[N-])=C1F	C#CCCCCS(N)(=O)=O	O=C(O)C1CC2CC2C1	**4**{*65,3,61*}	479 [M+H]$^+$	81.9	82
40	CC(=O)C=1C=CC(=CC1)CN2C=C(CCCC(=O)NC3=NN=C(S3)S(=O)(=O)NC(=O)C=4C=CN=CC4)N=N2	CC(=O)C=1C=CC(=CC1)CN=[N+]=[N-]	C#CCCCC(=O)NC1=NN=C(S1)S(N)(=O)=O	O=C(O)C=1C=CN=CC1	**4**{*43,5,50*}	555 [M+H]$^+$	80.6	81
41	CC=1C=C(C=CN1)C(=O)NS(=O)(=O)C=2C=CC(=CC2)C3=CN(N=N3)C4CCC(C#N)C4	[N-]=[N+]=NC1CCC(C#N)C1	C#CC=1C=CC(=CC1)S(N)(=O)=O	CC=1C=C(C=CN1)C(=O)O	**4**{*22,7,28*}	437 [M+H]$^+$	80.4	80
42	CN1C=C(C=N1)C(=O)NS(=O)(=O)CCCCCC2=CN(N=N2)C=3C=CC(F)=CC3	[N-]=[N+]=NC=1C=CC(F)=CC1	C#CCCCCCS(N)(=O)=O	CN1C=C(C=N1)C(=O)O	**4**{*44,4,47*}	421 [M+H]$^+$	80.2	80
43	CC(C)(C)C=1C=CC(=CC1)N2C=C(N=N2)C=3C=CC=C(C3)S(=O)(=O)NC(=O)C=4C=CN=CC4	CC(C)(C)C=1C=CC(=CC1)N=[N+]=[N-]	C#CC=1C=CC=C(C1)S(N)(=O)=O	O=C(O)C=1C=CN=CC1	**4**{*19,6,50*}	462 [M+H]$^+$	80	80
44	O=C(NC=1C=CC(=CC1)S(=O)(=O)NC(=O)C2CC2)C3=CN(N=N3)C=4C=CC=CC4F	[N-]=[N+]=NC=1C=CC=CC1F	C#CC(=O)NC=1C=CC(=CC1)S(N)(=O)=O	O=C(O)C1CC1	**4**{*3,8,2*}	430 [M+H]$^+$	79.6	80
45	COC1=CC(=CC(OC)=C1OC)N2C=C(N=N2)C=3C=CC=C(C3)S(=O)(=O)NC(=O)C=4C=CC=C(F)C4	COC1=CC(=CC(OC)=C1OC)N=[N+]=[N-]	C#CC=1C=CC=C(C1)S(N)(=O)=O	O=C(O)C=1C=CC=C(F)C1	**4**{*35,6,41*}	513 [M+H]$^+$	79.5	80
46	CCC1(C)CC1C(=O)NS(=O)(=O)CCCC2=CN(CC3=CN=C(C)C=N3)N=N2	CC=1C=NC(=CN1)CN=[N+]=[N-]	C#CCCCS(N)(=O)=O	CCC1(C)CC1C(=O)O	**4**{*74,2,70*}	407 [M+H]$^+$	79.2	79
47	CC1=CC=C(CN2C=C(CCS(=O)(=O)NC(=O)C3CC(C)(F)C3)N=N2)O1	CC1=CC=C(CN=[N+]=[N-])O1	C#CCCS(N)(=O)=O	CC1(F)CC(C1)C(=O)O	**4**{*59,1,46*}	385 [M+H]$^+$	79.2	79
48	CC1CC1CC(=O)NS(=O)(=O)CCC2=CN(CC3=CN=CC(=C3)C(C)(F)F)N=N2	CC(F)(F)C1=CN=CC(=C1)CN=[N+]=[N-]	C#CCCS(N)(=O)=O	CC1CC1CC(=O)O	**4**{*83,1,93*}	428 [M+H]$^+$	78.3	78

49	CC=1C=NC=C(N1)C(=O)NS(=O)(=O)C2=NN=C(NC(=O)CCCC3=CN(N=N3)C=4C=CC(Br)=CC4)S2	[N-]=[N+]=NC=1C=CC(Br)=CC1	C#CCCCC(=O)NC1=NN=C(S1)S(N)(=O)=O	CC=1C=NC=C(N1)C(=O)O	**4**{*47,5,30*}	592/594 as 1/1 [M+H]$^{+}$	77.7	78
50	COCC(=O)NS(=O)(=O)C=1C=CC(=CC1)C2=CN(CC3=CC=C(Cl)S3)N=N2	[N-]=[N+]=NCC1=CC=C(Cl)S1	C#CC=1C=CC(=CC1)S(N)(=O)=O	COCC(=O)O	**4**{*23,7,19*}	527/529 as 3/1 [M+H]$^{+}$	77.4	77
51	N#CCC1(CN2C=C(N=N2)C=3C=CC=C(C3)S(=O)(=O)NC(=O)C4=CN=CC=N4)CC1	[N-]=[N+]=NCC1(CC#N)CC1	C#CC=1C=CC=C(C1)S(N)(=O)=O	O=C(O)C=1C=NC=CN1	**4**{*33,6,51*}	424 [M+H]$^{+}$	77.1	77
52	CC=1C=CC=NC1C(=O)NS(=O)(=O)C=2C=CC=C(C2)C3=CN(N=N3)C=4C=CC(Cl)=C(Cl)C4	[N-]=[N+]=NC=1C=CC(Cl)=C(Cl)C1	C#CC=1C=CC=C(C1)S(N)(=O)=O	CC=1C=CC=NC1C(=O)O	**4**{*14,6,10*}	488/490/492 as 9/6/1 [M+H]$^{+}$	75.9	76
53	COC1=CC(=CC(OC)=C1OC)N2C=C(N=N2)C=3C=CC=C(C3)S(=O)(=O)NC(=O)C4=COC=N4	COC1=CC(=CC(OC)=C1OC)N=[N+]=[N-]	C#CC=1C=CC=C(C1)S(N)(=O)=O	O=C(O)C1=COC=N1	**4**{*35,6,40*}	486 [M+H]$^{+}$	75.9	76
54	COC=1C=CC(CN2C=C(CCS(=O)(=O)NC(=O)CC3CCN(C)C3=O)N=N2)=NN1	COC=1C=CC(CN=[N+]=[N-])=NN1	C#CCCS(N)(=O)=O	CN1CCC(CC(=O)O)C1=O	**4**{*76,1,24*}	438 [M+H]$^{+}$	75.6	76
55	O=C(NS(=O)(=O)CCC1=CN(CC2COC=3C=CC=CC3O2)N=N1)C=4C=CSN4	[N-]=[N+]=NCC1COC=2C=CC=CC2O1	C#CCCS(N)(=O)=O	O=C(O)C=1C=CSN1	**4**{*17,1,72*}	436 [M+H]$^{+}$	75.6	76
56	CC=1C=CC=CC1C(=O)NS(=O)(=O)C2=NN=C(NC(=O)CCCC3=CN(CC4(CC#N)CC4)N=N3)S2	[N-]=[N+]=NCC1(CC#N)CC1	C#CCCCC(=O)NC1=NN=C(S1)S(N)(=O)=O	CC=1C=CC=CC1C(=O)O	**4**{*33,5,52*}	529 [M+H]$^{+}$	74.8	75
57	CC(F)(F)CCC(=O)NS(=O)(=O)CCCCC1=CN(CC2C3CC=CCC23)N=N1	[N-]=[N+]=NCC1C2CC=CCC12	C#CCCCCS(N)(=O)=O	CC(F)(F)CCC(=O)O	**4**{*62,3,73*}	431 [M+H]$^{+}$	74.3	74
58	CN1C=CC=C1C(=O)NS(=O)(=O)C2=NN=C(NC(=O)CCCC3=CN(N=N3)C=4C=CC=CC4)S2	[N-]=[N+]=NC=1C=CC=CC1	C#CCCCC(=O)NC1=NN=C(S1)S(N)(=O)=O	CN1C=CC=C1C(=O)O	**4**{*48,5,82*}	501 [M+H]$^{+}$	73.9	74
59	CC=1C=CC=C(C1)C(=O)NS(=O)(=O)C=2C=CC(=CC2)NC(=O)C3=CN(CC=4C=CC=C5C=CC=CC45)N=N3	[N-]=[N+]=NCC1=CC=CC2=CC=CC=C12	C#CC(=O)NC=1C=CC(=CC1)S(N)(=O)=O	CC=1C=CC=C(C1)C(=O)O	**4**{*2,8,3*}	526 [M+H]$^{+}$	73.1	73
60	CN1C=C(C=N1)CN2C=C(CCCCCS(=O)(=O)NC(=O)C=3C=NN(C)C3)N=N2	CN1C=C(C=N1)CN=[N+]=[N-]	C#CCCCCCS(N)(=O)=O	CN1C=C(C=N1)C(=O)O	**4**{*34,4,47*}	421 [M+H]$^{+}$	72.5	73
61	CC(C)(C)C=1C=CC(=CC1)N2C=C(CCCC(=O)NC3=NN=C(S3)S(=O)(=O)NC(=O)C4=CC=NS4)N=N2	CC(C)(C)C=1C=CC(=CC1)N=[N+]=[N-]	C#CCCCC(=O)NC1=NN=C(S1)S(N)(=O)=O	O=C(O)C1=CC=NS1	**4**{*19,5,22*}	561 [M+H]$^{+}$	72.2	72
62	O=C(O)C(F)(F)F.COC=1C(C)=CN=C(CN2C=C(CCCC(=O)NC3=NN=C(S3)S(=O)(=O)NC(=O)CC(C)(C)C)N=N2)C1C	COC1=C(C)C=NC(CN=[N+]=[N-])=C1C	C#CCCCC(=O)NC1=NN=C(S1)S(N)(=O)=O	CC(C)(C)CC(=O)O	**4**{*46,5,81*}	565 [M–TFA+H]$^{+}$	71.4	71
63	O=C(CCCC1=CN(N=N1)C=2C=CC=NC2)NC3=NN=C(S3)S(=O)(=O)NC(=O)C4CC(F)(F)C4	[N-]=[N+]=NC=1C=CC=NC1	C#CCCCC(=O)NC1=NN=C(S1)S(N)(=O)=O	O=C(O)C1CC(F)(F)C1	**4**{*7,5,9*}	513 [M+H]$^{+}$	71.4	71
64	CC1CC1CC(=O)NS(=O)(=O)CCC2=CN(CC=3C=NN(C)C3)N=N2	CN1C=C(C=N1)CN=[N+]=[N-]	C#CCCS(N)(=O)=O	CC1CC1CC(=O)O	**4**{*34,1,93*}	367 [M+H]$^{+}$	70.2	70
65	O=C(CCCC1=CN(N=N1)C=2C=CC=NC2)NC3=NN=C(S3)S(=O)(=O)NC(=O)C4CC4	[N-]=[N+]=NC=1C=CC=NC1	C#CCCCC(=O)NC1=NN=C(S1)S(N)(=O)=O	O=C(O)C1CC1	**4**{*7,5,2*}	463 [M+H]$^{+}$	70.1	70
66	COC(=O)C1CCC2(CC(C2)N3C=C(CCCS(=O)(=O)NC(=O)C4=COC(C)=C4)N=N3)C1	COC(=O)C1CCC2(CC(C2)N=[N+]=[N-])C1	C#CCCCS(N)(=O)=O	CC1=CC(=CO1)C(=O)O	**4**{*75,2,66*}	465 [M+H]$^{+}$	69.3	69
67	CCCC(=O)NS(=O)(=O)C=1C=CC(=CC1)NC(=O)C2=CN(N=N2)C=3C=CC=CN3	[N-]=[N+]=NC=1C=CC=CN1	C#CC(=O)NC=1C=CC(=CC1)S(N)(=O)=O	CCCC(=O)O	**4**{*4,8,4*}	415 [M+H]$^{+}$	68.4	68
68	COC(=O)C=1C=CC=C(CN2C=C(CCCCS(=O)(=O)NC(=O)C3CC43CC4)N=N2)C1F	COC(=O)C1=CC=CC(CN=[N+]=[N-])=C1F	C#CCCCCS(N)(=O)=O	O=C(O)C1CC21CC2	**4**{*65,3,21*}	465 [M+H]$^{+}$	68	68
69	O=C(CCCC1=CN(N=N1)C=2C(F)=CC(Cl)=CC2F)NC3=NN=C(S3)S(=O)(=O)NC(=O)C=4C=COC4	[N-]=[N+]=NC=1C(F)=CC(Cl)=CC1F	C#CCCCC(=O)NC1=NN=C(S1)S(N)(=O)=O	O=C(O)C=1C=COC1	**4**{*42,5,48*}	558/560 as 3/1 [M+H]$^{+}$	67.6	68
70	CC1=CC(C)=C(C)C=C1CN2C=C(N=N2)C(=O)NC=3C=CC(=CC3)S(=O)(=O)NC(=O)C4CCCC4	CC1=CC(C)=C(C=C1C)CN=[N+]=[N-]	C#CC(=O)NC=1C=CC(=CC1)S(N)(=O)=O	O=C(O)C1CCCC1	**4**{*5,8,5*}	496 [M+H]$^{+}$	67.5	68
71	O=C(NS(=O)(=O)C=1C=CC(=CC1)C2=CN(CC3COC=4C=CC=CC4O3)N=N2)C=5C=NSC5	[N-]=[N+]=NCC1COC=2C=CC=CC2O1	C#CC=1C=CC(=CC1)S(N)(=O)=O	O=C(O)C=1C=NSC1	**4**{*17,7,27*}	484 [M+H]$^{+}$	67.1	67
72	O=C(NC=1C=CC(=CC1)S(=O)(=O)NC(=O)C2=CC=CO2)C3=CN(CC=4C=CC=C5C=CC=CC45)N=N3	[N-]=[N+]=NCC1=CC=CC2=CC=CC=C12	C#CC(=O)NC=1C=CC(=CC1)S(N)(=O)=O	O=C(O)C1=CC=CO1	**4**{*2,8,6*}	502 [M+H]$^{+}$	66.6	67
73	O=C(O)C(F)(F)F.CC=1C=CC(=CN1)C(=O)NS(=O)(=O)C=2C=CC=C(C2)C3=CN(CCCN4CCCC4)N=N3	Cl.[N-]=[N+]=NCCCN1CCCC1	C#CC=1C=CC=C(C1)S(N)(=O)=O	CC=1C=CC(=CN1)C(=O)O	**4**{*12,6,13*}	455 [M–TFA+H]$^{+}$	66.6	67
74	CC1(F)CC(C1)C(=O)NS(=O)(=O)CCCCC2=CN(CC=3C=NC(=NC3)C4CC4)N=N2	[N-]=[N+]=NCC=1C=NC(=NC1)C2CC2	C#CCCCCS(N)(=O)=O	CC1(F)CC(C1)C(=O)O	**4**{*64,3,46*}	451 [M+H]$^{+}$	66.6	67
75	CC1=NN=C(CN2C=C(N=N2)C=3C=CC=C(C3)S(=O)(=O)NC(=O)C=4C=CC(C)=NC4)O1	CC1=NN=C(CN=[N+]=[N-])O1	C#CC=1C=CC=C(C1)S(N)(=O)=O	CC=1C=CC(=CN1)C(=O)O	**4**{*36,6,13*}	440 [M+H]$^{+}$	65.7	66
76	CC1=NC(=CO1)C(=O)NS(=O)(=O)CCCCCC2=CN(N=N2)C(CC=3C=CC=CC3)C(N)=O	[N-]=[N+]=NC(CC=1C=CC=CC1)C(N)=O	C#CCCCCCS(N)(=O)=O	CC1=NC(=CO1)C(=O)O	**4**{*21,4,33*}	475 [M+H]$^{+}$	65.7	66
77	COC=1N=CC=NC1C(=O)NS(=O)(=O)C2=NN=C(NC(=O)CCCC3=CN(CCOCCOCCO)N=N3)S2	[N-]=[N+]=NCCOCCOCCO	C#CCCCC(=O)NC1=NN=C(S1)S(N)(=O)=O	COC=1N=CC=NC1C(=O)O	**4**{*49,5,79*}	586 [M+H]$^{+}$	65.6	66
78	O=C(CCCC1=CN(N=N1)C=2C=CC(Br)=CC2)NC3=NN=C(S3)S(=O)(=O)NC(=O)C4=CSC=N4	[N-]=[N+]=NC=1C=CC(Br)=CC1	C#CCCCC(=O)NC1=NN=C(S1)S(N)(=O)=O	O=C(O)C1=CSC=N1	**4**{*47,5,80*}	583/585 as 1/1 [M+H]$^{+}$	65.1	65
79	O=C(NS(=O)(=O)C=1C=CC=C(C1)C2=CN(N=N2)C3=CC=C(F)C(Cl)=C3F)C4=COC=N4	[N-]=[N+]=NC=1C=CC(F)=C(Cl)C1F	C#CC=1C=CC=C(C1)S(N)(=O)=O	O=C(O)C1=COC=N1	**4**{*37,6,40*}	466/468 as 3/1 [M+H]$^{+}$	64.8	65
80	CCC(CC)C(=O)NS(=O)(=O)C=1C=CC(=CC1)NC(=O)C2=CN(N=N2)C=3C=CC(OC)=C(C3)OC	COC=1C=CC(=CC1OC)N=[N+]=[N-]	C#CC(=O)NC=1C=CC(=CC1)S(N)(=O)=O	CCC(CC)C(=O)O	**4**{*6,8,7*}	502 [M+H]$^{+}$	64.4	64
81	CC=1C=CC=CC1C(=O)NS(=O)(=O)C=2C=CC=C(C2)C3=CN(CC=4C=CC(=CC4)N5C=CC=N5)N=N3	[N-]=[N+]=NCC=1C=CC(=CC1)N2C=CC=N2	C#CC=1C=CC=C(C1)S(N)(=O)=O	CC=1C=CC=CC1C(=O)O	**4**{*18,6,52*}	499 [M+H]$^{+}$	63.5	64
82	O=C(CC1CC1)NS(=O)(=O)C=2C=CC(=CC2)NC(=O)C3=CN(N=N3)C=4C=CC=NC4	[N-]=[N+]=NC=1C=CC=NC1	C#CC(=O)NC=1C=CC(=CC1)S(N)(=O)=O	O=C(O)CC1CC1	**4**{*7,8,8*}	427 [M+H]$^{+}$	63	63
83	CC1=CC=C(CN2C=C(CCCCS(=O)(=O)NC(=O)C3=CN=C(C)O3)N=N2)O1	CC1=CC=C(CN=[N+]=[N-])O1	C#CCCCCS(N)(=O)=O	CC1=NC=C(O1)C(=O)O	**4**{*59,3,87*}	408 [M+H]$^{+}$	63	63
84	CC(CC(F)F)C(=O)NS(=O)(=O)CCC1=CN(N=N1)[C@@H]2COC3(CCOCC3)[C@H]2O |&1:19,28,r|	[N-]=[N+]=N[C@H]1COC2(CCOCC2)[C@@H]1O |o1:3,12,r|	C#CCCS(N)(=O)=O	CC(CC(F)F)C(=O)O	**4**{*81,1,38*}	453 [M+H]$^{+}$	63	63
85	CC(C)C(=O)NS(=O)(=O)C1=NN=C(NC(=O)CCCC2=CN(N=N2)C=3C=CC=CC3F)S1	[N-]=[N+]=NC=1C=CC=CC1F	C#CCCCC(=O)NC1=NN=C(S1)S(N)(=O)=O	CC(C)C(=O)O	**4**{*3,5,68*}	482 [M+H]$^{+}$	62.5	63
86	CC1(CC(=O)NS(=O)(=O)CCC2=CN(CCC(F)(F)C=3C=CN=CC3)N=N2)CCC1	[N-]=[N+]=NCCC(F)(F)C=1C=CN=CC1	C#CCCS(N)(=O)=O	CC1(CC(=O)O)CCC1	**4**{*63,1,44*}	442 [M+H]$^{+}$	62.1	62
87	COC(=O)C=1C=C(F)C=C(C1)CN2C=C(CCCCS(=O)(=O)NC(=O)CCC3CCC3)N=N2	COC(=O)C1=CC(F)=CC(=C1)CN=[N+]=[N-]	C#CCCCCS(N)(=O)=O	O=C(O)CCC1CCC1	**4**{*50,3,75*}	481 [M+H]$^{+}$	61.2	61
88	O=C(NS(=O)(=O)CCCCCC1=CN(N=N1)C2=CC=C(Cl)C=C2F)[C@@H]3CC3(F)F	[N-]=[N+]=NC=1C=CC(Cl)=CC1F	C#CCCCCCS(N)(=O)=O	O=C(O)[C@@H]1CC1(F)F	**4**{*52,4,42*}	451/453 as 3/1 [M+H]$^{+}$	60.3	60
89	CC(=O)NC=1C=CC(C)=C(C1)N2C=C(N=N2)C=3C=CC=C(C3)S(=O)(=O)NC(=O)C4=CC=CS4	CC(=O)NC1=CC=C(C)C(=C1)N=[N+]=[N-]	C#CC=1C=CC=C(C1)S(N)(=O)=O	O=C(O)C1=CC=CS1	**4**{*38,6,53*}	482 [M+H]$^{+}$	59.9	60
90	CC(C)(C)C=1C=CC(=CC1)N2C=C(N=N2)C=3C=CC=C(C3)S(=O)(=O)NC(=O)C=4C=COC4	CC(C)(C)C=1C=CC(=CC1)N=[N+]=[N-]	C#CC=1C=CC=C(C1)S(N)(=O)=O	O=C(O)C=1C=COC1	**4**{*19,6,48*}	451 [M+H]$^{+}$	59.9	60
91	COC(=O)C=1C=C(C=CC1F)CN2C=C(CCCCS(=O)(=O)NC(=O)CCC3CCC3)N=N2	COC(=O)C1=CC(=CC=C1F)CN=[N+]=[N-]	C#CCCCCS(N)(=O)=O	O=C(O)CCC1CCC1	**4**{*66,3,75*}	481 [M+H]$^{+}$	58.5	59
92	CC=1C=C(C=CN1)C(=O)NS(=O)(=O)C=2C=CC(=CC2)C3=CN(CC4=CC=C(Cl)S4)N=N3	[N-]=[N+]=NCC1=CC=C(Cl)S1	C#CC=1C=CC(=CC1)S(N)(=O)=O	CC=1C=C(C=CN1)C(=O)O	**4**{*23,7,28*}	474/476 as 3/1 [M+H]$^{+}$	57.6	58
93	COC(=O)CC1CC(CCO1)N2C=C(N=N2)C=3C=CC=C(C3)S(=O)(=O)NC(=O)C=4C=NC=CC4C	COC(=O)CC1CC(CCO1)N=[N+]=[N-]	C#CC=1C=CC=C(C1)S(N)(=O)=O	CC=1C=CN=CC1C(=O)O	**4**{*39,6,54*}	500 [M+H]$^{+}$	56.7	57
94	O=C(NS(=O)(=O)CCCCCC1=CN(CC2=NN=C(O2)C3CC3)N=N1)C4CC4F	[N-]=[N+]=NCC1=NN=C(O1)C2CC2	C#CCCCCCS(N)(=O)=O	O=C(O)C1CC1F	**4**{*51,4,26*}	427 [M+H]$^{+}$	55.8	56
95	COC=1C=CC(=NN1)C(=O)NS(=O)(=O)C2=NN=C(NC(=O)CCCC3=CN(N=N3)C=4C=CC(=CC4)C(C)(C)C)S2	CC(C)(C)C=1C=CC(=CC1)N=[N+]=[N-]	C#CCCCC(=O)NC1=NN=C(S1)S(N)(=O)=O	COC=1C=CC(=NN1)C(=O)O	**4**{*19,5,37*}	586 [M+H]$^{+}$	55.4	55
96	CC1CC1CC(=O)NS(=O)(=O)CCC2=CN(N=N2)[C@@H]3COC4(CCOCC4)[C@H]3O |&1:18,27,r|	[N-]=[N+]=N[C@H]1COC2(CCOCC2)[C@@H]1O |o1:3,12,r|	C#CCCS(N)(=O)=O	CC1CC1CC(=O)O	**4**{*81,1,93*}	429 [M+H]$^{+}$	55.4	55
97	O=C(NC=1C=CC(=CC1)S(=O)(=O)NC(=O)C2CC(F)(F)C2)C3=CN(N=N3)C=4C=CC=CC4F	[N-]=[N+]=NC=1C=CC=CC1F	C#CC(=O)NC=1C=CC(=CC1)S(N)(=O)=O	O=C(O)C1CC(F)(F)C1	**4**{*3,8,9*}	480 [M+H]$^{+}$	53.1	53
98	CCOC(=O)C1CCC(=CN2C=C(CCCS(=O)(=O)NC(=O)CC3CCN(C)C3=O)N=N2)CC1	CCOC(=O)C1CCC(=CN=[N+]=[N-])CC1	C#CCCCS(N)(=O)=O	CN1CCC(CC(=O)O)C1=O	**4**{*73,2,24*}	496 [M+H]$^{+}$	53.1	53
99	O=C(NS(=O)(=O)C=1C=CC=C(C1)C2=CN(N=N2)C=3C=C(F)C(Cl)=C(F)C3)C4=COC=N4	[N-]=[N+]=NC=1C=C(F)C(Cl)=C(F)C1	C#CC=1C=CC=C(C1)S(N)(=O)=O	O=C(O)C1=COC=N1	**4**{*40,6,40*}	466/468 as 3/1 [M+H]$^{+}$	52.2	52
100	COC=1C=CC(CN2C=C(CCCS(=O)(=O)NC(=O)C3=COC=C3C)N=N2)=NN1	COC=1C=CC(CN=[N+]=[N-])=NN1	C#CCCCS(N)(=O)=O	CC1=COC=C1C(=O)O	**4**{*76,2,67*}	421 [M+H]$^{+}$	52.2	52
101	CC1(CC(=O)NS(=O)(=O)CCCCC2=CN(CCC(F)(F)C=3C=CN=CC3)N=N2)CC1	[N-]=[N+]=NCCC(F)(F)C=1C=CN=CC1	C#CCCCCS(N)(=O)=O	CC1(CC(=O)O)CC1	**4**{*63,3,71*}	456 [M+H]$^{+}$	51.8	52
102	CCOC(=O)C1CCC(=CN2C=C(CCS(=O)(=O)NC(=O)C3CC3Cl)N=N2)CC1	CCOC(=O)C1CCC(=CN=[N+]=[N-])CC1	C#CCCS(N)(=O)=O	O=C(O)C1CC1Cl	**4**{*73,1,23*}	445/447 as 3/1 [M+H]$^{+}$	51.3	51

103	CC=1C=NC=C(N1)C(=O)NS(=O)(=O)C2=NN=C(NC(=O)CCCC3=CN(N=N3)C=4C=CC=CC4)S2	[N-]=[N+]=NC=1C=CC=CC1	C#CCCCC(=O)NC1=NN=C(S1)S(N)(=O)=O	CC=1C=NC=C(N1)C(=O)O	**4**{*48,5,30*}	514 $[M+H]^+$	50.9	51
104	CC(C)(C)CC(=O)NS(=O)(=O)C1=NN=C(NC(=O)CCCC2=CN(CCOCCOCCO)N=N2)S1	[N-]=[N+]=NCCOCCOCCO	C#CCCCC(=O)NC1=NN=C(S1)S(N)(=O)=O	CC(C)(C)CC(=O)O	**4**{*49,5,81*}	548 $[M+H]^+$	49.5	50
105	COC(=O)C1CCC2(CC(C2)N3C=C(CCCS(=O)(=O)NC(=O)CC4(C)CCC4)N=N3)C1	COC(=O)C1CCC2(CC(C2)N=[N+]=[N-])C1	C#CCCCS(N)(=O)=O	CC1(CC(=O)O)CCC1	**4**{*75,2,44*}	467 $[M+H]^+$	49.5	50
106	COC(=O)C=1C=CC=C(CN2C=C(CCCCS(=O)(=O)NC(=O)C34CC(C)(C3)C4)N=N2)C1F	COC(=O)C1=CC=CC(CN=[N+]=[N-])=C1F	C#CCCCCS(N)(=O)=O	CC12CC(C1)(C2)C(=O)O	**4**{*65,3,64*}	479 $[M+H]^+$	49.1	49
107	CC1(CC(=O)NS(=O)(=O)CCCC2=CN(N=N2)C=3C=CC(Cl)=C(F)C3F)CCC1	[N-]=[N+]=NC=1C=CC(Cl)=C(F)C1F	C#CCCCS(N)(=O)=O	CC1(CC(=O)O)CCC1	**4**{*77,2,44*}	447/449 as 3/1 $[M+H]^+$	49.1	49
108	CC=1C=CC=C(C1)C(=O)NS(=O)(=O)C=2C=CC(=CC2)NC(=O)C3=CN(N=N3)C(C)C(=O)NC=4C=CC=CC4	CC(N=[N+]=[N-])C(=O)NC=1C=CC=CC1	C#CC(=O)NC=1C=CC(=CC1)S(N)(=O)=O	CC=1C=CC=C(C1)C(=O)O	**4**{*8,8,3*}	533 $[M+H]^+$	48.6	49
109	O=C(CCCC1=CN(N=N1)C=2C=CC=CC2)NC3=NN=C(S3)S(=O)(=O)NC(=O)C4=CSC=N4	[N-]=[N+]=NC=1C=CC=CC1	C#CCCCC(=O)NC1=NN=C(S1)S(N)(=O)=O	O=C(O)C1=CSC=N1	**4**{*48,5,80*}	505 $[M+H]^+$	48.6	49
110	CCOCC(=O)NS(=O)(=O)C=1C=CC(=CC1)NC(=O)C2=CN(N=N2)C=3C=CC(Br)=CN3	[N-]=[N+]=NC=1C=CC(Br)=CN1	C#CC(=O)NC=1C=CC(=CC1)S(N)(=O)=O	CCOCC(=O)O	**4**{*9,8,1*}	509/511 as 1/1 $[M+H]^+$	48.2	48
111	O=C(NS(=O)(=O)CCCCCC1=CN(CC2=NN=C(O2)C3CC3)N=N1)C=4C=NSC4	[N-]=[N+]=NCC1=NN=C(O1)C2CC2	C#CCCCCCS(N)(=O)=O	O=C(O)C=1C=NSC1	**4**{*51,4,27*}	452 $[M+H]^+$	47.3	47
112	CC=1N=CC=NC1C(=O)NS(=O)(=O)C=2C=CC(=CC2)C3=CN(N=N3)C4=C(F)C(F)=CC(F)=C4F	[N-]=[N+]=NC=1C(F)=C(F)C=C(F)C1F	C#CC=1C=CC(=CC1)S(N)(=O)=O	CC=1N=CC=NC1C(=O)O	**4**{*24,7,34*}	493 $[M+H]^+$	46.5	47
113	O=C(NS(=O)(=O)CCCCCC1=CN(CC2=NN=C(O2)C3CC3)N=N1)[C@@H]4CC4(F)F	[N-]=[N+]=NCC1=NN=C(O1)C2CC2	C#CCCCCCS(N)(=O)=O	O=C(O)[C@@H]1CC1(F)F	**4**{*51,4,42*}	445 $[M+H]^+$	46.4	46
114	N#CC1CCC(C1)N2C=C(N=N2)C=3C=CC(=CC3)S(=O)(=O)NC(=O)C4=CN=CS4	[N-]=[N+]=NC1CCC(C#N)C1	C#CC=1C=CC(=CC1)S(N)(=O)=O	O=C(O)C1=CN=CS1	**4**{*22,7,35*}	429 $[M+H]^+$	45.6	46
115	N#CC1CCC(C1)N2C=C(CCCC(=O)NC3=NN=C(S3)S(=O)(=O)NC(=O)C4CC(F)C4)N=N2	[N-]=[N+]=NC1CCC(C#N)C1	C#CCCCC(=O)NC1=NN=C(S1)S(N)(=O)=O	O=C(O)C1CC(F)C1	**4**{*22,5,83*}	511 $[M+H]^+$	45.5	46
116	CC1(F)CC(C1)C(=O)NS(=O)(=O)CCCC2=CN(N=N2)C=3C=CC(Cl)=C(F)C3F	[N-]=[N+]=NC=1C=CC(Cl)=C(F)C1F	C#CCCCS(N)(=O)=O	CC1(F)CC(C1)C(=O)O	**4**{*77,2,46*}	451/453 as 3/1 $[M+H]^+$	45.5	46
117	O=C(NS(=O)(=O)C=1C=CC(=CC1)C2=CN(N=N2)C3=C(F)C(F)=CC(F)=C3F)C=4C=CC=NN4	[N-]=[N+]=NC=1C(F)=C(F)C=C(F)C1F	C#CC=1C=CC(=CC1)S(N)(=O)=O	O=C(O)C=1C=CC=NN1	**4**{*24,7,36*}	479 $[M+H]^+$	44.7	45
118	CC=1C=CC=C(N1)C(=O)NS(=O)(=O)C=2C=CC=C(C2)C3=CN(N=N3)C=4C=C(F)C(Cl)=C(F)C4	[N-]=[N+]=NC=1C=C(F)C(Cl)=C(F)C1	C#CC=1C=CC=C(C1)S(N)(=O)=O	CC=1C=CC=C(N1)C(=O)O	**4**{*40,6,55*}	490/492 as 3/1 $[M+H]^+$	44.6	45
119	CC=1C=CN=CC1C(=O)NS(=O)(=O)C=2C=CC=C(C2)C3=CN(N=N3)C=4C=CN=CC4	[N-]=[N+]=NC=1C=CN=CC1	C#CC=1C=CC=C(C1)S(N)(=O)=O	CC=1C=CN=CC1C(=O)O	**4**{*41,6,54*}	421 $[M+H]^+$	44.6	45
120	CC(F)(F)C=1C=CC=C(CN2C=C(CCCCS(=O)(=O)NC(=O)C3CC43CCC4)N=N2)N1	CC(F)(F)C=1C=CC=C(CN=[N+]=[N-])N1	C#CCCCCS(N)(=O)=O	O=C(O)C1CC21CCC2	**4**{*67,3,29*}	468 $[M+H]^+$	44.1	44
121	CC(C)C(=O)NS(=O)(=O)C1=NN=C(NC(=O)CCCC2=CN(CCS(C)(=O)=O)N=N2)S1	CS(=O)(=O)CCN=[N+]=[N-]	C#CCCCC(=O)NC1=NN=C(S1)S(N)(=O)=O	CC(C)C(=O)O	**4**{*45,5,68*}	494 $[M+H]^+$	42.8	43
122	O=C(NS(=O)(=O)C=1C=CC=C(C1)C2=CN(N=N2)C=3C=C(F)C(Cl)=C(F)C3)C=4C=NC=NC4	[N-]=[N+]=NC=1C=C(F)C(Cl)=C(F)C1	C#CC=1C=CC=C(C1)S(N)(=O)=O	O=C(O)C=1C=NC=NC1	**4**{*40,6,56*}	477/479 as 3/1 $[M+H]^+$	41.9	42
123	CC=1C=CC=NC1C(=O)NS(=O)(=O)C=2C=CC(=CC2)NC(=O)C3=CN(N=N3)C=4C=CC=C(C4)C(C)(C)C	CC(C)(C)C1=CC=CC(=C1)N=[N+]=[N-]	C#CC(=O)NC=1C=CC(=CC1)S(N)(=O)=O	CC=1C=CC=NC1C(=O)O	**4**{*10,8,10*}	519 $[M+H]^+$	41.4	41
124	CC(C(=O)NC=1C=CC=CC1)N2C=C(N=N2)C(=O)NC=3C=CC(=CC3)S(=O)(=O)NC(=O)C4CC4(F)F	CC(N=[N+]=[N-])C(=O)NC=1C=CC=CC1	C#CC(=O)NC=1C=CC(=CC1)S(N)(=O)=O	O=C(O)C1CC1(F)F	**4**{*8,8,11*}	519 $[M+H]^+$	41.4	41
125	COC(=O)C1=CC=CC(CN2C=C(CCCCS(=O)(=O)NC(=O)CN3CCCCC3=O)N=N2)=C1F	COC(=O)C1=CC=CC(CN=[N+]=[N-])=C1F	C#CCCCCS(N)(=O)=O	O=C(O)CN1CCCCC1=O	**4**{*65,3,89*}	510 $[M+H]^+$	41.4	41
126	O=C(NS(=O)(=O)CCCC1=CN(N=N1)C=2C=CC(Cl)=C(F)C2F)C=3SC=NC3Cl	[N-]=[N+]=NC=1C=CC(Cl)=C(F)C1F	C#CCCCS(N)(=O)=O	O=C(O)C=1SC=NC1Cl	**4**{*77,2,45*}	480/482/484 as 9/6/1 $[M–H]^–$	41.4	41
127	CC(=O)C=1C=CC=C(C1)CN2C=C(N=N2)C(=O)NC=3C=CC(=CC3)S(=O)(=O)NC(=O)C4=CC=CO4	CC(=O)C1=CC=CC(=C1)CN=[N+]=[N-]	C#CC(=O)NC=1C=CC(=CC1)S(N)(=O)=O	O=C(O)C1=CC=CO1	**4**{*11,8,6*}	494 $[M+H]^+$	41	41
128	O=C(NS(=O)(=O)C=1C=CC(=CC1)C2=CN(N=N2)C3=C(F)C(F)=CC(F)=C3F)C4=CN=CS4	[N-]=[N+]=NC=1C(F)=C(F)C=C(F)C1F	C#CC=1C=CC(=CC1)S(N)(=O)=O	O=C(O)C1=CN=CS1	**4**{*24,7,35*}	484 $[M+H]^+$	40.4	40
129	CC(CC(F)F)C(=O)NS(=O)(=O)CCC1=CN(N=N1)C=2C=CC(F)=CC2	[N-]=[N+]=NC=1C=CC(F)=CC1	C#CCCS(N)(=O)=O	CC(CC(F)F)C(=O)O	**4**{*44,1,38*}	391 $[M+H]^+$	39.6	40
130	CC=1C=C(CN2C=C(N=N2)C=3C=CC(=CC3)S(=O)(=O)NC(=O)C=4C=CSC4)ON1	CC=1C=C(CN=[N+]=[N-])ON1	C#CC=1C=CC(=CC1)S(N)(=O)=O	O=C(O)C=1C=CSC1	**4**{*20,7,32*}	430 $[M+H]^+$	39.5	40
131	CC=1C=CN=CC1C(=O)NS(=O)(=O)C=2C=CC=C(C2)C3=CN(N=N3)C=4C(F)=CC(Cl)=CC4F	[N-]=[N+]=NC=1C(F)=CC(Cl)=CC1F	C#CC=1C=CC=C(C1)S(N)(=O)=O	CC=1C=CN=CC1C(=O)O	**4**{*42,6,54*}	490/492 as 3/1 $[M+H]^+$	39.2	39
132	CC1CC1C(=O)NS(=O)(=O)CCCCCC2=CN(CC(F)(F)CO)N=N2	[N-]=[N+]=NCC(F)(F)CO	C#CCCCCCS(N)(=O)=O	CC1CC1C(=O)O	**4**{*25,4,84*}	395 $[M+H]^+$	39.2	39
133	O=C(NS(=O)(=O)C=1C=CC(=CC1)C2=CN(CC(F)(F)CO)N=N2)C=3C=NSC3	[N-]=[N+]=NCC(F)(F)CO	C#CC=1C=CC(=CC1)S(N)(=O)=O	O=C(O)C=1C=NSC1	**4**{*25,7,27*}	430 $[M+H]^+$	39	39
134	COC=1C=CC(CN2C=C(CCCS(=O)(=O)NC(=O)CC(C)C(F)F)N=N2)=NN1	COC=1C=CC(CN=[N+]=[N-])=NN1	C#CCCCS(N)(=O)=O	CC(CC(=O)O)C(F)F	**4**{*76,2,69*}	433 $[M+H]^+$	38.7	39
135	CC1(F)CC(C1)C(=O)NS(=O)(=O)CCCC2=CN(CC=3C=NC(=NC3)C4CC4)N=N2	[N-]=[N+]=NCC=1C=NC(=NC1)C2CC2	C#CCCCS(N)(=O)=O	CC1(F)CC(C1)C(=O)O	**4**{*64,2,46*}	437 $[M+H]^+$	38.3	38
136	COC=1N=CC=NC1C(=O)NS(=O)(=O)C2=NN=C(NC(=O)CCCC3=CN(N=N3)C=4C=CC(Br)=CC4)S2	[N-]=[N+]=NC=1C=CC(Br)=CC1	C#CCCCC(=O)NC1=NN=C(S1)S(N)(=O)=O	COC=1N=CC=NC1C(=O)O	**4**{*47,5,79*}	608/610 as 1/1 $[M+H]^+$	36.9	37
137	COC(=O)C=1C=C(F)C=C(C1)CN2C=C(CCCCS(=O)(=O)NC(=O)C34CC(C3)C4)N=N2	COC(=O)C1=CC(F)=CC(=C1)CN=[N+]=[N-]	C#CCCCCS(N)(=O)=O	O=C(O)C12CC(C1)C2	**4**{*50,3,90*}	465 $[M+H]^+$	36.5	37
138	O=C(CCCC1=CN(N=N1)C=2C=CC=CC2F)NC3=NN=C(S3)S(=O)(=O)NC(=O)C4CC4	[N-]=[N+]=NC=1C=CC=CC1F	C#CCCCC(=O)NC1=NN=C(S1)S(N)(=O)=O	O=C(O)C1CC1	**4**{*3,5,2*}	480 $[M+H]^+$	36	36
139	CC1(F)CC(C1)C(=O)NS(=O)(=O)CCCC2=CN(C[C@@H]3C[C@H]3C(F)(F)F)N=N2 |&1:19,21,r|	[N-]=[N+]=NC[C@@H]1C[C@H]1C(F)(F)F |o1:4,6,r|	C#CCCCS(N)(=O)=O	CC1(F)CC(C1)C(=O)O	**4**{*78,2,46*}	427 $[M+H]^+$	36	36
140	CC1=COC=C1C(=O)NS(=O)(=O)CCCC2=CN(CC=3C=CC(=CN3)C(C)(F)F)N=N2	CC(F)(F)C1=CC=C(CN=[N+]=[N-])N=C1	C#CCCCS(N)(=O)=O	CC1=COC=C1C(=O)O	**4**{*70,2,67*}	454 $[M+H]^+$	35.1	35
141	CC1=CC(C)=C(C)C=C1CN2C=C(N=N2)C(=O)NC=3C=CC(=CC3)S(=O)(=O)NC(=O)C=4C=CC=NC4C	CC1=CC(C)=C(C=C1C)CN=[N+]=[N-]	C#CC(=O)NC=1C=CC(=CC1)S(N)(=O)=O	CC=1N=CC=CC1C(=O)O	**4**{*5,8,12*}	519 $[M+H]^+$	34.2	34
142	O=C(CCCC1=CN(N=N1)C=2C=C(F)C(Cl)=C(F)C2)NC3=NN=C(S3)S(=O)(=O)NC(=O)C=4C=CSC4	[N-]=[N+]=NC=1C=C(F)C(Cl)=C(F)C1	C#CCCCC(=O)NC1=NN=C(S1)S(N)(=O)=O	O=C(O)C=1C=CSC1	**4**{*40,5,32*}	574/576 as 3/1 $[M+H]^+$	32.4	32
143	CC(F)(F)C=1C=NC=C(C1)CN2C=C(CCS(=O)(=O)NC(=O)COCCCF)N=N2	CC(F)(F)C1=CN=CC(=C1)CN=[N+]=[N-]	C#CCCS(N)(=O)=O	O=C(O)COCCCF	**4**{*83,1,94*}	519 $[M+H]^+$	31.5	32
144	CC(=O)NC=1C=CC(C)=C(C1)N2C=C(N=N2)C3=CC=CC(=C3)S(=O)(=O)NC(=O)C=4C=CC=CC4C	CC(=O)NC1=CC=C(C)C(=C1)N=[N+]=[N-]	C#CC=1C=CC=C(C1)S(N)(=O)=O	CC=1C=CC=CC1C(=O)O	**4**{*38,6,52*}	490 $[M+H]^+$	31.1	31
145	CC1=COC(=C1)C(=O)NS(=O)(=O)CCCC2=CN(CC=3C=NC(C)=CN3)N=N2	CC=1C=NC(=CN1)CN=[N+]=[N-]	C#CCCCS(N)(=O)=O	CC1=COC(=C1)C(=O)O	**4**{*74,2,63*}	405 $[M+H]^+$	31.1	31
146	O=C(NS(=O)(=O)CCCCCC1=CN(CC=2C=C(ON2)C3=CC=CO3)N=N1)C4CC4F	[N-]=[N+]=NCC=1C=C(ON1)C2=CC=CO2	C#CCCCCCS(N)(=O)=O	O=C(O)C1CC1F	**4**{*53,4,26*}	452 $[M+H]^+$	30.6	31
147	COC1=CC=CC(OC)=C1CN2C=C(CCS(=O)(=O)NC(=O)C3=COC=C3C)N=N2	COC1=CC=CC(OC)=C1CN=[N+]=[N-]	C#CCCS(N)(=O)=O	CC1=COC=C1C(=O)O	**4**{*54,1,67*}	435 $[M+H]^+$	29.7	30
148	CC=1C=NC=C(N1)C(=O)NS(=O)(=O)C2=NN=C(NC(=O)CCCC3=CN(CCOCCOCCO)N=N3)S2	[N-]=[N+]=NCCOCCOCCO	C#CCCCC(=O)NC1=NN=C(S1)S(N)(=O)=O	CC=1C=NC=C(N1)C(=O)O	**4**{*49,5,30*}	570 $[M+H]^+$	29.3	29
149	COC=1C=CC(=NN1)C(=O)NS(=O)(=O)C=2C=CC(=CC2)C3=CN(N=N3)C=4C=CN=CC4Br	[N-]=[N+]=NC=1C=CN=CC1Br	C#CC=1C=CC(=CC1)S(N)(=O)=O	COC=1C=CC(=NN1)C(=O)O	**4**{*26,7,37*}	516/518 as 1/1 $[M+H]^+$	29.1	29
150	COC1=CC=CC(OC)=C1CN2C=C(CCCCCS(=O)(=O)NC(=O)C3CC(F)C3)N=N2	COC1=CC=CC(OC)=C1CN=[N+]=[N-]	C#CCCCCCS(N)(=O)=O	O=C(O)C1CC(F)C1	**4**{*54,4,83*}	469 $[M+H]^+$	28.8	29
151	CC12CC(C1)(C2)C(=O)NS(=O)(=O)CCC3=CN(CC(F)(F)CO)N=N3	[N-]=[N+]=NCC(F)(F)CO	C#CCCS(N)(=O)=O	CC12CC(C1)(C2)C(=O)O	**4**{*25,1,64*}	379 $[M+H]^+$	28.8	29
152	CC1=NC=2C=C(C=CC2S1)N3C=C(CCCCCS(=O)(=O)NC(=O)C=4C=CN(C)N4)N=N3	CC1=NC2=CC(=CC=C2S1)N=[N+]=[N-]	C#CCCCCCS(N)(=O)=O	CN1C=CC(=N1)C(=O)O	**4**{*55,4,85*}	474 $[M+H]^+$	27.5	28
153	O=C(COCCCF)NS(=O)(=O)CCC1=CN(N=N1)C=2C=CC(F)=CC2	[N-]=[N+]=NC=1C=CC(F)=CC1	C#CCCS(N)(=O)=O	O=C(O)COCCCF	**4**{*44,1,94*}	389 $[M+H]^+$	27.5	28
154	O=C(COCCCF)NS(=O)(=O)CCC1=CN(N=N1)[C@@H]2COC3(CCOCC3)[C@H]2O |&1:19,28,r|	[N-]=[N+]=N[C@H]1COC2(CCOCC2)[C@@H]1O |o1:3,12,r|	C#CCCS(N)(=O)=O	O=C(O)COCCCF	**4**{*81,1,94*}	451 $[M+H]^+$	27	27
155	CC(C(=O)NC=1C=CC=CC1)N2C=C(N=N2)C(=O)NC=3C=CC(=CC3)S(=O)(=O)NC(=O)C4CC(F)(F)C4	CC(N=[N+]=[N-])C(=O)NC=1C=CC=CC1	C#CC(=O)NC=1C=CC(=CC1)S(N)(=O)=O	O=C(O)C1CC(F)(F)C1	**4**{*8,8,9*}	533 $[M+H]^+$	26.1	26
156	COC(=O)C=1C=NN(C)C1CCN2C=C(CCCS(=O)(=O)NC(=O)CC3(C)CCC3)N=N2	COC(=O)C=1C=NN(C)C1CCN=[N+]=[N-]	C#CCCCS(N)(=O)=O	CC1(CC(=O)O)CCC1	**4**{*71,2,44*}	467 $[M+H]^+$	24.8	25
157	CCOC(=O)C1CCC(=CN2C=C(CCS(=O)(=O)NC(=O)C=3C=NN(C)N3)N=N2)CC1	CCOC(=O)C1CCC(=CN=[N+]=[N-])CC1	C#CCCS(N)(=O)=O	CN1N=CC(=N1)C(=O)O	**4**{*73,1,76*}	452 $[M+H]^+$	24.8	25
158	O=C(O)C(F)(F)F.CC=1C=CC(=CN1)C(=O)NS(=O)(=O)C=2C=CC(=CC2)NC(=O)C3=CN(CCCN4CCCC4)N=N3	Cl.[N-]=[N+]=NCCCN1CCCC1	C#CC(=O)NC=1C=CC(=CC1)S(N)(=O)=O	CC=1C=CC(=CN1)C(=O)O	**4**{*12,8,13*}	498 $[M–TFA+H]^+$	23.9	24

159	COC(=O)C=1C=CC=C(CN2C=C(CCCCS(=O)(=O)NC(=O)C=3C=CC=C4CCCC34)N=N2)C1F	COC(=O)C1=CC=CC(CN=[N+]=[N-])=C1F	C#CCCCCS(N)(=O)=O	O=C(O)C1=CC=CC=2CCCC12	**4**{*65,3,77*}	515 [M+H]$^{+}$	23.4	23
160	O=C(O)C(F)(F)F.O=C(NC=1C=CC(=CC1)S(=O)(=O)NC(=O)C=2C=CC(F)=CC2)C3=CN(CCCN4CCCC4)N=N3	Cl.[N-]=[N+]=NCCCN1CCCC1	C#CC(=O)NC=1C=CC(=CC1)S(N)(=O)=O	O=C(O)C=1C=CC(F)=CC1	**4**{*12,8,14*}	501 [M-TFA+H]$^{+}$	22.1	22
161	O=C(NS(=O)(=O)C=1C=CC=C(C1)C2=CN(N=N2)C=3C(F)=CC(Cl)=CC3F)C=4C=NC=CN4	[N-]=[N+]=NC=1C(F)=CC(Cl)=CC1F	C#CC=1C=CC=C(C1)S(N)(=O)=O	O=C(O)C=1C=NC=CN1	**4**{*42,6,51*}	477/479 as 3/1 [M+H]$^{+}$	22.1	22
162	CN1N=CC(=N1)C(=O)NS(=O)(=O)CCC2=CN(N=N2)C3CCC4=CC=CN=C43	Cl.[N-]=[N+]=NC1CCC2=CC=CN=C21	C#CCCS(N)(=O)=O	CN1N=CC(=N1)C(=O)O	**4**{*82,1,76*}	403 [M+H]$^{+}$	22.1	22
163	CC(=O)C=1C=CC(=CC1)CN2C=C(N=N2)C3=CC=CC(=C3)S(=O)(=O)NC(=O)C4=CC=CC(C)=N4	CC(=O)C=1C=CC(=CC1)CN=[N+]=[N-]	C#CC=1C=CC=C(C1)S(N)(=O)=O	CC=1C=CC=C(N1)C(=O)O	**4**{*43,6,55*}	476 [M+H]$^{+}$	21.2	21
164	CC1=CC=C(O1)C(=O)NS(=O)(=O)C=2C=CC=C(C2)C3=CN(N=N3)C=4C=CC=CN4	[N-]=[N+]=NC=1C=CC=CN1	C#CC=1C=CC=C(C1)S(N)(=O)=O	CC1=CC=C(O1)C(=O)O	**4**{*4,6,43*}	410 [M+H]$^{+}$	20.3	20
165	CC=1N=CC=CC1C(=O)NS(=O)(=O)C=2C=CC(=CC2)NC(=O)C3=CN(CC=4C=CC=C(C4)C=5C=CC=CC5)N=N3	[N-]=[N+]=NCC=1C=CC=C(C1)C=2C=CC=CC2	C#CC(=O)NC=1C=CC(=CC1)S(N)(=O)=O	CC=1N=CC=CC1C(=O)O	**4**{*13,8,12*}	553 [M+H]$^{+}$	17.6	18
166	CN1N=NC=C1C(=O)NS(=O)(=O)CCCCCC2=CN(CC3=NN=C(O3)C4CC4)N=N2	[N-]=[N+]=NCC1=NN=C(O1)C2CC2	C#CCCCCCS(N)(=O)=O	CN1N=NC=C1C(=O)O	**4**{*51,4,86*}	450 [M+H]$^{+}$	16.2	16
167	CC=1N=CC=NC1C(=O)NS(=O)(=O)C=2C=CC(=CC2)C3=CN(N=N3)C=4C=CC=CC4S(C)(=O)=O	CS(=O)(=O)C=1C=CC=CC1N=[N+]=[N-]	C#CC=1C=CC(=CC1)S(N)(=O)=O	CC=1N=CC=NC1C(=O)O	**4**{*27,7,34*}	499 [M+H]$^{+}$	15.5	16
168	O=C(NS(=O)(=O)CCCCCC1=CN(CC2COC3=CC=CC=C3O2)N=N1)C4=CC=NS4	[N-]=[N+]=NCC1COC=2C=CC=CC2O1	C#CCCCCCS(N)(=O)=O	O=C(O)C1=CC=NS1	**4**{*17,4,22*}	478 [M+H]$^{+}$	14.4	14
169	COC=1C=CC(=NC1)N2C=C(CCCCS(=O)(=O)NC(=O)CC3(C)CC3)N=N2	COC=1C=CC(N=[N+]=[N-])=NC1	C#CCCCCS(N)(=O)=O	CC1(CC(=O)O)CC1	**4**{*68,3,71*}	408 [M+H]$^{+}$	14	14
170	CC(CC(F)F)C(=O)NS(=O)(=O)CCC1=CN(CC=2C=NC=C(C2)C(C)(F)F)N=N1	CC(F)(F)C1=CN=CC(=C1)CN=[N+]=[N-]	C#CCCS(N)(=O)=O	CC(CC(F)F)C(=O)O	**4**{*83,1,38*}	452 [M+H]$^{+}$	14	14
171	CC1=NC=2C=CC(=CC2S1)N3C=C(N=N3)C=4C=CC(=CC4)S(=O)(=O)NC(=O)C=5N=COC5C	CC1=NC2=CC=C(C=C2S1)N=[N+]=[N-]	C#CC=1C=CC(=CC1)S(N)(=O)=O	CC=1OC=NC1C(=O)O	**4**{*28,7,20*}	481 [M+H]$^{+}$	13.6	14
172	CC=1N=CC=NC1C(=O)NS(=O)(=O)C=2C=CC(=CC2)C3=CN(N=N3)C=4C=CC=5N=CNC5C4	[N-]=[N+]=NC1=CC=C2N=CNC2=C1	C#CC=1C=CC(=CC1)S(N)(=O)=O	CC=1N=CC=NC1C(=O)O	**4**{*29,7,34*}	461 [M+H]$^{+}$	13.6	14
173	CN1C=C(C=N1)C(=O)NS(=O)(=O)C=2C=CC=C(C2)C3=CN(N=N3)C=4C=CC(F)=CC4	[N-]=[N+]=NC=1C=CC(F)=CC1	C#CC=1C=CC=C(C1)S(N)(=O)=O	CN1C=C(C=N1)C(=O)O	**4**{*44,6,47*}	427 [M+H]$^{+}$	12.2	12
174	COC=1C=NN=C(C1)C(=O)NS(=O)(=O)CCC2=CN(CC3=CN=CC(=C3)C(C)(F)F)N=N2	CC(F)(F)C1=CN=CC(=C1)CN=[N+]=[N-]	C#CCCS(N)(=O)=O	COC=1C=NN=C(C1)C(=O)O	**4**{*83,1,95*}	468 [M+H]$^{+}$	11.7	12
175	COC(=O)C=1C=C(C=CC1F)CN2C=C(CCCCS(=O)(=O)NC(=O)CN3CCCCC3=O)N=N2	COC(=O)C1=CC(=CC=C1F)CN=[N+]=[N-]	C#CCCCCS(N)(=O)=O	O=C(O)CN1CCCCC1=O	**4**{*66,3,89*}	510 [M+H]$^{+}$	10.8	11
176	CC=1C=CC=NC1C(=O)NS(=O)(=O)C=2C=CC(=CC2)NC(=O)C3=CN(N=N3)C=4C=CC(Cl)=C(Cl)C4	[N-]=[N+]=NC=1C=CC(Cl)=C(Cl)C1	C#CC(=O)NC=1C=CC(=CC1)S(N)(=O)=O	CC=1C=CC=NC1C(=O)O	**4**{*14,8,10*}	531/533/535 as 9/6/1 [M+H]$^{+}$	10.4	10
177	CC=1OC=NC1C(=O)NS(=O)(=O)C=2C=CC(=CC2)C3=CN(N=N3)C=4C=CN=C(C4)CO	[N-]=[N+]=NC=1C=CN=C(C1)CO	C#CC=1C=CC(=CC1)S(N)(=O)=O	CC=1OC=NC1C(=O)O	**4**{*30,7,20*}	439 [M–H]$^{-}$	9.9	10
178	O=C(NS(=O)(=O)CCCCCC1=CN(CC=2C=C(ON2)C3=CC=CO3)N=N1)C4CC(F)C4	[N-]=[N+]=NCC=1C=C(ON1)C2=CC=CO2	C#CCCCCCS(N)(=O)=O	O=C(O)C1CC(F)C1	**4**{*53,4,83*}	466 [M+H]$^{+}$	9.5	10
179	COC=1C=CC(=CC1OC)N2C=C(N=N2)C(=O)NC=3C=CC(=CC3)S(=O)(=O)NC(=O)C4=NC=CS4	COC=1C=CC(=CC1OC)N=[N+]=[N-]	C#CC(=O)NC=1C=CC(=CC1)S(N)(=O)=O	O=C(O)C1=NC=CS1	**4**{*6,8,15*}	515 [M+H]$^{+}$	7.7	8
180	COC=1C=NN=C(C1)C(=O)NS(=O)(=O)CCC2=CN(N=N2)C=3C=CC(F)=CC3	[N-]=[N+]=NC=1C=CC(F)=CC1	C#CCCS(N)(=O)=O	COC=1C=NN=C(C1)C(=O)O	**4**{*44,1,95*}	407 [M+H]$^{+}$	7.2	7
181	CC1=CON=C1C(=O)NS(=O)(=O)CCC2=CN(CC3=NN=C(O3)C4CC4)N=N2	[N-]=[N+]=NCC1=NN=C(O1)C2CC2	C#CCCS(N)(=O)=O	CC1=CON=C1C(=O)O	**4**{*51,1,65*}	408 [M+H]$^{+}$	6.8	7
182	CC(F)(F)C=1C=CC=C(CN2C=C(CCCCS(=O)(=O)NC(=O)C34CC(C3)C4)N=N2)N1	CC(F)(F)C=1C=CC=C(CN=[N+]=[N-])N1	C#CCCCCS(N)(=O)=O	O=C(O)C12CC(C1)C2	**4**{*67,3,90*}	454 [M+H]$^{+}$	5.9	6
183	CC1=CC=C(CN2C=C(CCCCS(=O)(=O)NC(=O)[C@@H]3COCCO3)N=N2)O1	CC1=CC=C(CN=[N+]=[N-])O1	C#CCCCCS(N)(=O)=O	O=C(O)[C@@H]1COCCO1	**4**{*59,3,59*}	413 [M+H]$^{+}$	5	5
184	CCC(CC)C(=O)NS(=O)(=O)C=1C=CC(=CC1)NC(=O)C2=CN(C[C@H](O)C=3C=CC=CC3OC)N=N2	COC=1C=CC=CC1[C@@H](O)CN=[N+]=[N-]	C#CC(=O)NC=1C=CC(=CC1)S(N)(=O)=O	CCC(CC)C(=O)O	**4**{*15,8,7*}	516 [M+H]$^{+}$	4.5	5
185	O=C(NC=1C=CC(=CC1)S(=O)(=O)NC(=O)C=2C=CC(F)=CC2)C3=CN(N=N3)C=4C=CC(Br)=CN4	[N-]=[N+]=NC=1C=CC(Br)=CN1	C#CC(=O)NC=1C=CC(=CC1)S(N)(=O)=O	O=C(O)C=1C=CC(F)=CC1	**4**{*9,8,14*}	545/547 as 1/1 [M+H]$^{+}$	4.5	5
186	O=C(O)C(F)(F)F.O=C(NS(=O)(=O)C=1C=CC=C(C1)C2=CN(N=N2)C=3C=CC=4N=CNC4C3)C5=CN=CS5	[N-]=[N+]=NC1=CC=C2N=CNC2=C1	C#CC=1C=CC=C(C1)S(N)(=O)=O	O=C(O)C1=CN=CS1	**4**{*29,6,35*}	452 [M–TFA+H]$^{+}$	2.7	3
187	CC(C(=O)NC=1C=CC=CC1)N2C=C(N=N2)C(=O)NC=3C=CC(=CC3)S(=O)(=O)NC(=O)C4=NC=CS4	CC(N=[N+]=[N-])C(=O)NC=1C=CC=CC1	C#CC(=O)NC=1C=CC(=CC1)S(N)(=O)=O	O=C(O)C1=NC=CS1	**4**{*8,8,15*}	N/A	0	0
188	O=C(NC=1C=CC(=CC1)S(=O)(=O)NC(=O)C2=CC=CO2)C3=CN(N=N3)C=4C=CC=NC4	[N-]=[N+]=NC=1C=CC=NC1	C#CC(=O)NC=1C=CC(=CC1)S(N)(=O)=O	O=C(O)C1=CC=CO1	**4**{*7,8,6*}	N/A	0	0
189	O=C(NS(=O)(=O)C=1C=CC(=CC1)C2=CN(N=N2)C=3C=CC=4N=CNC4C3)C=5C=CC=NN5	[N-]=[N+]=NC1=CC=C2N=CNC2=C1	C#CC=1C=CC(=CC1)S(N)(=O)=O	O=C(O)C=1C=CC=NN1	**4**{*29,7,36*}	N/A	0	0
190	O=C(NS(=O)(=O)C=1C=CC(=CC1)C2=CN(CC(CO)OC=3C=CC=CC3)N=N2)C=4C=CC=NN4	[N-]=[N+]=NCC(CO)OC=1C=CC=CC1	C#CC=1C=CC(=CC1)S(N)(=O)=O	O=C(O)C=1C=CC=NN1	**4**{*31,7,36*}	N/A	0	0
191	CC=1OC=NC1C(=O)NS(=O)(=O)C=2C=CC(=CC2)C3=CN(N=N3)C(CC=4C=CC=CC4)C(N)=O	[N-]=[N+]=NC(CC=1C=CC=CC1)C(N)=O	C#CC=1C=CC(=CC1)S(N)(=O)=O	CC=1OC=NC1C(=O)O	**4**{*21,7,20*}	N/A	0	0
192	COC=1C=CC(=NN1)C(=O)NS(=O)(=O)C=2C=CC(=CC2)C3=CN(CC(CO)OC=4C=CC=CC4)N=N3	[N-]=[N+]=NCC(CO)OC=1C=CC=CC1	C#CC=1C=CC(=CC1)S(N)(=O)=O	COC=1C=CC(=NN1)C(=O)O	**4**{*31,7,37*}	N/A	0	0
193	CC=1OC=NC1C(=O)NS(=O)(=O)C=2C=CC(=CC2)C3=CN(N=N3)C=4C=CC=5N=CNC5C4	[N-]=[N+]=NC1=CC=C2N=CNC2=C1	C#CC=1C=CC(=CC1)S(N)(=O)=O	CC=1OC=NC1C(=O)O	**4**{*29,7,20*}	N/A	0	0
194	O=C(NS(=O)(=O)C=1C=CC=C(C1)C2=CN(N=N2)C=3C=CC=4N=CNC4C3)C=5C=CC=NN5	[N-]=[N+]=NC1=CC=C2N=CNC2=C1	C#CC=1C=CC=C(C1)S(N)(=O)=O	O=C(O)C=1C=CC=NN1	**4**{*29,6,36*}	N/A	0	0
195	CC=1C=CC=NC1C(=O)NS(=O)(=O)C=2C=CC=C(C2)C3=CN(N=N3)C=4C=CC=CN4	[N-]=[N+]=NC=1C=CC=CN1	C#CC=1C=CC=C(C1)S(N)(=O)=O	CC=1C=CC=NC1C(=O)O	**4**{*4,6,10*}	N/A	0	0
196	O=C(CCCC1=CN(CCOCCOCCO)N=N1)NC2=NN=C(S2)S(=O)(=O)NC(=O)C3=CSC=N3	[N-]=[N+]=NCCOCCOCCO	C#CCCCC(=O)NC1=NN=C(S1)S(N)(=O)=O	O=C(O)C1=CSC=N1	**4**{*49,5,80*}	N/A	0	0
197	COC=1C=CC(=CC1OC)CN2C=C(CCCC(=O)NC3=NN=C(S3)S(=O)(=O)NC(=O)C4=NC=CS4)N=N2	COC1=CC=C(C=C1OC)CN=[N+]=[N-]	C#CCCCC(=O)NC1=NN=C(S1)S(N)(=O)=O	O=C(O)C1=NC=CS1	**4**{*1,5,15*}	N/A	0	0
198	O=C(CCCC1=CN(N=N1)C=2C=CC=CN2)NC3=NN=C(S3)S(=O)(=O)NC(=O)C4=COC=N4	[N-]=[N+]=NC=1C=CC=CN1	C#CCCCC(=O)NC1=NN=C(S1)S(N)(=O)=O	O=C(O)C1=COC=N1	**4**{*4,5,40*}	N/A	0	0
199	CC=1C=C(ON1)C(=O)NS(=O)(=O)C2=NN=C(NC(=O)CCCC3=CN(CC=4C=CC(=CC4)N5C=CC=N5)N=N3)S2	[N-]=[N+]=NCC=1C=CC(=CC1)N2C=CC=N2	C#CCCCC(=O)NC1=NN=C(S1)S(N)(=O)=O	CC=1C=C(ON1)C(=O)O	**4**{*18,5,60*}	N/A	0	0
200	CC1=NN=C(CN2C=C(CCCC(=O)NC3=NN=C(S3)S(=O)(=O)NC(=O)C=4C=NC=NC4)N=N2)O1	CC1=NN=C(CN=[N+]=[N-])O1	C#CCCCC(=O)NC1=NN=C(S1)S(N)(=O)=O	O=C(O)C=1C=NC=NC1	**4**{*36,5,56*}	N/A	0	0
201	CC1=NC=C(O1)C(=O)NS(=O)(=O)CCCCCC2=CN(N=N2)C3=CC=C4SC(C)=NC4=C3	CC1=NC2=CC(=CC=C2S1)N=[N+]=[N-]	C#CCCCCCS(N)(=O)=O	CC1=NC=C(O1)C(=O)O	**4**{*55,4,87*}	N/A	0	0
202	COC(=O)C(CN1C=C(CCCCCS(=O)(=O)NC(=O)C2COCCO2)N=N1)C(F)(F)F	COC(=O)C(CN=[N+]=[N-])C(F)(F)F	C#CCCCCCS(N)(=O)=O	O=C(O)C1COCCO1	**4**{*56,4,74*}	N/A	0	0
203	CC1CC1C(=O)NS(=O)(=O)CCCCCC2=CN(N=N2)C=3C(F)=C(F)C(F)=C(F)C3F	[N-]=[N+]=NC=1C(F)=C(F)C(F)=C(F)C1F	C#CCCCCCS(N)(=O)=O	CC1CC1C(=O)O	**4**{*57,4,84*}	N/A	0	0
204	O=C(NS(=O)(=O)CCCCCC1=CN(CC2=NO[C@@H]3[C@H]4CC[C@H](C4)[C@H]23)N=N1)[C@@H]5CC5(F)F |&1:18,19,22,24,r|	[N-]=[N+]=NCC1=NO[C@@H]2[C@H]3CC[C@H](C3)[C@H]12 |o1:7,8,11,13,r|	C#CCCCCCS(N)(=O)=O	O=C(O)[C@@H]1CC1(F)F	**4**{*58,4,42*}	N/A	0	0
205	COC(=O)C(CN1C=C(CCCCCS(=O)(=O)NC(=O)[C@@H]2COCCO2)N=N1)C(F)(F)F	COC(=O)C(CN=[N+]=[N-])C(F)(F)F	C#CCCCCCS(N)(=O)=O	O=C(O)[C@@H]1COCCO1	**4**{*56,4,59*}	N/A	0	0
206	CC1=CC=C(CN2C=C(CCCCCS(=O)(=O)NC(=O)[C@@H]3COCCO3)N=N2)O1	CC1=CC=C(CN=[N+]=[N-])O1	C#CCCCCCS(N)(=O)=O	O=C(O)[C@@H]1COCCO1	**4**{*59,4,59*}	N/A	0	0
207	NC(=O)C(CC=1C=CC=CC1)N2C=C(CCCCCS(=O)(=O)NC(=O)C3=CC=NS3)N=N2	[N-]=[N+]=NC(CC=1C=CC=CC1)C(N)=O	C#CCCCCCS(N)(=O)=O	O=C(O)C1=CC=NS1	**4**{*21,4,22*}	N/A	0	0
208	CC=1C=COC1C(=O)NS(=O)(=O)CCCCCC2=CN(CC3=NO[C@@H]4[C@H]5CC[C@H](C5)[C@H]34)N=N2 |&1:24,25,28,30,r|	[N-]=[N+]=NCC1=NO[C@@H]2[C@H]3CC[C@H](C3)[C@H]12 |o1:7,8,11,13,r|	C#CCCCCCS(N)(=O)=O	CC=1C=COC1C(=O)O	**4**{*58,4,88*}	N/A	0	0
209	O=C(NS(=O)(=O)CCCCCC1=CN(CCN2CCC(F)(F)CC2)N=N1)C3COCCO3	[N-]=[N+]=NCCN1CCC(F)(F)CC1	C#CCCCCCS(N)(=O)=O	O=C(O)C1COCCO1	**4**{*60,4,74*}	N/A	0	0
210	CN1C=CC(=N1)C(=O)NS(=O)(=O)CCCCCC2=CN(CC(F)(F)CO)N=N2	[N-]=[N+]=NCC(F)(F)CO	C#CCCCCCS(N)(=O)=O	CN1C=CC(=N1)C(=O)O	**4**{*25,4,85*}	N/A	0	0
211	COC1=CC=CC(OC)=C1CN2C=C(CCCCCS(=O)(=O)NC(=O)C3COCCO3)N=N2	COC1=CC=CC(OC)=C1CN=[N+]=[N-]	C#CCCCCCS(N)(=O)=O	O=C(O)C1COCCO1	**4**{*54,4,74*}	N/A	0	0
212	CC1=NC2=CC(=CC=C2S1)N3C=C(CCCCCS(=O)(=O)NC(=O)C=4OC=CC4C)N=N3	CC1=NC2=CC(=CC=C2S1)N=[N+]=[N-]	C#CCCCCCS(N)(=O)=O	CC=1C=COC1C(=O)O	**4**{*55,4,88*}	N/A	0	0
213	CC=1C=C(ON1)C(=O)NS(=O)(=O)CCCCCC2=CN(CC(F)(F)CO)N=N2	[N-]=[N+]=NCC(F)(F)CO	C#CCCCCCS(N)(=O)=O	CC=1C=C(ON1)C(=O)O	**4**{*25,4,60*}	N/A	0	0
214	N#CC1(CN2C=C(CCCCCS(=O)(=O)NC(=O)[C@@H]3CC3(F)F)N=N2)CCCC1	[N-]=[N+]=NCC1(C#N)CCCC1	C#CCCCCCS(N)(=O)=O	O=C(O)[C@@H]1CC1(F)F	**4**{*61,4,42*}	N/A	0	0
215	CC1CC(C1)C(=O)NS(=O)(=O)CCCCCC2=CN(N=N2)C=3C(F)=C(F)C(F)=C(F)C3F	[N-]=[N+]=NC=1C(F)=C(F)C(F)=C(F)C1F	C#CCCCCCS(N)(=O)=O	CC1CC(C1)C(=O)O	**4**{*57,4,31*}	N/A	0	0

216	CC12CC(C1)(C2)C(=O)NS(=O)(=O)CCCCC3=CN(CC4=NC5=C(Cl)N=CC=C5O4)N=N3	[N-]=[N+]=NCC1=NC2=C(Cl)N=CC=C2O1	C#CCCCCS(N)(=O)=O	CC12CC(C1)(C2)C(=O)O	**4**{*69,3,64*}	N/A	0	0
217	CC1=CON=C1C(=O)NS(=O)(=O)CCCCC2=CN(CC=3C=NC(=NC3)C4CC4)N=N2	[N-]=[N+]=NCC=1C=NC(=NC1)C2CC2	C#CCCCCS(N)(=O)=O	CC1=CON=C1C(=O)O	**4**{*64,3,65*}	N/A	0	0
218	COC(=O)C1=CC=CC(CN2C=C(CCCCS(=O)(=O)NC(=O)C3CCC(=O)N(C)C3)N=N2)=C1F	COC(=O)C1=CC=CC(CN=[N+]=[N-])=C1F	C#CCCCCS(N)(=O)=O	CN1CC(CCC1=O)C(=O)O	**4**{*65,3,91*}	N/A	0	0
219	O=C(NS(=O)(=O)CCCCC1=CN(CC2=NC=3C(Cl)=NC=CC3O2)N=N1)C4CC5CC5C4	[N-]=[N+]=NCC1=NC2=C(Cl)N=CC=C2O1	C#CCCCCS(N)(=O)=O	O=C(O)C1CC2CC2C1	**4**{*69,3,61*}	N/A	0	0
220	CC1=CON=C1C(=O)NS(=O)(=O)CCCCC2=CN(CC3=CC=C(C=N3)C(C)(F)F)N=N2	CC(F)(F)C1=CC=C(CN=[N+]=[N-])N=C1	C#CCCCCS(N)(=O)=O	CC1=CON=C1C(=O)O	**4**{*70,3,65*}	N/A	0	0
221	COC=1C=CC(=NC1)N2C=C(CCCCS(=O)(=O)NC(=O)C=3C=CSN3)N=N2	COC=1C=CC(N=[N+]=[N-])=NC1	C#CCCCCS(N)(=O)=O	O=C(O)C=1C=CSN1	**4**{*68,3,72*}	N/A	0	0
222	O=C(NS(=O)(=O)CCCCC1=CN(CC2C3CC=CCC23)N=N1)C=4C=CSN4	[N-]=[N+]=NCC1C2CC=CCC12	C#CCCCCS(N)(=O)=O	O=C(O)C=1C=CSN1	**4**{*62,3,72*}	N/A	0	0
223	O=C(NS(=O)(=O)CCCCC1=CN(CCC(F)(F)C=2C=CN=CC2)N=N1)[C@H]3COCCO3	[N-]=[N+]=NCCC(F)(F)C=1C=CN=CC1	C#CCCCCS(N)(=O)=O	O=C(O)[C@H]1COCCO1	**4**{*63,3,62*}	N/A	0	0
224	CC=1C(F)=CN=CC1C(=O)NS(=O)(=O)CCCCC2=CN(CC=3C=NC(=NC3)C4CC4)N=N2	[N-]=[N+]=NCC=1C=NC(=NC1)C2CC2	C#CCCCCS(N)(=O)=O	CC=1C(F)=CN=CC1C(=O)O	**4**{*64,3,92*}	N/A	0	0
225	O=C(NS(=O)(=O)CCCC1=CN(C[C@@H]2C[C@H]2C(F)(F)F)N=N1)C=3SC=NC3Cl |&1:13,15,r|	[N-]=[N+]=NC[C@@H]1C[C@H]1C(F)(F)F |o1:4,6,r|	C#CCCCS(N)(=O)=O	O=C(O)C=1SC=NC1Cl	**4**{*78,2,45*}	N/A	0	0
226	CC1=COC(=C1)C(=O)NS(=O)(=O)CCCC2=CN(N=N2)[C@H](C)C=3C=CC(=CC3)N4C=CN=C4	C[C@@H](N=[N+]=[N-])C=1C=CC(=CC1)N2C=CN=C2	C#CCCCS(N)(=O)=O	CC1=COC(=C1)C(=O)O	**4**{*72,2,63*}	N/A	0	0
227	COC(=O)[C@H]1CC[C@H]([C@H](O)C1)N2C=C(CCCS(=O)(=O)NC(=O)C3=NC(Cl)=CS3)N=N2 |&1:4,7,8,r|	COC(=O)[C@@H]1CC[C@H](N=[N+]=[N-])[C@@H](O)C1 |o1:4,7,11,r|	C#CCCCS(N)(=O)=O	O=C(O)C1=NC(Cl)=CS1	**4**{*79,2,25*}	N/A	0	0
228	COC(=O)C12CCC(CN3C=C(CCCS(=O)(=O)NC(=O)[C@@H]4C[C@H]4C(C)C)N=N3)(CC1)C2 |&1:21,23,r|	COC(=O)C12CCC(CN=[N+]=[N-])(CC1)C2	C#CCCCS(N)(=O)=O	CC(C)[C@@H]1C[C@H]1C(=O)O |o1:3,5,r|	**4**{*80,2,57*}	N/A	0	0
229	CN1N=CC(=N1)C(=O)NS(=O)(=O)CCCC2=CN(N=N2)[C@@H]3COC4(CCOCC4)[C@H]3O |&1:20,29,r|	[N-]=[N+]=N[C@H]1COC2(CCOCC2)[C@@H]1O |o1:3,12,r|	C#CCCCS(N)(=O)=O	CN1N=CC(=N1)C(=O)O	**4**{*81,2,76*}	N/A	0	0
230	CC1=CON=C1C(=O)NS(=O)(=O)CCCC2=CN(CC=3C=CC(=CN3)C(C)(F)F)N=N2	CC(F)(F)C1=CC=C(CN=[N+]=[N-])N=C1	C#CCCCS(N)(=O)=O	CC1=CON=C1C(=O)O	**4**{*70,2,65*}	N/A	0	0
231	COC(=O)[C@H]1CC[C@H]([C@H](O)C1)N2C=C(CCCS(=O)(=O)NC(=O)C3CC3Cl)N=N2 |&1:4,7,8,r|	COC(=O)[C@@H]1CC[C@H](N=[N+]=[N-])[C@@H](O)C1 |o1:4,7,11,r|	C#CCCCS(N)(=O)=O	O=C(O)C1CC1Cl	**4**{*79,2,23*}	N/A	0	0
232	O=C(NS(=O)(=O)CCCC1=CN(N=N1)C2CCC=3C=CC=NC32)C4=CSN=C4Cl	Cl.[N-]=[N+]=NC1CCC2=CC=CN=C21	C#CCCCS(N)(=O)=O	O=C(O)C1=CSN=C1Cl	**4**{*82,2,58*}	N/A	0	0
233	CC(CC(=O)NS(=O)(=O)CCCC1=CN(C[C@@H]2C[C@H]2C(F)(F)F)N=N1)C(F)F |&1:16,18,r|	[N-]=[N+]=NC[C@@H]1C[C@H]1C(F)(F)F |o1:4,6,r|	C#CCCCS(N)(=O)=O	CC(CC(=O)O)C(F)F	**4**{*78,2,69*}	N/A	0	0
234	CCOC(=O)C1CCC(=CN2C=C(CCCS(=O)(=O)NC(=O)C3=NC(Cl)=CS3)N=N2)CC1	CCOC(=O)C1CCC(=CN=[N+]=[N-])CC1	C#CCCCS(N)(=O)=O	O=C(O)C1=NC(Cl)=CS1	**4**{*73,2,25*}	N/A	0	0
235	COC(=O)C12CCC(CN3C=C(CCCS(=O)(=O)NC(=O)C4=CSN=C4Cl)N=N3)(CC1)C2	COC(=O)C12CCC(CN=[N+]=[N-])(CC1)C2	C#CCCCS(N)(=O)=O	O=C(O)C1=CSN=C1Cl	**4**{*80,2,58*}	N/A	0	0
236	COC(=O)C12CCC(CN3C=C(CCCS(=O)(=O)NC(=O)C4=NC(Cl)=CS4)N=N3)(CC1)C2	COC(=O)C12CCC(CN=[N+]=[N-])(CC1)C2	C#CCCCS(N)(=O)=O	O=C(O)C1=NC(Cl)=CS1	**4**{*80,2,25*}	N/A	0	0
237	CN1CCC(CC(=O)NS(=O)(=O)CCCC2=CN(N=N2)C3CCC=4C=CC=NC43)C1=O	Cl.[N-]=[N+]=NC1CCC2=CC=CN=C21	C#CCCCS(N)(=O)=O	CN1CCC(CC(=O)O)C1=O	**4**{*82,2,24*}	N/A	0	0
238	O=C(NS(=O)(=O)CCC1=CN(N=N1)[C@@H]2COC3(CCOCC3)[C@H]2O)C4=CC(Cl)=NS4 |&1:13,22,r|	[N-]=[N+]=N[C@H]1COC2(CCOCC2)[C@@H]1O |o1:3,12,r|	C#CCCS(N)(=O)=O	O=C(O)C1=CC(Cl)=NS1	**4**{*81,1,39*}	N/A	0	0
239	COC=1C=NN=C(C1)C(=O)NS(=O)(=O)CCC2=CN(CC=3C=NN(C)C3)N=N2	CN1C=C(C=N1)CN=[N+]=[N-]	C#CCCS(N)(=O)=O	COC=1C=NN=C(C1)C(=O)O	**4**{*34,1,95*}	N/A	0	0
240	CC(F)(F)C=1C=NC=C(C1)CN2C=C(CCS(=O)(=O)NC(=O)C3=CC(Cl)=NS3)N=N2	CC(F)(F)C1=CN=CC(=C1)CN=[N+]=[N-]	C#CCCS(N)(=O)=O	O=C(O)C1=CC(Cl)=NS1	**4**{*83,1,39*}	N/A	0	0
241	COC(=O)[C@H]1CC[C@H]([C@H](O)C1)N2C=C(CCS(=O)(=O)NC(=O)C3=CC(Cl)=NS3)N=N2 |&1:4,7,8,r|	COC(=O)[C@@H]1CC[C@H](N=[N+]=[N-])[C@@H](O)C1 |o1:4,7,11,r|	C#CCCS(N)(=O)=O	O=C(O)C1=CC(Cl)=NS1	**4**{*79,1,39*}	N/A	0	0
242	COC(=O)[C@H]1CC[C@H]([C@H](O)C1)N2C=C(CCS(=O)(=O)NC(=O)C=3C=NN(C)N3)N=N2 |&1:4,7,8,r|	COC(=O)[C@@H]1CC[C@H](N=[N+]=[N-])[C@@H](O)C1 |o1:4,7,11,r|	C#CCCS(N)(=O)=O	CN1N=CC(=N1)C(=O)O	**4**{*79,1,76*}	N/A	0	0
243	CC(F)(F)C=1C=CC(CN2C=C(CCS(=O)(=O)NC(=O)C3=CSN=C3Cl)N=N2)=NC1	CC(F)(F)C1=CC=C(CN=[N+]=[N-])N=C1	C#CCCS(N)(=O)=O	O=C(O)C1=CSN=C1Cl	**4**{*70,1,58*}	N/A	0	0
244	O=C(NS(=O)(=O)CCC1=CN(CC2=NO[C@@H]3[C@H]4CC[C@H](C4)[C@H]23)N=N1)C5CC6CC6C5 |&1:15,16,19,21,r|	[N-]=[N+]=NCC1=NO[C@@H]2[C@H]3CC[C@H](C3)[C@H]12 |o1:7,8,11,13,r|	C#CCCS(N)(=O)=O	O=C(O)C1CC2CC2C1	**4**{*58,1,61*}	N/A	0	0
245	CC1(F)CC(C1)C(=O)NS(=O)(=O)CCC2=CN(CCN3CCC(F)(F)CC3)N=N2	[N-]=[N+]=NCCN1CCC(F)(F)CC1	C#CCCS(N)(=O)=O	CC1(F)CC(C1)C(=O)O	**4**{*60,1,46*}	N/A	0	0
246	O=C(NS(=O)(=O)CCC1=CN(C[C@@H]2C[C@H]2C(F)(F)F)N=N1)C3=NC(Cl)=CS3 |&1:12,14,r|	[N-]=[N+]=NC[C@@H]1C[C@H]1C(F)(F)F |o1:4,6,r|	C#CCCS(N)(=O)=O	O=C(O)C1=NC(Cl)=CS1	**4**{*78,1,25*}	N/A	0	0
247	O=C(NS(=O)(=O)CCC1=CN(CCC(F)(F)C=2C=CN=CC2)N=N1)C=3SC=NC3Cl	[N-]=[N+]=NCCC(F)(F)C=1C=CN=CC1	C#CCCS(N)(=O)=O	O=C(O)C=1SC=NC1Cl	**4**{*63,1,45*}	N/A	0	0

Library 7

#	Product SMILES	Reactant 5 SMILES	Reactant 6 SMILES	Reactant 3 SMILES	*Product ID*	LC/MS *m/z*	Yield, mg	Yield, %
248	CC(CC(F)F)C(=O)NS(=O)(=O)C=1C=CC(=CC1)N2C=C(N=N2)C=3C=CC(Br)=CN3	[N-]=[N+]=NC=1C=CC(=CC1)S(N)(=O)=O	C#CC=1C=CC(Br)=CN1	CC(CC(F)F)C(=O)O	**7**{*3,11,38*}	500/502 as 1/1 [M+H]$^{+}$	99.8	100
249	CC(F)(F)CCC(=O)NS(=O)(=O)C=1C=CC=C(C1)N2C=C(N=N2)C3=NC=4C=CC=CC4S3	[N-]=[N+]=NC=1C=CC=C(C1)S(N)(=O)=O	C#CC1=NC=2C=CC=CC2S1	CC(F)(F)CCC(=O)O	**7**{*2,37,73*}	478 [M+H]$^{+}$	99.8	100
250	CC=1C=CN=CC1C(=O)NS(=O)(=O)C=2C=CC(=CC2)CN3C=C(CNC(=O)C=4C=CC=C(C#N)C4)N=N3	[N-]=[N+]=NCC=1C=CC(=CC1)S(N)(=O)=O	C#CCNC(=O)C1=CC=CC(C#N)=C1	CC=1C=CN=CC1C(=O)O	**7**{*1,47,54*}	516 [M+H]$^{+}$	99.8	100
251	O=C(NS(=O)(=O)C=1C=CC=C(C1)N2C=C(N=N2)C3=CC(Cl)=NC=C3C(F)(F)F)C=4C=NSC4	[N-]=[N+]=NC=1C=CC=C(C1)S(N)(=O)=O	C#CC=1C=C(Cl)N=CC1C(F)(F)F	O=C(O)C=1C=NSC1	**7**{*2,7,27*}	515/517 as 3/1 [M+H]$^{+}$	99.3	99
252	CCCC(=O)NS(=O)(=O)C=1C=CC(=CC1)CN2C=C(CNC(=O)NC=3C=CC=CC3[N+]([O-])=O)N=N2	[N-]=[N+]=NCC=1C=CC(=CC1)S(N)(=O)=O	C#CCNC(=O)NC=1C=CC=CC1[N+]([O-])=O	CCCC(=O)O	**7**{*1,48,4*}	502 [M+H]$^{+}$	98.4	98
253	CC1=CC(=CO1)C(=O)NS(=O)(=O)C=2C=CC(=CC2)N3C=C(N=N3)C4=CSC(=N4)C(F)(F)F	[N-]=[N+]=NC=1C=CC(=CC1)S(N)(=O)=O	C#CC1=CSC(=N1)C(F)(F)F	CC1=CC(=CO1)C(=O)O	**7**{*3,36,66*}	484 [M+H]$^{+}$	97.6	98
254	CC=1N=CC=CC1C(=O)NS(=O)(=O)C=2C=CC(=CC2)CN3C=C(N=N3)C=4C=CC=C(C4)C=5C=CC=CC5	[N-]=[N+]=NCC=1C=CC(=CC1)S(N)(=O)=O	C#CC=1C=CC=C(C1)C=2C=CC=CC2	CC=1N=CC=CC1C(=O)O	**7**{*1,16,12*}	510 [M+H]$^{+}$	96.8	97
255	O=C(NS(=O)(=O)C=1C=CC=C(C1)N2C=C(N=N2)C34CCC(CC3)(CC4)C=5C=CC=CC5)C6COCCO6	[N-]=[N+]=NC=1C=CC=C(C1)S(N)(=O)=O	C#CC12CCC(CC1)(CC2)C=3C=CC=CC3	O=C(O)C1COCCO1	**7**{*2,38,74*}	523 [M+H]$^{+}$	96.8	97
256	CC(C)[C@@H]1C[C@H]1C(=O)NS(=O)(=O)C=2C=CC(=CC2)N3C=C(N=N3)C=4C=CC=5C=C(Cl)C=CC5C4 |&1:3,5,r|	[N-]=[N+]=NC=1C=CC(=CC1)S(N)(=O)=O	C#CC=1C=CC2=CC(Cl)=CC=C2C1	CC(C)[C@@H]1C[C@H]1C(=O)O |o1:3,5,r|	**7**{*3,19,57*}	495/497 as 3/1 [M+H]$^{+}$	95.5	96
257	O=C(NS(=O)(=O)C=1C=CC=C(C1)N2C=C(N=N2)C=3C=CC=CC3C=4C=CC=CC4)C=5C=CN=CC5	[N-]=[N+]=NC=1C=CC=C(C1)S(N)(=O)=O	C#CC=1C=CC=CC1C=2C=CC=CC2	O=C(O)C=1C=CN=CC1	**7**{*2,18,50*}	482 [M+H]$^{+}$	93.6	94
258	O=C(CCC1CCC1)NS(=O)(=O)C=2C=CC=C(C2)N3C=C(N=N3)C4CCCC(F)(F)C4	[N-]=[N+]=NC=1C=CC=C(C1)S(N)(=O)=O	C#CC1CCCC(F)(F)C1	O=C(O)CCC1CCC1	**7**{*2,40,75*}	453 [M+H]$^{+}$	91.8	92
259	CN1CCC(CC(=O)NS(=O)(=O)C=2C=CC(=CC2)N3C=C(N=N3)C=4C=CC=5C=C(Cl)C=CC5C4)C1=O	[N-]=[N+]=NC=1C=CC(=CC1)S(N)(=O)=O	C#CC=1C=CC2=CC(Cl)=CC=C2C1	CN1CCC(CC(=O)O)C1=O	**7**{*3,19,24*}	524/526 as 3/1 [M+H]$^{+}$	91.4	91
260	CC(C)(C)C=1C=CC=CC1CCC2=CN(N=N2)C=3C=CC(=CC3)S(=O)(=O)NC(=O)C4=CC(Cl)=NS4	[N-]=[N+]=NC=1C=CC(=CC1)S(N)(=O)=O	C#CCCC=1C=CC=CC1C(C)(C)C	O=C(O)C1=CC(Cl)=NS1	**7**{*3,43,39*}	530/532 as 3/1 [M+H]$^{+}$	91.4	91
261	CCCC(=O)NS(=O)(=O)C=1C=CC(=CC1)CN2C=C(CN3N=C(Br)C=CC3=O)N=N2	[N-]=[N+]=NCC=1C=CC(=CC1)S(N)(=O)=O	C#CCN1N=C(Br)C=CC1=O	CCCC(=O)O	**7**{*1,46,4*}	495/497 as 1/1 [M+H]$^{+}$	90.	90
262	O=C(NS(=O)(=O)C=1C=CC=C(C1)N2C=C(N=N2)C3=CC(Cl)=NC=C3C(F)(F)F)C4CC4F	[N-]=[N+]=NC=1C=CC=C(C1)S(N)(=O)=O	C#CC=1C=C(Cl)N=CC1C(F)(F)F	O=C(O)C1CC1F	**7**{*2,7,26*}	490/492 as 3/1 [M+H]$^{+}$	87.9	88
263	CC=1C=CC(=CN1)C(=O)NS(=O)(=O)C=2C=CC(=CC2)CN3C=C(N=N3)C=4C=CN=C5C=CC=CC45	[N-]=[N+]=NCC=1C=CC(=CC1)S(N)(=O)=O	C#CC=1C=CN=C2C=CC=CC12	CC=1C=CC(=CN1)C(=O)O	**7**{*1,22,13*}	485 [M+H]$^{+}$	87.3	87

264	O=C(NS(=O)(=O)C=1C=CC=C(C1)N2C=C(N=N2)C=3C=C(C=CC3F)C(F)(F)F)C=4C=CC=CN4	[N-]=[N+]=NC=1C=CC=C(C1)S(N)(=O)=O	C#CC=1C=C(C=CC1F)C(F)(F)F	O=C(O)C=1C=CC=CN1	**7**{*2,2,18*}	492 $[M+H]^+$	85.6	86
265	O=C(NS(=O)(=O)C=1C=CC(=CC1)CN2C=C(N=N2)C3=CC=C(Br)N=C3)C4=COC=N4	[N-]=[N+]=NCC=1C=CC(=CC1)S(N)(=O)=O	C#CC=1C=CC(Br)=NC1	O=C(O)C1=COC=N1	**7**{*1,12,40*}	489/491 as 1/1 $[M+H]^+$	85.1	85
266	CC1(CC(=O)NS(=O)(=O)C=2C=CC(=CC2)N3C=C(N=N3)C4=CC=C5SC=NC5=C4)CCC1	[N-]=[N+]=NC=1C=CC(=CC1)S(N)(=O)=O	C#CC=1C=CC=2SC=NC2C1	CC1(CC(=O)O)CCC1	**7**{*3,15,44*}	468 $[M+H]^+$	84.6	85
267	CC1(CC(=O)NS(=O)(=O)C=2C=CC(=CC2)N3C=C(N=N3)C4=NC5=CC=CC=C5S4)CC1	[N-]=[N+]=NC=1C=CC(=CC1)S(N)(=O)=O	C#CC1=NC=2C=CC=CC2S1	CC1(CC(=O)O)CC1	**7**{*3,37,71*}	454 $[M+H]^+$	83.7	84
268	O=C(O)C(F)(F)F.COC(=O)C=1C=CC(=CC1)CN(C)CC2=CN(N=N2)C=3C=CC=C(C3)S(=O)(=O)NC(=O)C=4 C=CC=CN4	[N-]=[N+]=NC=1C=CC=C(C1)S(N)(=O)=O	Cl.C#CCN(C)CC=1C=CC(=CC1)C(=O)OC	O=C(O)C=1C=CC=CN1	**7**{*2,50,18*}	521 [M–TFA+H]$^+$	83.3	83
269	O=C(NS(=O)(=O)C=1C=CC(=CC1)N2C=C(N=N2)C=3C=CC=4C=C(Cl)C=CC4C3)C5=CSN=C5Cl	[N-]=[N+]=NC=1C=CC(=CC1)S(N)(=O)=O	C#CC=1C=CC2=CC(Cl)=CC=C2C1	O=C(O)C1=CSN=C1Cl	**7**{*3,19,58*}	530/532/534 as 9/6/1 $[M+H]^+$	80.6	81
270	COCC(=O)NS(=O)(=O)C=1C=CC=C(C1)N2C=C(N=N2)C3=CC(=CC=C3F)C(F)(F)F	[N-]=[N+]=NC=1C=CC=C(C1)S(N)(=O)=O	C#CC=1C=C(C=CC1F)C(F)(F)F	COCC(=O)O	**7**{*2,2,19*}	459 $[M+H]^+$	79.6	80
271	CS(=O)(=O)C=1C=CC(=CC1)C2=CN(CC=3C=CC(=CC3)S(=O)(=O)NC(=O)C=4C=CC(F)=CC4)N=N2	[N-]=[N+]=NCC=1C=CC(=CC1)S(N)(=O)=O	C#CC=1C=CC(=CC1)S(C)(=O)=O	O=C(O)C=1C=CC(F)=CC1	**7**{*1,10,14*}	515 $[M+H]^+$	77.9	78
272	CCC1(C)CC1C(=O)NS(=O)(=O)C=2C=CC(=CC2)N3C=C(N=N3)C=4C=CC=C(Cl)C4Br	[N-]=[N+]=NC=1C=CC(=CC1)S(N)(=O)=O	C#CC1=CC=CC(Cl)=C1Br	CCC1(C)CC1C(=O)O	**7**{*3,34,70*}	523/525/527 as 3/4/1 $[M+H]^+$	76.5	77
273	CC(C)C(=O)NS(=O)(=O)C=1C=CC(=CC1)CN2C=C(N=N2)C=3C=CC=4N=CC=CC4C3	[N-]=[N+]=NCC=1C=CC(=CC1)S(N)(=O)=O	C#CC1=CC=C2N=CC=CC2=C1	CC(C)C(=O)O	**7**{*1,30,68*}	436 $[M+H]^+$	75.6	76
274	CC1=NC(=CO1)C(=O)NS(=O)(=O)C=2C=CC=C(C2)N3C=C(N=N3)C4CCC(F)(F)CC4	[N-]=[N+]=NC=1C=CC=C(C1)S(N)(=O)=O	C#CC1CCC(F)(F)CC1	CC1=NC(=CO1)C(=O)O	**7**{*2,39,33*}	452 $[M+H]^+$	75.2	75
275	CS(=O)(=O)C=1C=CC(=CC1)C2=CN(CC=3C=CC(=CC3)S(=O)(=O)NC(=O)C4CC4(F)F)N=N2	[N-]=[N+]=NCC=1C=CC(=CC1)S(N)(=O)=O	C#CC=1C=CC(=CC1)S(C)(=O)=O	O=C(O)C1CC1(F)F	**7**{*1,10,11*}	497 $[M+H]^+$	74.3	74
276	CC(=O)C=1C=C(C=CC1Cl)C2=CN(N=N2)C=3C=CC=C(C3)S(=O)(=O)NC(=O)[C@@H]4CC4(F)F	[N-]=[N+]=NC=1C=CC=C(C1)S(N)(=O)=O	C#CC=1C=CC(Cl)=C(C1)C(C)=O	O=C(O)[C@@H]1CC1(F)F	**7**{*2,13,42*}	481/483 as 3/1 $[M+H]^+$	74.3	74
277	O=C(NS(=O)(=O)C=1C=CC(=CC1)CN2C=C(N=N2)C=3C=NC(Br)=CC3)C=4C=CC=C(F)C4	[N-]=[N+]=NCC=1C=CC(=CC1)S(N)(=O)=O	C#CC=1C=CC(Br)=NC1	O=C(O)C=1C=CC=C(F)C1	**7**{*1,12,41*}	516/518 as 1/1 $[M+H]^+$	74.3	74
278	CN1C=C2C=CC(=CC2=N1)C3=CN(N=N3)C=4C=CC=C(C4)S(=O)(=O)NC(=O)[C@@H]5COCCO5	[N-]=[N+]=NC=1C=CC=C(C1)S(N)(=O)=O	C#CC=1C=CC2=CN(C)N=C2C1	O=C(O)[C@@H]1COCCO1	**7**{*2,20,59*}	469 $[M+H]^+$	73.4	73
279	O=C(NS(=O)(=O)C=1C=CC(=CC1)N2C=C(N=N2)C=3C=CN=CC3Br)C4CC5CC5C4	[N-]=[N+]=NC=1C=CC(=CC1)S(N)(=O)=O	C#CC=1C=CN=CC1Br	O=C(O)C1CC2CC2C1	**7**{*3,23,61*}	488/490 as 1/1 $[M+H]^+$	70.7	71
280	O=C(NS(=O)(=O)C=1C=CC=C(C1)N2C=C(CCC3=CC(F)=CC=C3F)N=N2)C4CC4F	[N-]=[N+]=NC=1C=CC=C(C1)S(N)(=O)=O	C#CCCC=1C=C(F)C=CC1F	O=C(O)C1CC1F	**7**{*2,42,26*}	451 $[M+H]^+$	70.7	71
281	CCOCC(=O)NS(=O)(=O)C=1C=CC(=CC1)CN2C=C(N=N2)C=3C=CC(=CC3)S(C)(=O)=O	[N-]=[N+]=NCC=1C=CC(=CC1)S(N)(=O)=O	C#CC=1C=CC(=CC1)S(C)(=O)=O	CCOCC(=O)O	**7**{*1,10,1*}	479 $[M+H]^+$	69.3	69
282	CCCC(=O)NS(=O)(=O)C=1C=CC(=CC1)CN2C=C(CNC(=O)C=3C=CC=C(C#N)C3)N=N2	[N-]=[N+]=NCC=1C=CC(=CC1)S(N)(=O)=O	C#CCNC(=O)C1=CC=CC(C#N)=C1	CCCC(=O)O	**7**{*1,47,4*}	467 $[M+H]^+$	69.3	69
283	O=C(NS(=O)(=O)C=1C=CC(=CC1)CN2C=C(N=N2)C3=CC=CC(=C3)C=4C=CC=CC4)C5CCCC5	[N-]=[N+]=NCC=1C=CC(=CC1)S(N)(=O)=O	C#CC=1C=CC=C(C1)C=2C=CC=CC2	O=C(O)C1CCCC1	**7**{*1,16,5*}	487 $[M+H]^+$	68.4	68
284	CC=1C=CC=C(C1)C(=O)NS(=O)(=O)C=2C=CC(=CC2)CN3C=C(N=N3)C=4C=CC(=CC4)S(C)(=O)=O	[N-]=[N+]=NCC=1C=CC(=CC1)S(N)(=O)=O	C#CC=1C=CC(=CC1)S(C)(=O)=O	CC=1C=CC=C(C1)C(=O)O	**7**{*1,10,3*}	511 $[M+H]^+$	67.5	68
285	CC=1C=NC=C(N1)C(=O)NS(=O)(=O)C=2C=CC(=CC2)CN3C=C(N=N3)C=4C=CC(=CC4)S(C)(=O)=O	[N-]=[N+]=NCC=1C=CC(=CC1)S(N)(=O)=O	C#CC=1C=CC(=CC1)S(C)(=O)=O	CC=1C=NC=C(N1)C(=O)O	**7**{*1,10,30*}	513 $[M+H]^+$	65.3	65
286	CC=1C=CC=NC1C(=O)NS(=O)(=O)C=2C=CC(=CC2)CN3C=C(N=N3)C=4C=CN=C5C=CC=CC45	[N-]=[N+]=NCC=1C=CC(=CC1)S(N)(=O)=O	C#CC=1C=CN=C2C=CC=CC12	CC=1C=CC=NC1C(=O)O	**7**{*1,22,10*}	485 $[M+H]^+$	64.4	64
287	O=C(NS(=O)(=O)C=1C=CC(=CC1)N2C=C(N=N2)C3=CC(Cl)=NC(=C3)C(F)(F)F)C4CC4Cl	[N-]=[N+]=NC=1C=CC(=CC1)S(N)(=O)=O	C#CC=1C=C(Cl)N=C(C1)C(F)(F)F	O=C(O)C1CC1Cl	**7**{*3,6,23*}	506/508/510 as 9/6/1 $[M+H]^+$	63.5	64
288	CN1N=CC(=N1)C(=O)NS(=O)(=O)C=2C=CC(=CC2)N3C=C(CCC=4C=CC=CC4C(C)(C)C)N=N3	[N-]=[N+]=NC=1C=CC(=CC1)S(N)(=O)=O	C#CCCC=1C=CC=CC1C(C)(C)C	CN1N=CC(=N1)C(=O)O	**7**{*3,43,76*}	494 $[M+H]^+$	61.7	62
289	CC=1C=CC=NC1C(=O)NS(=O)(=O)C=2C=CC(=CC2)CN3C=C(N=N3)C=4C=CC=5C=CC=CC5N4	[N-]=[N+]=NCC=1C=CC(=CC1)S(N)(=O)=O	C#CC=1C=CC=2C=CC=CC2N1	CC=1C=CC=NC1C(=O)O	**7**{*1,14,10*}	485 $[M+H]^+$	60.3	60
290	CC=1C(=CC=CC1C(F)(F)F)C2=CN(N=N2)C=3C=CC(=CC3)S(=O)(=O)NC(=O)CC(C)C(F)F	[N-]=[N+]=NC=1C=CC(=CC1)S(N)(=O)=O	C#CC1=CC=CC(=C1C)C(F)(F)F	CC(CC(=O)O)C(F)F	**7**{*3,32,69*}	503 $[M+H]^+$	59	59
291	CC1=COC=C1C(=O)NS(=O)(=O)C=2C=CC(=CC2)N3C=C(N=N3)C=4C=CC=5C=NN(C)C5C4	[N-]=[N+]=NC=1C=CC(=CC1)S(N)(=O)=O	C#CC1=CC=C2C=NN(C)C2=C1	CC1=COC=C1C(=O)O	**7**{*3,29,67*}	463 $[M+H]^+$	56.7	57
292	CCCC(=O)NS(=O)(=O)C=1C=CC(=CC1)CN2C=C(N=N2)C3=CN=CC(Br)=C3	[N-]=[N+]=NCC=1C=CC(=CC1)S(N)(=O)=O	C#CC1=CN=CC(Br)=C1	CCCC(=O)O	**7**{*1,35,4*}	464/466 as 1/1 $[M+H]^+$	56.7	57
293	CC=1C=C(ON1)C(=O)NS(=O)(=O)C=2C=CC=C(C2)N3C=C(N=N3)C=4C=CC5=NON=C5C4	[N-]=[N+]=NC=1C=CC=C(C1)S(N)(=O)=O	C#CC=1C=CC2=NON=C2C1	CC=1C=C(ON1)C(=O)O	**7**{*2,21,60*}	452 $[M+H]^+$	56.3	56
294	COC(=O)C1=CC(=CC(=C1)C2=CN(CC=3C=CC(=CC3)S(=O)(=O)NC(=O)C4CC(F)(F)C4)N=N2)C(=O)OC	[N-]=[N+]=NCC=1C=CC(=CC1)S(N)(=O)=O	C#CC1=CC(=CC(=C1)C(=O)OC)C(=O)OC	O=C(O)C1CC(F)(F)C1	**7**{*1,27,9*}	449 $[M+H]^+$	55.4	55
295	CC1=CC=C(O1)C(=O)NS(=O)(=O)C=2C=CC(=CC2)CN3C=C(N=N3)C=4C=CC=5C=CC=CC5N4	[N-]=[N+]=NCC=1C=CC(=CC1)S(N)(=O)=O	C#CC=1C=CC=2C=CC=CC2N1	CC1=CC=C(O1)C(=O)O	**7**{*1,14,43*}	474 $[M+H]^+$	47.7	48
296	O=C(NS(=O)(=O)C=1C=CC=C(C1)N2C=C(N=N2)C3=NC=4C=CC=CC4S3)C=5C=CSN5	[N-]=[N+]=NC=1C=CC=C(C1)S(N)(=O)=O	C#CC1=NC=2C=CC=CC2S1	O=C(O)C=1C=CSN1	**7**{*2,37,72*}	469 $[M+H]^+$	46.4	46
297	CCC(CC)C(=O)NS(=O)(=O)C=1C=CC(=CC1)CN2C=C(N=N2)C=3C=CC=C(C3)C=4C=CC=CC4	[N-]=[N+]=NCC=1C=CC(=CC1)S(N)(=O)=O	C#CC=1C=CC=C(C1)C=2C=CC=CC2	CCC(CC)C(=O)O	**7**{*1,16,7*}	489 $[M+H]^+$	45	45
298	CC1=COC(=C1)C(=O)NS(=O)(=O)C=2C=CC(=CC2)N3C=C(N=N3)C4=CSC(=N4)C(F)(F)F	[N-]=[N+]=NC=1C=CC(=CC1)S(N)(=O)=O	C#CC1=CSC(=N1)C(F)(F)F	CC1=COC(=C1)C(=O)O	**7**{*3,36,63*}	484 $[M+H]^+$	42.3	42
299	CC=1C=C(C=CN1)C(=O)NS(=O)(=O)C=2C=CC=C(C2)N3C=C(COC=4N=CC(Br)=CN4)N=N3	[N-]=[N+]=NC=1C=CC=C(C1)S(N)(=O)=O	C#CCOC=1N=CC(Br)=CN1	CC=1C=C(C=CN1)C(=O)O	**7**{*2,49,28*}	530/532 as 1/1 $[M+H]^+$	41	41
300	O=C(NS(=O)(=O)C=1C=CC(=CC1)CN2C=C(N=N2)C=3C(Cl)=CC=CC3Br)C4CC(F)(F)C4	[N-]=[N+]=NCC=1C=CC(=CC1)S(N)(=O)=O	C#CC1=C(Cl)C=CC=C1Br	O=C(O)C1CC(F)(F)C1	**7**{*1,26,9*}	545/547/549 as 3/4/1 $[M+H]^+$	40.5	41
301	CC=1OC=NC1C(=O)NS(=O)(=O)C=2C=CC=C(C2)N3C=C(N=N3)C4CCC(F)(F)CC4	[N-]=[N+]=NC=1C=CC=C(C1)S(N)(=O)=O	C#CC1CCC(F)(F)CC1	CC=1OC=NC1C(=O)O	**7**{*2,39,20*}	452 $[M+H]^+$	40.5	41
302	CC=1C=CC=C(N1)C(=O)NS(=O)(=O)C=2C=CC(=CC2)CN3C=C(N=N3)C=4C=CC=C5C=CC=NC45	[N-]=[N+]=NCC=1C=CC(=CC1)S(N)(=O)=O	C#CC=1C=CC=C2C=CC=NC12	CC=1C=CC=C(N1)C(=O)O	**7**{*1,17,55*}	485 $[M+H]^+$	40.1	40
303	CCOC(=O)COC1(COC1)C2=CN(N=N2)C=3C=CC(=CC3)S(=O)(=O)NC(=O)CC4(C)CCC4	[N-]=[N+]=NC=1C=CC(=CC1)S(N)(=O)=O	C#CC1(COC1)OCC(=O)OCC	CC1(CC(=O)O)CCC1	**7**{*3,25,44*}	493 $[M+H]^+$	39.6	40
304	CC=1OC=NC1C(=O)NS(=O)(=O)C=2C=CC=C(C2)N3C=C(N=N3)C=4C=C(Cl)C(Cl)=C(Cl)C4	[N-]=[N+]=NC=1C=CC=C(C1)S(N)(=O)=O	C#CC=1C=C(Cl)C(Cl)=C(Cl)C1	CC=1OC=NC1C(=O)O	**7**{*2,3,20*}	507/509/511 /513 as 27/27/9/1 $[M+H]^+$	39.2	39
305	O=C(NS(=O)(=O)C=1C=CC(=CC1)N2C=C(N=N2)C=3C=NC=4C=CSC4C3)C5=CSN=C5Cl	[N-]=[N+]=NC=1C=CC(=CC1)S(N)(=O)=O	C#CC=1C=NC=2C=CSC2C1	O=C(O)C1=CSN=C1Cl	**7**{*3,24,58*}	503/505 as 3/1 $[M+H]^+$	38.4	38
306	COC(=O)C=1C=C(C=C(C1)C2=CN(CC=3C=CC(=CC3)S(=O)(=O)NC(=O)CC4CC4)N=N2)C(=O)OC	[N-]=[N+]=NCC=1C=CC(=CC1)S(N)(=O)=O	C#CC1=CC(=CC(=C1)C(=O)OC)C(=O)OC	O=C(O)CC1CC1	**7**{*1,27,8*}	513 $[M+H]^+$	37.9	38
307	CC1=CON=C1C(=O)NS(=O)(=O)C=2C=CC(=CC2)N3C=C(N=N3)C=4C=CC=5C=NN(C)C5C4	[N-]=[N+]=NC=1C=CC(=CC1)S(N)(=O)=O	C#CC1=CC=C2C=NN(C)C2=C1	CC1=CON=C1C(=O)O	**7**{*3,29,65*}	464 $[M+H]^+$	36.6	37
308	CC(C)(C)C=1C=CC=CC1CCC2=CN(N=N2)C=3C=CC(=CC3)S(=O)(=O)NC(=O)C4CC4Cl	[N-]=[N+]=NC=1C=CC(=CC1)S(N)(=O)=O	C#CCCC=1C=CC=CC1C(C)(C)C	O=C(O)C1CC1Cl	**7**{*3,43,23*}	487/489 as 3/1 $[M+H]^+$	35.2	35
309	O=C(O)C(F)(F)F.O=C(NS(=O)(=O)C=1C=CC=C(C1)N2C=C(CN3CCC=4C=5C=CC=CC5NC4C3)N=N2)C=6 C=CC=NN6	[N-]=[N+]=NC=1C=CC=C(C1)S(N)(=O)=O	C#CCN1CCC=2C3=CC=CC=C3NC2C1	O=C(O)C=1C=CC=NN1	**7**{*2,45,36*}	515 [M-TFA+H]$^+$	33.3	33
310	O=C(NS(=O)(=O)C=1C=CC(=CC1)N2C=C(N=N2)C=3C=CN=CC3Br)[C@H]4COCCO4	[N-]=[N+]=NC=1C=CC(=CC1)S(N)(=O)=O	C#CC=1C=CN=CC1Br	O=C(O)[C@H]1COCCO1	**7**{*3,23,62*}	494/496 as 1/1 $[M+H]^+$	31.1	31

311	O=C(NS(=O)(=O)C=1C=CC=C(C1)N2C=C(CCC3=CC=CC(F)=C3F)N=N2)C=4C=CC=C5CCCC45	[N-]=[N+]=NC=1C=CC=C(C1)S(N)(=O)=O	C#CCCC1=CC=CC(F)=C1F	O=C(O)C1=CC=CC=2CCCC 12	**7**{*2,44,77*}	509 $[M+H]^+$	31.1	31
312	CC=1C=CC(=CC1C2=CN(N=N2)C=3C=CC=C(C3)S(=O)(=O)NC(=O)C=4C=CC=NC4)C(F)(F)F	[N-]=[N+]=NC=1C=CC=C(C1)S(N)(=O)=O	C#CC=1C=C(C=CC1C)C(F)(F)F	O=C(O)C=1C=CC=NC1	**7**{*2,1,17*}	488 $[M+H]^+$	27.9	28
313	CN1CCC(CC(=O)NS(=O)(=O)C=2C=CC(=CC2)N3C=C(N=N3)C=4C=C(Cl)N=C(C4)C(F)(F)F)C1=O	[N-]=[N+]=NC=1C=CC(=CC1)S(N)(=O)=O	C#CC=1C=C(Cl)N=C(C1)C(F)(F)F	CN1CCC(CC(=O)O)C1=O	**7**{*3,6,24*}	543/545 as 3/1 $[M+H]^+$	26.1	26
314	O=C(NS(=O)(=O)C=1C=CC=C(C1)N2C=C(N=N2)C=3C=C(Br)C=C(Cl)C3)C4CC54CC5	[N-]=[N+]=NC=1C=CC=C(C1)S(N)(=O)=O	C#CC=1C=C(Cl)C=C(Br)C1	O=C(O)C1CC21CC2	**7**{*2,4,21*}	509/511/513 as 3/4/1 $[M+H]^+$	25.2	25
315	CC1=COC(=C1)C(=O)NS(=O)(=O)C=2C=CC=C(C2)N3C=C(N=N3)C=4C=CN=CC4Br	[N-]=[N+]=NC=1C=CC=C(C1)S(N)(=O)=O	C#CC=1C=CN=CC1Br	CC1=COC(=C1)C(=O)O	**7**{*2,23,63*}	488/490 as 1/1 $[M+H]^+$	25.2	25
316	CC12CC(C1)(C2)C(=O)NS(=O)(=O)C=3C=CC(=CC3)N4C=C(N=N4)C=5C=CN=CC5Br	[N-]=[N+]=NC=1C=CC(=CC1)S(N)(=O)=O	C#CC=1C=CN=CC1Br	CC12CC(C1)(C2)C(=O)O	**7**{*3,23,64*}	488/490 as 1/1 $[M+H]^+$	24.3	24
317	CN1N=CC=C1C(=O)NS(=O)(=O)C=2C=CC=C(C2)N3C=C(N=N3)C=4C=CC=CC4C=5C=CC=CC5	[N-]=[N+]=NC=1C=CC=C(C1)S(N)(=O)=O	C#CC=1C=CC=CC1C=2C=CC=CC2	CN1N=CC=C1C(=O)O	**7**{*2,18,49*}	485 $[M+H]^+$	20.3	20
318	CC=1C=C(C=CN1)C(=O)NS(=O)(=O)C=2C=CC=C(C2)N3C=C(N=N3)C=4C=CC(=CC4)COC(C)(C)C	[N-]=[N+]=NC=1C=CC=C(C1)S(N)(=O)=O	C#CC=1C=CC(=CC1)COC(C)(C)C	CC=1C=C(C=CN1)C(=O)O	**7**{*2,8,28*}	506 $[M+H]^+$	17.1	17
319	O=C(NS(=O)(=O)C=1C=CC(=CC1)N2C=C(N=N2)C=3C=CC=C(C3)C=4C=CC(Cl)=CC4)C5=NC(Cl)=CS5	[N-]=[N+]=NC=1C=CC(=CC1)S(N)(=O)=O	C#CC1=CC=CC(=C1)C=2C=CC(Cl)=CC2	O=C(O)C1=NC(Cl)=CS1	**7**{*3,31,25*}	556/558/560 as 9/6/1 $[M+H]^+$	16.7	17
320	CC(C)[C@@H]1C[C@H]1C(=O)NS(=O)(=O)C=2C=CC(=CC2)N3C=C(N=N3)C=4C=NC=5C=CSC5C4 |&1:3,5,r|	[N-]=[N+]=NC=1C=CC(=CC1)S(N)(=O)=O	C#CC=1C=NC=2C=CSC2C1	CC(C)[C@@H]1C[C@H]1C(=O)O |o1:3,5,r|	**7**{*3,24,57*}	468 $[M+H]^+$	16.2	16
321	O=C(NS(=O)(=O)C=1C=CC=C(C1)N2C=C(N=N2)C=3C=CC(=CC3)OC4CCCC4)C5CC65CCC6	[N-]=[N+]=NC=1C=CC=C(C1)S(N)(=O)=O	C#CC=1C=CC(=CC1)OC2CCCC2	O=C(O)C1CC21CCC2	**7**{*2,9,29*}	493 $[M+H]^+$	13.5	14
322	CC=1C=CC(=CC1C2=CN(N=N2)C=3C=CC=C(C3)S(=O)(=O)NC(=O)CC(C)C)C(F)(F)F	[N-]=[N+]=NC=1C=CC=C(C1)S(N)(=O)=O	C#CC=1C=C(C=CC1C)C(F)(F)F	CC(C)CC(=O)O	**7**{*2,1,16*}	467 $[M+H]^+$	9	9
323	O=C(NS(=O)(=O)C=1C=CC(=CC1)CN2C=C(N=N2)C=3C=CC=C(Br)N3)C4=CC=CO4	[N-]=[N+]=NCC=1C=CC(=CC1)S(N)(=O)=O	C#CC1=CC=CC(Br)=N1	O=C(O)C1=CC=CO1	**7**{*1,33,6*}	488/490 as 1/1 $[M+H]^+$	6.3	6
324	O=C(NS(=O)(=O)C=1C=CC=C(C1)N2C=C(N=N2)C3=CC(Cl)=CC=C3Br)C4=CC=NS4	[N-]=[N+]=NC=1C=CC=C(C1)S(N)(=O)=O	C#CC=1C=C(Cl)C=CC1Br	O=C(O)C1=CC=NS1	**7**{*2,5,22*}	N/A	0	0
325	CCOC(=O)COC1(COC1)C2=CN(N=N2)C=3C=CC(=CC3)S(=O)(=O)NC(=O)C4=COC(C)=C4	[N-]=[N+]=NC=1C=CC(=CC1)S(N)(=O)=O	C#CC1(COC1)OCC(=O)OCC	CC1=CC(=CO1)C(=O)O	**7**{*3,25,66*}	N/A	0	0
326	O=C(NS(=O)(=O)C=1C=CC(=CC1)CN2C=C(CCN3CCN(CC3)C=4C=CC=CC4)N=N2)C=5C=CC=C(F)C5	[N-]=[N+]=NCC=1C=CC(=CC1)S(N)(=O)=O	Cl.Cl.C#CCCN1CCN(CC1)C=2C=CC=CC2	O=C(O)C=1C=CC=C(F)C1	**7**{*1,51,41*}	N/A	0	0
327	O=C(NS(=O)(=O)C=1C=CC(=CC1)N2C=C(N=N2)C3=CC(Cl)=NC(=C3)C(F)(F)F)C4=NC(Cl)=CS4	[N-]=[N+]=NC=1C=CC(=CC1)S(N)(=O)=O	C#CC=1C=C(Cl)N=C(C1)C(F)(F)F	O=C(O)C1=NC(Cl)=CS1	**7**{*3,6,25*}	N/A	0	0
328	O=C(NS(=O)(=O)C=1C=CC(=CC1)N2C=C(N=N2)C=3C=CC(Br)=CN3)C4=CC(Cl)=NS4	[N-]=[N+]=NC=1C=CC(=CC1)S(N)(=O)=O	C#CC=1C=CC(Br)=CN1	O=C(O)C1=CC(Cl)=NS1	**7**{*3,11,39*}	N/A	0	0
329	CC1CC(C1)C(=O)NS(=O)(=O)C=2C=CC=C(C2)N3C=C(N=N3)C4=CC(Cl)=CC(=C4)CO	[N-]=[N+]=NC=1C=CC=C(C1)S(N)(=O)=O	C#CC1=CC(Cl)=CC(=C1)CO	CC1CC(C1)C(=O)O	**7**{*2,28,31*}	N/A	0	0
330	CC1CC(C1)C(=O)NS(=O)(=O)C=2C=CC=C(C2)N3C=C(CCC=4C=C(F)C=C(F)C4)N=N3	[N-]=[N+]=NC=1C=CC=C(C1)S(N)(=O)=O	C#CCCC=1C=C(F)C=C(F)C1	CC1CC(C1)C(=O)O	**7**{*2,41,31*}	N/A	0	0
331	CC1(F)CC(C1)C(=O)NS(=O)(=O)C=2C=CC(=CC2)N3C=C(N=N3)C=4C=CC=5SC=NC5C4	[N-]=[N+]=NC=1C=CC(=CC1)S(N)(=O)=O	C#CC=1C=CC=2SC=NC2C1	CC1(F)CC(C1)C(=O)O	**7**{*3,15,46*}	N/A	0	0
332	O=C(NS(=O)(=O)C=1C=CC(=CC1)CN2C=C(N=N2)C=3C=CC=C(C3)C=4C=CC=CC4)C5=NC=CS5	[N-]=[N+]=NCC=1C=CC(=CC1)S(N)(=O)=O	C#CC=1C=CC=C(C1)C=2C=CC=CC2	O=C(O)C1=NC=CS1	**7**{*1,16,15*}	N/A	0	0
333	CC=1C=CN=CC1C(=O)NS(=O)(=O)C=2C=CC(=CC2)CN3C=C(N=N3)C=4C=CC=C5C=CC=NC45	[N-]=[N+]=NCC=1C=CC(=CC1)S(N)(=O)=O	C#CC=1C=CC=C2C=CC=NC12	CC=1C=CN=CC1C(=O)O	**7**{*1,17,54*}	N/A	0	0
334	O=C(NS(=O)(=O)C=1C=CC(=CC1)N2C=C(N=N2)C=3C=CC=4SC=NC4C3)C=5SC=NC5Cl	[N-]=[N+]=NC=1C=CC(=CC1)S(N)(=O)=O	C#CC=1C=CC=2SC=NC2C1	O=C(O)C=1SC=NC1Cl	**7**{*3,15,45*}	N/A	0	0
335	CC1=CON=C1C(=O)NS(=O)(=O)C=2C=CC(=CC2)N3C=C(N=N3)C=4C=CN=CC4Br	[N-]=[N+]=NC=1C=CC(=CC1)S(N)(=O)=O	C#CC=1C=CN=CC1Br	CC1=CON=C1C(=O)O	**7**{*3,23,65*}	N/A	0	0
336	O=C(NS(=O)(=O)C=1C=CC(=CC1)CN2C=C(N=N2)C=3C=CC=C4C=CC=NC34)C=5C=NC=NC5	[N-]=[N+]=NCC=1C=CC(=CC1)S(N)(=O)=O	C#CC=1C=CC=C2C=CC=NC12	O=C(O)C=1C=NC=NC1	**7**{*1,17,56*}	N/A	0	0

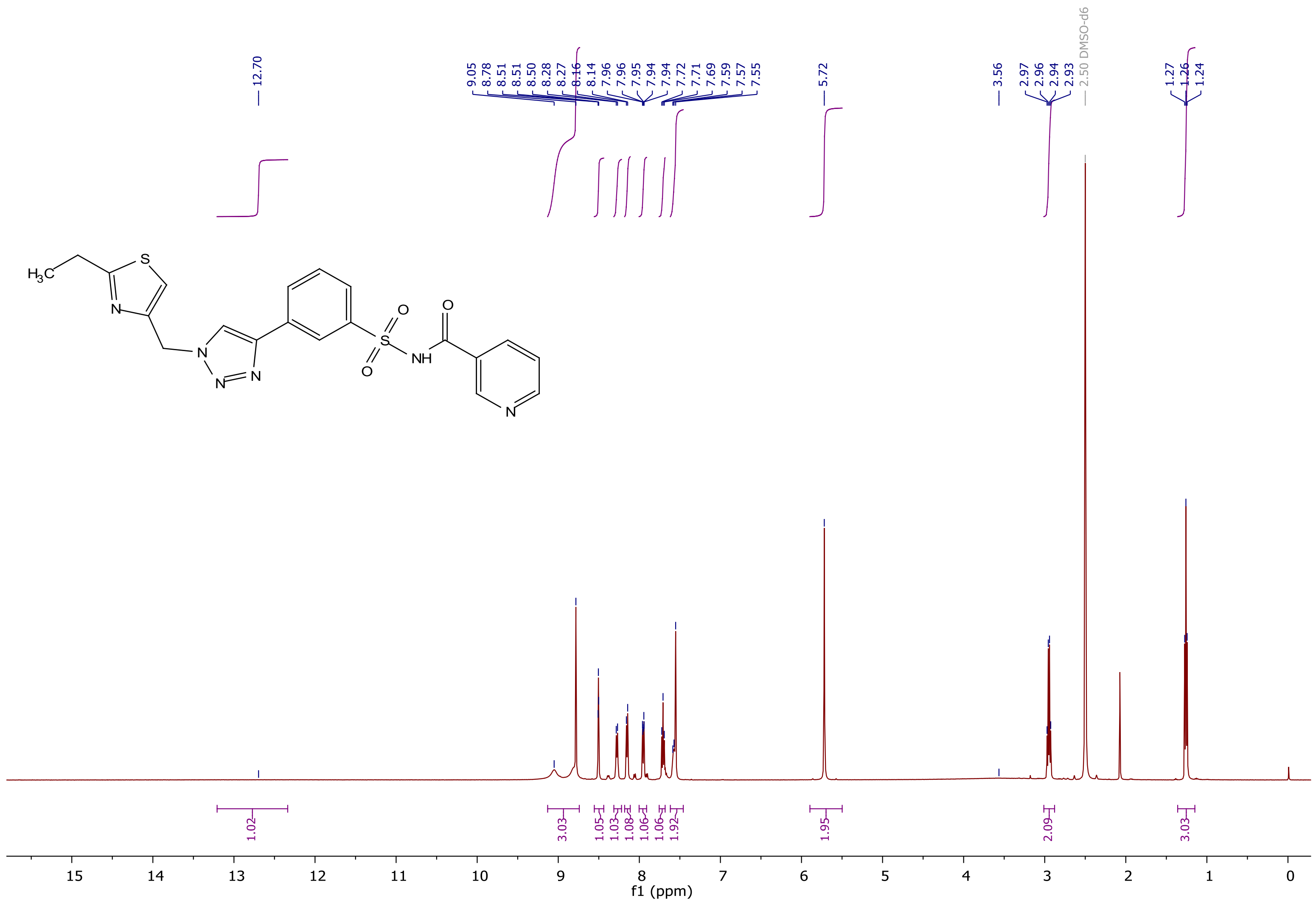

***N*-((3-(1-((2-Ethylthiazol-4-yl)methyl)-1*H*-1,2,3-triazol-4-yl)phenyl)sulfonyl)nicotinamide (4{32,6,17}) ^{1}H NMR**

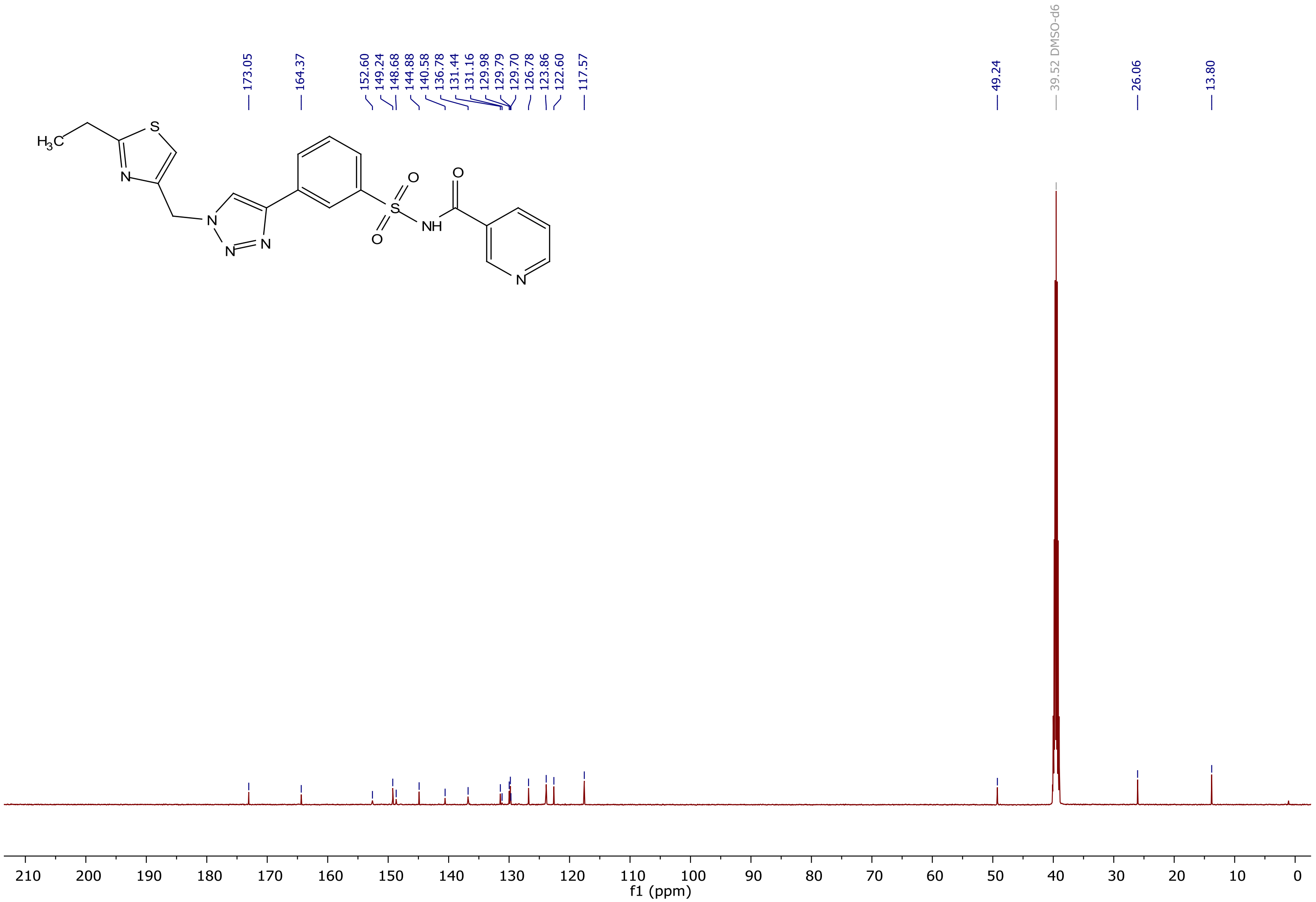

***N*-((3-(1-((2-Ethylthiazol-4-yl)methyl)-1*H*-1,2,3-triazol-4-yl)phenyl)sulfonyl)nicotinamide (4{32,6,17}) ^{13}C NMR**

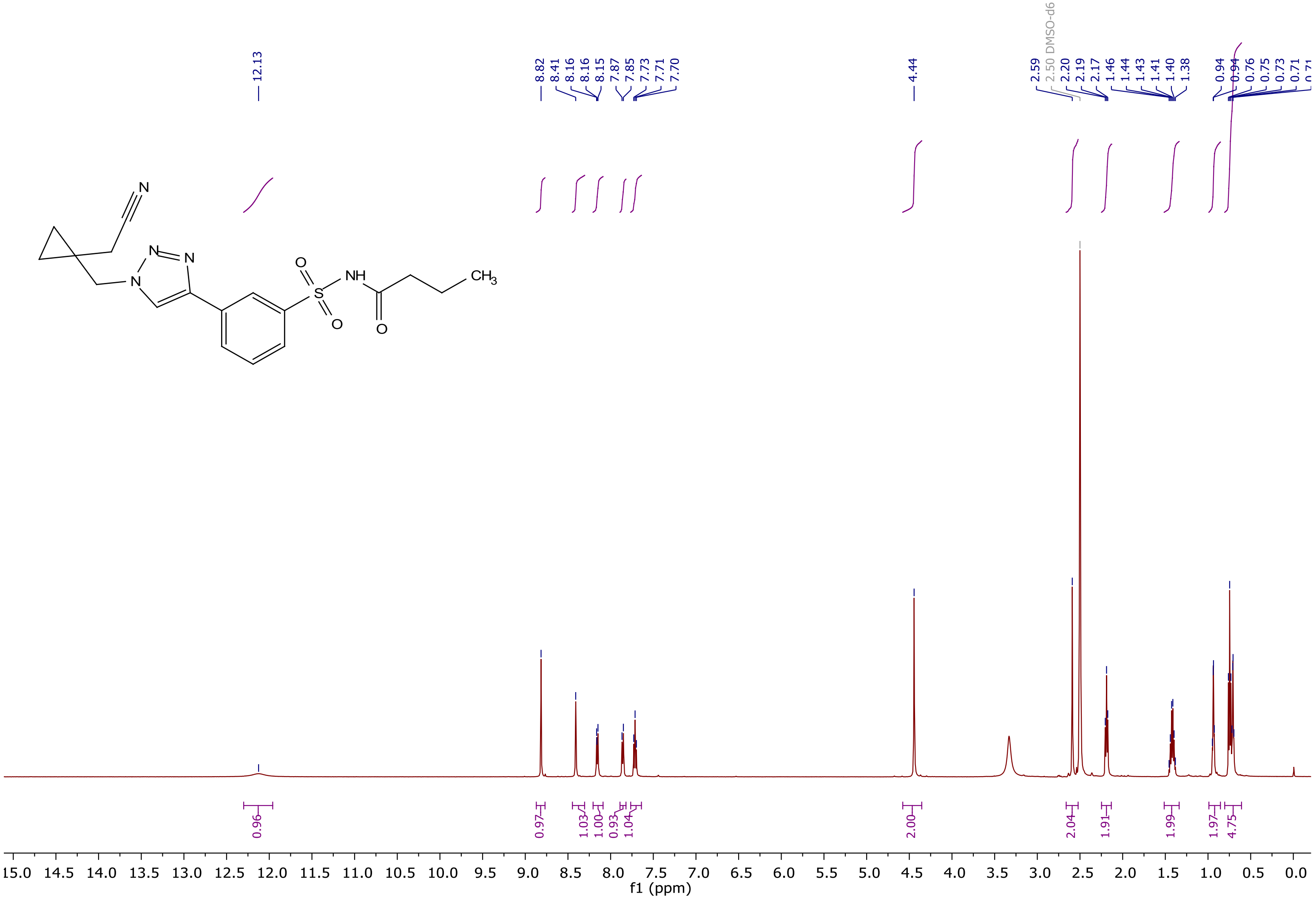

***N*-((3-(1-((1-(Cyanomethyl)cyclopropyl)methyl)-1*H*-1,2,3-triazol-4-yl)phenyl)sulfonyl)butyramide (4{33,6,4}) ^{1}H NMR**

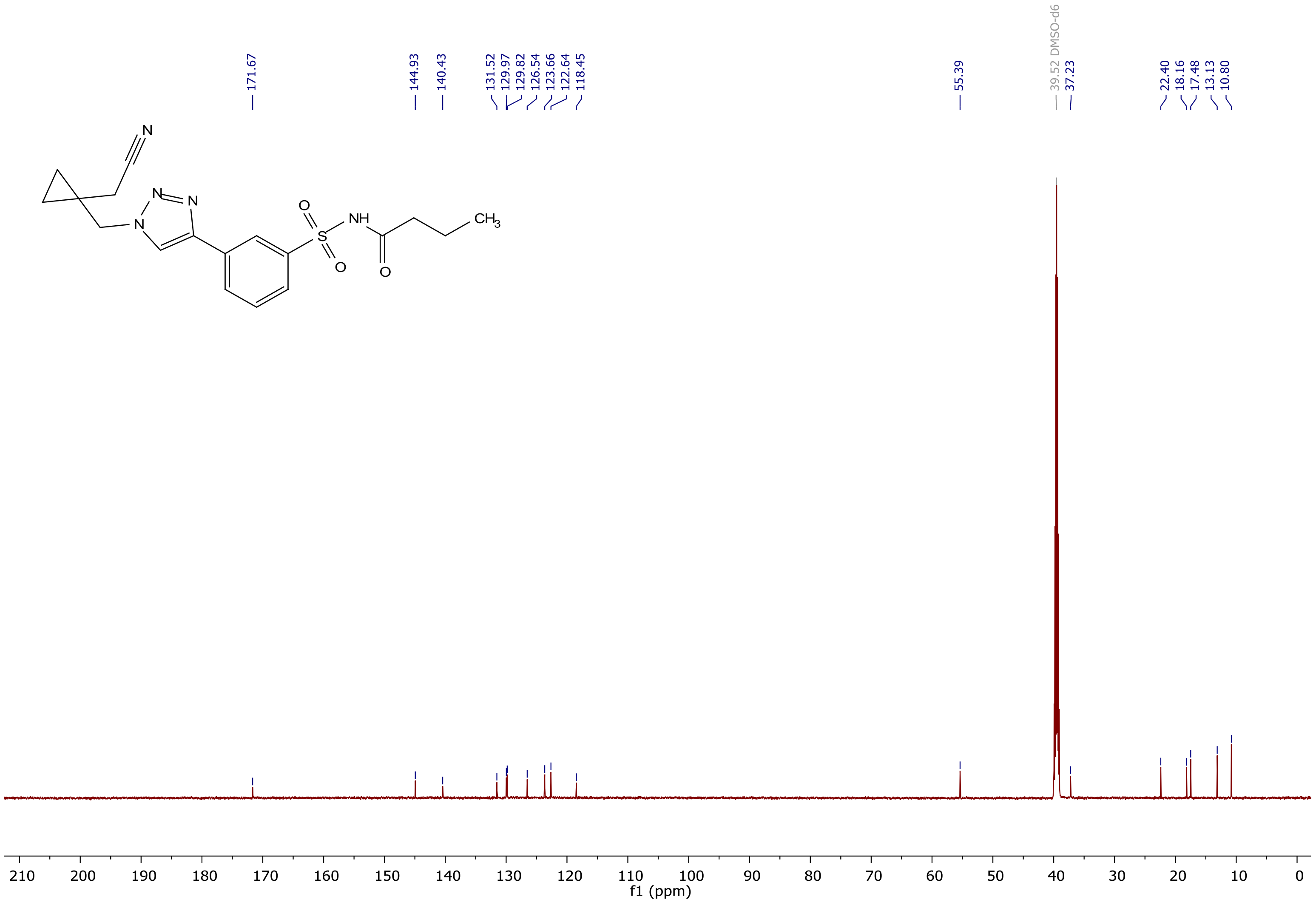

***N*-((3-(1-((1-(Cyanomethyl)cyclopropyl)methyl)-1*H*-1,2,3-triazol-4-yl)phenyl)sulfonyl)butyramide (4{33,6,4}) ^{13}C NMR**

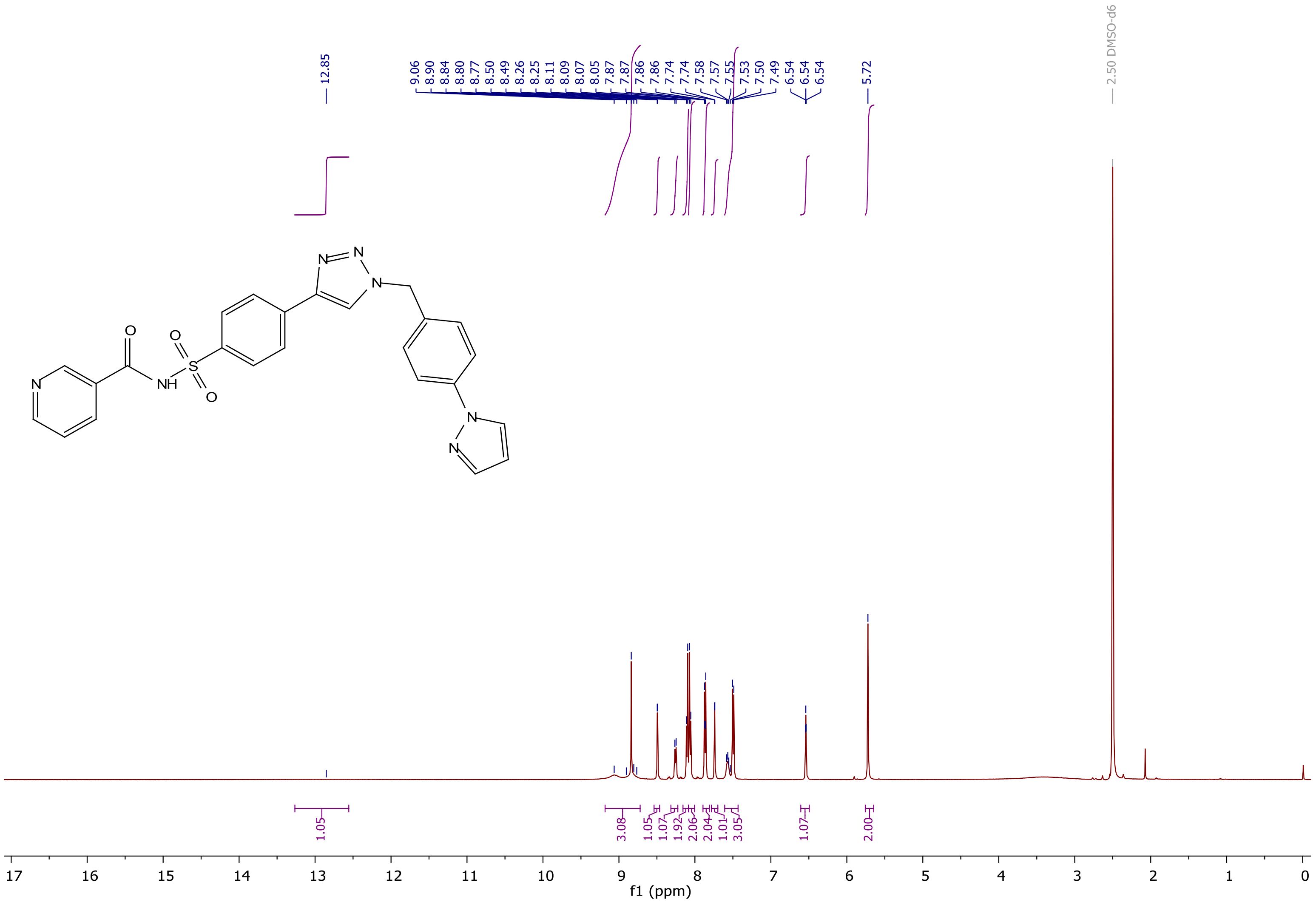

***N*-((4-(1-(4-(1*H*-Pyrazol-1-yl)benzyl)-1*H*-1,2,3-triazol-4-yl)phenyl)sulfonyl)nicotinamide (4{18,7,17}) ^{1}H NMR**

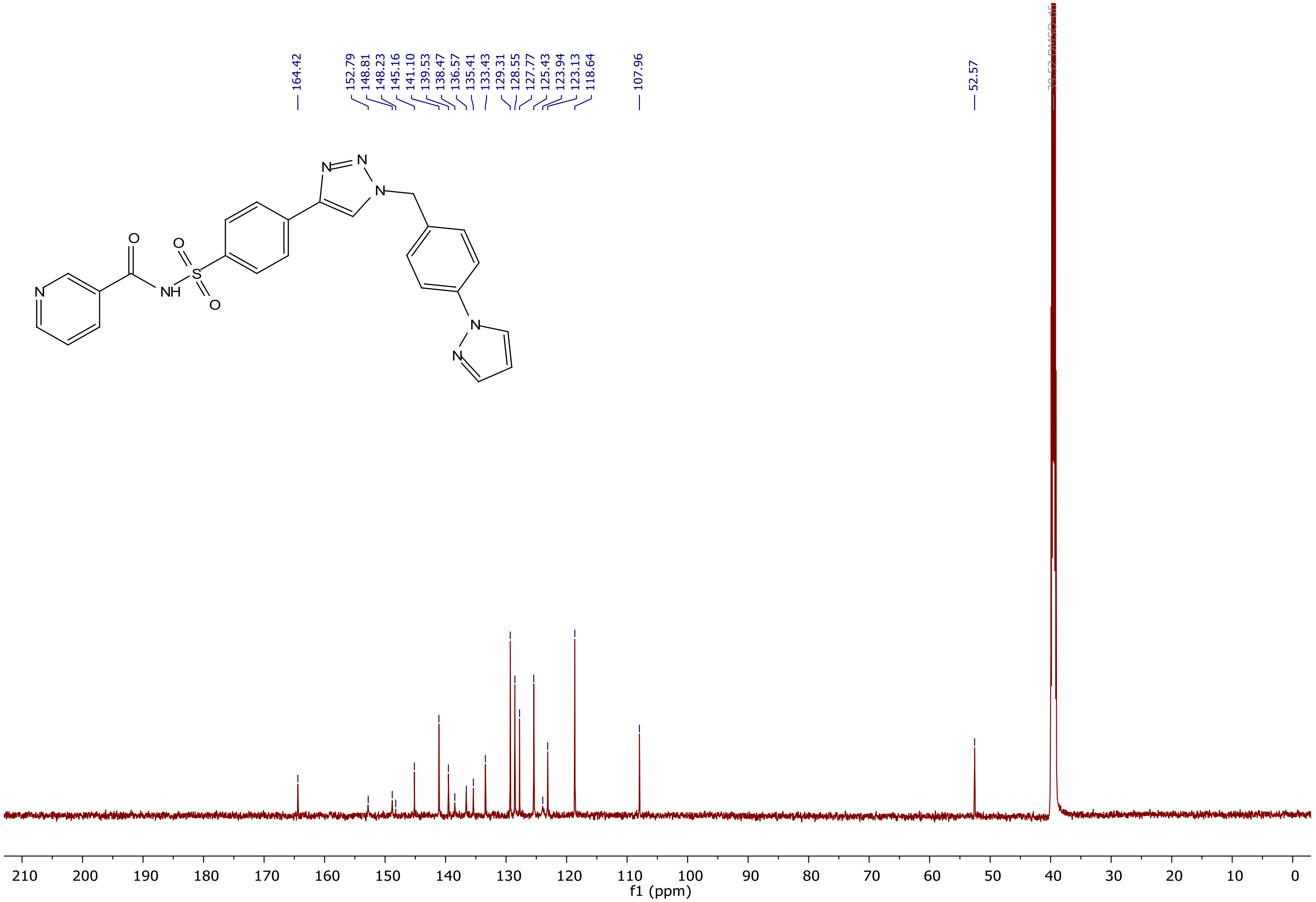

***N*-((4-(1-(4-(1*H*-Pyrazol-1-yl)benzyl)-1*H*-1,2,3-triazol-4-yl)phenyl)sulfonyl)nicotinamide (4{18,7,17}) ^{13}C NMR**

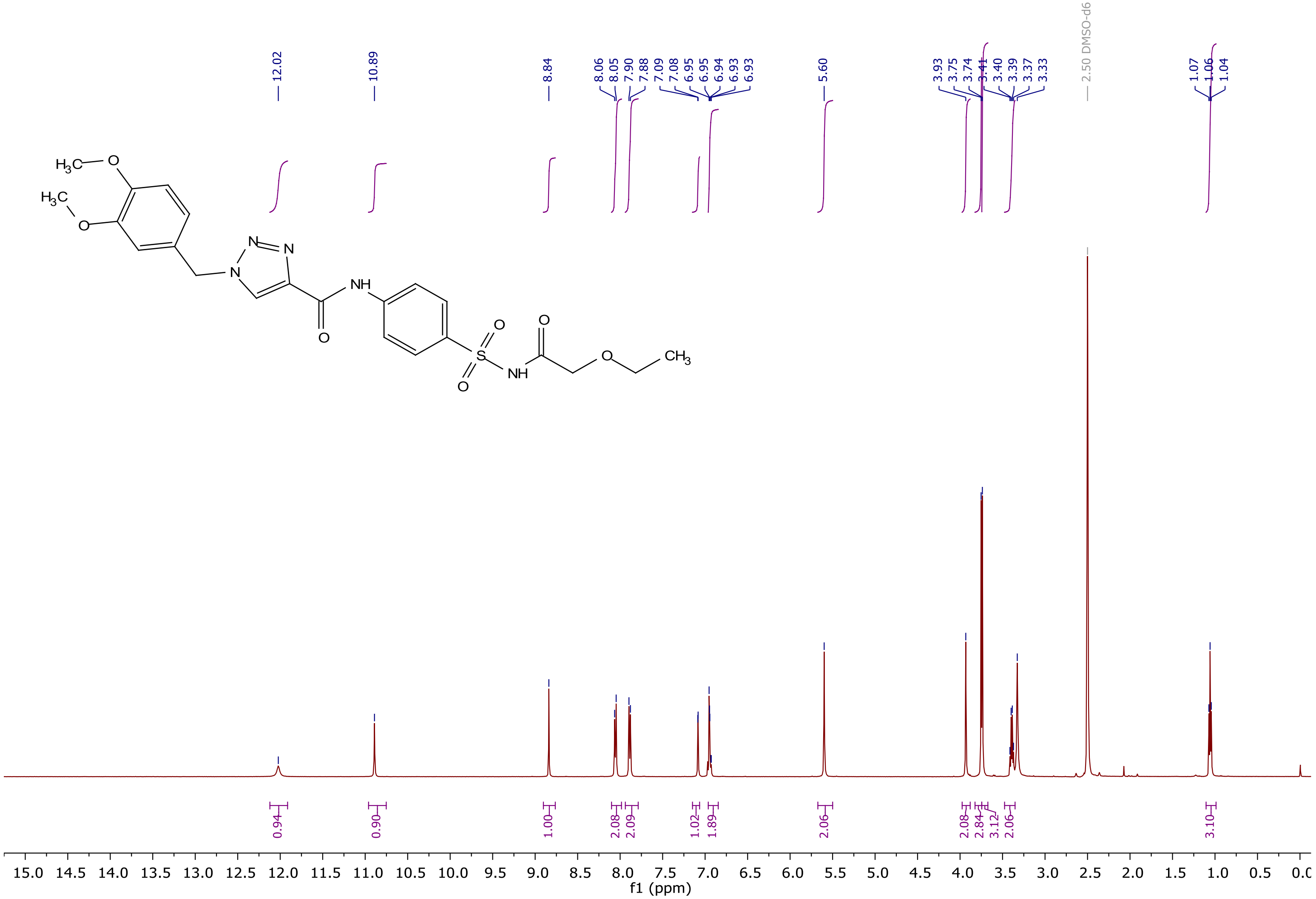

1-(3,4-Dimethoxybenzyl)-*N*-(4-(*N*-(2-ethoxyacetyl)sulfamoyl)phenyl)-1*H*-1,2,3-triazole-4-carboxamide (4{1,8,1}) ^{1}H NMR

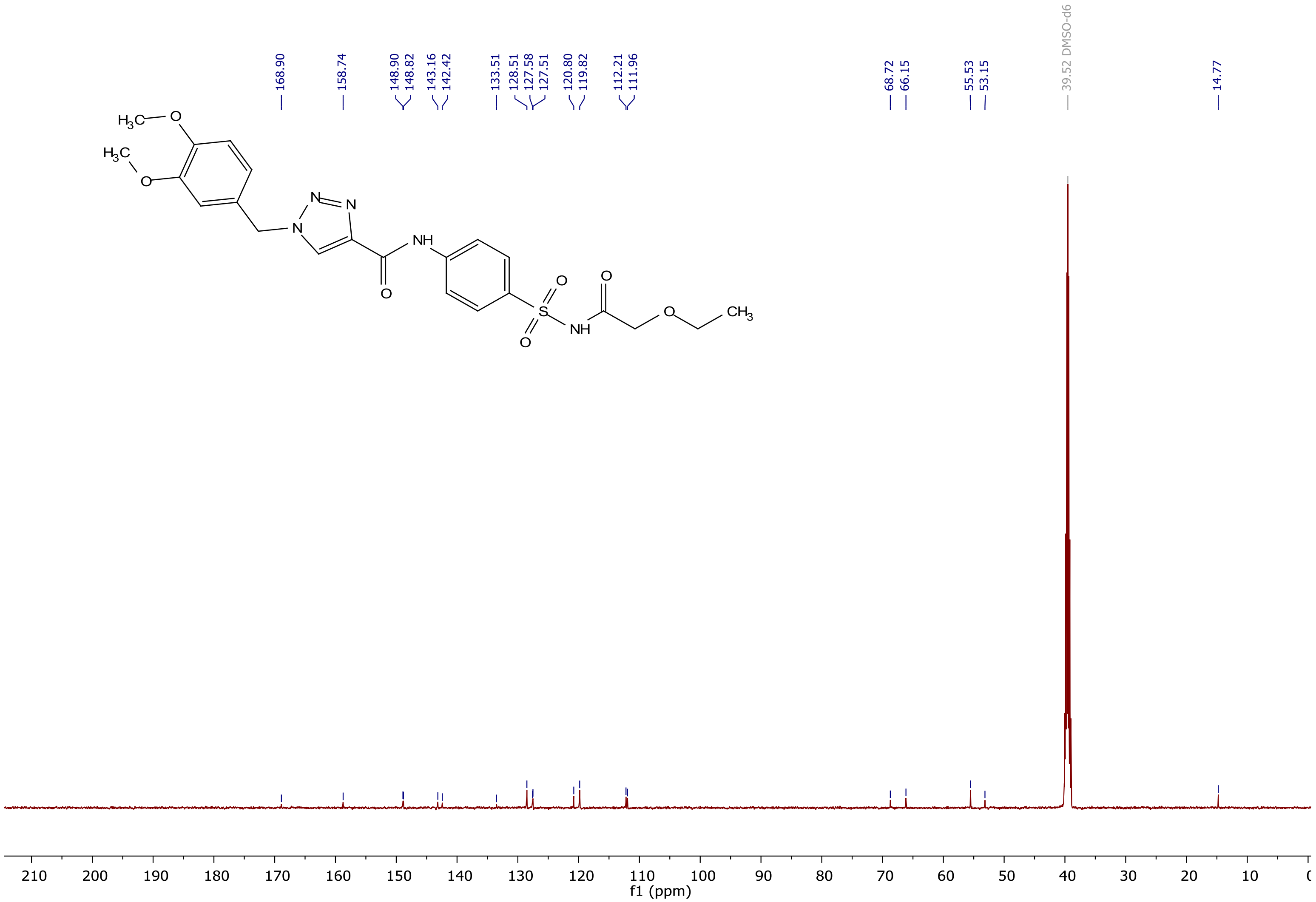

1-(3,4-Dimethoxybenzyl)-*N*-(4-(*N*-(2-ethoxyacetyl)sulfamoyl)phenyl)-1*H*-1,2,3-triazole-4-carboxamide (4{1,8,1}) ^{13}C NMR

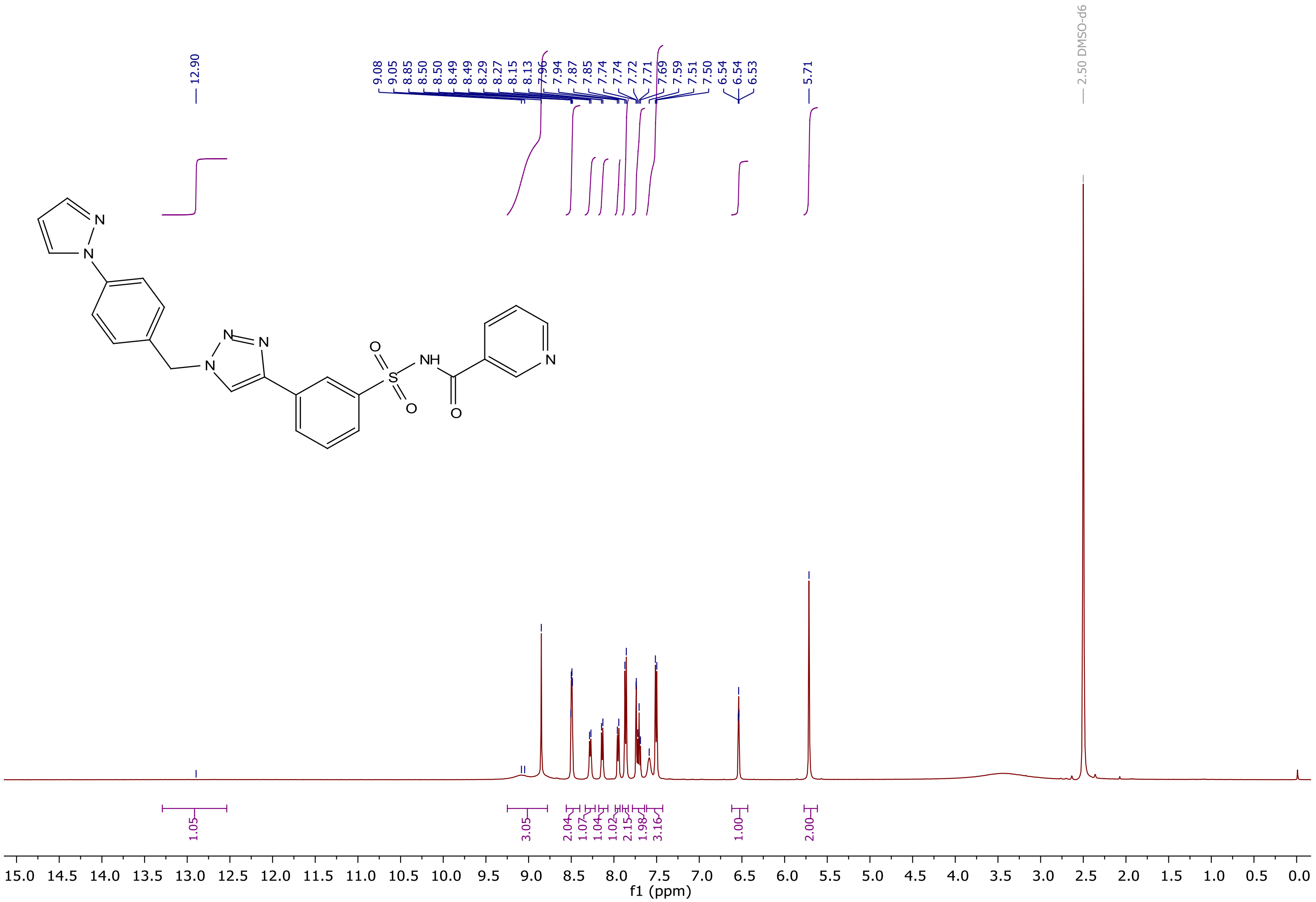

***N*-((3-(1-(4-(1*H*-Pyrazol-1-yl)benzyl)-1*H*-1,2,3-triazol-4-yl)phenyl)sulfonyl)nicotinamide (4{18,6,17}) ^{1}H NMR**

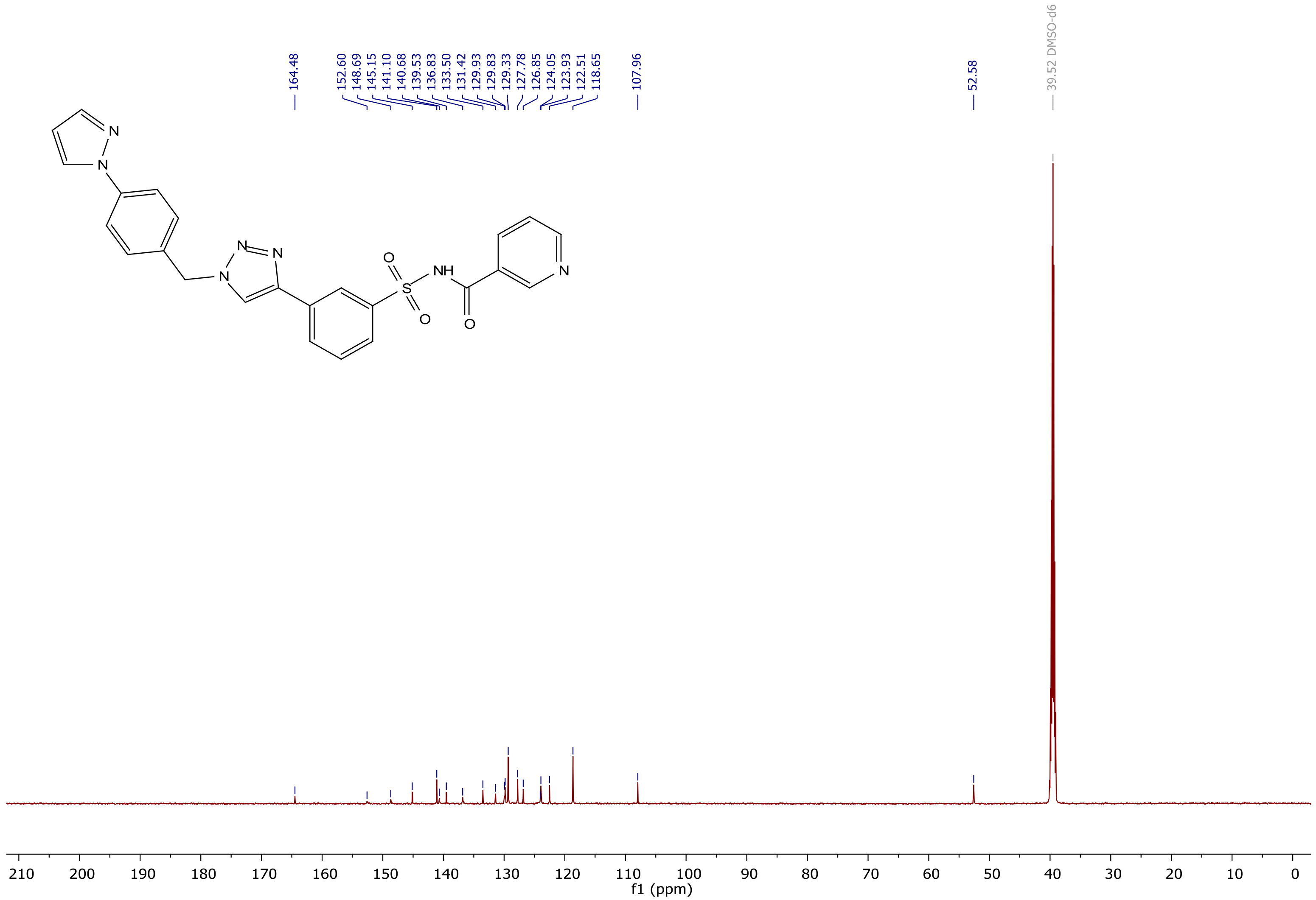

***N*-((3-(1-(4-(1*H*-Pyrazol-1-yl)benzyl)-1*H*-1,2,3-triazol-4-yl)phenyl)sulfonyl)nicotinamide (4{18,6,17}) ^{13}C NMR**

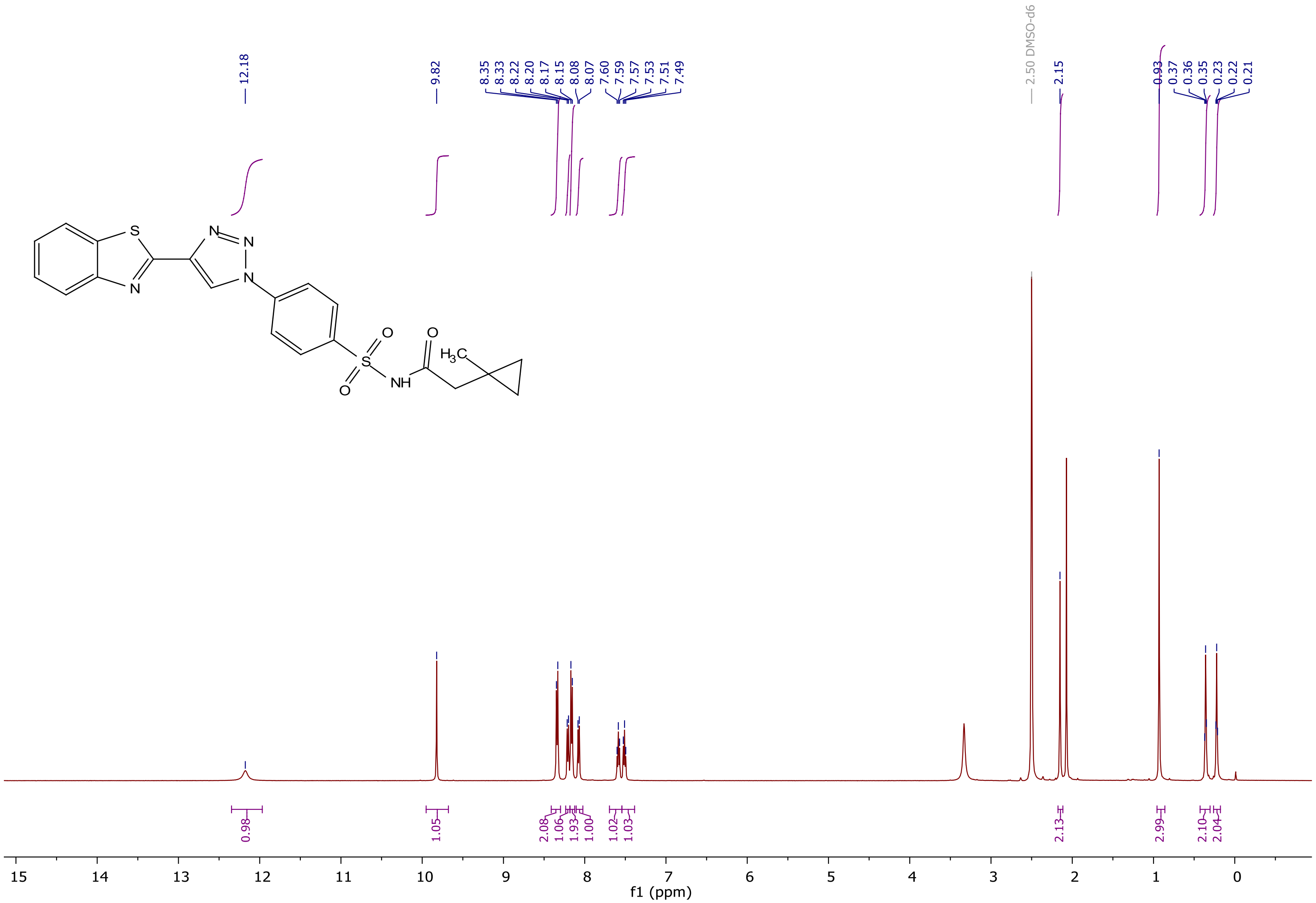

***N*-((4-(4-(Benzo[*d*]thiazol-2-yl)-1*H*-1,2,3-triazol-1-yl)phenyl)sulfonyl)-2-(1-methylcyclopropyl)acetamide (7{3,37,71}) ^{1}H NMR**

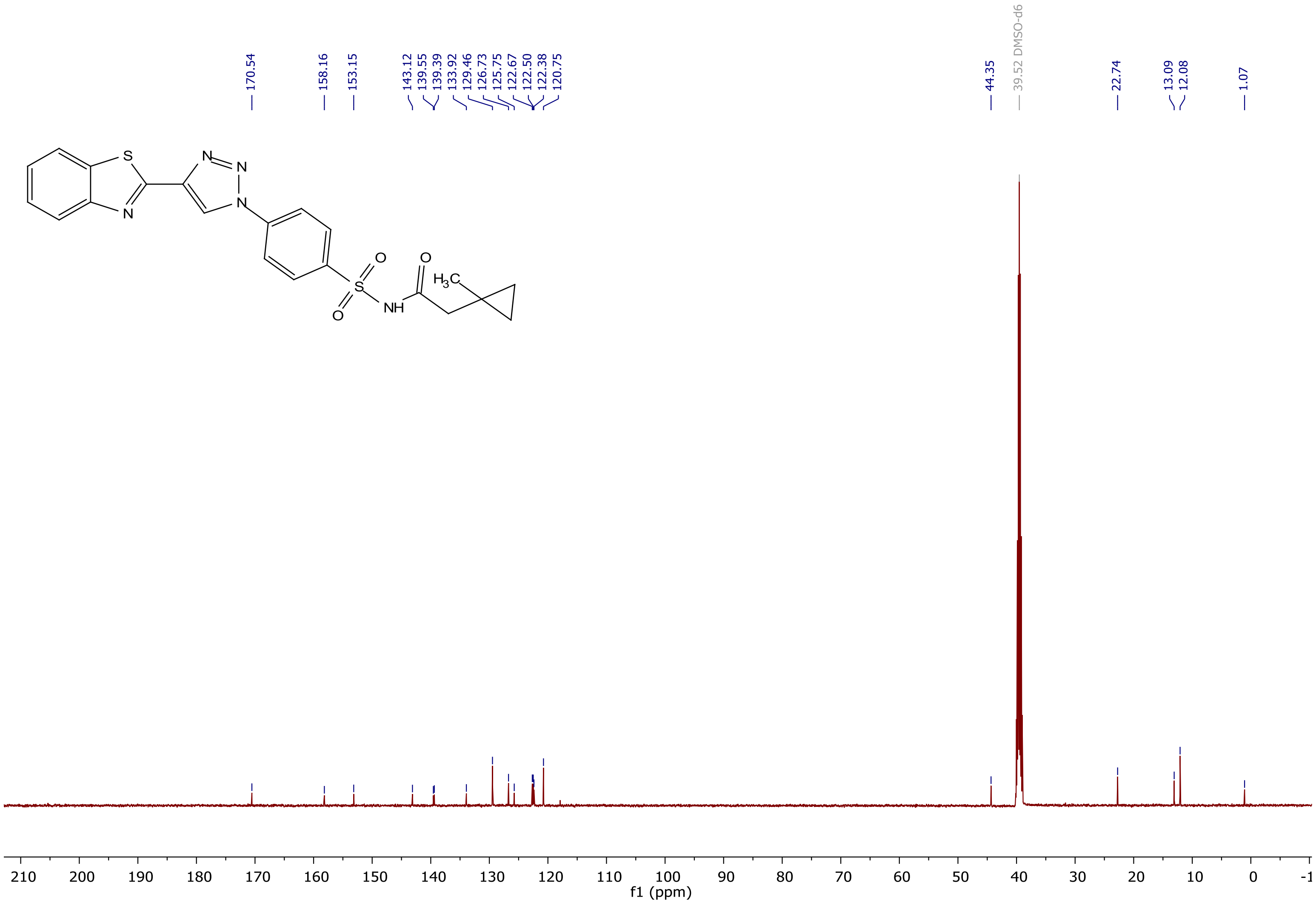

***N*-((4-(4-(Benzo[*d*]thiazol-2-yl)-1*H*-1,2,3-triazol-1-yl)phenyl)sulfonyl)-2-(1-methylcyclopropyl)acetamide (7{3,37,71}) ^{13}C NMR**

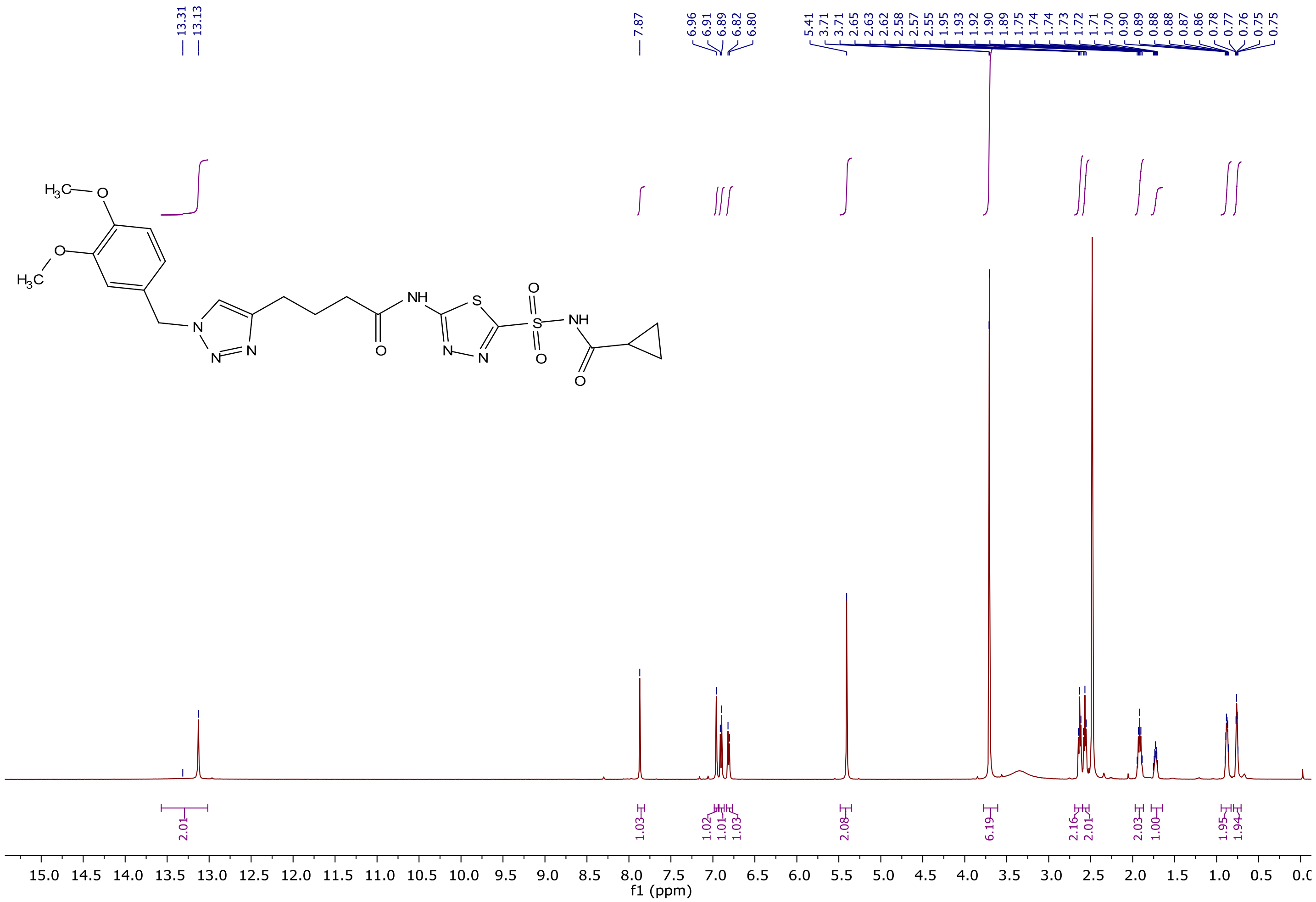

N-((5-(4-(1-(3,4-Dimethoxybenzyl)-1*H*-1,2,3-triazol-4-yl)butanamido)-1,3,4-thiadiazol-2-yl)sulfonyl)cyclopropanecarboxamide (4{1,5,2}) ^{1}H NMR

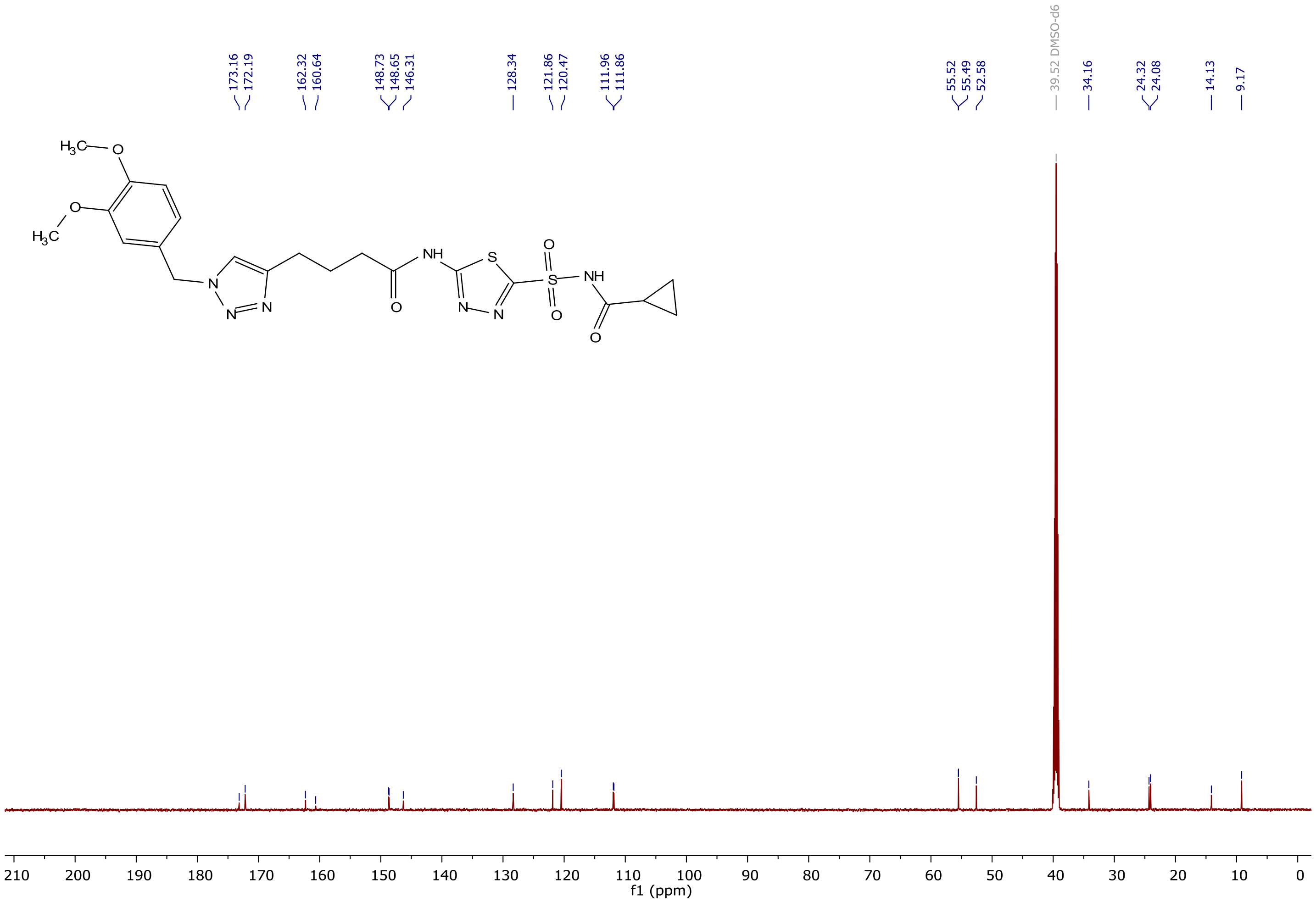

***N*-((5-(4-(1-(3,4-Dimethoxybenzyl)-1*H*-1,2,3-triazol-4-yl)butanamido)-1,3,4-thiadiazol-2-yl)sulfonyl)cyclopropanecarboxamide (4{1,5,2}) ^{13}C NMR**

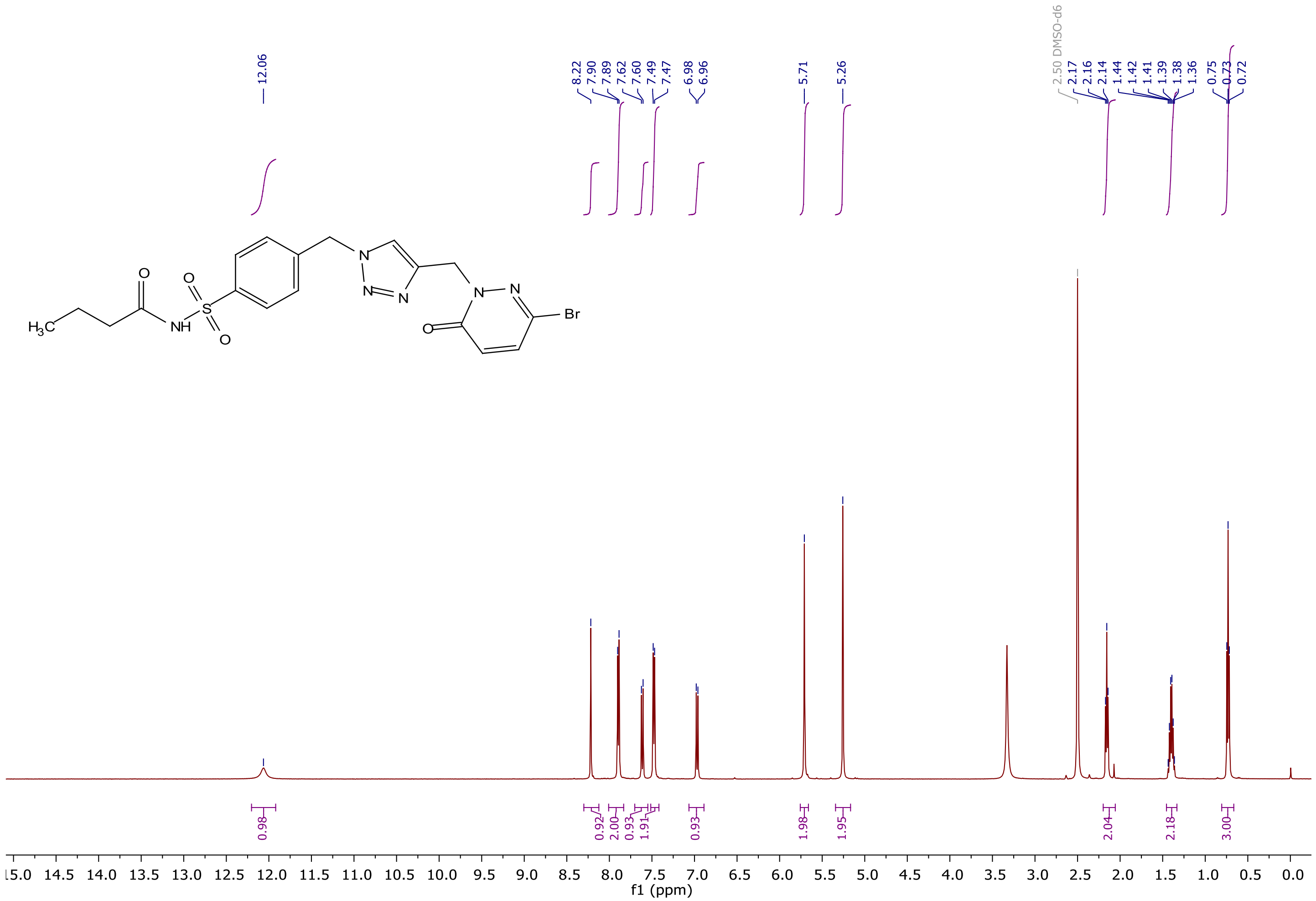

***N*-((4-((4-((3-Bromo-6-oxopyridazin-1(6*H*)-yl)methyl)-1*H*-1,2,3-triazol-1-yl)methyl)phenyl)sulfonyl)butyramide (7{1,46,4})**

^{1}H NMR

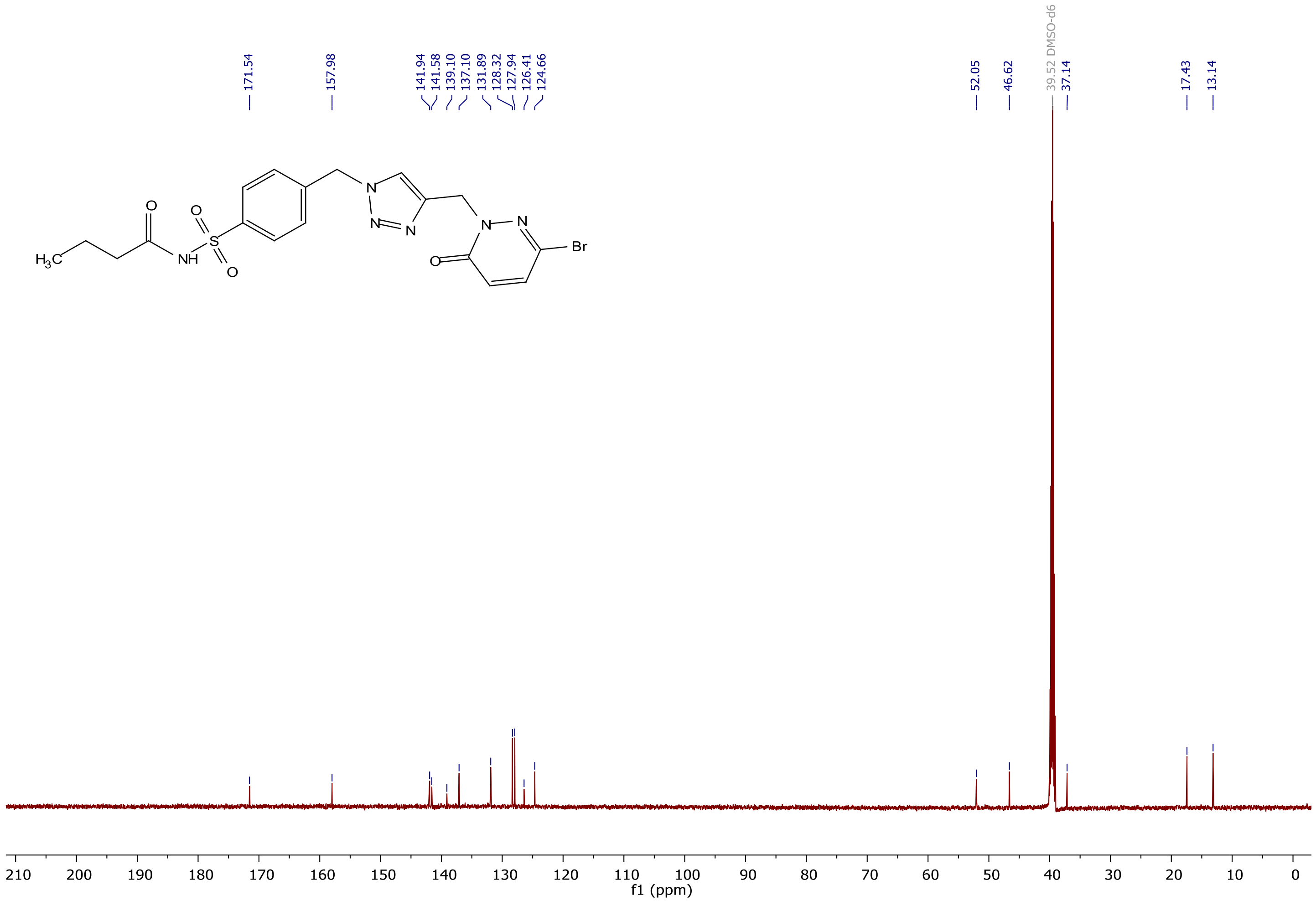

N-((4-((4-((3-Bromo-6-oxopyridazin-1(6H)-yl)methyl)-1H-1,2,3-triazol-1-yl)methyl)phenyl)sulfonyl)butyramide (7{1,46,4})

^{13}C NMR

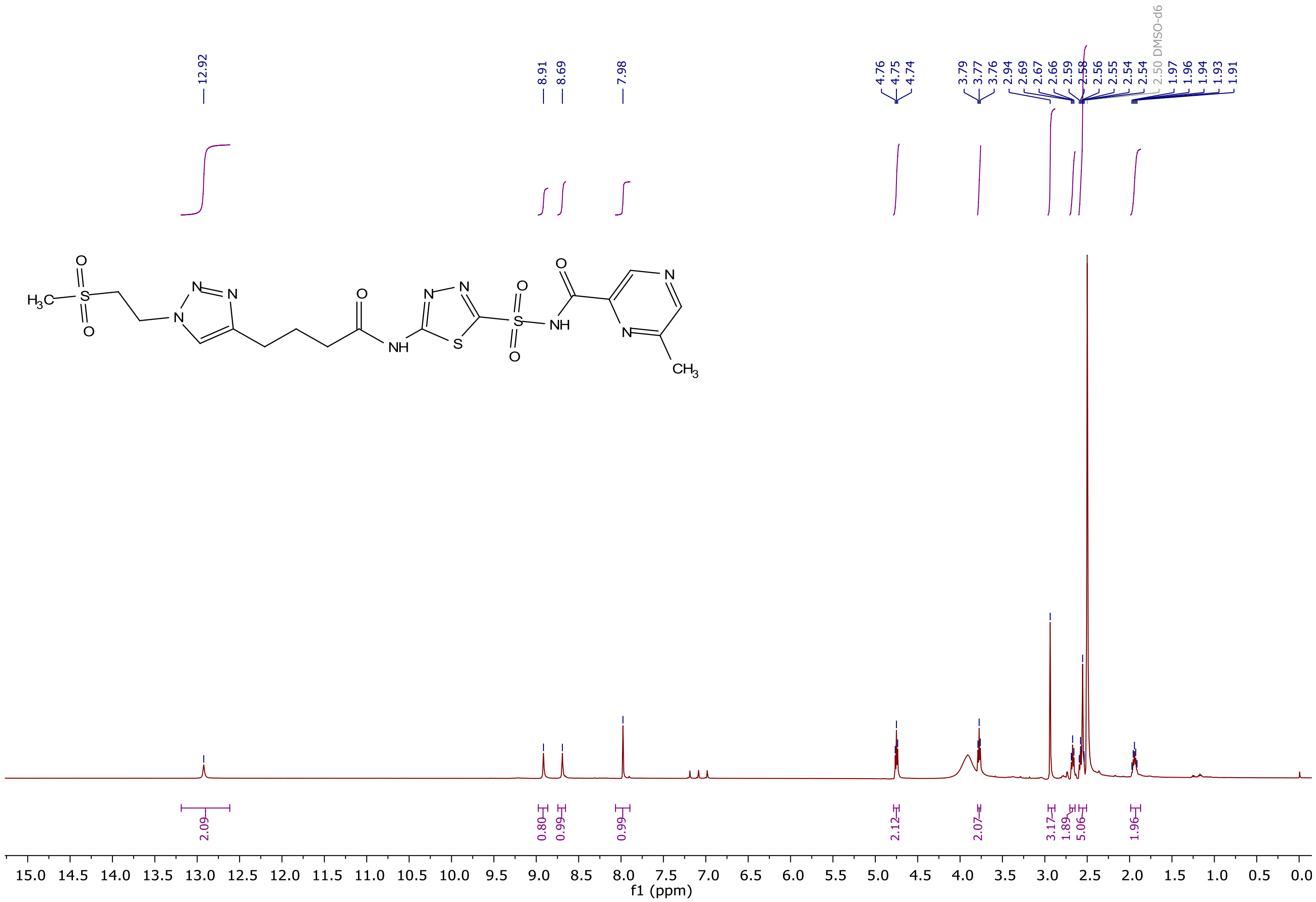

6-Methyl-*N*-((5-(4-(1-(2-(methylsulfonyl)ethyl)-1*H*-1,2,3-triazol-4-yl)butanamido)-1,3,4-thiadiazol-2-yl)sulfonyl)pyrazine-2-carboxamide (4{45,5,30}) ^{1}H NMR

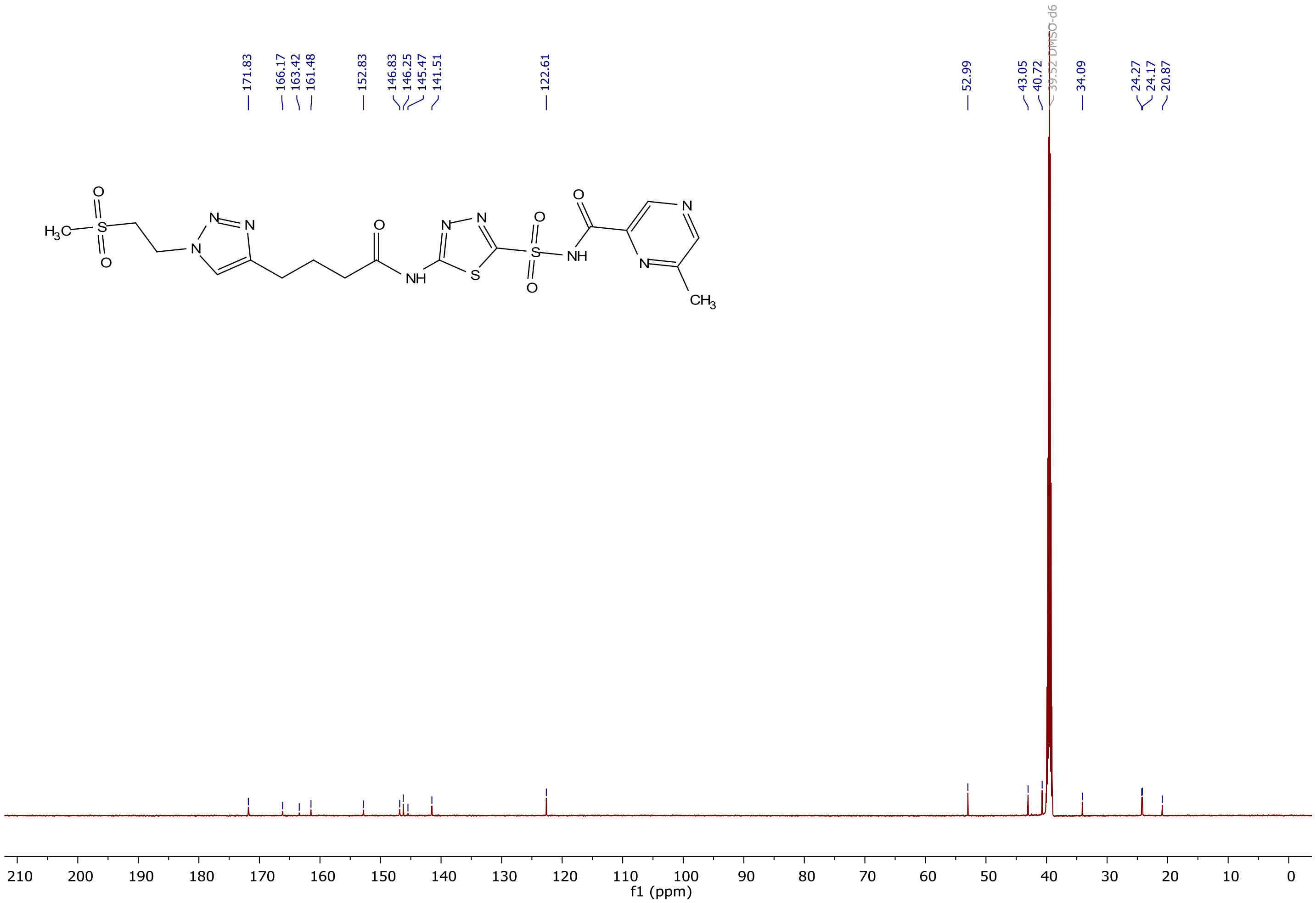

6-Methyl-*N*-((5-(4-(1-(2-(methylsulfonyl)ethyl)-1*H*-1,2,3-triazol-4-yl)butanamido)-1,3,4-thiadiazol-2-yl)sulfonyl)pyrazine-2-carboxamide (4{45,5,30}) ^{13}C NMR

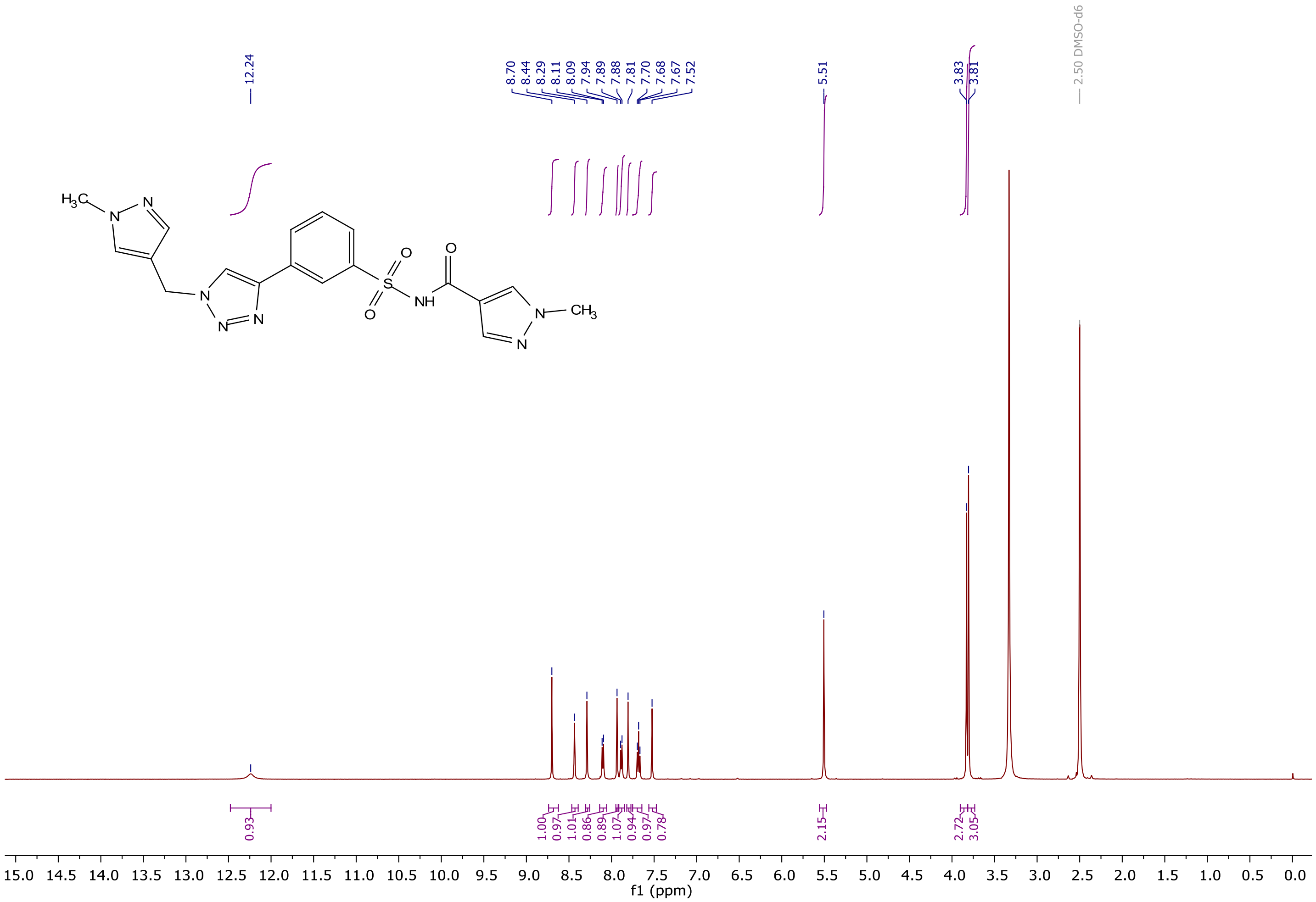

1-Methyl-*N*-((3-(1-((1-methyl-1*H*-pyrazol-4-yl)methyl)-1*H*-1,2,3-triazol-4-yl)phenyl)sulfonyl)-1*H*-pyrazole-4-carboxamide (4{34,6,47}) ^{1}H NMR

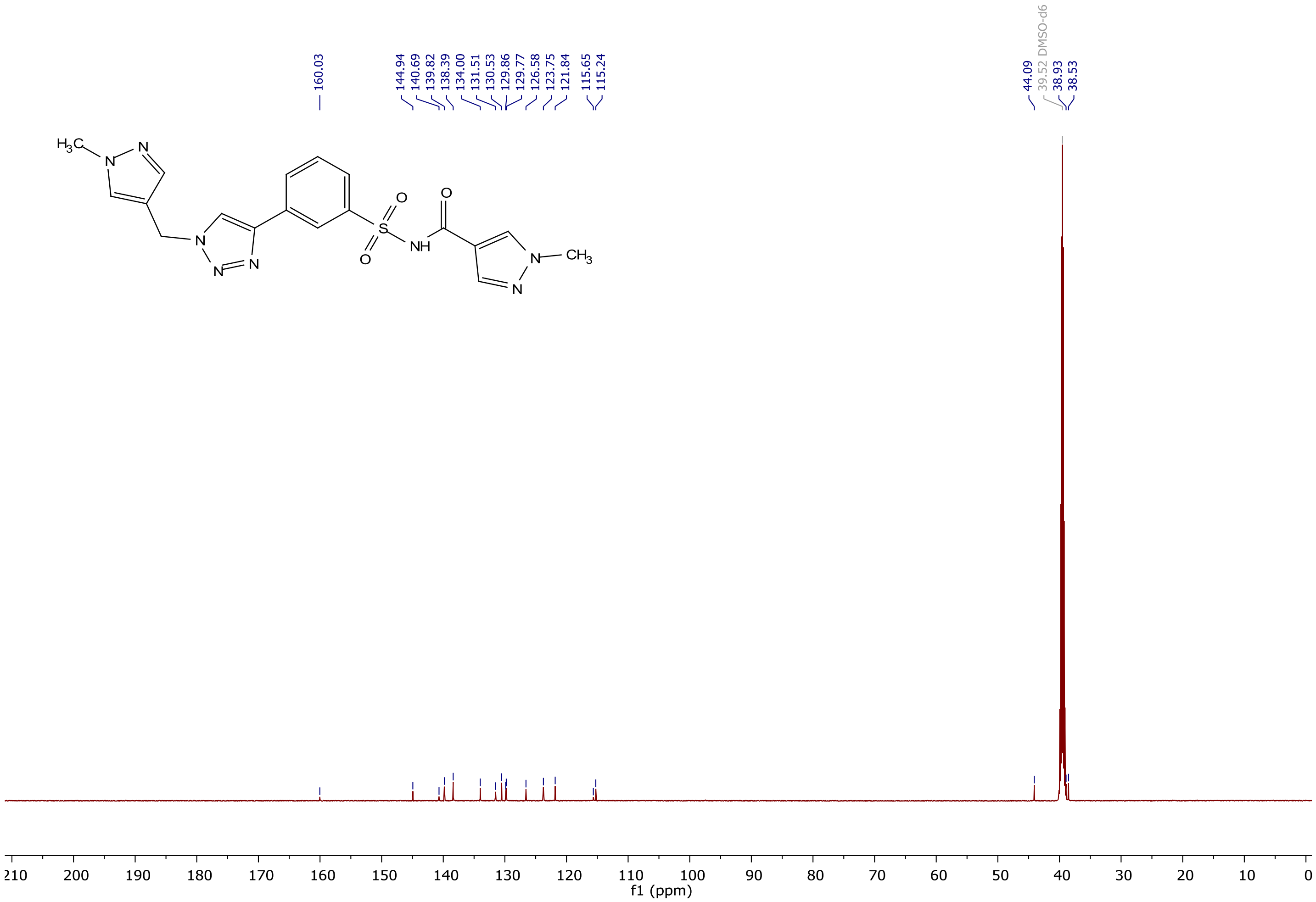

1-Methyl-*N*-((3-(1-((1-methyl-1*H*-pyrazol-4-yl)methyl)-1*H*-1,2,3-triazol-4-yl)phenyl)sulfonyl)-1*H*-pyrazole-4-carboxamide (4{34,6,47}) ^{13}C NMR

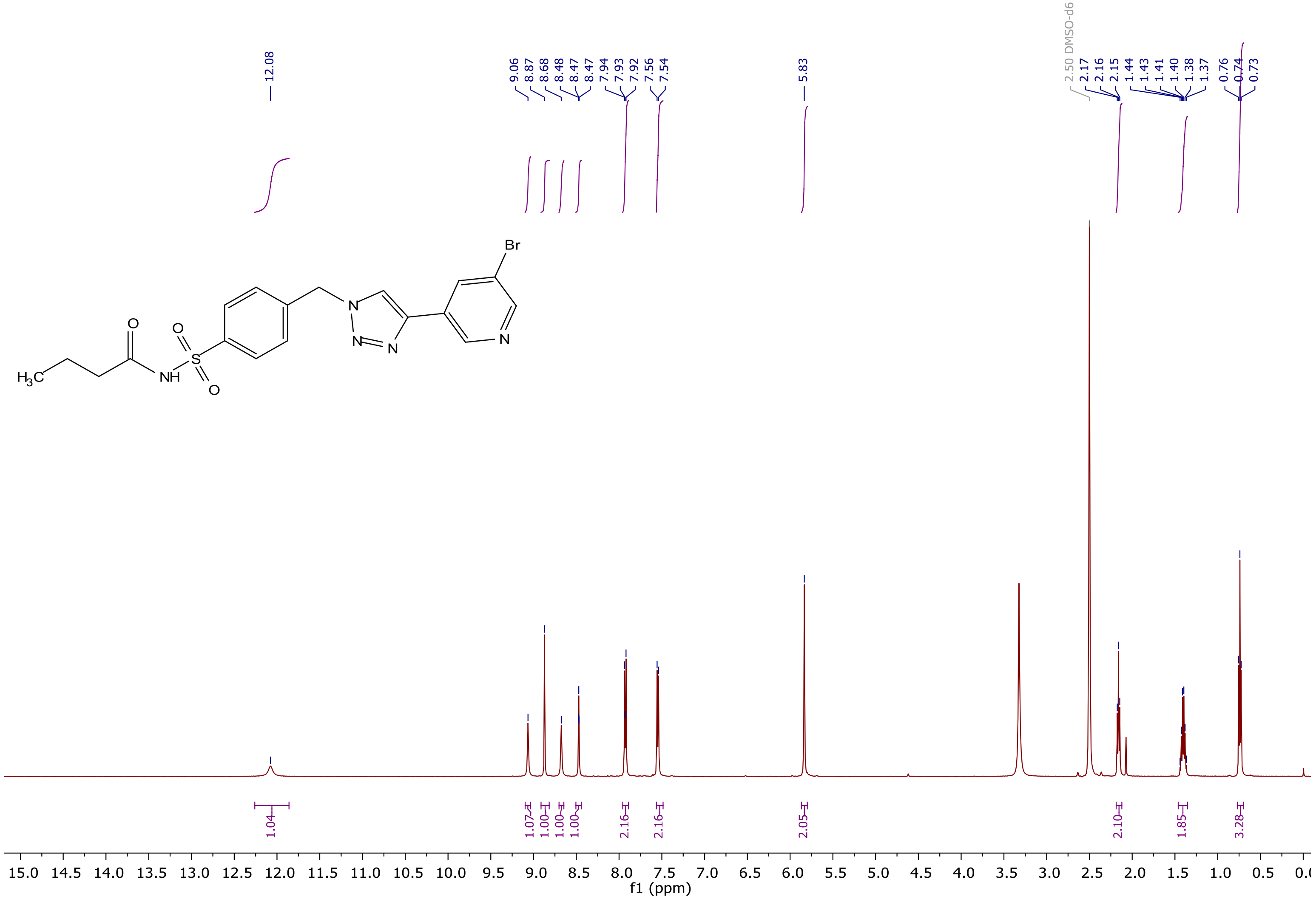

***N*-((4-((4-(5-Bromopyridin-3-yl)-1*H*-1,2,3-triazol-1-yl)methyl)phenyl)sulfonyl)butyramide (7{1,35,4}) ^{1}H NMR**

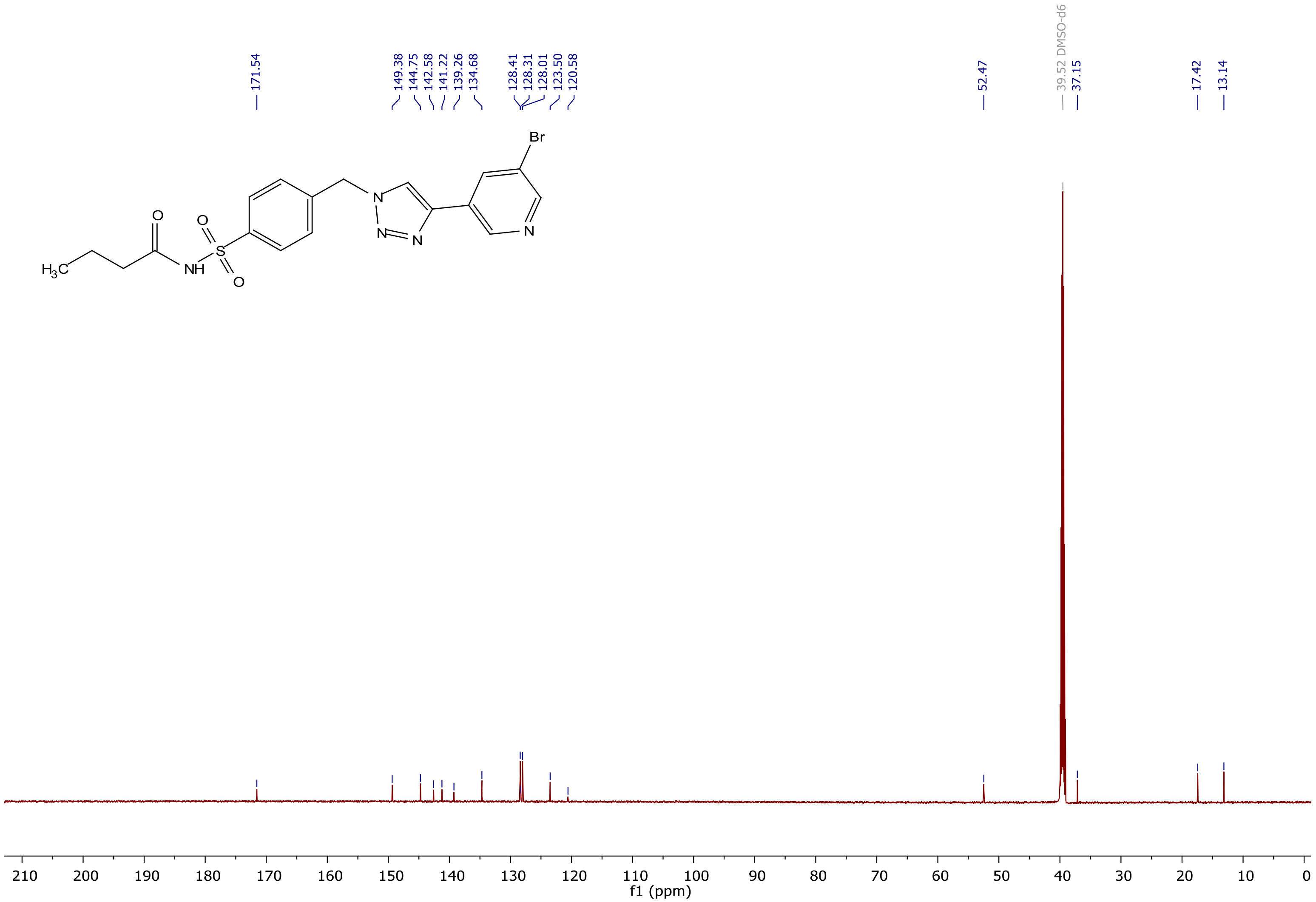

***N*-((4-((4-(5-Bromopyridin-3-yl)-1*H*-1,2,3-triazol-1-yl)methyl)phenyl)sulfonyl)butyramide (7{1,35,4}) ^{13}C NMR**

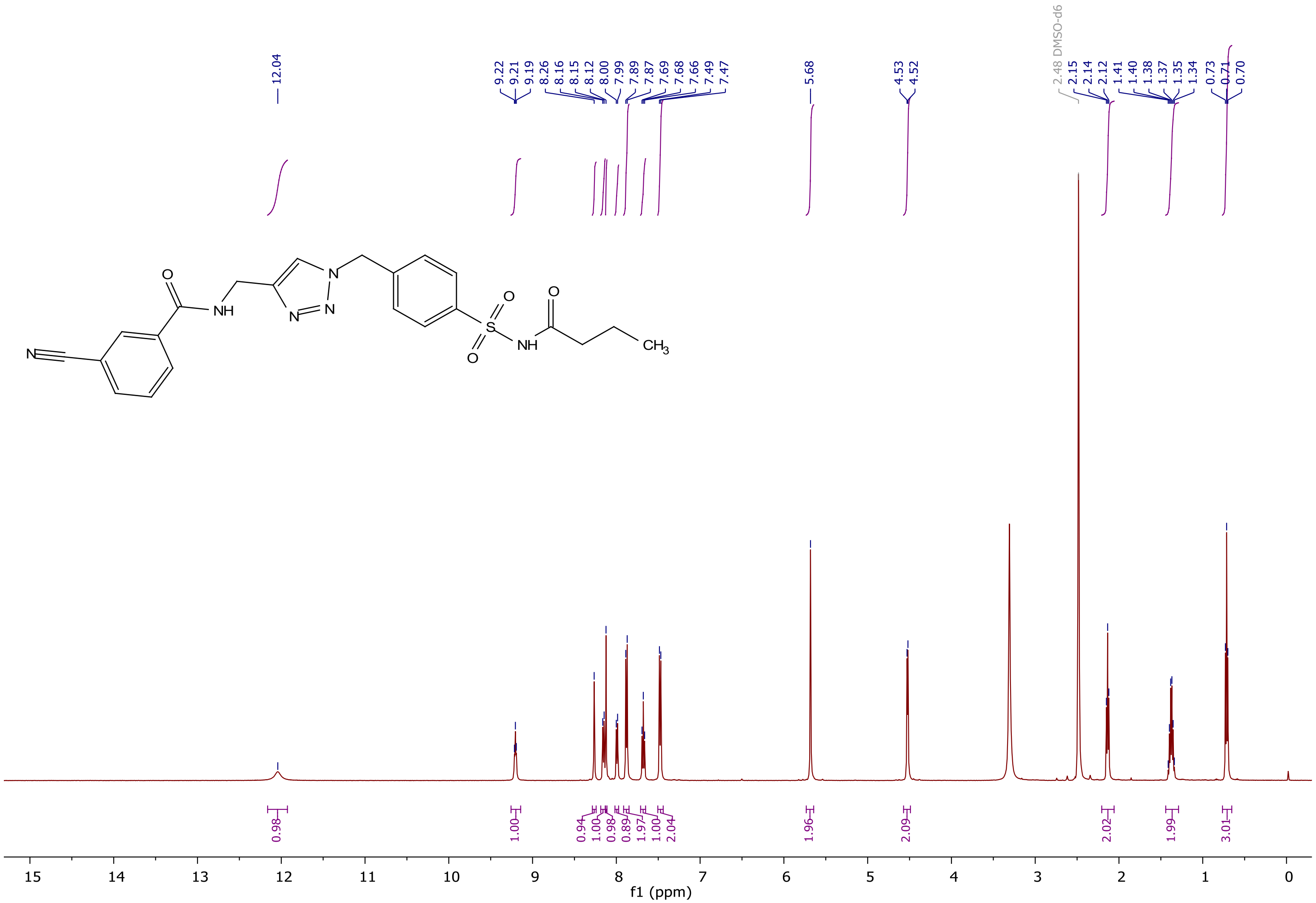

N-((1-(4-(N-Butyrylsulfamoyl)benzyl)-1H-1,2,3-triazol-4-yl)methyl)-3-cyanobenzamide (7{1,47,4}) ^{1}H NMR

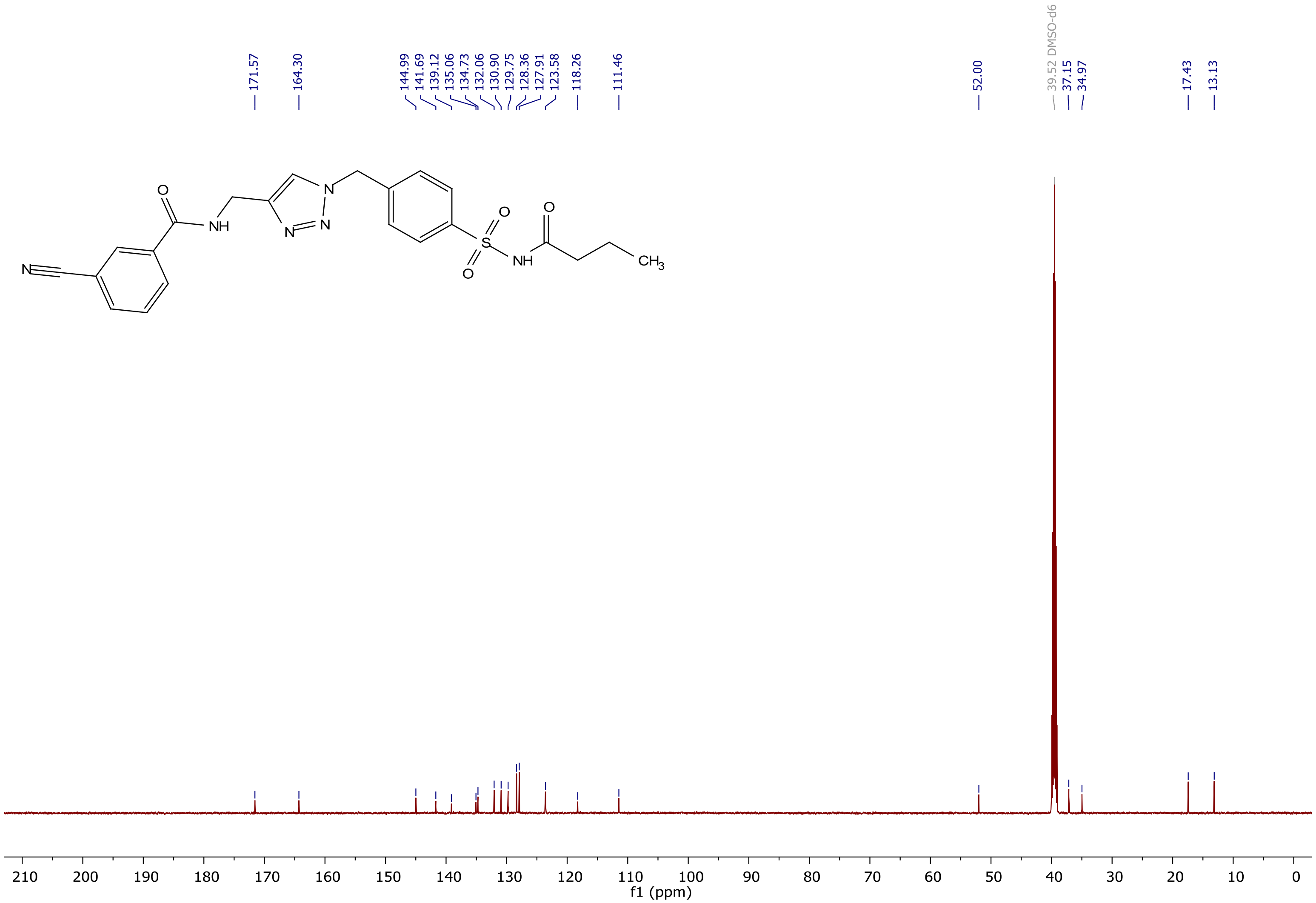

_N_-((1-(4-(_N_-Butyrylsulfamoyl)benzyl)-1_H_-1,2,3-triazol-4-yl)methyl)-3-cyanobenzamide (7{1,47,4}) ^{13}C NMR

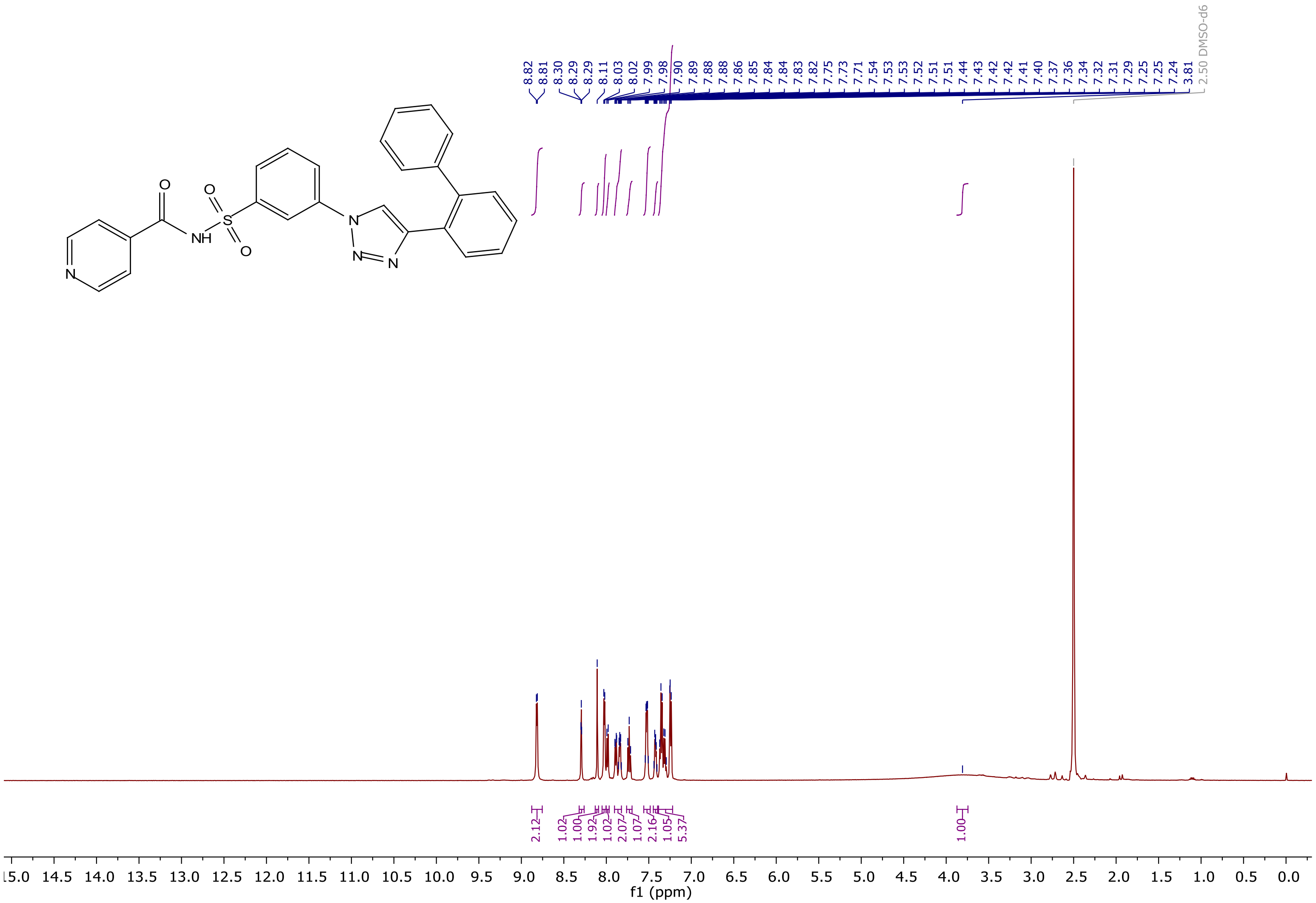

N-((3-(4-([1,1'-Biphenyl]-2-yl)-1H-1,2,3-triazol-1-yl)phenyl)sulfonyl)isonicotinamide (7{2,18,50}) ^{1}H NMR

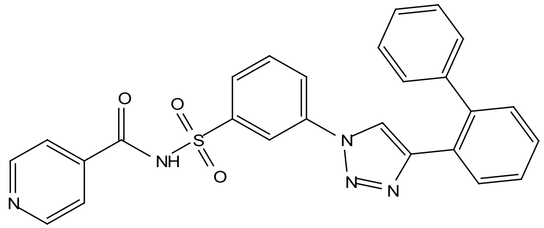

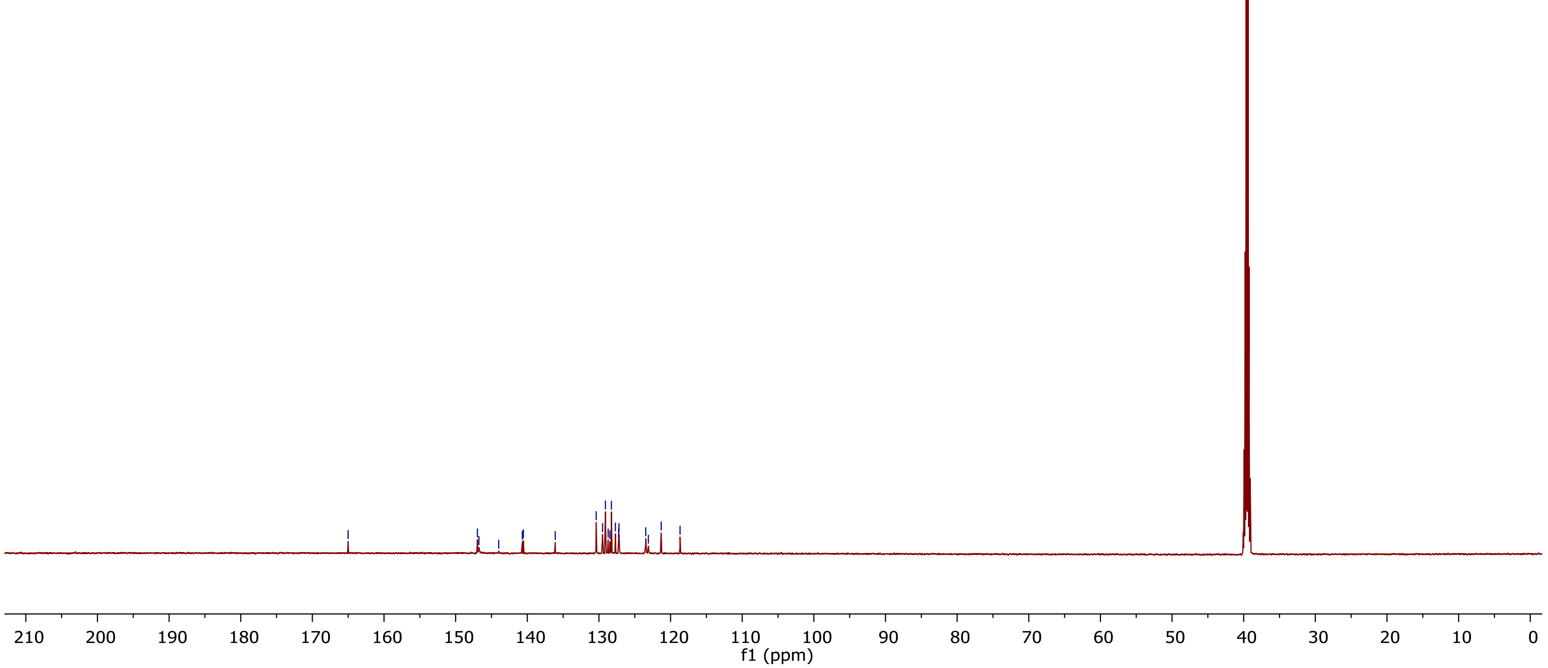

***N*-((3-(4-([1,1'-Biphenyl]-2-yl)-1*H*-1,2,3-triazol-1-yl)phenyl)sulfonyl)isonicotinamide (7{2,18,50}) ^{13}C NMR**

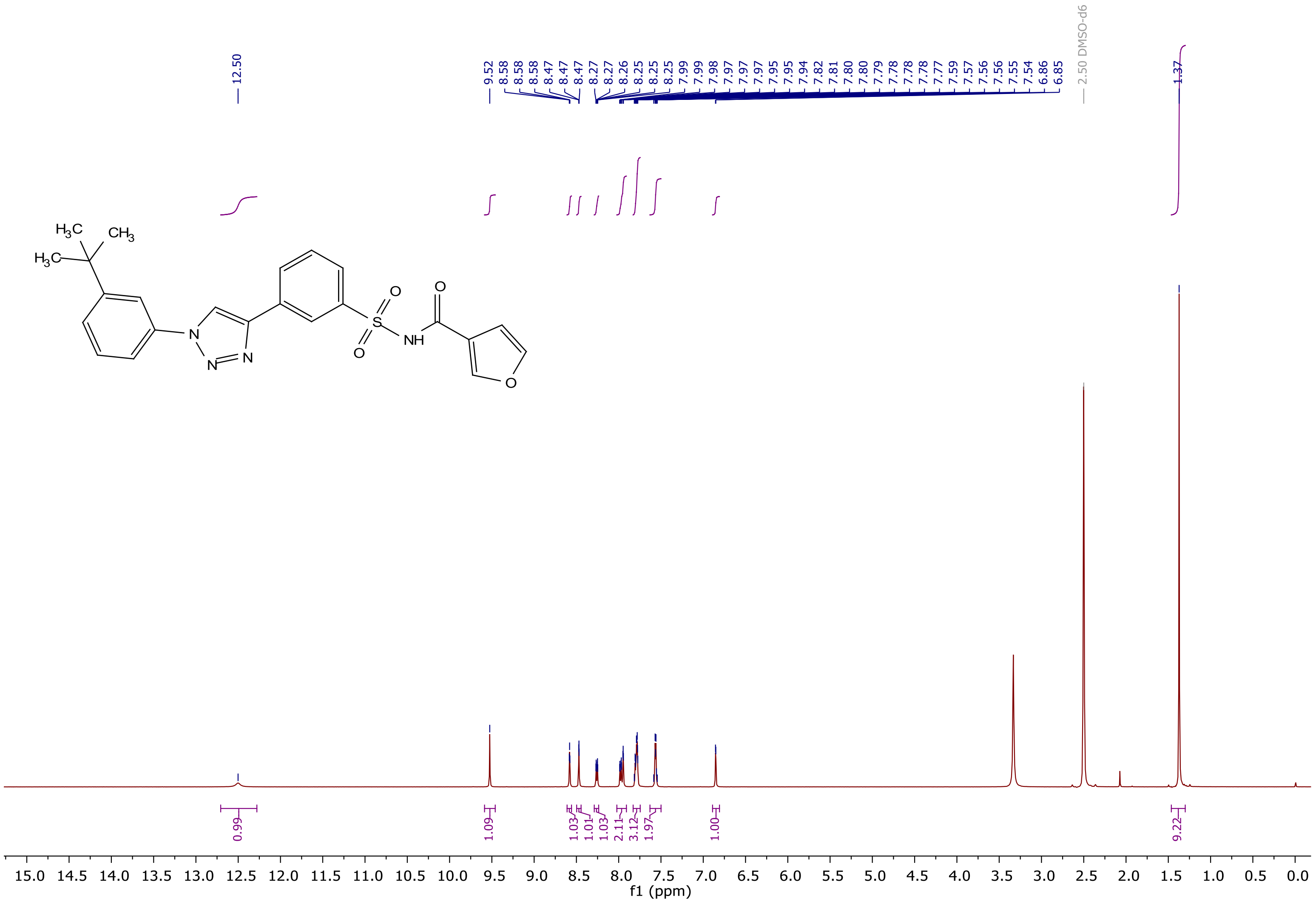

***N*-((3-(1-(3-(*tert*-Butyl)phenyl)-1*H*-1,2,3-triazol-4-yl)phenyl)sulfonyl)furan-3-carboxamide (4{10,6,48}) ^{1}H NMR**

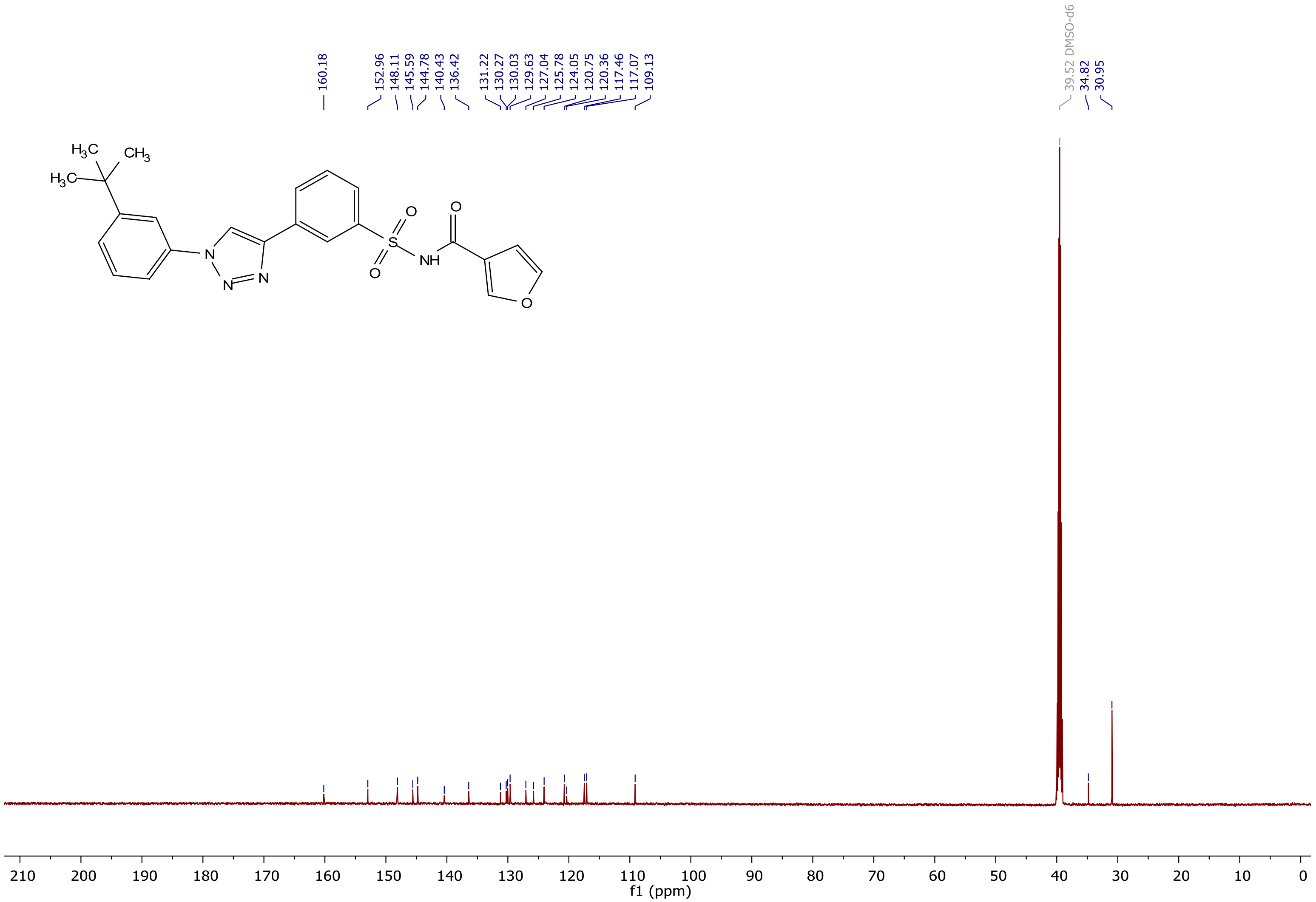

N-((3-(1-(3-(*tert*-Butyl)phenyl)-1H-1,2,3-triazol-4-yl)phenyl)sulfonyl)furan-3-carboxamide (4{10,6,48}) ^{13}C NMR

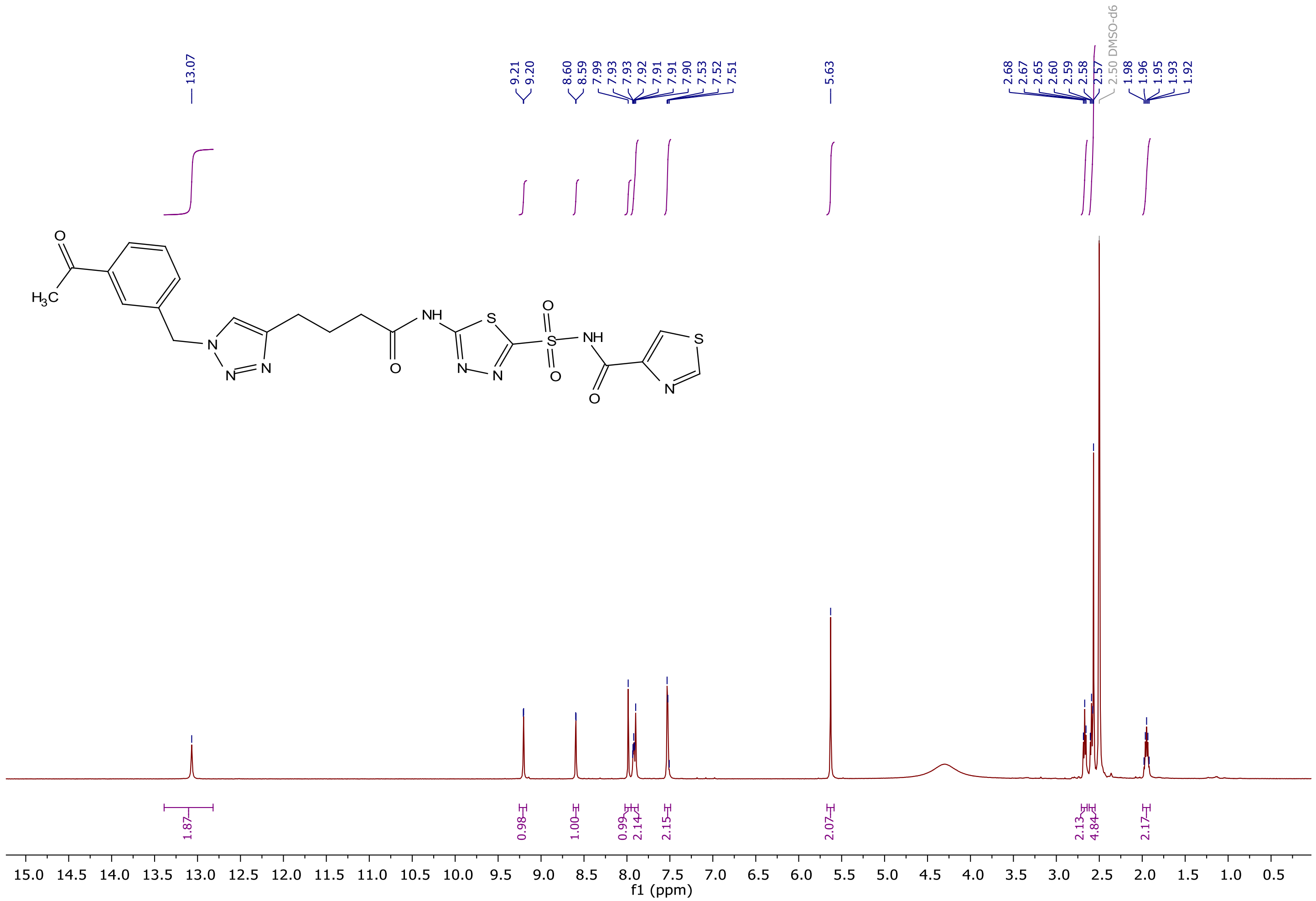

***N*-((5-(4-(1-(3-Acetylbenzyl)-1*H*-1,2,3-triazol-4-yl)butanamido)-1,3,4-thiadiazol-2-yl)sulfonyl)thiazole-4-carboxamide (4{11,5,80}) ^{1}H NMR**

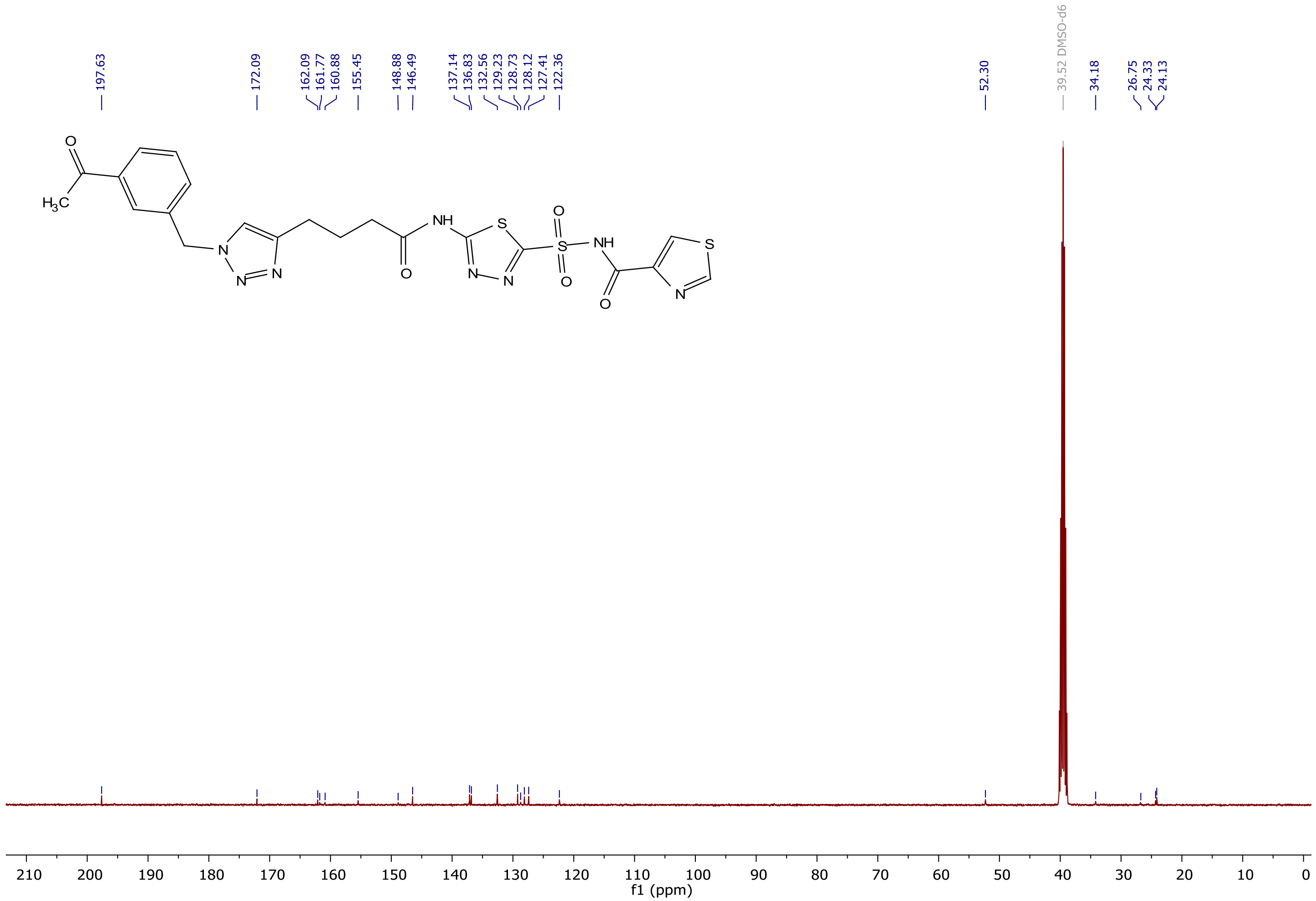

_N_-((5-(4-(1-(3-Acetylbenzyl)-1_H_-1,2,3-triazol-4-yl)butanamido)-1,3,4-thiadiazol-2-yl)sulfonyl)thiazole-4-carboxamide (4{11,5,80}) ^{13}C NMR

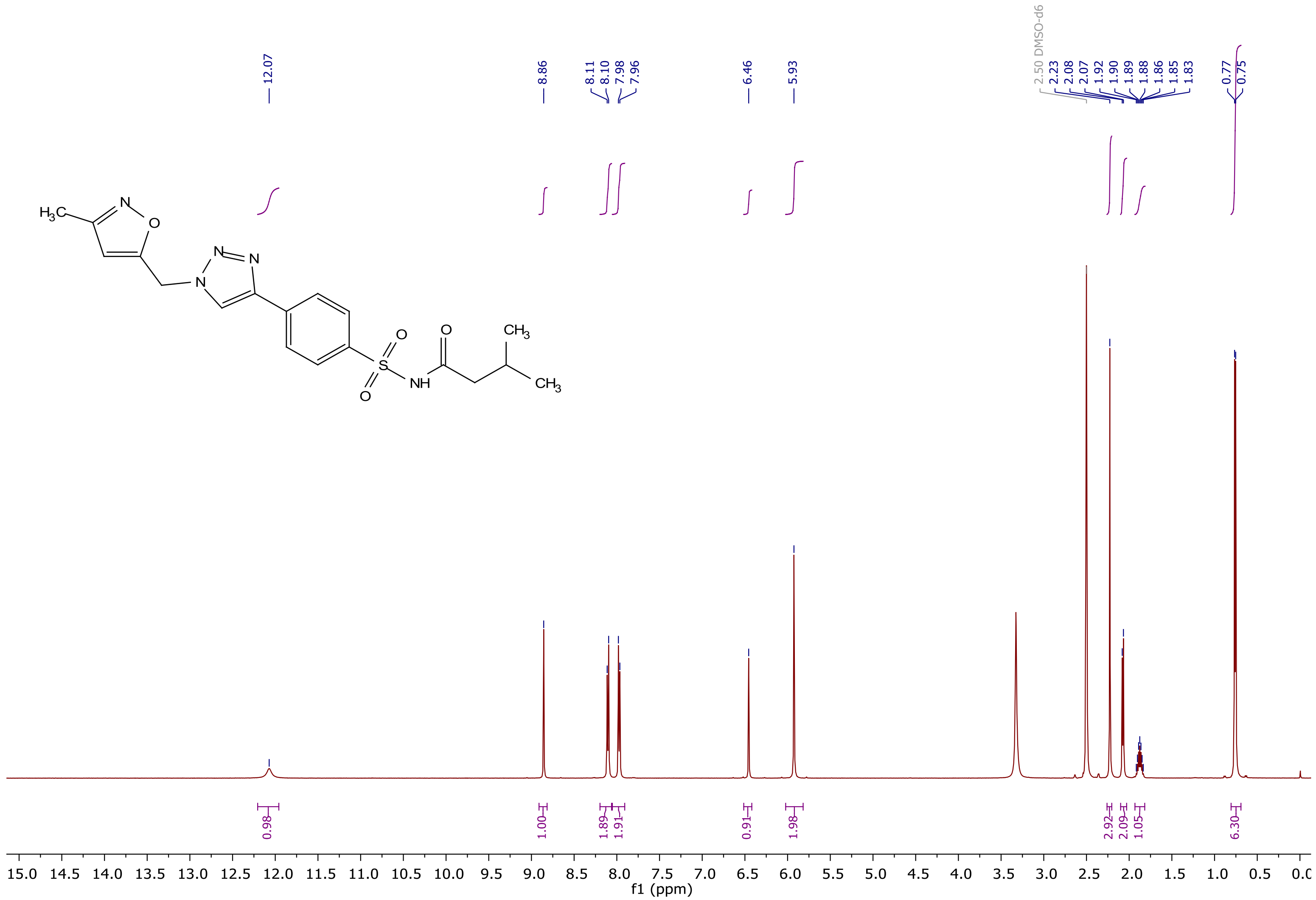

3-Methyl-*N*-((4-(1-((3-methylisoxazol-5-yl)methyl)-1*H*-1,2,3-triazol-4-yl)phenyl)sulfonyl)butanamide (4{20,7,16}) ^{1}H NMR

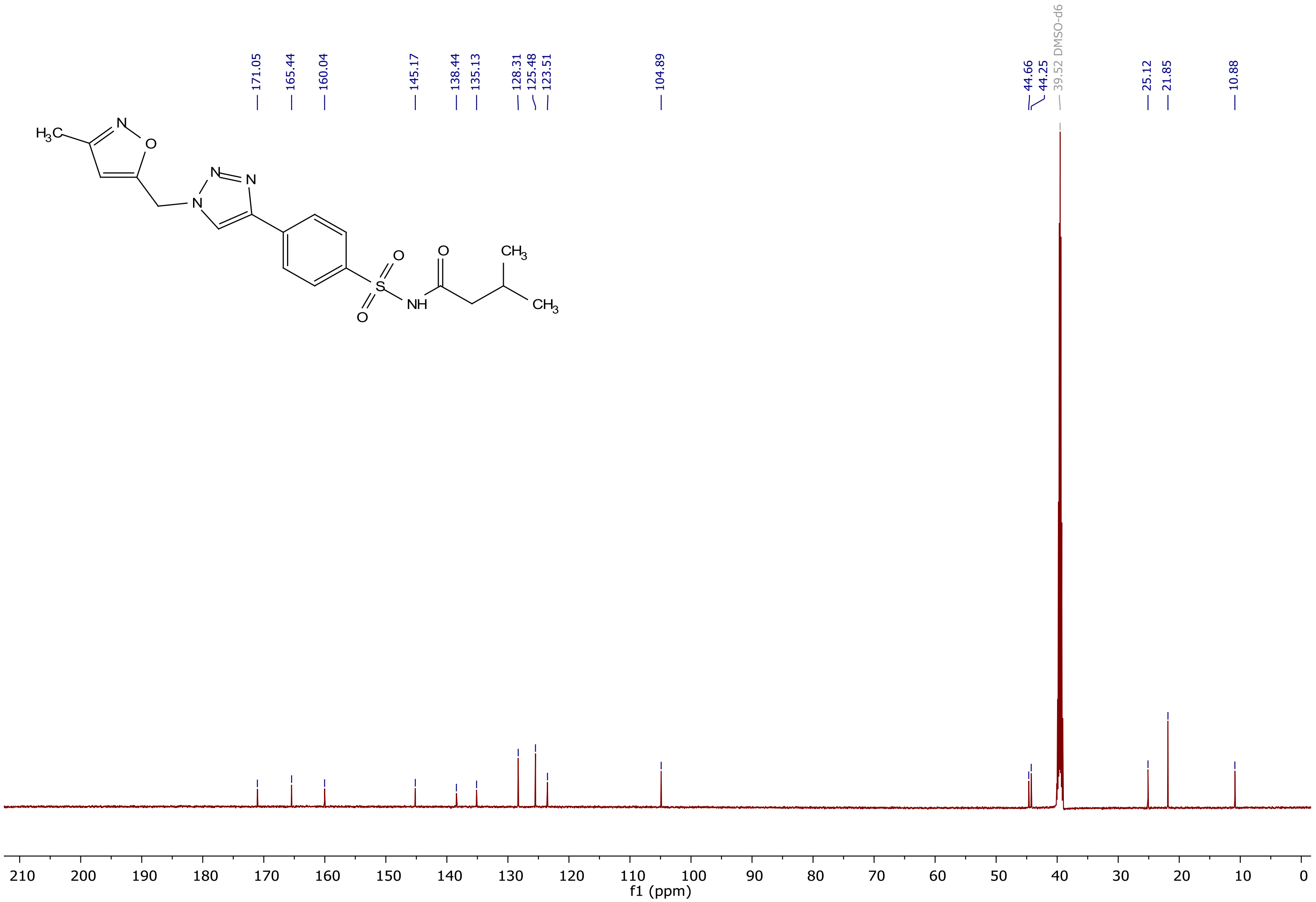

3-Methyl-*N*-((4-(1-((3-methylisoxazol-5-yl)methyl)-1*H*-1,2,3-triazol-4-yl)phenyl)sulfonyl)butanamide (4{20,7,16}) ^{13}C NMR

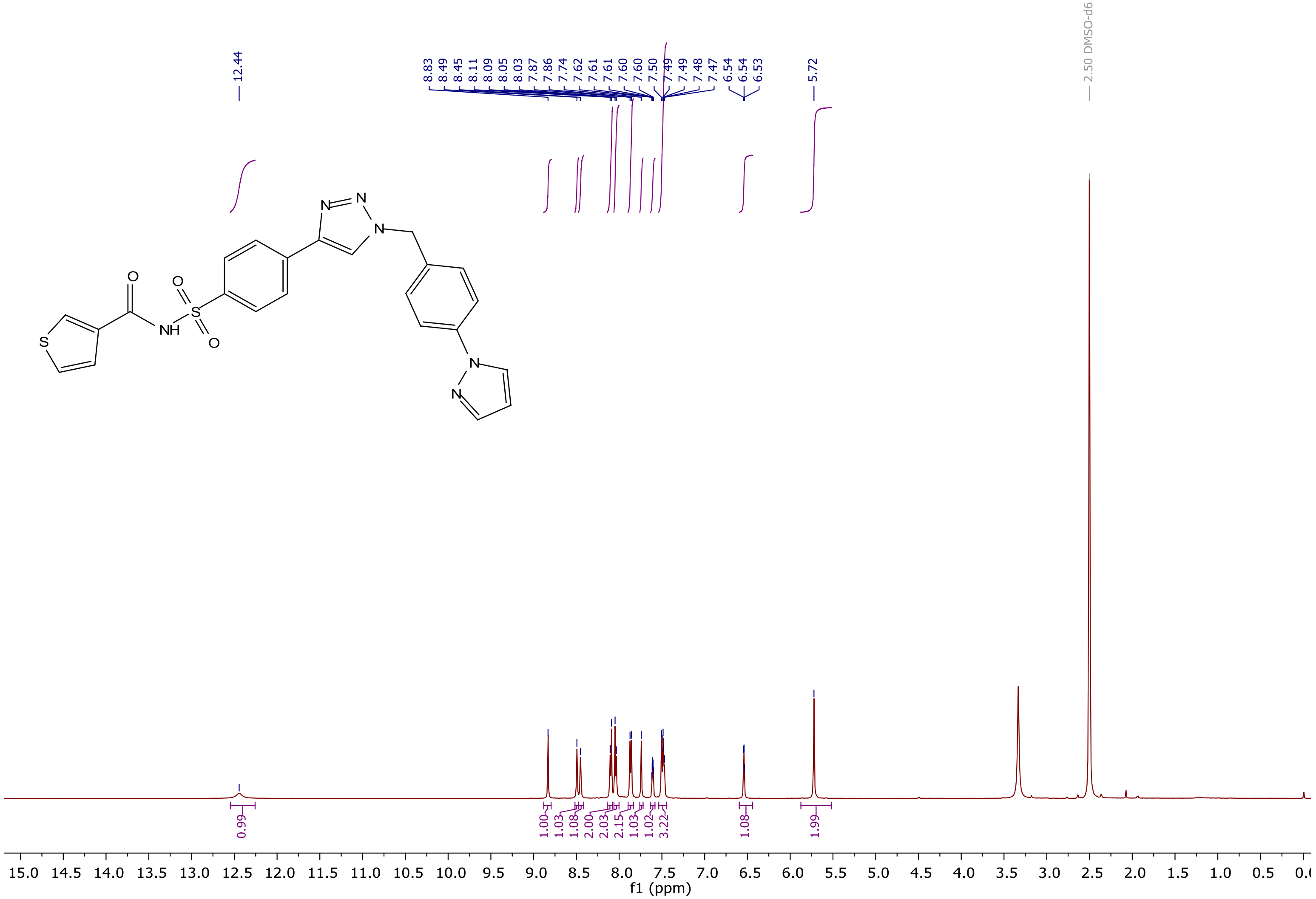

***N*-((4-(1-(4-(1*H*-Pyrazol-1-yl)benzyl)-1*H*-1,2,3-triazol-4-yl)phenyl)sulfonyl)thiophene-3-carboxamide (4{18,7,32}) ^{1}H NMR**

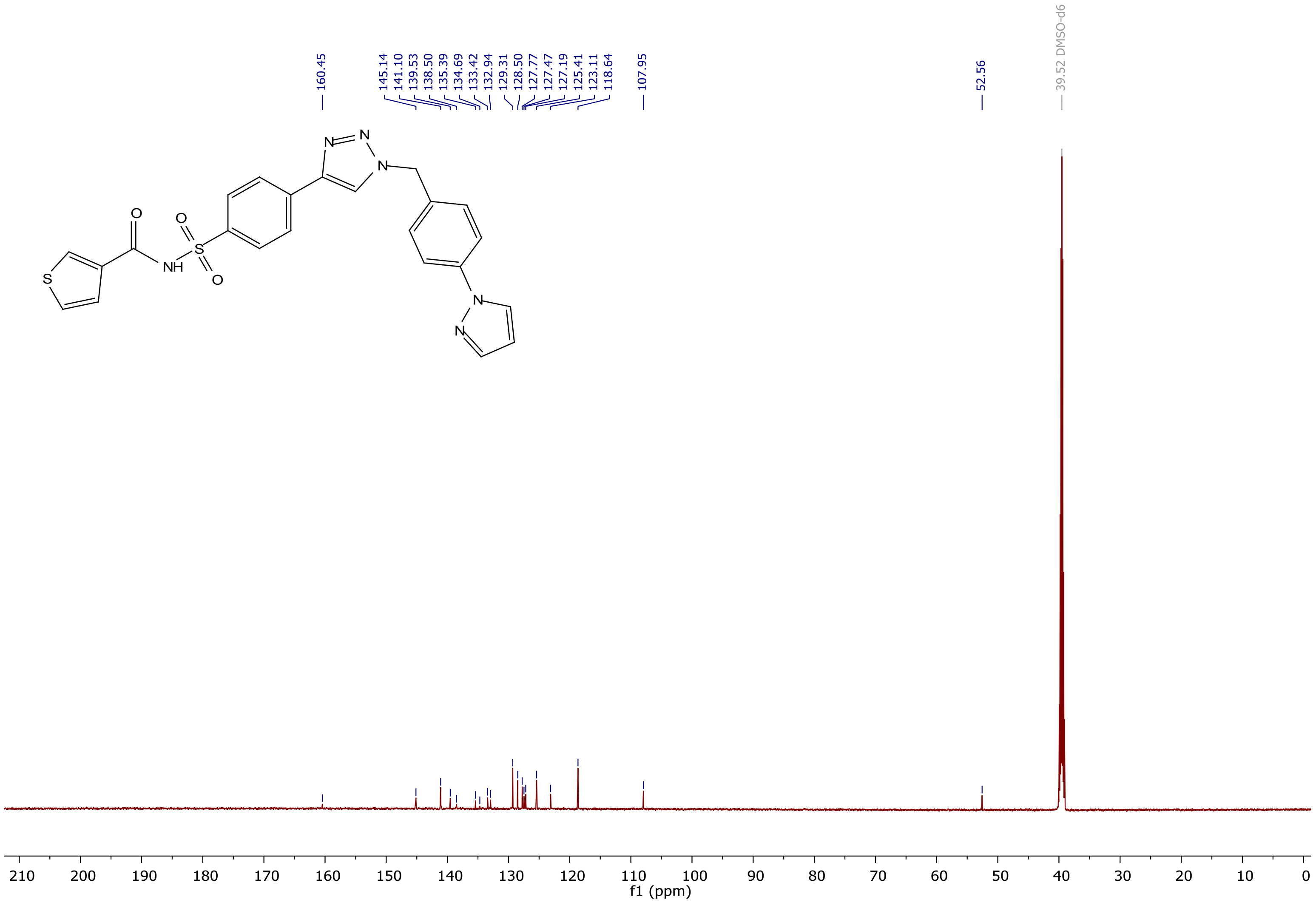

***N*-((4-(1-(4-(1*H*-Pyrazol-1-yl)benzyl)-1*H*-1,2,3-triazol-4-yl)phenyl)sulfonyl)thiophene-3-carboxamide (4{18,7,32})** ^{13}**C NMR**

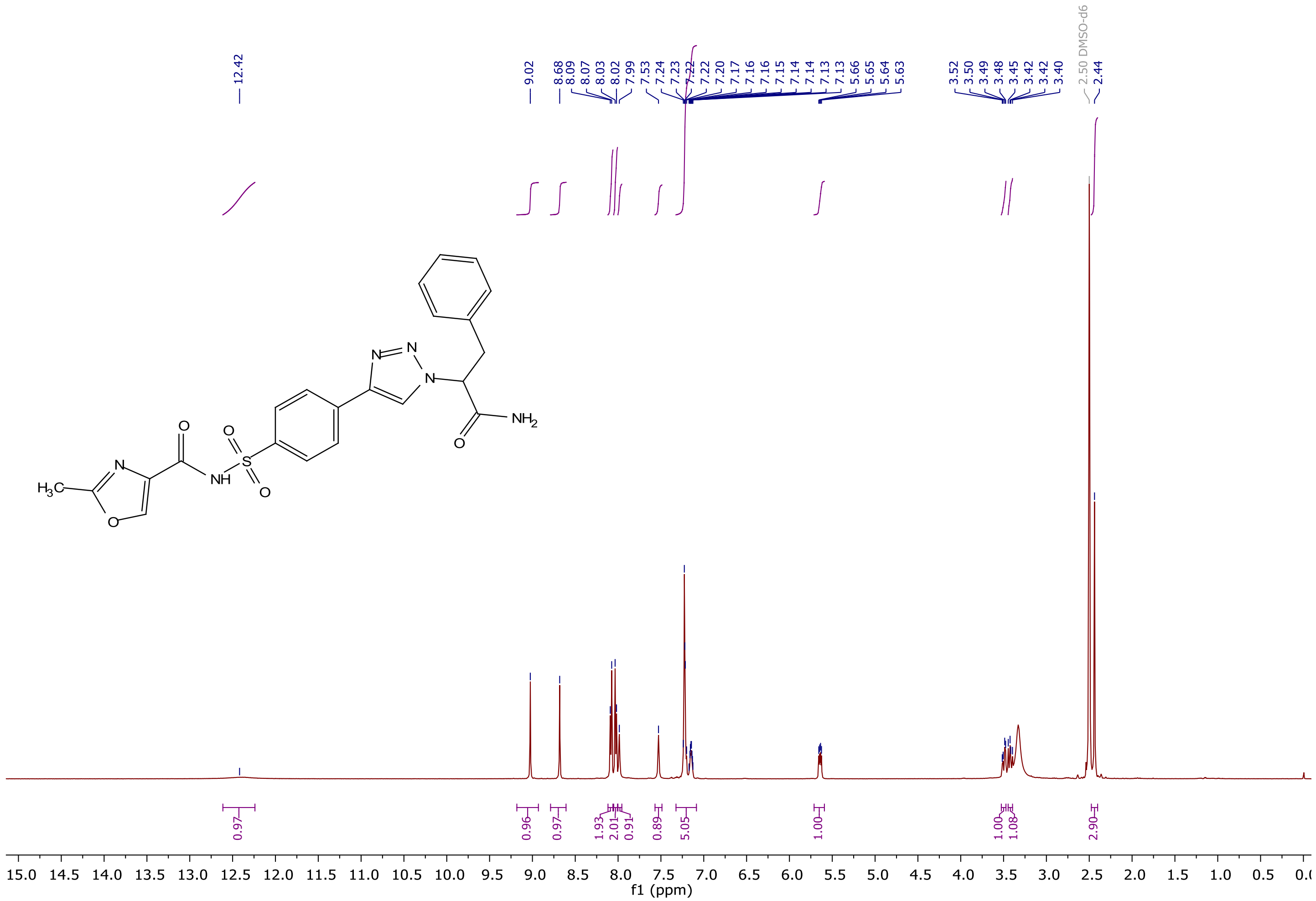

***N*-((4-(1-(1-Amino-1-oxo-3-phenylpropan-2-yl)-1*H*-1,2,3-triazol-4-yl)phenyl)sulfonyl)-2-methyloxazole-4-carboxamide (4{21,7,33}) ^{1}H NMR**

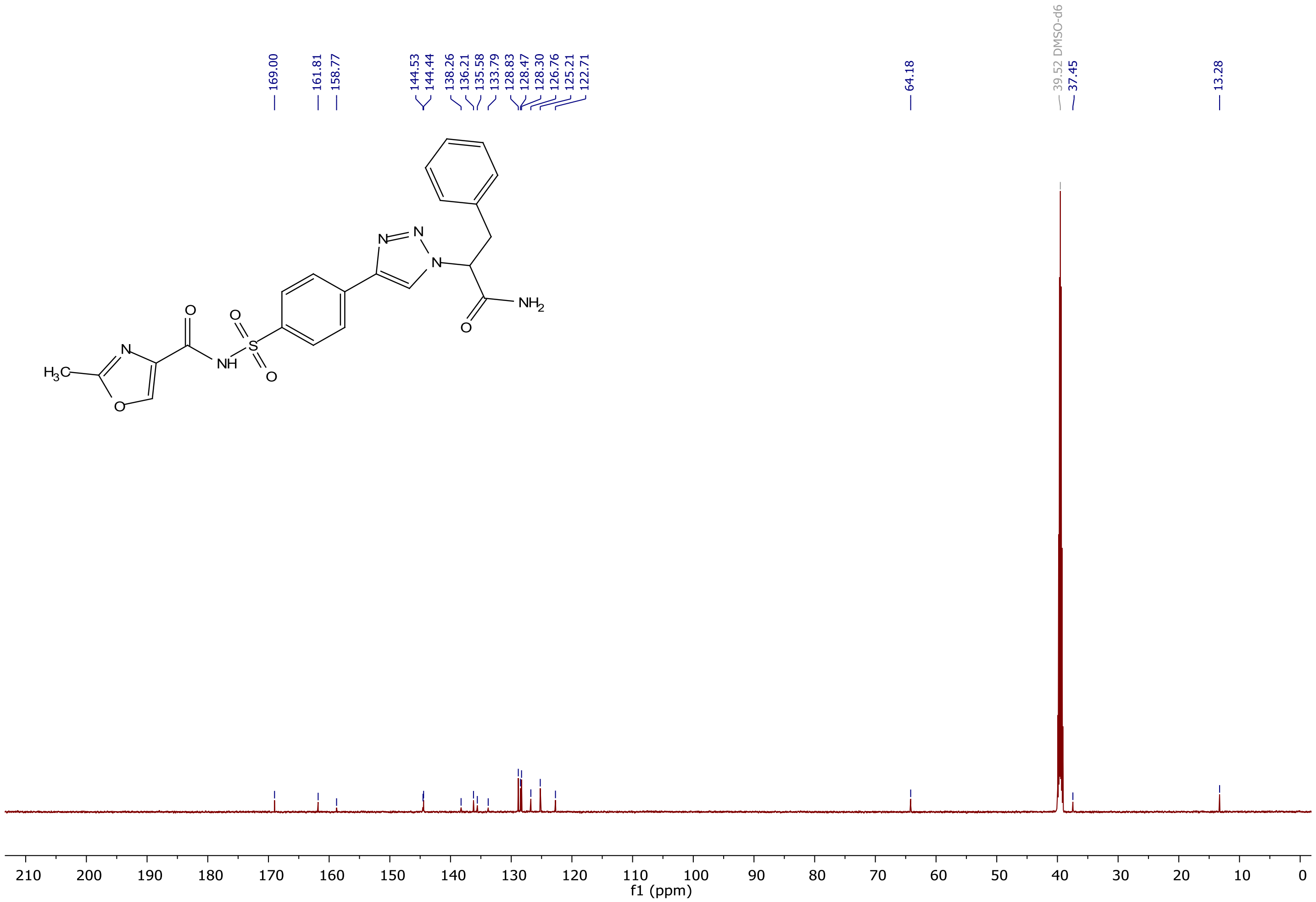

***N*-((4-(1-(1-Amino-1-oxo-3-phenylpropan-2-yl)-1*H*-1,2,3-triazol-4-yl)phenyl)sulfonyl)-2-methyloxazole-4-carboxamide (4{21,7,33}) ^{13}C NMR**

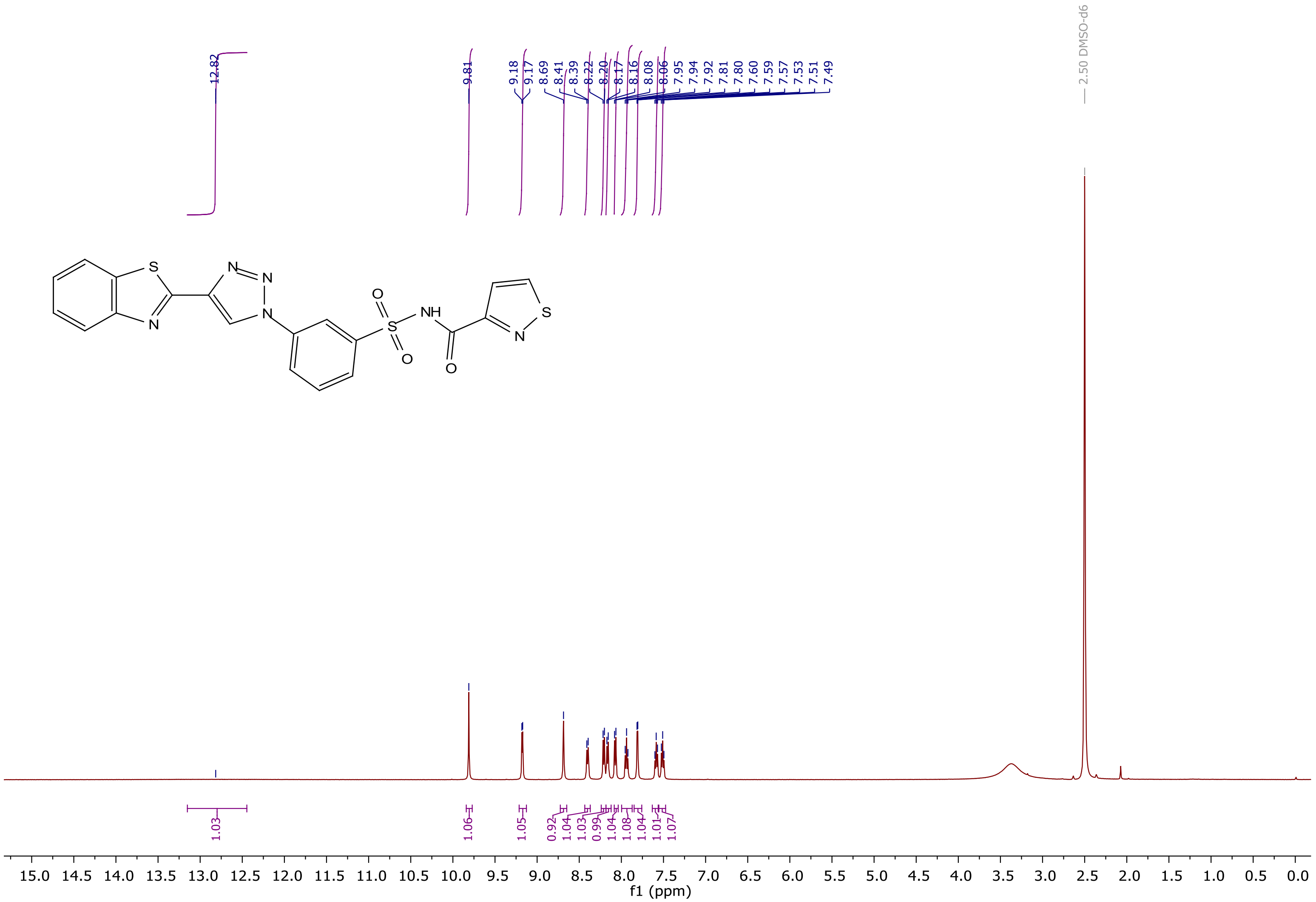

***N*-((3-(4-(Benzo[*d*]thiazol-2-yl)-1*H*-1,2,3-triazol-1-yl)phenyl)sulfonyl)isothiazole-3-carboxamide (7{2,37,72}) ^{1}H NMR**

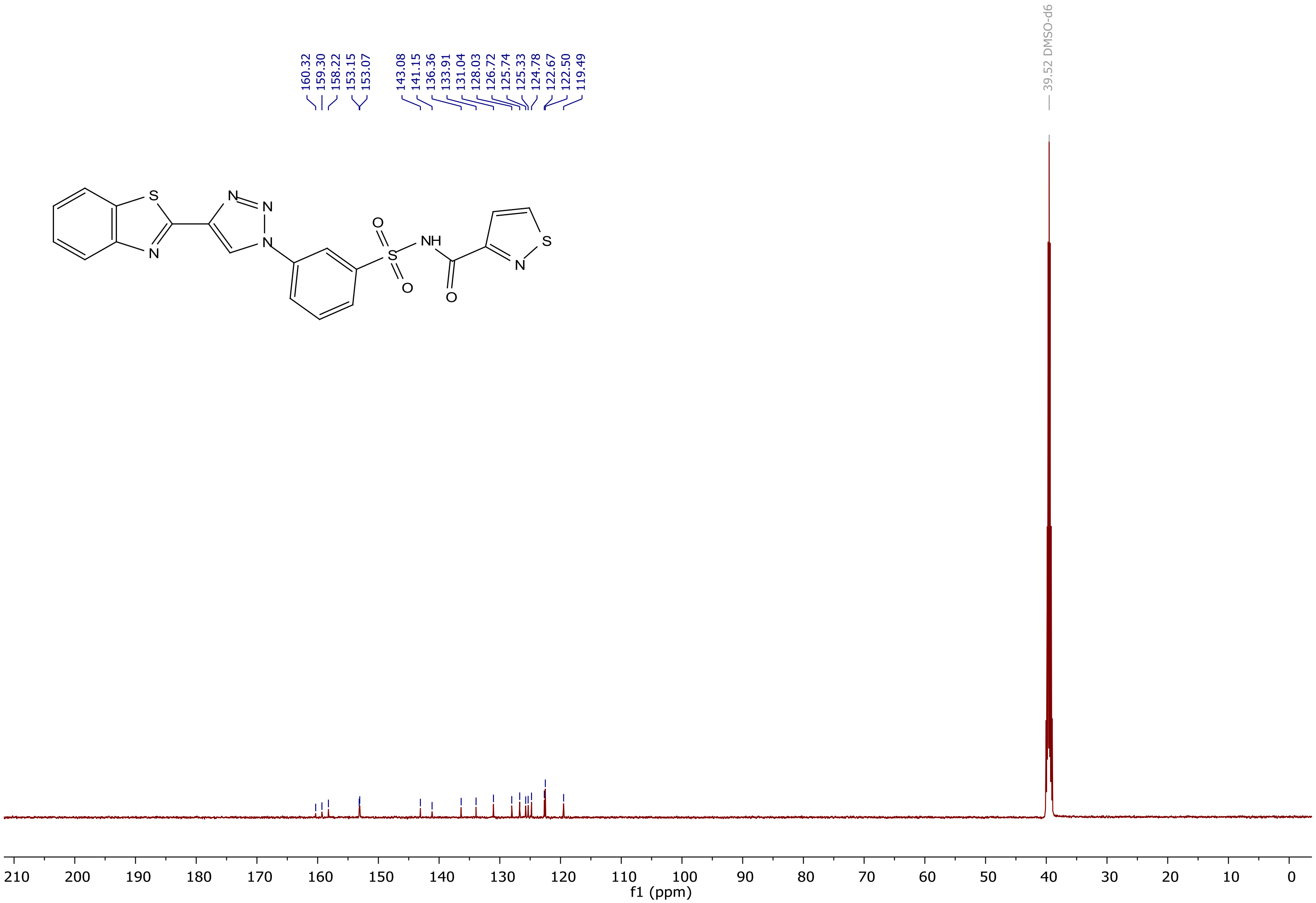

***N*-((3-(4-(Benzo[*d*]thiazol-2-yl)-1*H*-1,2,3-triazol-1-yl)phenyl)sulfonyl)isothiazole-3-carboxamide (7{2,37,72}) ^{13}C NMR**

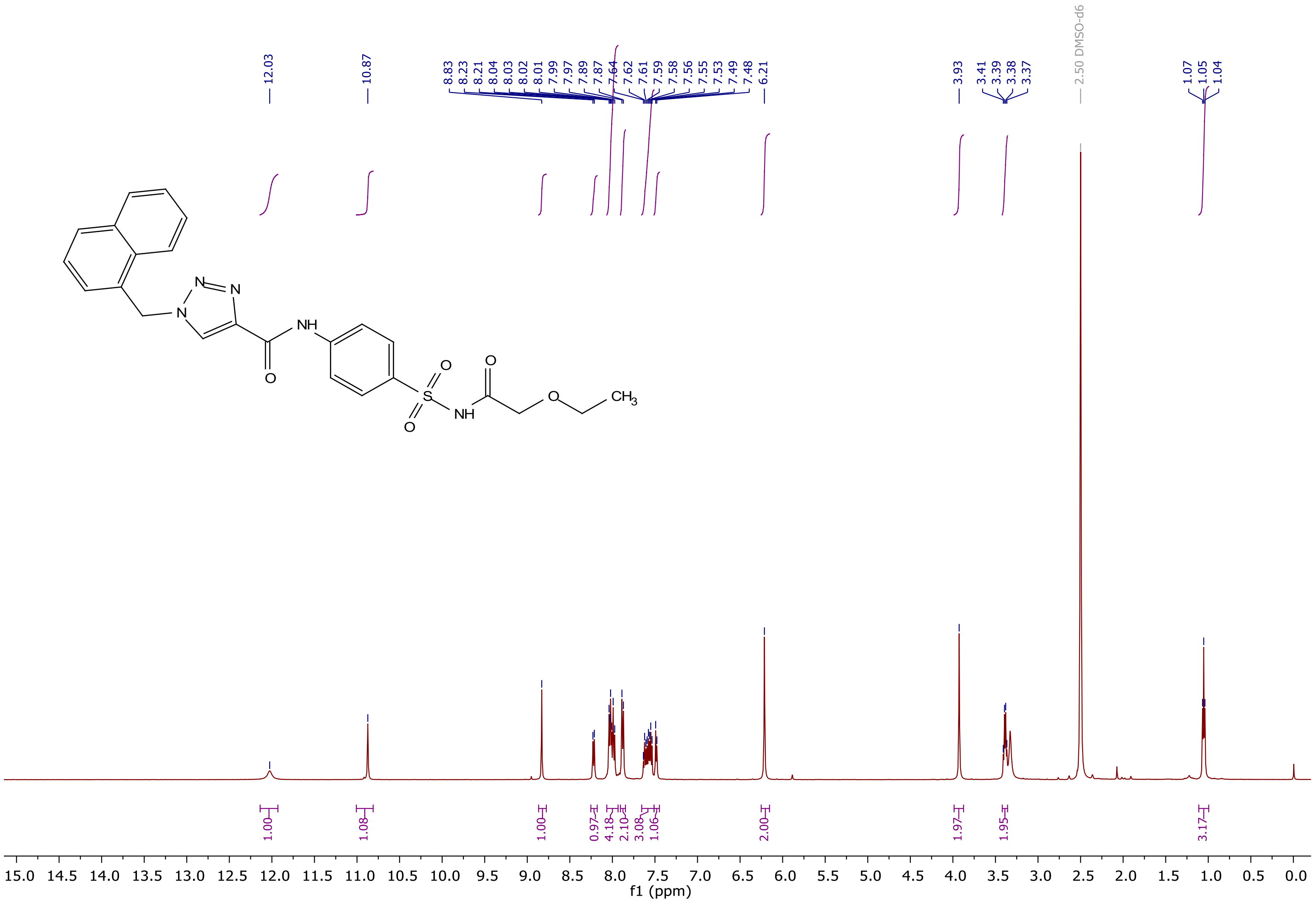

***N*-(4-(*N*-(2-Ethoxyacetyl)sulfamoyl)phenyl)-1-(naphthalen-1-ylmethyl)-1*H*-1,2,3-triazole-4-carboxamide (4{2,8,1}) ^{1}H NMR**

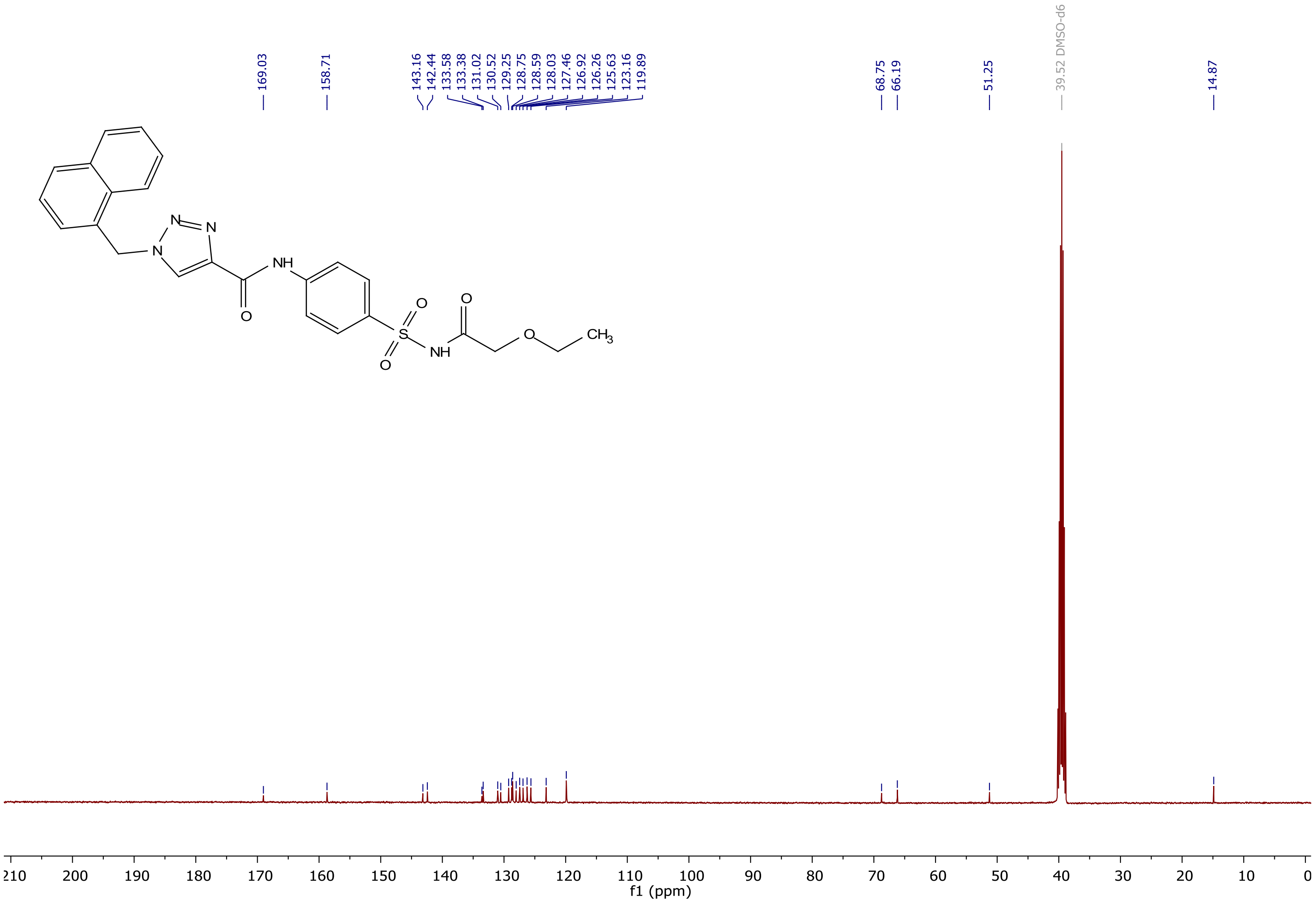

***N*-(4-(*N*-(2-Ethoxyacetyl)sulfamoyl)phenyl)-1-(naphthalen-1-ylmethyl)-1*H*-1,2,3-triazole-4-carboxamide (4{2,8,1}) ^{13}C NMR**

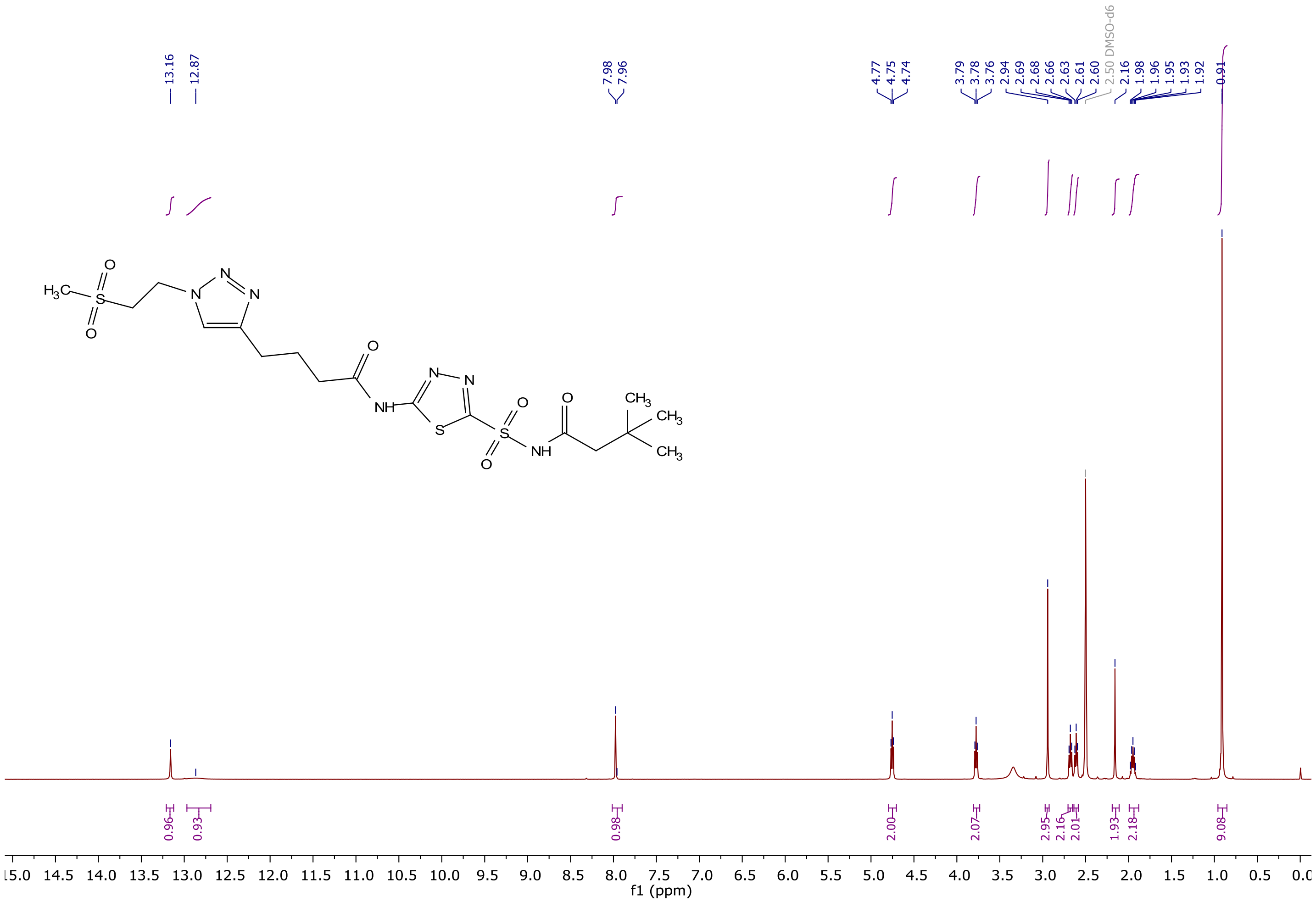

3,3-Dimethyl-*N*-((5-(4-(1-(2-(methylsulfonyl)ethyl)-1*H*-1,2,3-triazol-4-yl)butanamido)-1,3,4-thiadiazol-2-yl)sulfonyl)butanamide (4{45,5,81}) ^{1}H NMR

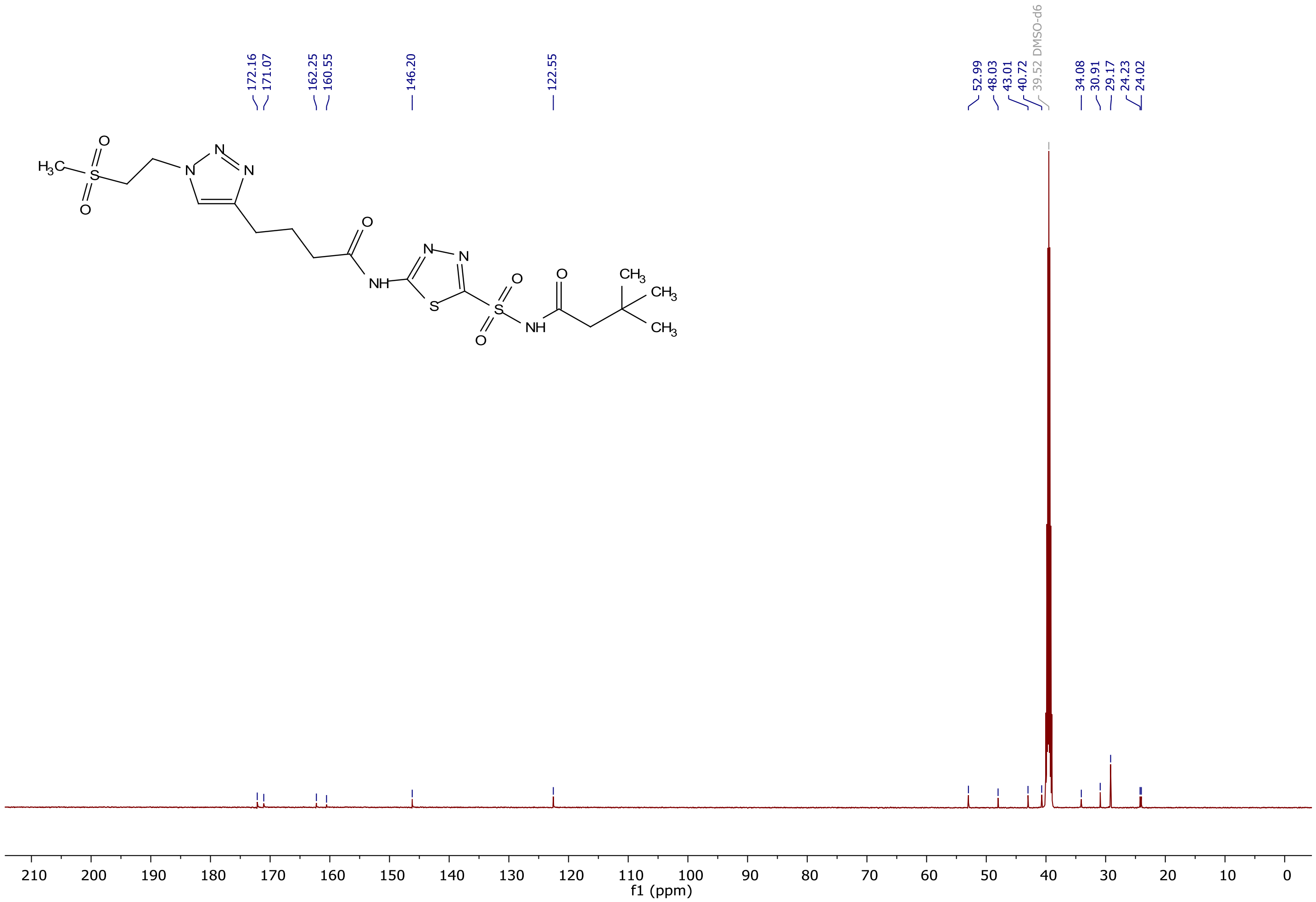

3,3-Dimethyl-*N*-((5-(4-(1-(2-(methylsulfonyl)ethyl)-1*H*-1,2,3-triazol-4-yl)butanamido)-1,3,4-thiadiazol-2-yl)sulfonyl)butanamide (4{45,5,81}) ^{13}C NMR

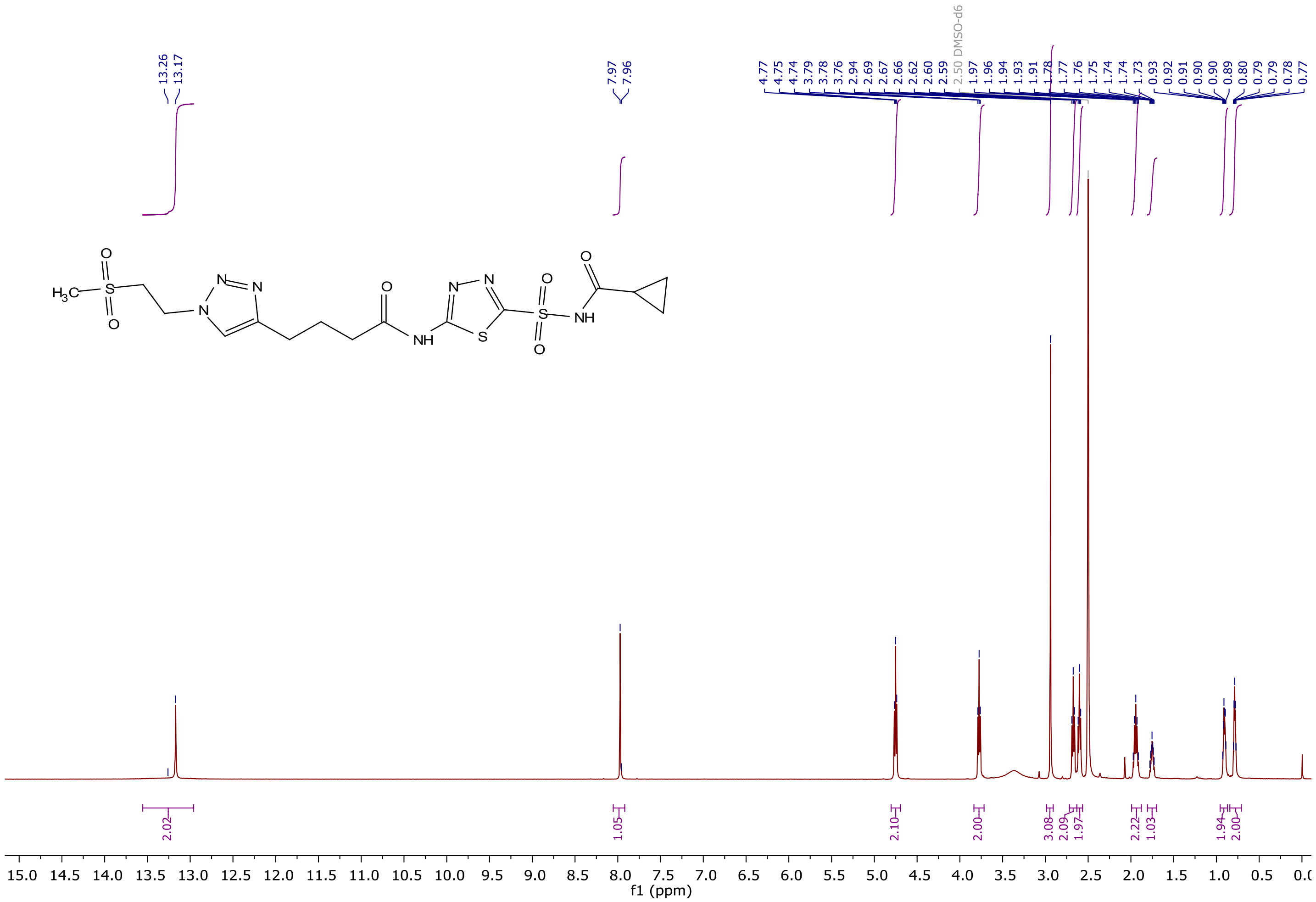

***N*-((5-(4-(1-(2-(Methylsulfonyl)ethyl)-1*H*-1,2,3-triazol-4-yl)butanamido)-1,3,4-thiadiazol-2-yl)sulfonyl)cyclopropanecarboxamide (4{45,5,2}) ^{1}H NMR**

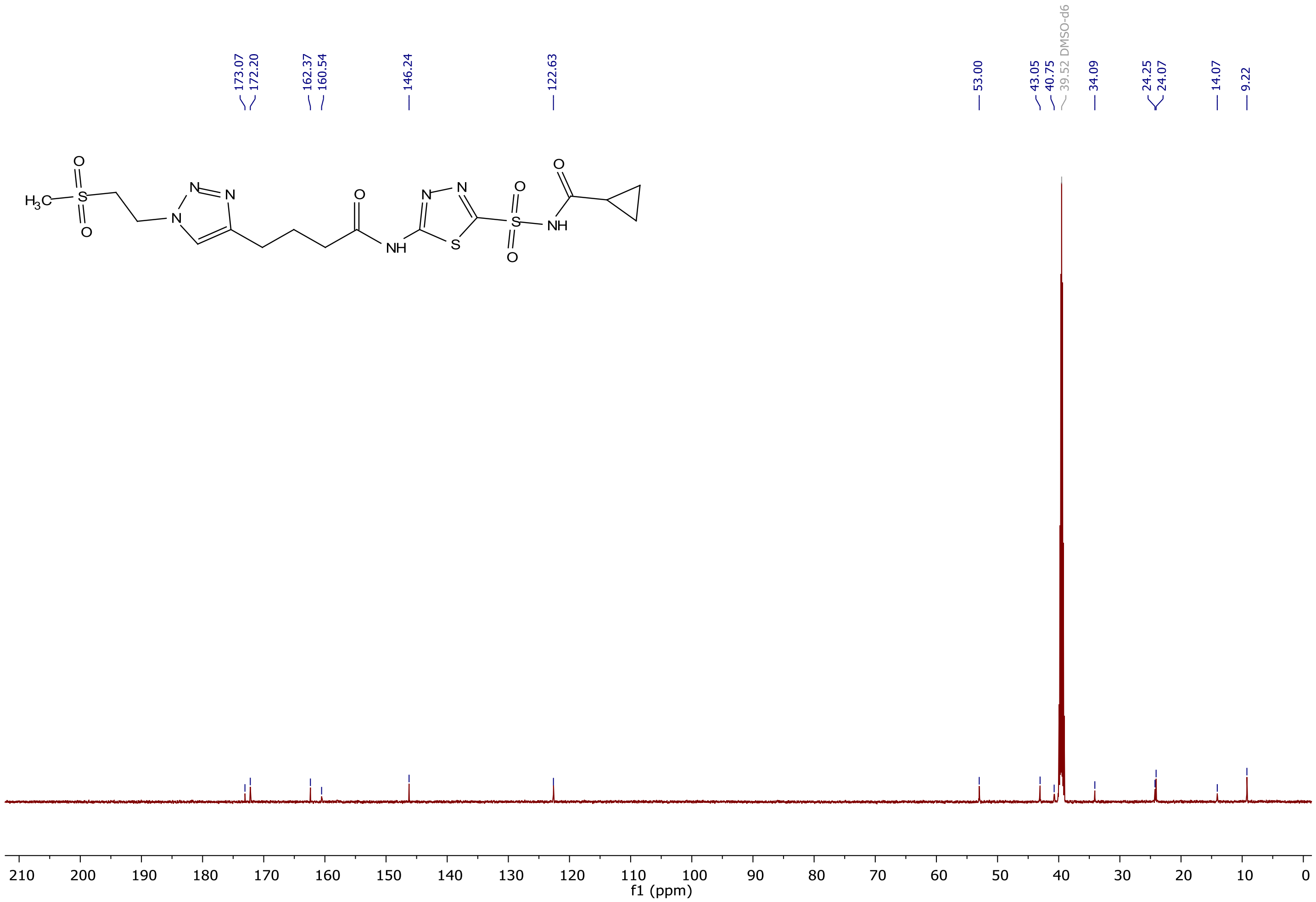

***N*-((5-(4-(1-(2-(Methylsulfonyl)ethyl)-1*H*-1,2,3-triazol-4-yl)butanamido)-1,3,4-thiadiazol-2-yl)sulfonyl)cyclopropanecarboxamide (4{45,5,2}) ^{13}C NMR**

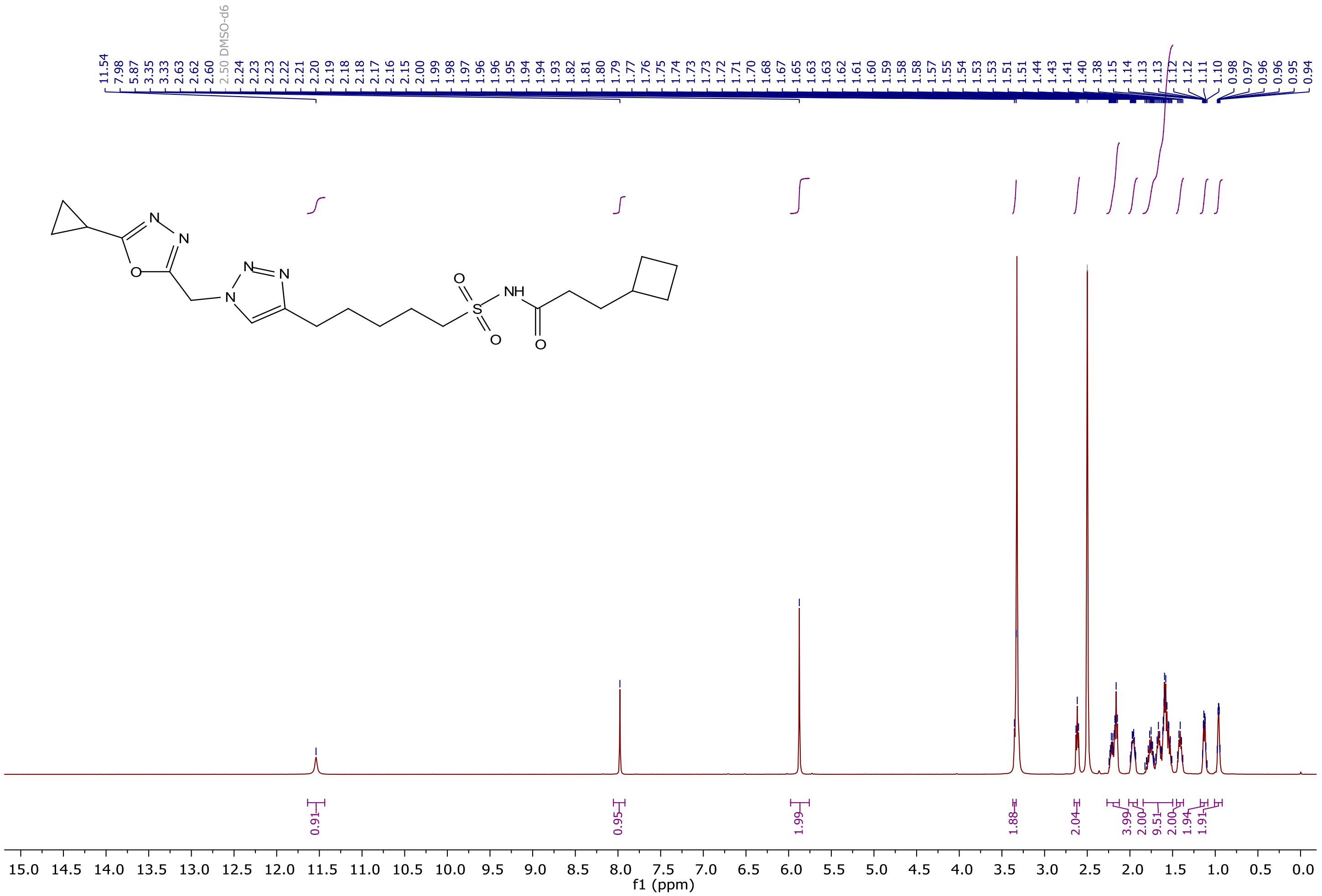

3-Cyclobutyl-*N*-((5-(1-((5-cyclopropyl-1,3,4-oxadiazol-2-yl)methyl)-1*H*-1,2,3-triazol-4-yl)pentyl)sulfonyl)propanamide (4{51,4,75}) ^{1}**H NMR**

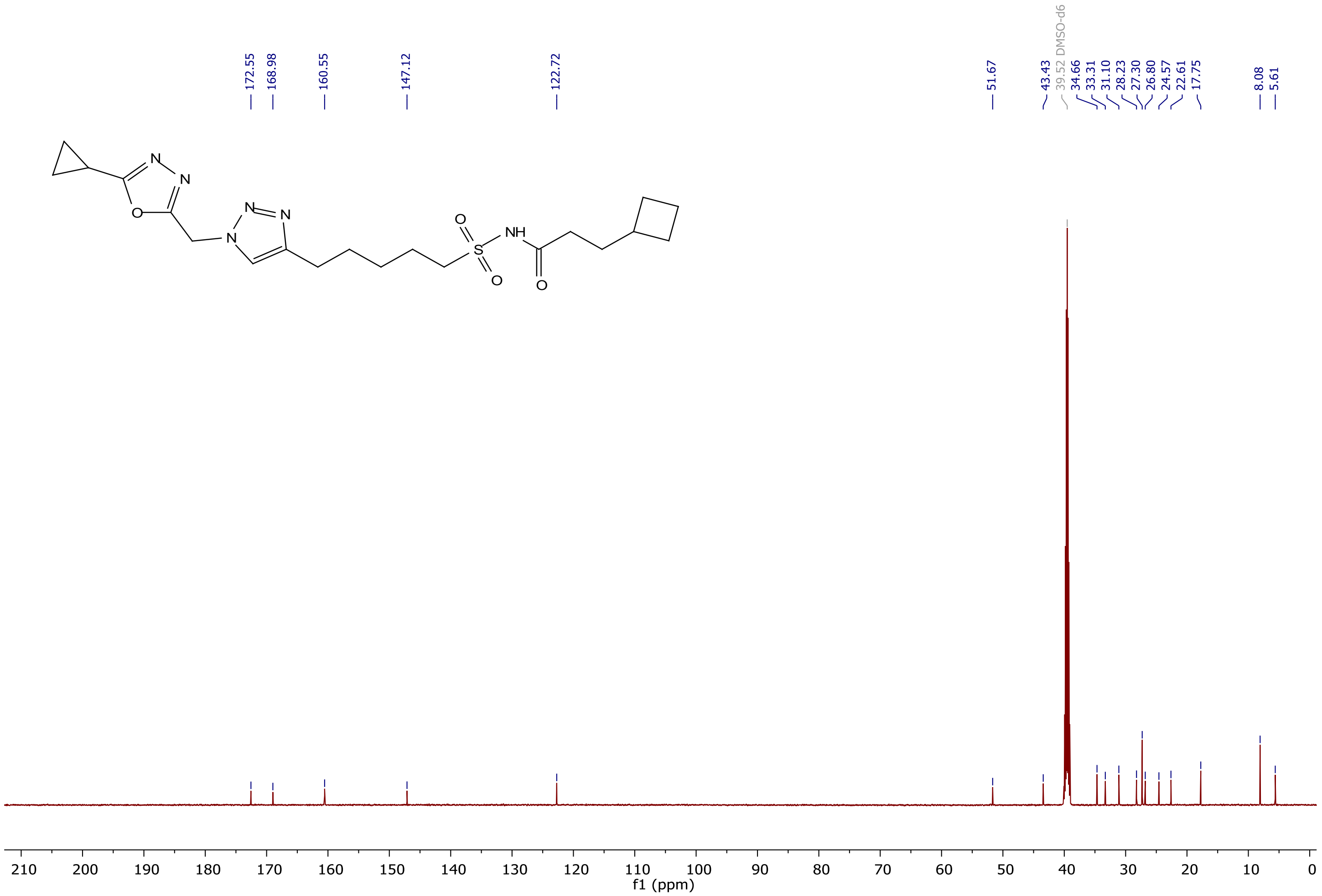

3-Cyclobutyl-*N*-((5-(1-((5-cyclopropyl-1,3,4-oxadiazol-2-yl)methyl)-1*H*-1,2,3-triazol-4-yl)pentyl)sulfonyl)propanamide (4{51,4,75}) ^{13}C NMR

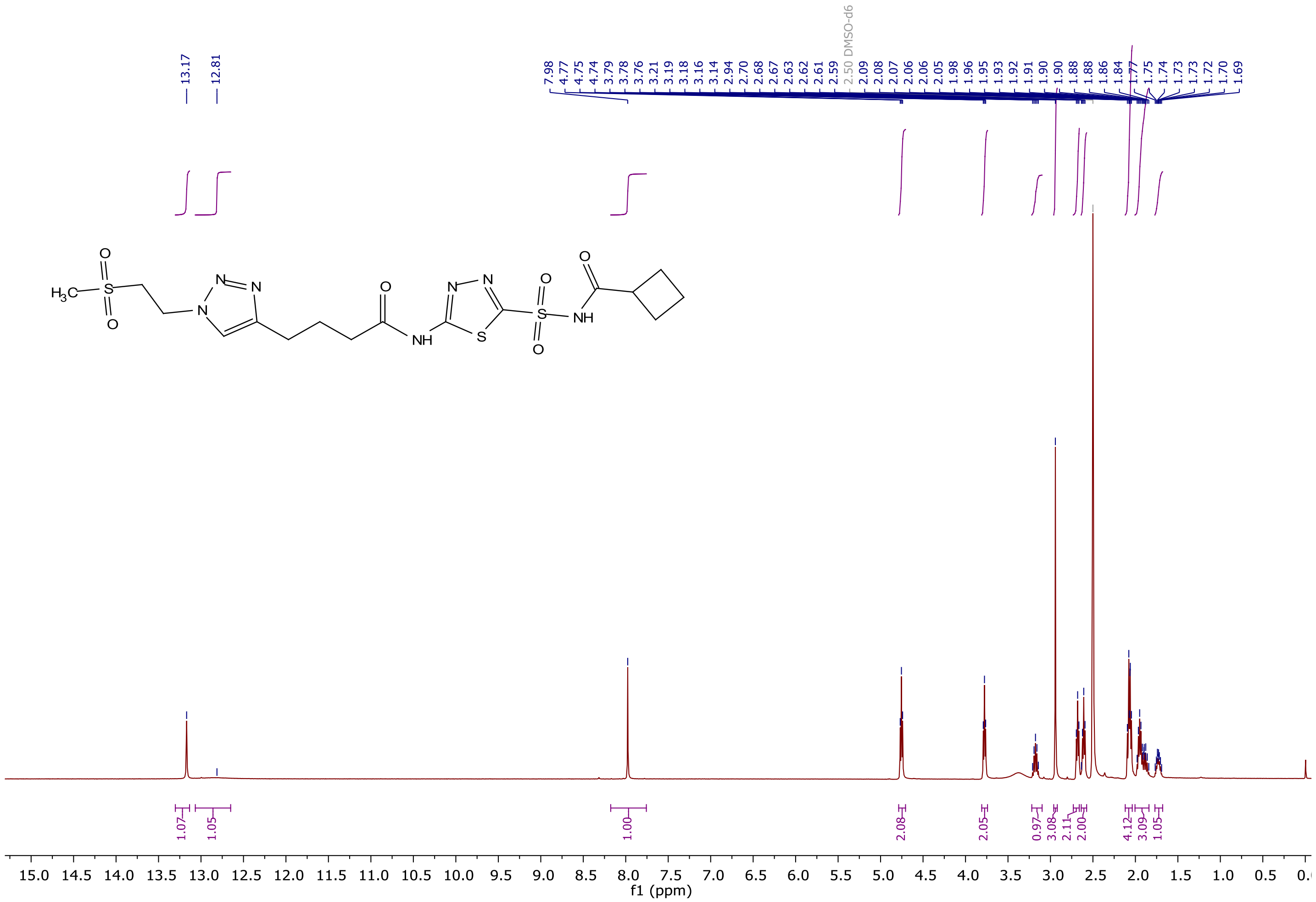

***N*-((5-(4-(1-(2-(Methylsulfonyl)ethyl)-1*H*-1,2,3-triazol-4-yl)butanamido)-1,3,4-thiadiazol-2-yl)sulfonyl)cyclobutanecarboxamide (4{45,5,78}) ^{1}H NMR**

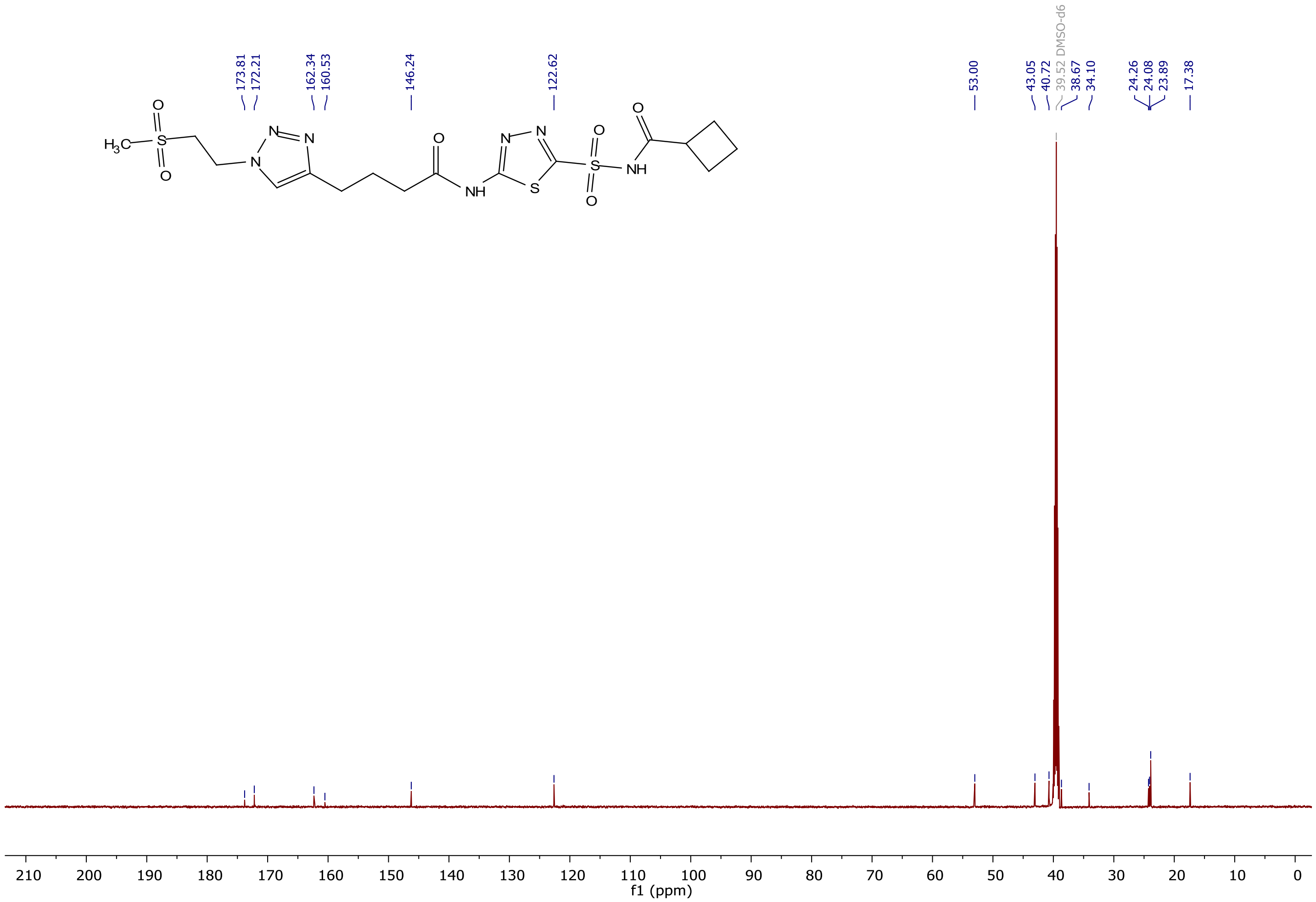

***N*-((5-(4-(1-(2-(Methylsulfonyl)ethyl)-1*H*-1,2,3-triazol-4-yl)butanamido)-1,3,4-thiadiazol-2-yl)sulfonyl)cyclobutanecarboxamide (4{45,5,78})** ^{13}**C NMR**

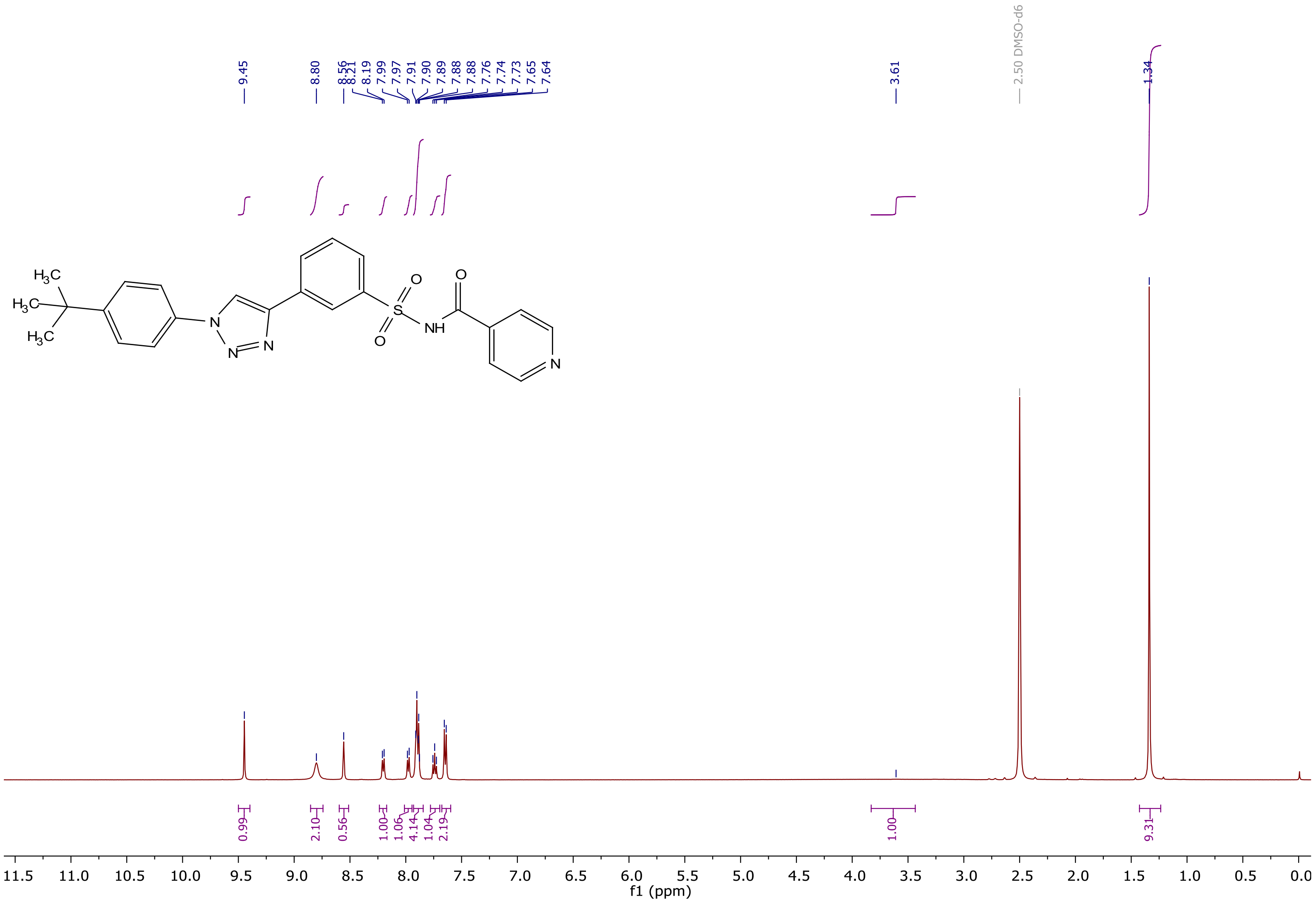

***N*-((3-(1-(4-(*tert*-Butyl)phenyl)-1*H*-1,2,3-triazol-4-yl)phenyl)sulfonyl)isonicotinamide (4{19,6,50}) ^{1}H NMR**

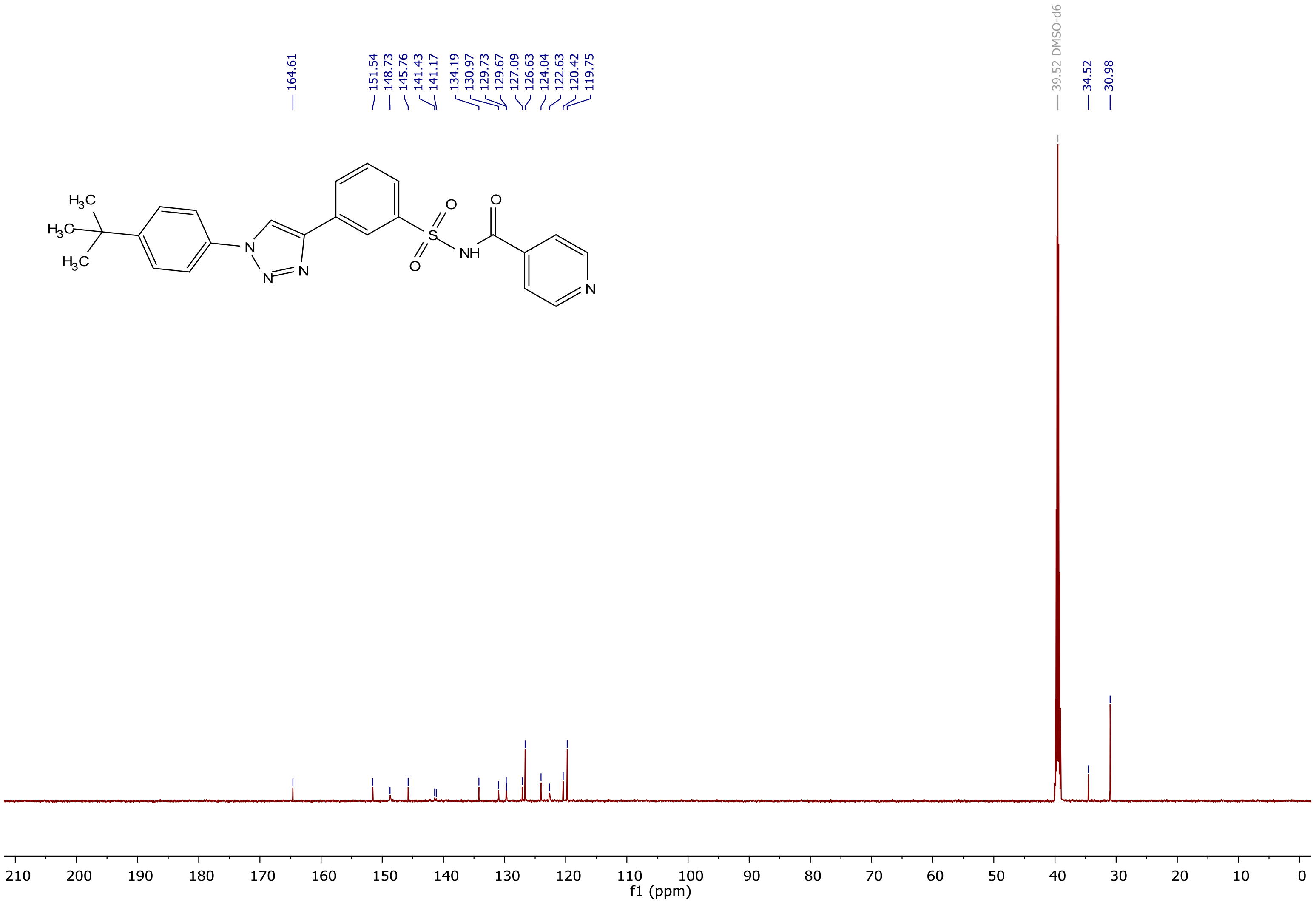

***N*-((3-(1-(4-(*tert*-Butyl)phenyl)-1*H*-1,2,3-triazol-4-yl)phenyl)sulfonyl)isonicotinamide (4{19,6,50}) ^{13}C NMR**

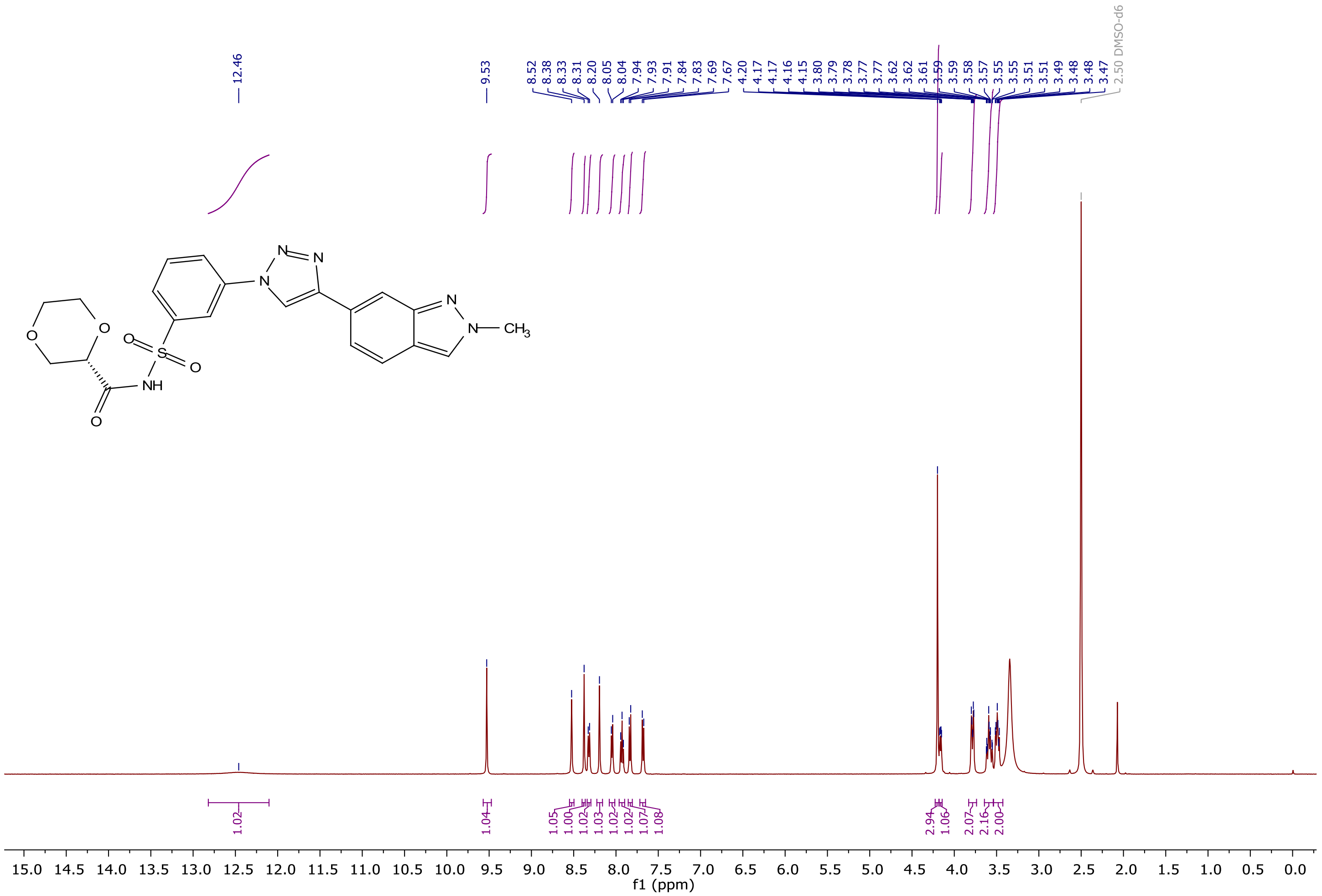

(*S*)-*N*-((3-(4-(2-Methyl-2*H*-indazol-6-yl)-1*H*-1,2,3-triazol-1-yl)phenyl)sulfonyl)-1,4-dioxane-2-carboxamide (7{2,20,59})

^{1}H NMR

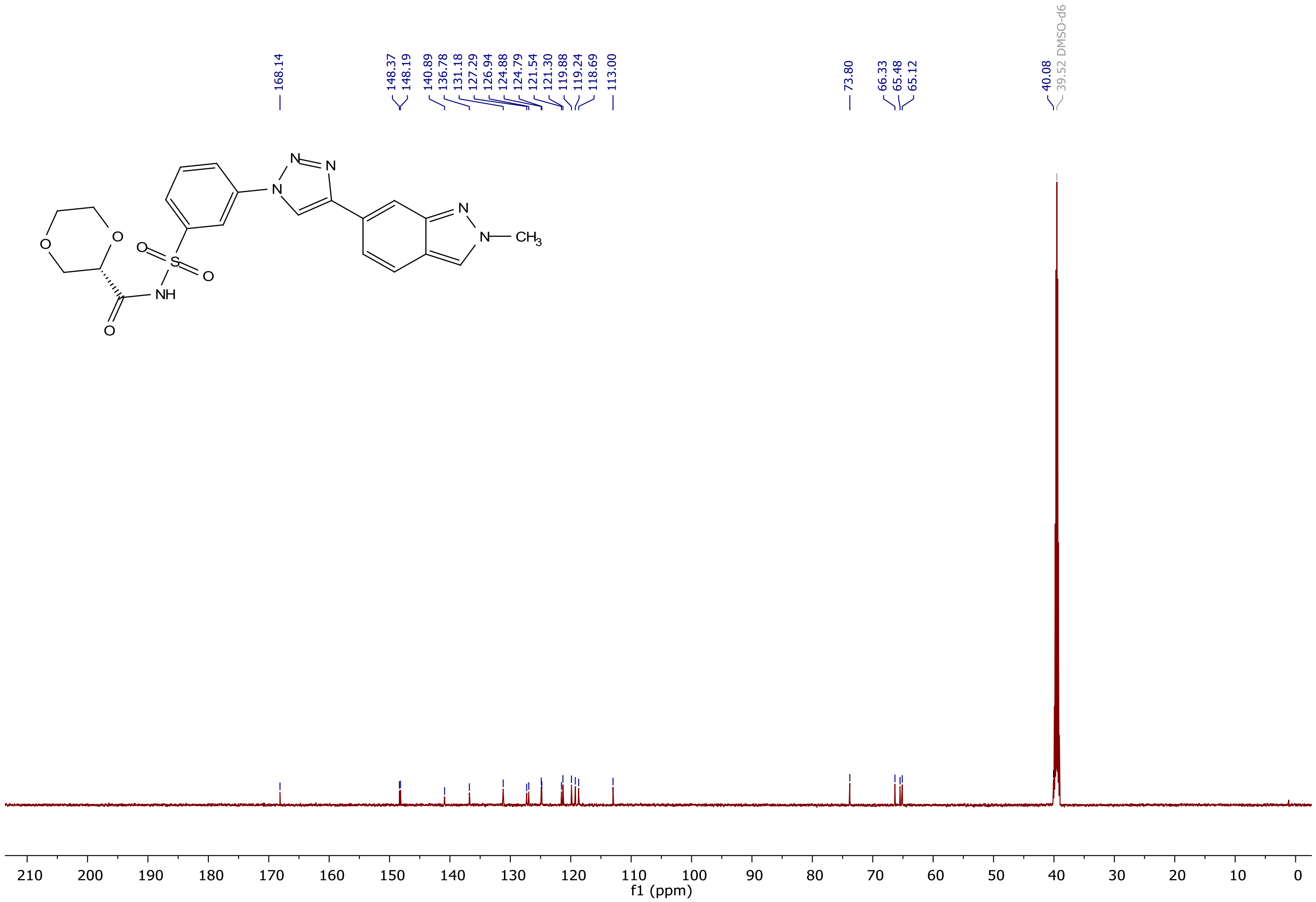

(*S*)-*N*-((3-(4-(2-Methyl-2*H*-indazol-6-yl)-1*H*-1,2,3-triazol-1-yl)phenyl)sulfonyl)-1,4-dioxane-2-carboxamide (7{2,20,59})

^{13}C NMR

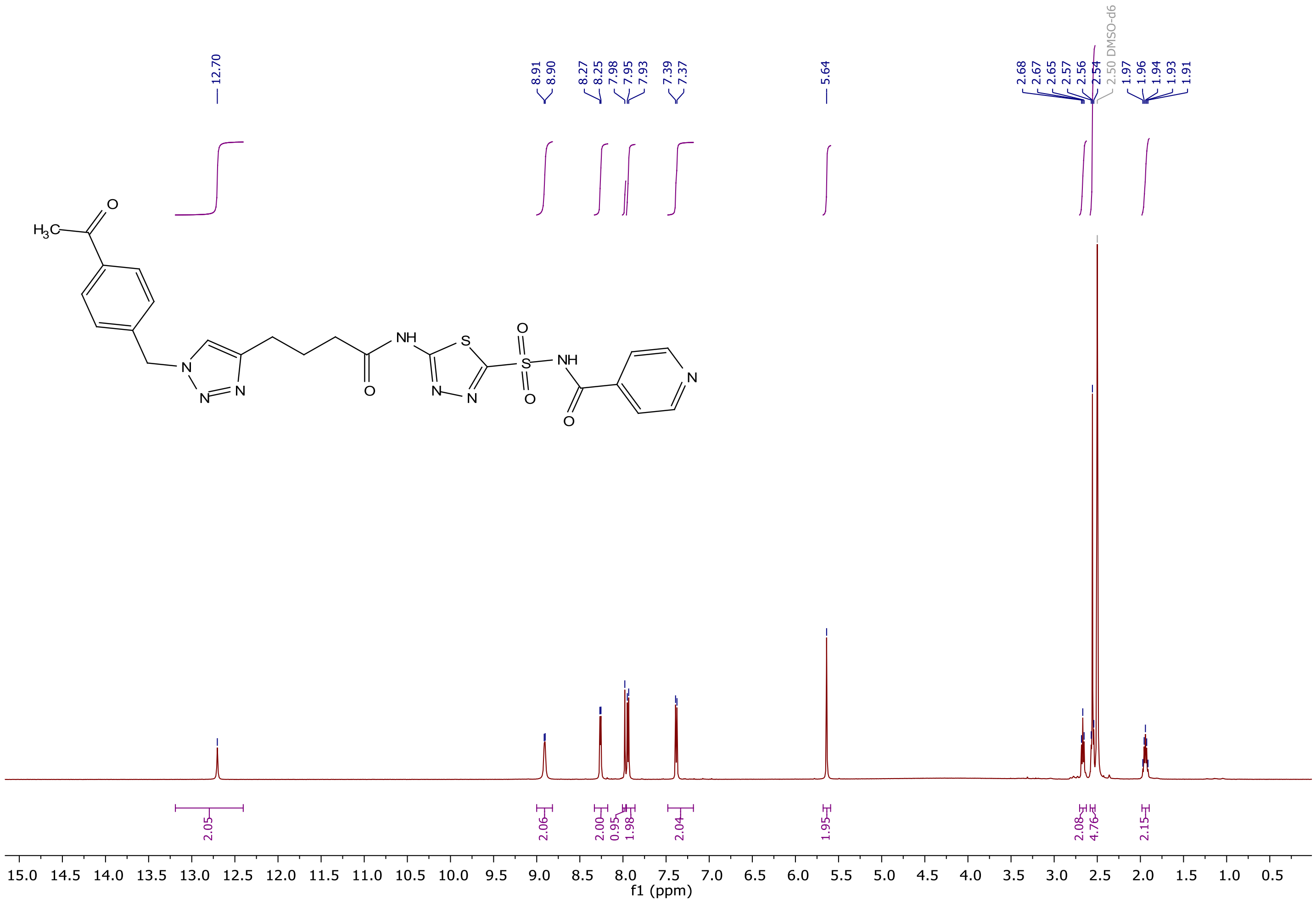

***N*-((5-(4-(1-(4-Acetylbenzyl)-1*H*-1,2,3-triazol-4-yl)butanamido)-1,3,4-thiadiazol-2-yl)sulfonyl)isonicotinamide (4{43,5,50})**

^{1}H NMR

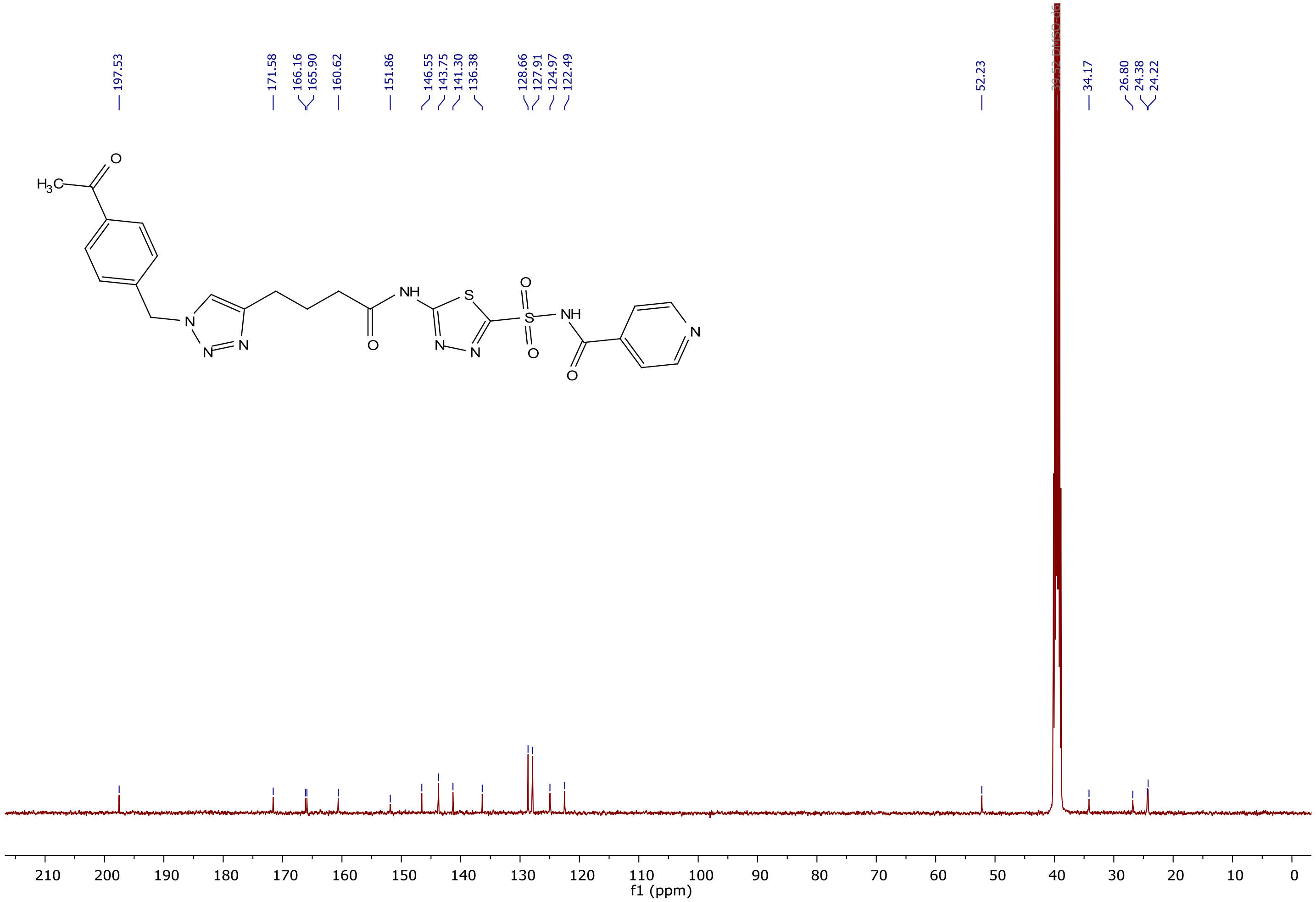

***N*-((5-(4-(1-(4-Acetylbenzyl)-1*H*-1,2,3-triazol-4-yl)butanamido)-1,3,4-thiadiazol-2-yl)sulfonyl)isonicotinamide (4{43,5,50})**

^{13}C NMR

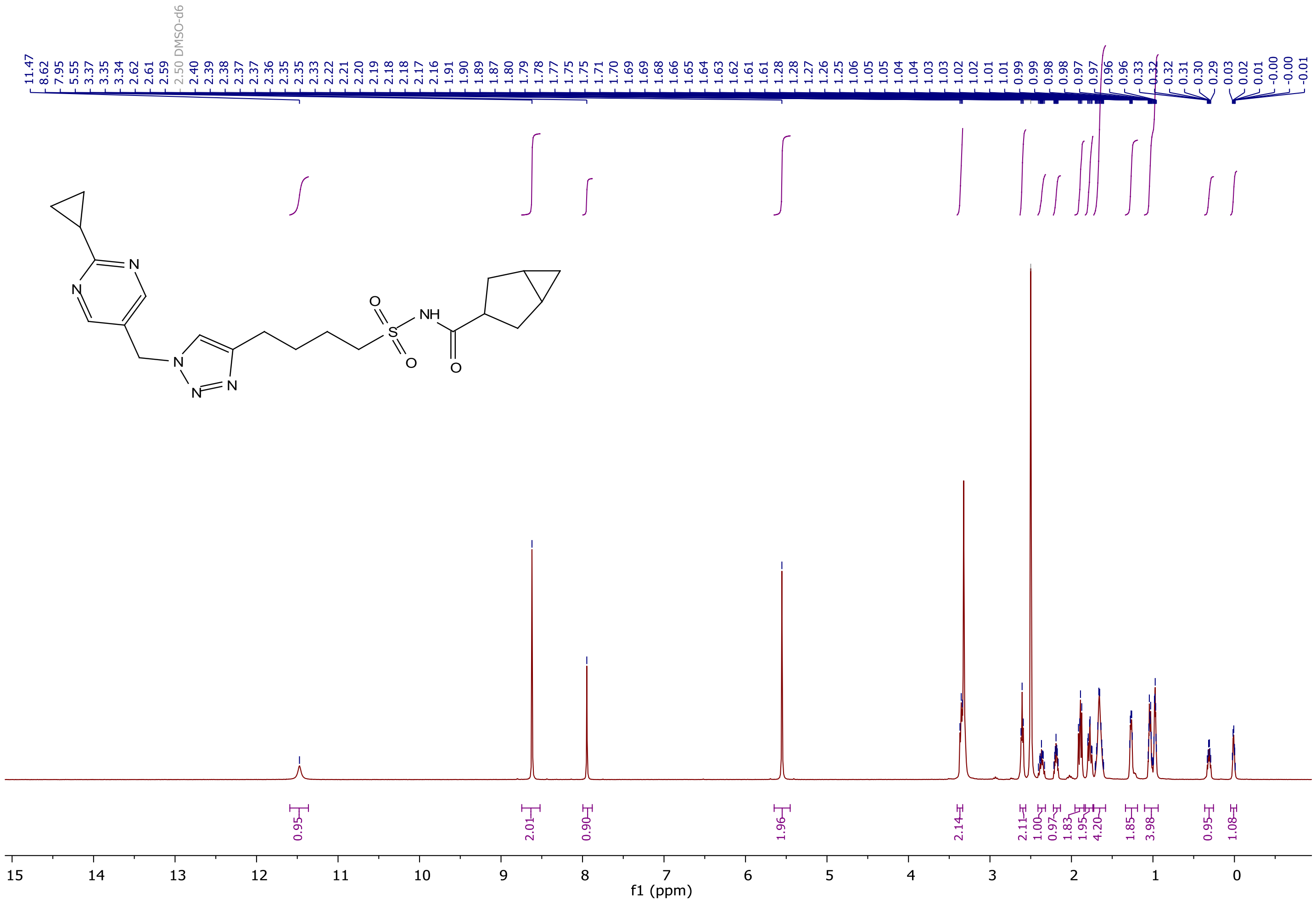

***N*-((4-(1-((2-Cyclopropylpyrimidin-5-yl)methyl)-1*H*-1,2,3-triazol-4-yl)butyl)sulfonyl)bicyclo[3.1.0]hexane-3-carboxamide (4{64,3,61}) ^{1}H NMR**

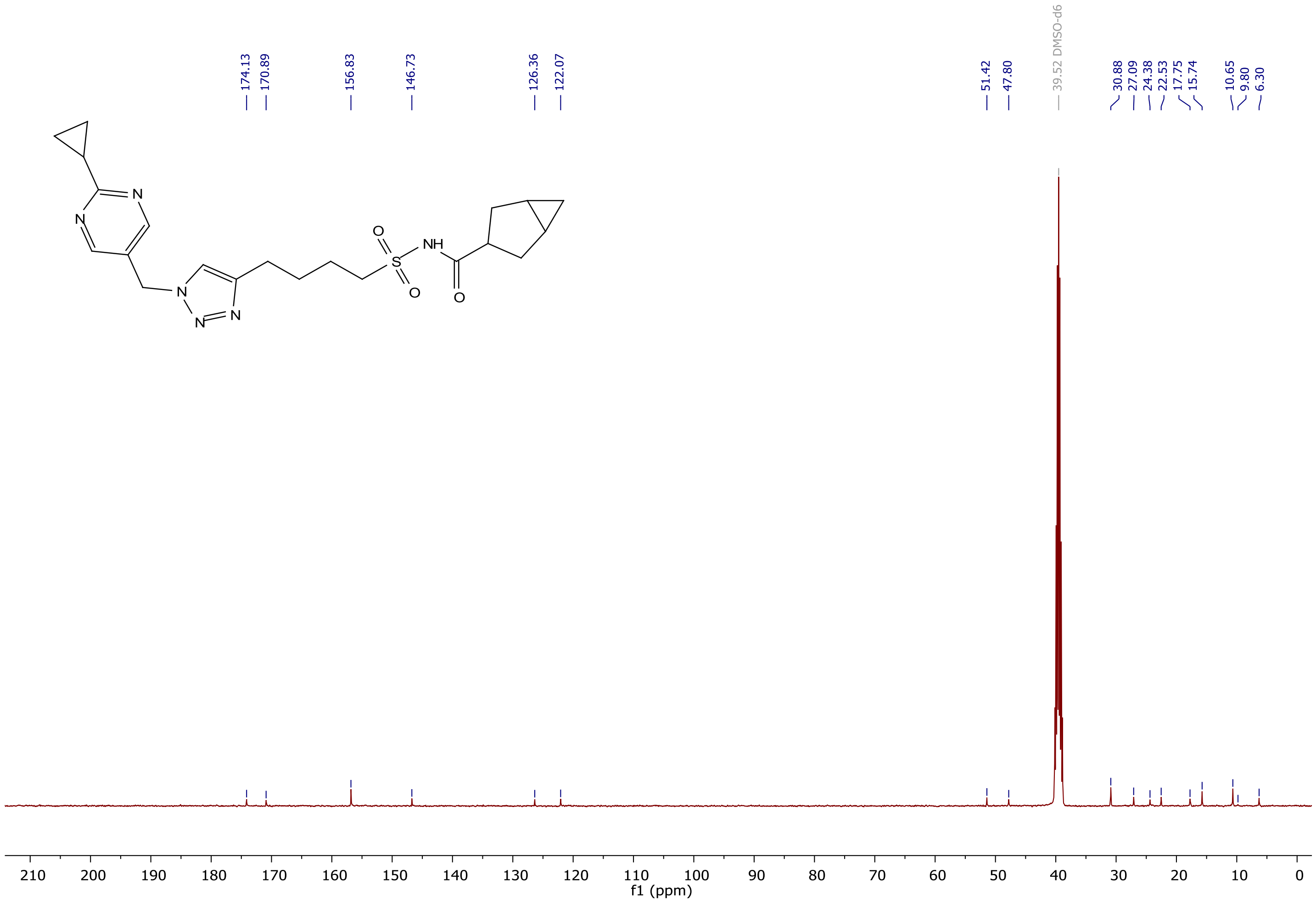

***N*-((4-(1-((2-Cyclopropylpyrimidin-5-yl)methyl)-1*H*-1,2,3-triazol-4-yl)butyl)sulfonyl)bicyclo[3.1.0]hexane-3-carboxamide (4{64,3,61}) ^{13}C NMR**

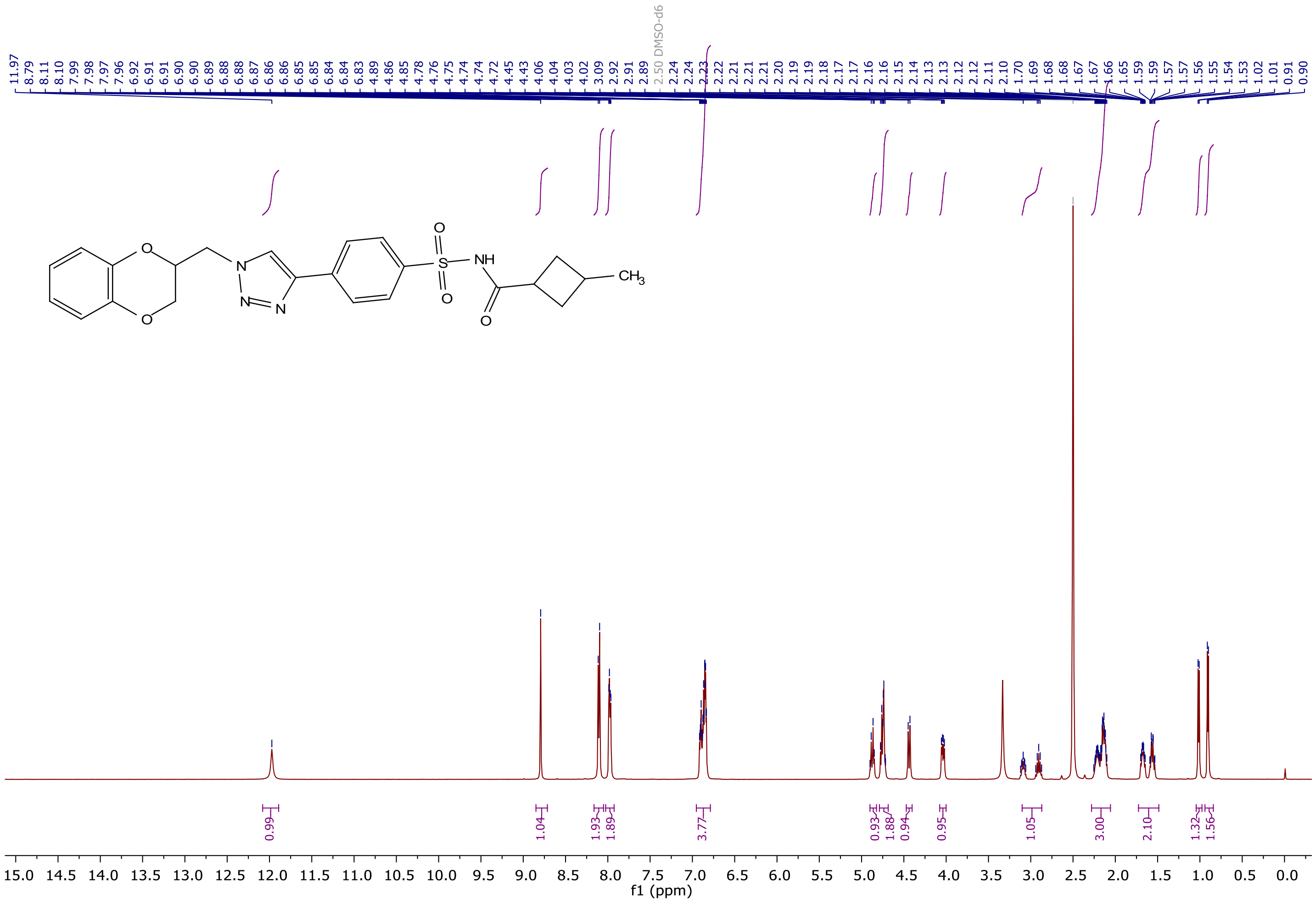

N-((4-(1-((2,3-Dihydrobenzo[b][1,4]dioxin-2-yl)methyl)-1H-1,2,3-triazol-4-yl)phenyl)sulfonyl)-3-methylcyclobutane-1-carboxamide (4{17,7,31}) ^{1}H NMR

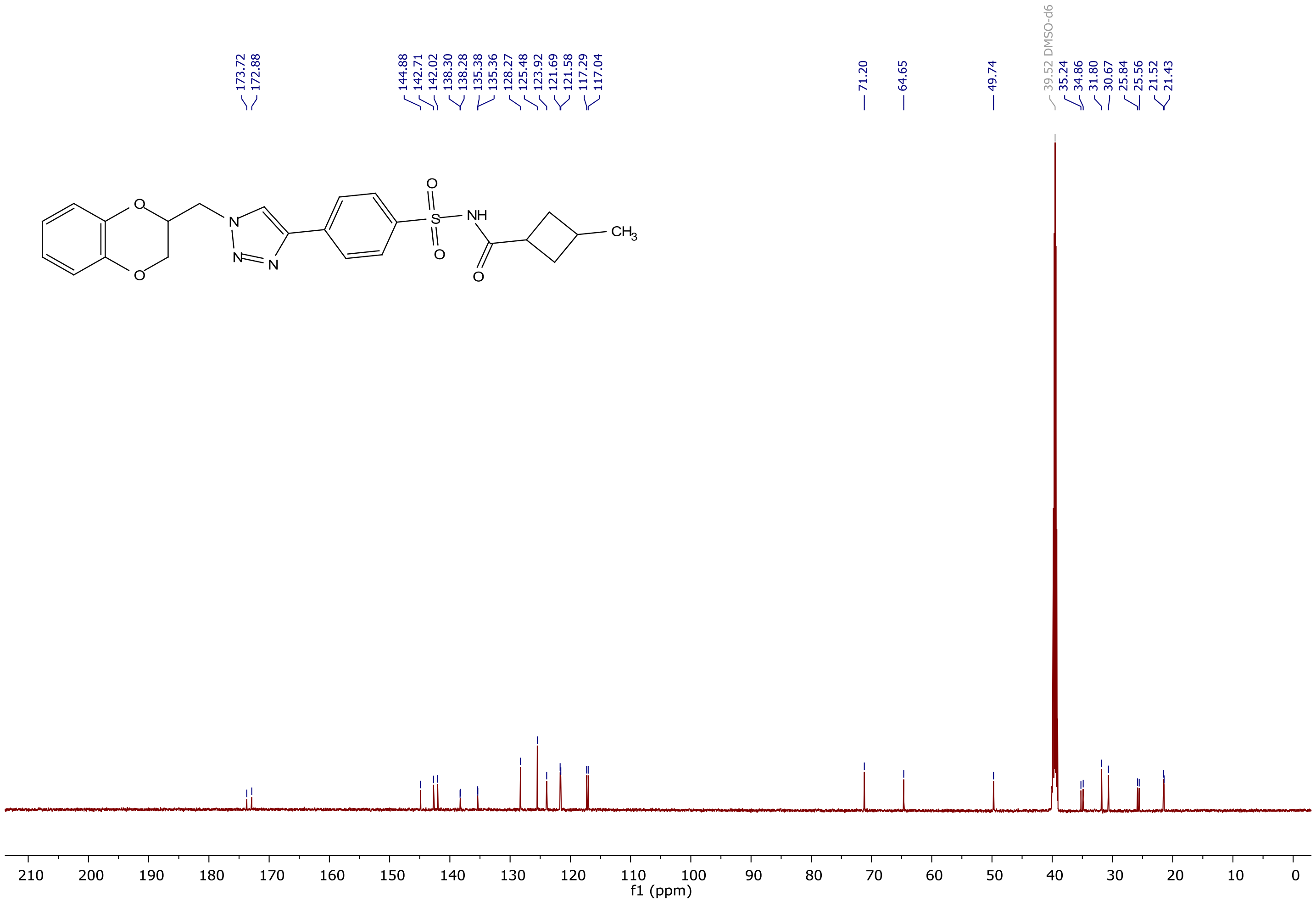

***N*-((4-(1-((2,3-Dihydrobenzo[*b*][1,4]dioxin-2-yl)methyl)-1*H*-1,2,3-triazol-4-yl)phenyl)sulfonyl)-3-methylcyclobutane-1-carboxamide (4{17,7,31}) ^{13}C NMR**

References

(1) Mahon, B. P.; Bhatt, A.; Socorro, L.; Driscoll, J. M.; Okoh, C.; Lomelino, C. L.; Mboge, M. Y.; Kurian, J. J.; Tu, C.; Agbandje-McKenna, M.; Frost, S. C.; McKenna, R. The Structure of Carbonic Anhydrase IX Is Adapted for Low-PH Catalysis. *Biochemistry* **2016**, *55* (33), 4642–4653. https://doi.org/10.1021/acs.biochem.6b00243.

(2) Banerjee, A. L.; Swanson, M.; Mallik, S.; Srivastava, D. K. Purification of Recombinant Human Carbonic Anhydrase-II by Metal Affinity Chromatography without Incorporating Histidine Tags. *Protein Expr. Purif.* **2004**, *37* (2), 450–454. https://doi.org/10.1016/j.pep.2004.06.031.

(3) Wu, G.; Yuan, Y.; Hodge, C. N. Determining Appropriate Substrate Conversion for Enzymatic Assays in High-Throughput Screening. *SLAS Discov.* **2003**, *8* (6), 694–700. https://doi.org/10.1177/1087057103260050.

(4) Behnke, C. A.; Le Trong, I.; Godden, J. W.; Merritt, E. A.; Teller, D. C.; Bajorath, J.; Stenkamp, R. E. Atomic Resolution Studies of Carbonic Anhydrase II. *Acta Crystallogr. Sect. D Biol. Crystallogr.* **2010**, *66* (5), 616–627. https://doi.org/10.1107/S0907444910006554.

(5) Kazokaitė, J.; Niemans, R.; Dudutienė, V.; Becker, H. M.; Leitāns, J.; Zubrienė, A.; Baranauskienė, L.; Gondi, G.; Zeidler, R.; Matulienė, J.; Tārs, K.; Yaromina, A.; Lambin, P.; Dubois, L. J.; Matulis, D. Novel Fluorinated Carbonic Anhydrase IX Inhibitors Reduce Hypoxia-Induced Acidification and Clonogenic Survival of Cancer Cells. *Oncotarget* **2018**, *9* (42), 26800–26816. https://doi.org/10.18632/oncotarget.25508.

(6) *https://www.molsoft.com*.

(7) Abagyan, R.; Totrov, M.; Kuznetsov, D. ICM—A New Method for Protein Modeling and Design: Applications to Docking and Structure Prediction from the Distorted Native Conformation. *J. Comput. Chem.* **1994**, *15* (5), 488–506. https://doi.org/10.1002/jcc.540150503.

(8) Abagyan, R.; Totrov, M. Biased Probability Monte Carlo Conformational Searches and Electrostatic Calculations for Peptides and Proteins. *J. Mol. Biol.* **1994**, *235* (3), 983–1002. https://doi.org/10.1006/jmbi.1994.1052.

(9) Abraham, M. J.; Murtola, T.; Schulz, R.; Páll, S.; Smith, J. C.; HeS, B.; Lindahl, E. GROMACS: High Performance Molecular Simulations through Multi-Level Parallelism from Laptops to Supercomputers. *SoftwareX* **2015**, *1–2*, 19–25. https://doi.org/10.1016/j.softx.2015.06.001.

(10) Valdés-Tresanco, M. S.; Valdés-Tresanco, M. E.; Valiente, P. A.; Moreno, E. Gmx_MMPBSA: A New Tool to Perform End-State Free Energy Calculations with GROMACS. *J. Chem. Theory Comput.* **2021**, *17* (10), 6281–6291. https://doi.org/10.1021/acs.jctc.1c00645.
